# Genetic architecture and selection of Anhui autochthonous pig population revealed by whole genome resequencing

**DOI:** 10.3389/fgene.2022.1022261

**Published:** 2022-10-17

**Authors:** Wei Zhang, Xiaojin Li, Yao Jiang, Mei Zhou, Linqing Liu, Shiguang Su, Chengliang Xu, Xueting Li, Chonglong Wang

**Affiliations:** Key Laboratory of Pig Molecular Quantitative Genetics of Anhui Academy of Agricultural Sciences, Anhui Provincial Key Laboratory of Livestock and Poultry Product Safety Engineering, Institute of Animal Husbandry and Veterinary Medicine, Anhui Academy of Agricultural Sciences, Hefei, China

**Keywords:** whole genome sequencing, SNP, signatures of selection, Anhui pig population, population structure

## Abstract

The genetic resources among pigs in Anhui Province are diverse, but their value and potential have yet to be discovered. To illustrate the genetic diversity and population structure of the Anhui pigs population, we resequenced the genome of 150 pigs from six representative Anhui pigs populations and analyzed this data together with the sequencing data from 40 Asian wild boars and commercial pigs. Our results showed that Anhui pigs were divided into two distinct types based on ancestral descent: Wannan Spotted pig (WSP) and Wannan Black pig (WBP) origins from the same ancestor and the other four populations origins from another ancestor. We also identified several potential selective sweep regions associated with domestication characteristics among Anhui pigs, including reproduction-associated genes (CABS1, INSL6, MAP3K12, IGF1R, INSR, LIMK2, PATZ1, MAPK1), lipid- and meat-related genes (SNX19, MSTN, MC5R, PRKG1, CREBBP, ADCY9), and ear size genes (MSRB3 and SOX5). Therefore, these findings expand the catalogue and how these genetic differences among pigs and this newly generated data will be a valuable resource for future genetic studies and for improving genome-assisted breeding of pigs and other domesticated animals.

## 1 Introduction

Domestic pig (*Sus scrofa*) is an important livestock species, and has served as a source of stable food, organic fertilizer, industrial raw material, and medicine for humans since its domestication in the early Neolithic period. Since then, the domestic pig has promoted population growth and socioeconomic transformation from a hunter-gatherer society to sedentary agricultural settlements. Through domestication, natural and artificial selection has generated 48 pig breeds in China (China National Commission of Animal Genetic Resources. 2011). Among evolutionary biologists, there is substantial interest in elucidating how natural and artificial selection have shaped modern pig genomes and the how these genetic differences contribute to the complex phenotypes seen among pigs.

Whole-genome resequencing has the potential to resolve pig population structure and identify phenotype-associated functional genes that arose during domestication and breeding of different pig breeds. In recent years, whole-genome sequencing studies on germplasm characteristics among different pig breeds have revealed the evolutionary history of the pig population and elucidated the genetic basis of complex phenotypes, while also providing new focuses in the future breeding of Chinese indigenous pigs (Rubin et al., 2012; Li et al., 2013; Ai et al., 2015; Frantz et al., 2015). Several causative genes have been reported as responsible for phenotypic variation among Chinese indigenous pigs. For instance, HIFA has been identified as the gene responsible for hypoxic adaptation in Tibetan pigs (Li et al., 2013). Moreover, TGFB3 and DAB2IP have been shown to be important in regulating the number of ribs (Zhu et al., 2015). Additionally, MITF, EDNRB, and MC1R have been identified as the genes responsible for color variations among Chinese breeds (Fang et al., 2009; Wang et al., 2015; Zhao et al., 2018). However, most of the aforementioned studies focused only on a few phenotypes among a limited number of breeds or populations. Therefore, considering that China has such a large and a diverse number of pig breeds, more genes with germplasm characteristics still need to be identified.

The Anhui Province, located in the Yangtze River Delta, is one of the important birthplaces of Chinese civilization. Additionally, the province is home to a wide variety of indigenous pig populations, including the Dingyuan pig (DYP), Huoshou Black pig (HBP), Wannan Spotted pig (WSP), Wannan Black pig (WBP), Anqing Six-end-white pig (ASP), and Wei pig (WP). These pig breeds are representative autochthonous Chinese breeds with long histories of breeding in the country. Of mention is that Anhui indigenous pig populations are facing crises of population decline and loss of genetic characteristics. The reasons for this are complex, including long cycles of feeding, the introduction of modern commercial pigs, etc. however, insufficient protection is the most direct cause. To date, there have been no systematic studies (i.e., based on a large panel of Anhui indigenous pig populations) seeking to illustrate the genomic diversity and signatures of selection among Anhui indigenous pig populations. Therefore, a comprehensive study of the genetic diversity, phylogenetic relationships, population structure, demographic history, and selection signatures among the Anhui pig populations are critical. Furthermore, the Anhui pig populations represent unique genetic resources for understanding the role of functional diversity, which can be applied in other contexts as well. Moreover, characterizing genetic variations and identifying the associated phenotypes are vital for future breeding of indigenous pig populations.

For this purpose, we performed whole-genome sequencing of six representative pig populations in Anhui (WSP, *n* = 25; WBP, *n* = 25; ASP, *n* = 25; WP, *n* = 25; DYP, *n* = 25; HBP, *n* = 25). Using whole-genome sequencing data in conjunction with downloaded sequence data, we explored the genetic diversity, phylogenetic relationships, population structure, and demographic history of the six pig populations. In addition, we calculated the F_ST_ (fixation index) and log_2_ (θπ ratio) values to elucidate the signatures of selection and then characterized these candidates’ genes are present in the genome of Anhui pigs. Our findings provide valuable insights into the evolutionary history of Anhui pig populations and reveal several promising candidate genes for future indigenous pig breeding.

## 2 Materials and methods

### 2.1 Sample collection and genome sequencing

**FIGURE 1:**
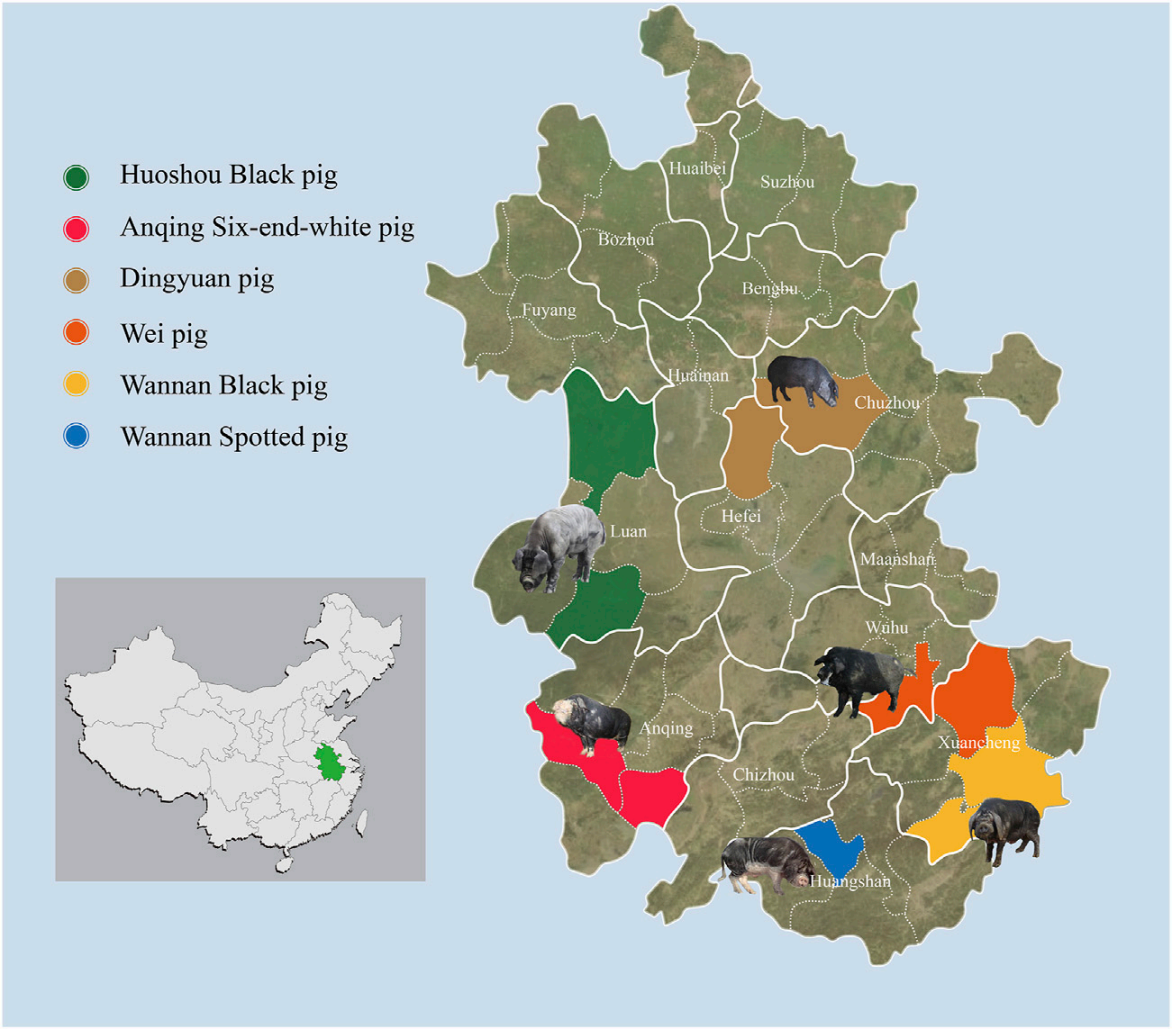
Geographic distribution and appearances of typical pigs. 150 Anhui indigenous pigs representing six populations were included.

To survey the overall genetic diversity among autochthonous pig breeds in Anhui province, blood samples from 150 individuals within six indigenous Anhui pig populations were collected from pig conservation farms (Figure 1; Supplementary Table S1). These breeds included: ASP (*N* = 25, ♀15, ♂10), WSP (*N* = 25, ♀10, ♂15), WBP (*N* = 25, ♀11, ♂14), WP (*N* = 25, ♀13, ♂12), DYP (*N* = 25, ♀4, ♂21), HBP (*N* = 25, ♀10, ♂15). This study was conducted in accordance with and was approved by the Animal Care Committee of the Anhui Academy of Agricultural Sciences (Hefei, China; no. AAAS2020-04). For the blood samples, genomic DNA was extracted using the standard phenol–chloroform method (Sambrook and Russel 2001) and stored at 4°C to avoid freeze–thawing. The quality and concentration of the DNA were assessed using a 0.5% agarose gel (run for >8 h at 25 V) and a Nanodrop spectrophotometer (Thermo Fisher Scientific). DNA was then fragmented and treated in accordance with the Illumina DNA sample preparation protocol. Sequencing libraries were constructed and sequenced on the Illumina NovaSeq 6000 platform (Illumina, San Diego, CA, United States) using paired-end 150 bp reads through the Novogene service (Beijing, China).

For comparison, 40 pig resequenced data sets from the National Center for Biotechnology Information (https://www.ncbi.nlm.nih.gov/sra/) were downloaded (Ai et al., 2015; Rubin et al., 2012; Zhao et al., 2018; Groenen et al., 2012) (Supplementary Table S2), including Asian wild boar, Duroc, Pietrain, Yorkshire, and Landrace.

### 2.2 Read mapping and variant calling

Before alignment, adapter and low-quality reads [e.g., reads with 10% unidentified nucleotides (*N*); reads with >10 nt aligned to the adapter; reads with >30% bases with Phred quality ≤25] were filtered using the NGSQCToolkit_v2.3.351. The remaining, high-quality reads were aligned with the pig reference genome (ftp://ftp.ensembl.org/pub/release-99/variation/gvf/sus_scrofa/) using BWA-0.7.12 software (https://sourceforge.net/projects/bio-bwa/) default parameters (Li and Durbin. 2009). For the mapping process, a BAM file index was built using SAMtools (https://github.com/samtools/samtools/releases/) (Li et al., 2009). The BAM file was sorted using the Picard-tools-1.105 software (https://github.com/broadinstitute/picard/releases). Duplicate read data were excluded. Next, we performed variant calling for all samples on a population-scale using SAMtools mpileup with the parameters “-m 2 -F 0.002 -d 1000” and then bcftools to view variants (Li and Durbin 2011). We further resolved the SNPs according to the following criteria: QD (Variant Confidence/Quality by Depth) < 5.0, MQ (RMS Mapping Quality) < 40.0, FS (Phred-scaled *p* value using Fisher’s exact test to detect strand bias) > 60.0, SOR (Strand Odd Ratio) > 3.0, MQRankSum (Z-score from Wilcoxon rank sum test of Alt vs. Ref read mapping qualities) < −10.0, ReadPosRankSum (Z-score from Wilcoxon rank sum test of Alt vs. Ref read position bias) < −8.0 and QUAL < 30. SNPs that were bi-allelic missed >40% of calls and had a MAF < 0.05 were removed and the remaining SNPs comprised the basic set. Variants were annotated using ANNOVAR (Wang et al., 2011). The downloaded resequencing data were analyzed using the process described above.

### 2.3 Genomic diversity and population genetic structure

Nucleotide diversity (θ_π_) was calculated based on the maximum likelihood estimates of the fold-site frequency spectrum using a sliding window approach (50-kb windows with 50-kb steps; Kim et al., 2011). To illustrate the population structure, principal component analysis was performed using the GCTA software (v.1.25; Yang et al., 2011). Additionally, phylogenetic trees were inferred through the neighbor-joining (NJ) method and implemented in TreeBest (http://treesoft.sourceforge.net/treebest.shtml) with 1000 bootstraps. The population structure was deduced using the ADMIXTURE software (https://dalexander.github.io/admixture/index.html) with a kinship (K) set from 2 to 6 (Alexander et al., 2009).

### 2.4 Pairwise sequential Markovian coalescence analysis

The demographic history of the six Anhui indigenous pig populations was examined using the hidden Markov model approach and implemented according to the pairwise sequential Markovian coalescence (PSMC) model (Li and Durbin 2011). To estimate the distribution time, we used the parameters of *g* = 5 and a rate of 2.5 × 10^−8^ mutations per generation.

### 2.5 Selective sweeps analysis

To identify genetic variants associated with adaptation of the Anhui pig populations, comparisons were performed between three populations: 1) the Anhui pig populations versus the Asian wild boar, and 2) the Anhui pig populations versus the commercial pig population. The θ_π_ and F_ST_ were calculated using a 40-kb sliding window approach with a 20-kb step-size, in PopGenome (Pfeifer et al., 2014). The overlapping windows within the top 1% or 5% of the F_ST_ and θπ ratio (log_2_-transformed) distributions were considered putative selective regions (Li et al., 2013). Gene contents in “significant” genomic regions were retrieved from the Ensembl Genes 102 Database and analyzed in the BioMart software (http://asia.ensembl.org/biomart/martview/). To further explore the potential biological significance of the genes within these sweep regions, Gene ontology (GO) and Kyoto Encyclopedia of Genes and Genomes (KEGG) pathway analyses were conducted to perform functional annotation for these genes using KOBAS 3.0 (http://kobas.cbi.pku.edu.cn/). The terms and pathways exhibiting *p*-values < 0.05 were considered significant.

## 3 Results

### 3.1 Whole-genome sequencing and genetic variations

In total, 150 individuals from six pig populations indigenous to Anhui were selected for whole-genome resequencing. In total, 31 billion reads (or ∼4,678 Gb of sequences) were generated. Average data sets consisted of 31.2 Gb (10.85 X) per individual and 800.36, 750.16, 724.8, 797.18, 834.25, and 771.53 Gb for the HBP, ASP, WSP, DYP, WBP, and WP collective populations, respectively. We also downloaded data for 40 non-indigenous individuals (Asian wild boars, Duroc, Landrace, Yorkshire, and Pitelan), resulting in 5,732 Gb of data in total. Using BWA, reads were aligned to the pig reference genome with an average alignment rate of 98.49% ± 0.14% and an estimated error rate of 0.03% ± 0.005% (Supplementary Tables S3, S4). After stringent variant calling and filtering, approximately 23 million single nucleotide polymorphisms (SNPs) were identified. For all detected SNPs, the average transition/transversion ratio was 2.42, which is consistent with a previous study (Kerstens et al., 2009). Further annotation of these SNPs revealed that they were most abundant in intergenic regions (53.58%) and intronic regions (42.97%), followed by downstream (0.62%) and upstream (0.56%) regions, whereas only 0.7% were located in coding sequences. Most variations were located in non-coding sequences, indicating that during domestication and breeding, the non-coding sequences could potentially change protein function by regulating gene expression.

### 3.2 Genomic diversity and population genetic structure

**FIGURE 2:**
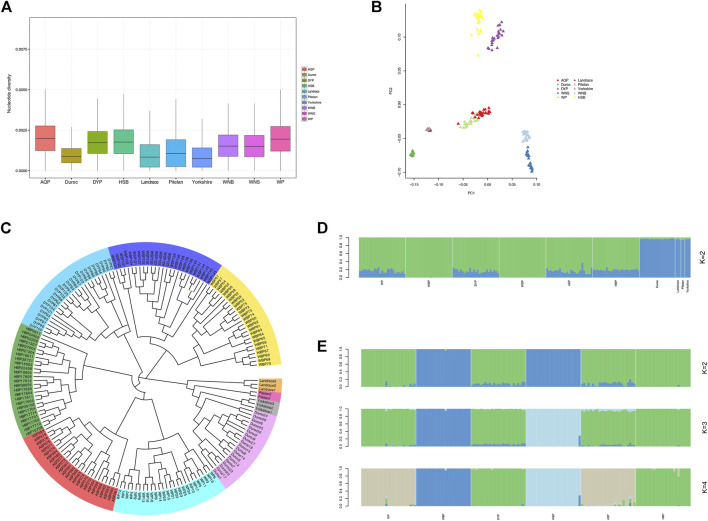
Population structure and relationship of Anhui in comparison to commercial pig. **(A)** Genome-wide distribution of nucleotide diversity among Anhui pig breeds and four commercial pig breeds with a 50-kb window. **(B)** Principal component analysis of Anhui pig breeds and four commercial pig breeds, PC1 against PC2. **(C)** Neighbor-joining tree of the relationships between the ten pig populations. The scale bar represents the identity-by-state (IBS) score between pairs of animals. **(D)** Proportion of ancestry for each individual assuming different number of ancestral population (*K* = 2). Colors in each vertical line represent the likelihood proportion of an animal genome assigned to a source population. **(E)** Proportion of ancestry for each individual in Anhui pig populations.

Nucleotide diversity reflects the degree of polymorphism within a population (Nei and Li. 1979). On a genome-wide window scale of 50-kb, with steps of 50-kb, the foreign pig breeds displayed reduced nucleotide diversity compared with the indigenous Anhui populations (Figure 2A). This is likely the result of extensive artificial selection over generations. We performed PCA analysis for the experimental populations in GCTA (Figure 2B). This analysis was successful in separating breed clusters based on genotypic data, where the foreign breeds, ASP and WP, HBP and DYP, and WBP and WSP were clustered together. These findings are consistent with the established information on the Anhui populations. To elucidate the phylogenetic relationship among the Anhui pig breeds, unrooted phylogenetic trees were constructed, revealing genetically distinct clusters (Figure 2C). This was consistent with the results of the PCA analysis, revealing clustering into distinct genetic groups. We used the ADMIXTURE software to determine the degree of admixture when K is increased from 2 to 6, where K is the assumed number of ancestral populations. *K* = 2 was suggested as the most plausible number among our samples (Figure 2D), reflecting the divergence of the Anhui populations and commercial breeds within the pig population. These results show that the current pig populations (WP, DYP, ASP, and HBP) may have some of the same genetic information as foreign pigs, possibly owing to the introduction of modern commercial pigs. We also conducted an admixture analysis of the Anhui populations (Figure 2E), and the results showed that *K* = 2 was the probable number of genetically distinct groups within the Anhui populations. It was revealed that WSP and WBP originated from the same ancestor and the other four populations from another ancestor. At *K* = 3, WBP was separated from WSP, and the others still descended from the same ancestor. At *K* = 4, WBP was still distinct from WSP, WP was grouped with ASP, and DYP was grouped with HBP, in agreement with prior knowledge of Anhui pig populations.

### 3.3 Demographic history

**FIGURE 3:**
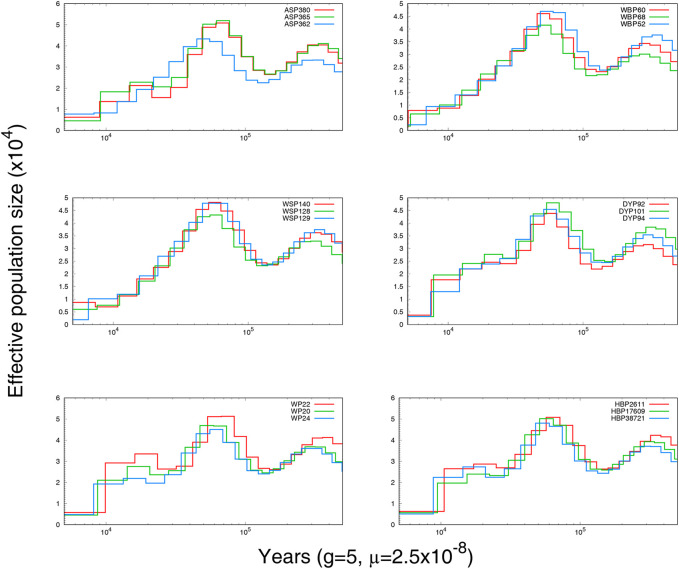
The effective population size and history of Anhui pig.

To explore the demographic histories of the six Anhui pig populations, PSMC was used to infer the historical population size. The variations in effective population size (Ne) over time are shown in Figure 3. Individuals from the same breed displayed similar historical fluctuations in effective population size. All breeds shared a collective population decline beginning at approximately 10,000 BP, likely a consequence of Neolithic domestication events (Zeder, 2008; Boitard et al., 2016).

#### 3.3.1 The selective regions of Anhui pig populations compared to those of the Asian wild boar population

**FIGURE 4:**
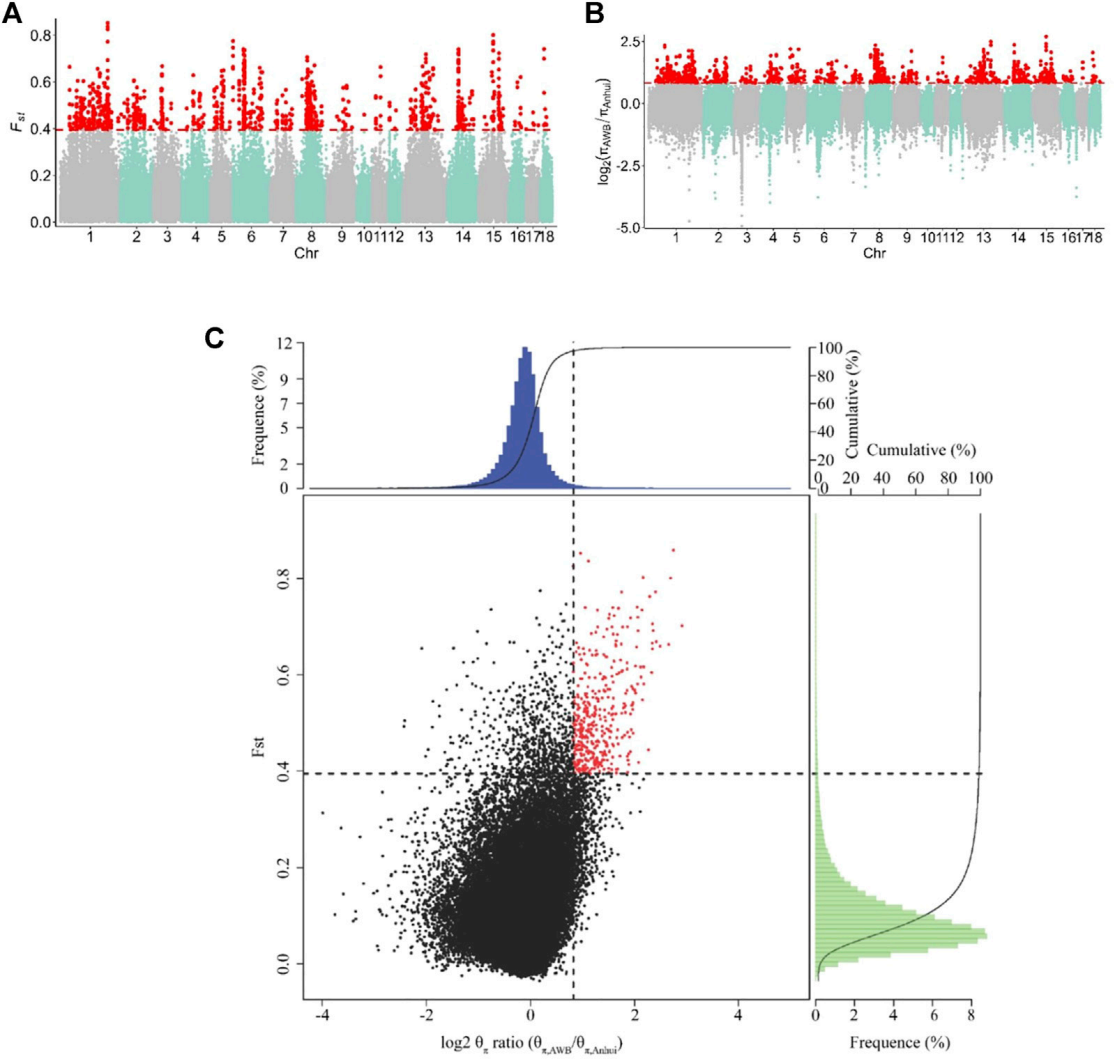
Identification of genomic regions with strong selective sweep signals in Anhui six pig population, which are calculated in a 40-kb sliding window approach with 20 kb step-size. **(A)** Distribution of FST values among autosome chromosomes. The red line represents the 0.01 level. **(B)** Distribution of log_2_ (θπ ratio) among autosomal chromosomes. The red line represents the 0.01 level. **(C)** The final selection regions based on two statistics. Points located to the right of the vertical dashed lines (corresponding to 1% right tails of the empirical log_2_ (θπ ratio) distribution, where log_2_ (θπ ratio) is 0.824992) and above the horizontal dashed line (1% above tail of the empirical FST distribution, where FST is 0.395220) were identified as selected regions for Anhui pig population (red points). The AWB refer to Asia wild boar, the Anhui refer to Anhui pig population.

To identify the signatures of selection, we compared the genomes of Anhui pig populations to those of Asian wild boars. Specifically, we used both θπ- and F_ST_-based cross approaches to investigate the signatures of selection. For this study, the regions within the top 1% were defined as the selected regions. The genome distribution of the two statistics were shown in Figures 4A,B. A total of 402 selective regions harboring 187 genes were identified in the top 1% of the F_ST_ and log_2_ (θπ, ratios) distributions (16.1 Mb of the genome, Figure 4C and Supplementary Tables S5, S6). All 187 genes were used for GO and KEGG analyses (Supplementary Tables S7, S8 and Supplementary Figures S1, S2). Functional annotation revealed that the selected genes may have effects on reproduction (CABS1, INSL6, MAP3K12, and MAPK1) and lipid- and meat-related processes (SNX19 and MSTN).

#### 3.3.2 The selective regions of Anhui pig populations compared to those of the commercial pig population

**FIGURE 5:**
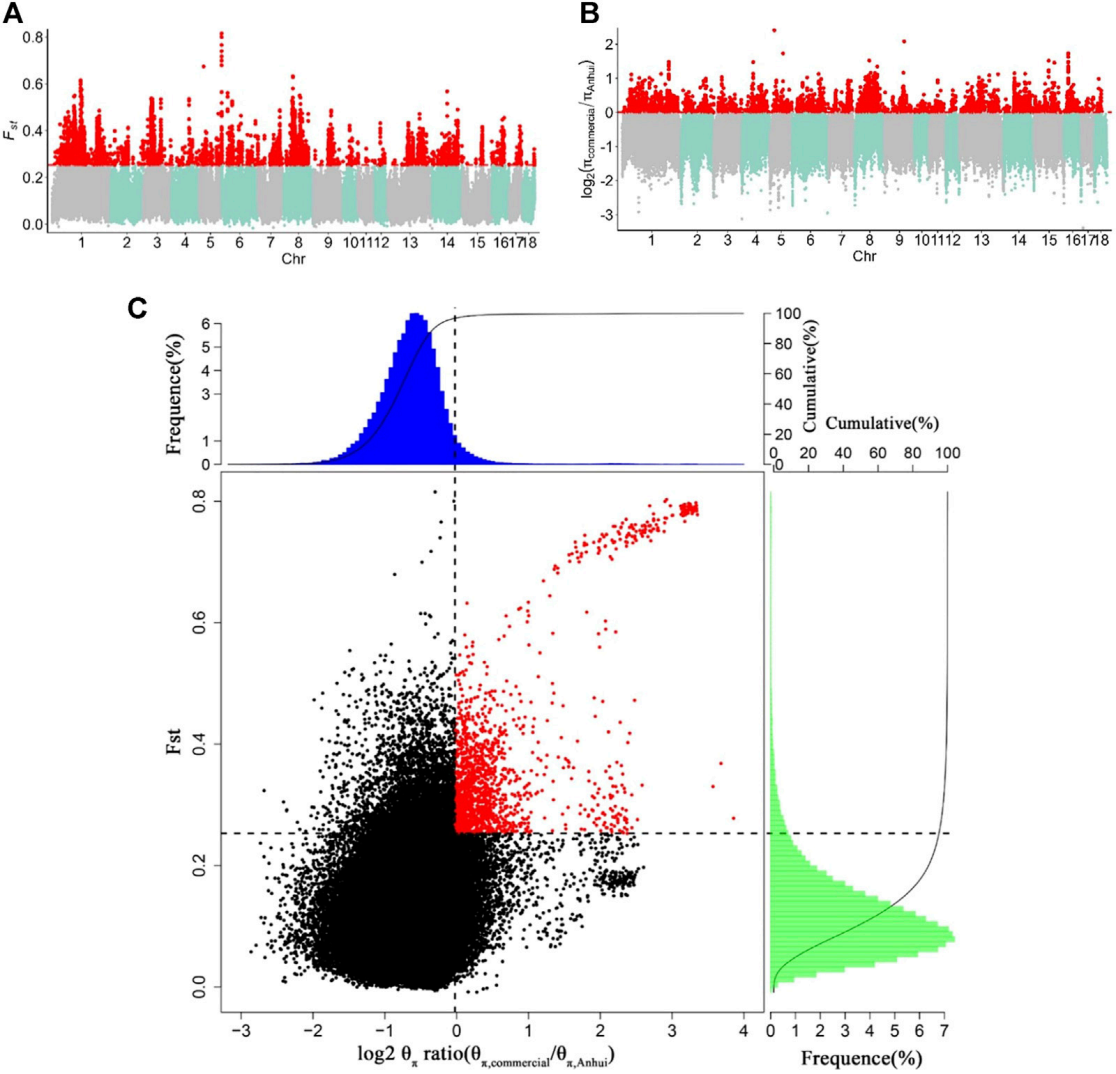
Identification of genomic regions with strong selective sweep signals between Anhui six pig population and four commercial pig population, which are calculated in a 40-kb sliding window approach with 20 kb step-size. **(A)** Distribution of F_ST_ values among autosome chromosomes. The red line represents the 0.05 level. **(B)** Distribution of log_2_ (θπ ratio) among autosomal chromosomes. The red line represents the 0.05 level. **(C)** The final selection regions based on two statistics. Points located to the right of the vertical dashed lines (corresponding to 5% right tails of the empirical log_2_ (θπ ratio) distribution, where log_2_ (θπ ratio) is 0.0.0011) and above the horizontal dashed line (5% above tail of the empirical FST distribution, where FST is 0.2527) were identified as selected regions for Anhui pig population (red points). The commercial refers to four commercial pigs, the Anhui refer to Anhui pig population.

To capture potential genes that underwent divergent selection between the Anhui and commercial pig populations, we estimated the genome-wide values of log_2_ (θπ ratio) and FST based on the whole-genome resequencing data of 177 samples from ten pig populations. The genome distribution of the two statistics were shown in Figures 5A,B. Using the top 5% FST cutoff (FST ≥ 0.2527) and log_2_ (θπ ratio) cutoff [log_2_ (θπ ratio) ≥ 0.0011], we detected nine hundred and sixty-seven selective regions containing 331 candidate genes (38.7 Mb of the genome; Figure 5C and Supplementary Tables S9, S10). All 331 genes were used for GO and KEGG analyses (Supplementary Figures S3, S4; Supplementary Tables S11, S12). Functional enrichment analyses revealed that the selected genes may play an important role in reproduction (*CABS1*, *MAPK1*, *IGF1R*, *INSR*, *LIMK2*, and *PATZ1*), meat quality and fat deposition capacity (*MC5R*, *MSTN*, *PRKG1*, *CREBBP*, and *ADCY9*), and ear size (*MSRB3* and *SOX5*).

## 4 Discussion

The pig, as one of the first domesticated animals, plays an important role in many aspects of human life. In recent years, the continued protection and utilization of the biological seed industry has attracted significant attention in China. The successively implemented “The third national census of livestock and poultry genetic resources” and “Precise identification of livestock and poultry germplasm resources” could help us to better understand the differences among pig breeds, and to protect and utilize the unique Chinese pig germplasm. To systematically analyze the genetic relationships, genetic diversity, population structure, and selection signatures resulting from natural and artificial selection in the Anhui pig populations, 150 unrelated samples were collected from six pig breeds along with sequencing data from 40 foreign pigs. In this study, the genetic variation in the Anhui pig populations was surveyed, using blood samples from pig conservation farms, from which DNA was isolated and sequenced in order to construct a genetic conservation system. Going forward, this information can serve to guide the protection and utilization of indigenous pig breeds in Anhui. Moreover, the genetic structure of this population has also been elucidated. The WSP and WBP descended from the same ancestor, and the other four breeds from another ancestor. We also found that the Anhui pig populations exhibited a highly similar pattern of historical fluctuation in effective population size at approximately 10,000 BP, which is likely the consequence of Neolithic domestication events. In addition, several genes have been found to be associated with important economic traits.

### 4.1 The selected genes of Anhui pig populations compared to those of the Asian wild boar population

Domestic pigs have higher fertility rates than those of wild boars. GO analysis revealed several genes related to reproduction, including those related to reproductive process (GO: 0022414, five genes), reproduction (GO: 0000003, six genes), and fertilization (GO: 0007283, two genes). The acrosome reactions decreased significantly after the porcine capacitated sperm were treated with anti-pCABS1 antiserum, suggesting that porcine expression of CABS1 plays an important role in acrosome reactions (Shawki et al., 2016). In this study, two variants of the CABS1 gene were identified (Supplementary Table S13; exon1: c; G1047T: p.S349S; exon1:c.C1069G: p.L357V). Notably, the “exon1:c.C1069G: p.L357V” variant results in a Leu to Val substitution, which may in turn alter the expression of CABS1. The CABS1 gene was identified as a selected gene in ASP in our previous study (Zhang et al., 2020), which suggests that CABS1 is commonly selected within the Anhui pig populations. Another gene involved in reproduction, insulin-like 6 (INSL6), is a member of the insulin superfamily that plays an important role in the progression of spermatogenesis. Moreover, a deficiency in INSL6 can result in varying levels of male infertility (Lok et al., 2000; Brailoiu et al., 2005; Ivell and Grutzner. 2009; Chan et al., 2011). In this study, a missense variant of INSL6 (Supplementary Table S14; exon2:c.C403G: p.Q135E) was found among the Anhui pig breeds. This variation promotes a Gln-to-Glu substitution, implying that variations may impact the progression of spermatogenesis. Mitogen-activated protein kinase 12 (MAP3K12) is involved in the MAPK signaling pathway and is specifically selected in a high fecundity goat lineage, indicating its role in reproduction (Lai et al., 2016). MAPK1 plays an important role in embryonic and placental development (Hatano et al., 2003). Furthermore, a signal adequate for promoting trophoblast proliferation and invasion can decrease below thresholds if MAPK1 protein expression is insufficient (Saba et al., 2003; Jeong et al., 2013). In a previous study, population high-density SNP array analysis of multiparous and uniparous sheep revealed that MAPK1 is a selected gene among Kazakhstan sheep (Wang et al., 2020), highlighting its role in reproduction. One synonymous SNV was also identified in MAPK1 (Supplementary Table S15; exon1:c.C144T: p.L48L).

Genes related to lipid and meat traits have also been identified. Sorting nexin 19 (SNX19) is primarily associated with meat quality and plays a vital role in the fat subnetwork (Kogelman et al., 2014). Interestingly, SNX19 was identified as a selected gene in a previous pig study (Guo et al., 2021). Five nonsynonymous variants were identified for SNX19 (Supplementary Table S16; exon11: c. A1037G: p.N346S, exon7:c.C487T: p.P163S, exon4: c. G94T: p.V32L, exon2:c.C1834T: p.L612F, exon1: c. G37C: p.A13P), and the variants with observable phenotypes were used to discriminate the functional site. We also identified myostatin (MSTN), a transcription factor belonging to the transforming growth factor beta (TGFβ) superfamily which plays an important role in muscle and fat development in pigs. Gene ontology annotation showed that among the Anhui pig populations, MSTN was enriched for growth (GO: 0040007), growth factor activity (GO: 0008083), and protein binding (GO: 0003824). Furthermore, a study by Deng et al. (2012) revealed that MSTN impacts the development of the longissimus muscle and the rectus superior muscle. Transgenic pigs with MSTN displayed decreased muscle growth and significant increases in intramuscular fat (Yang et al., 2009). Additionally, it has been shown that MSTN has the potential to regulate adipogenesis of mesenchymal stem cells during the determination and differentiation phases (Sun et al., 2016). MSTN inhibits adipogenesis in preadipocytes, (Hirai et al., 2007; Carrarelli et al., 2015), whereas it promotes adipogenesis in pluripotent stem cells (Feldman et al., 2006; Pantoja et al., 2008). In animals, MSTN-knockout typically reduces fat mass and resistance to diet-induced obesity (Guo et al., 2009; Gu et al., 2016).

### 4.2 The selected genes of Anhui pig populations compared to those of the commercial pig population

We identified several genes related to reproduction among the GO terms and KEGG pathways, with each playing an important role in spermatogenesis, ovarian steroidogenesis, the estrogen signaling pathway, the FoxO signaling pathway, or oocyte meiosis. Of these genes, calcium-binding protein spermatid associated 1(CABS1) and mitogen-activated protein kinase 1 (MAPK1) were those found in the selective regions of the Anhui pig population, but not in the selective regions of the Asian wild boar. The insulin-like growth factor 1 receptor (IGF1R) is widely expressed in mammals (including pigs, sheep, goats, and ducks) and is involved in growth, carcass traits, and reproductive performance (Liu et al., 1993; Terman, 2011; Hoopes et al., 2012; Kijas et al., 2012; Zhao et al., 2018; Jin et al., 2020; Grochowska et al., 2021; Jiang et al., 2021). In female mice, IGF1R was found to play a vital role in steroidogenesis, follicle survival, and fertility (Baumgarten et al., 2017), where it helped transfer nutrients to the fetus (Hellström et al., 2016). Zhou et al. (2021) found that IGF1R is also necessary for epithelial differentiation and normal uterine preparation for embryo implantation (Zhou et al., 2021). Given this background, IGF1R likely contributes to phenotypic differences in growth and reproduction among pigs. In this study, 450 variations were identified in the IGF1R gene (Supplementary Table S18), and only seven were located in exons, none of which resulted in amino acid changes. Conversely, three variations were found within the 3′UTR, and 30 within the 5′UTR that may regulate the function of the IGF1R. Insulin receptor (INSR), a tyrosine kinase receptor, affects development and growth (Hubbard. 2013; Bedinger and Adams. 2015). INSR has been validated as a polycystic ovary syndrome risk locus using a very large, well-designed case-control GWAS (Shi et al., 2012). Meanwhile, INSR has been confirmed to be necessary for optimal endometrial proliferation and implantation (Sekulovski et al., 2021). Furthermore, INSR plays a crucial role in controlling adipose tissue development and adipocyte survival (Boucher et al., 2012; Cignarelli et al., 2019). INSR may be responsible for fat accumulation in adipose tissues, which could induce angiopoietin-like 8, inhibit lipolysis, control postprandial fat storage in white adipose tissue, and direct that fatty acids be stored in the adipose tissue during the fed state (Ahbara et al., 2019). The testis-specific isoform of LIM kinase 2 (tLIMK2) plays a key role in the progression of spermatogenesis (Takahashi et al., 2002). The weight of LIMK2−/− mice sharply decreased by 20% compared to that of control mice. Furthermore, inhibition of LIMK1/2 activity results in failure of embryo cleavage and blastocyst formation (Duan et al., 2018). POZ/BTB and AT hook containing zinc finger 1(PATZ1), a zinc finger protein, can affect the basal activity of different promoters. PATZ1 plays a crucial role in embryonic development (Valentino et al., 2013) and normal male gametogenesis and impairment of PATZ1 result in disruption of testis cytoarchitecture and block spermatogenesis (Fedele et al., 2008).

According to previous research, indigenous Anhui pig breeds exhibit significant differences in meat quality traits and fat deposition capacity compared to commercial pig breeds (Xu et al., 2018; Hu et al., 2019; Li et al., 2021). In the present study, several genes were found to be associated with fat deposition and other carcass quality traits. The melanocortin five receptor gene (MC5R), a member of the G protein-coupled receptor superfamily, participates in lipid production, fatty acid oxidation of skeletal muscle, and lipolysis of dipocytes. MC5R is associated with the metabolism of skeletal muscle, fatty acids, and fat cells, as well as dysfunction of the exocrine gland under abnormal expression (Zhang et al., 2011). The polymorphisms of MC5R are associated with obesity in humans (Chagnon et al., 1997; Valli et al., 2008). Furthermore, MC5R deficiency accelerates lipolysis and reduces oil secretion (Chen et al., 1997). Whole genome analysis of 46 cattle from six representative Chinese breeds together with international breeds has shown that MC5R was under-selected (Mei et al., 2018). In the pig genome, MC5R was found close to the 97.625–98.725 Mb regions on swine chromosome 6 (SSC6). The regions of SSC6 are associated with lipid metabolism, back fat thickness, and intramuscular fat percentage (Switonski et al., 2013; Ma et al., 2018), and play a vital role in proinflammatory activity (Jun et al., 2010). Among the pigs in this study, three variations were found in the 3′UTR of the MC5R gene, and four variations were located in the exon (Supplementary Table S19). Two exonic variants (exon3:c.A952G:p. S318G; exon3:c.A580G:p.T194A) resulted in amino acid changes from Ser to Gly and Thr to Ala. The exon1: c. G92A created a premature stop codon in the MC5R amino acid sequence. Additionally, kinase cGMP-dependent 1 (PRKG1) has been shown to regulate adipocytes lipolysis and plays an important role in pig fatty acid composition (Revilla et al., 2017). Interestingly, PRKG1 exhibits differential expression between the high and low fatty acid composition groups in muscle according to RNA-Seq analysis (Puig et al., 2014). Moreover, PRKG1 knockout mice have decreased triglyceride stores in brown adipose tissue (Amieux and Mcknight, 2010). The taste and quality of cooked and cured meat are directly affected by the oxidative stability of the muscle, which is related to fatty acid (FA) composition (Serra et al., 1998; Wood et al., 2008). We also found two genes, CREB binding protein (CREBBP) and adenylate cyclase 9 (ADCY9), which are involved in cAMP signaling. cAMP signaling regulates energy homeostasis in multiple tissues by mediating the action of metabolism-controlling hormones such as glucagon and epinephrine (Ravnskjaer et al., 2016). A previous GWAS and post-GWAS study discovered that CREBBP and ADCY9 are located inside regions that are significantly associated with meat pH (Verardo et al., 2017). For ADCY9, there were 13 exonic variants, three of which were missense variants (Supplementary Table S20, exon8:c.A2594G:p.H865R; exon8:c.T2669C:p.M890T; exon11:c.G3943T:p.G1315C), results in amino acid changes that may impact meat quality. CREBBP regulates a plethora of metabolic target genes involved in glucose metabolism (Ravnskjaer et al., 2016), which can also potentially affect meat quality due to acidification. In this study, one missense variant was identified (Supplementary Table S21, exon22:c.A3907G:p. I1303V) in which the amino acid Ile was changed to Val.

Two selected genes were found to be associated with ear size phenotypic traits. The size and type of the ears are important conformational characteristics that distinguish pig breeds. Many indigenous Chinese pig breeds, such as the six Anhui pig populations, have unusually large floppy ears. In contrast, commercial breeds (Duroc, Landrace, Yorkshire, and Pitelan) have smaller, more erect ears. In this study, we identified two important candidate genes influenced by selection. The first is methionine sulfoxide reductase B3 (MSRB3), which has been shown to be associated with ear size in sheep, goats, dogs, and pigs (Webster et al., 2015; Zhang et al., 2015; Chen et al., 2018; Kumar et al., 2018; Zhao et al., 2020; Posbergh and Huson. 2021). In the present study, a variant in the 3′-UTR of MSRB3 was identified among the Anhui pig breeds (Supplementary Table S17; g. T29862412C), suggesting that it could be the source of their erect ear phenotype. SRY-box transcription factor 5 (SOX5) encodes a member of the SOX transcription factor family, and has been shown to play a role in chondrogenesis (Lefebvre et al., 1998; Smits et al., 2001). Additionally, it has been identified as a selected gene in Duroc pigs and Duroc × Korean native pigs, in which it plays a key role in ear morphology (Edea et al., 2017). Moreover, mutations in genes encoding homeobox transcription factors are often responsible for the defective development of the outer ear (Fekete, 1999). Positive selection of MSRB3 and SOX5 among the Anhui pig populations, but not among the commercial pig breeds, may be the genetic mechanism behind the ear size phenotype.

Although the present study has produced many interesting findings, it has its limitations. Firstly, as the phenotypic values were not collected, functional experimental assays are still needed to further validate the associations between the phenotypes and genotypes of the mentioned variants and to identify the targets involved in reproduction, lipid and meat quality, and ear size.

In this study, we generated a novel catalog of population genomic data on the Anhui pig populations using whole-genome resequencing. Population genomic analyses have further elucidated genomic variation, population structure, demographic history, and signatures of selection among Anhui pigs. Moreover, we discovered several potential signatures of selection associated with the domestication characteristics of the Anhui pig populations, with selected regions involved in reproduction, lipid and meat characteristics, and ear size. These findings substantially expand the catalogue of genetic variants among pigs, and the newly generated genome-wide data are a valuable resource for future genetic studies and those to improve genome-assisted breeding of pigs and other domestic animals.

## Data Availability

The datasets presented in this study can be found in online repositories. The names of the repository/repositories and accession number(s) can be found in the article/Supplementary Material.
