# Supplementary material for: Genetic architecture and selection of Anhui autochthonous pig population revealed by whole genome resequencing
Source: Front Genet. 2022 Oct 17;13:1022261. doi: 10.3389/fgene.2022.1022261 (PMC9618877; doi:10.3389/fgene.2022.1022261)
Supplement: Supplementary file 1 [file DataSheet1.DOCX]

Supplementary Material

# Supplementary Data

**Additional file 1: Note S1.** Characteristics of the Anhui pig breeds.

The **ASP** originated in Anqing, China. It is named after its black coat, but its forehead, four hooves, and tail are white. Its core production areas are located in Wangjiang County and Taihu County, and a small number of them are also distributed in Huaining and Susong County [1]. The body is medium to small, with large drooping ears, and obvious wrinkles on the forehead. It has the characteristics of strong disease resistance, resistance to crude-feed, and excellent meat quality. The animals studied were sampled in the Taihu county of Anqing, the geographic center of origin of the breed.

The **WBP** is an excellent indigenous pig breed in Anhui Province and a national-level protected pig breed, which has a long breeding history and is a precious local pig germplasm resource in my country [1]. The WBP are mainly distribute in the mountainous area of southern Anhui Province, with Jixi, Ningguo, Shexian, Jingde and other places as the central production areas. They are medium in size. Coat color is black. The ear is medium to large in size. The abdomen is large. The WBP have high farrowing rate, resistance to crude-feed, strong disease and stress resistance, and a gentle temperament. We sequenced WBP pig from Jixi County.

The **WP** is named after it is mainly produced in the polder and some hilly areas of more than ten counties and cities in Anhui Province [1]. It is a unique excellent pig breed in Anhui Province. It has been included in the key protection list of local breeds in China. The central production area of WP is in Nanling County and Xuancheng District along the polder area on both sides of the Yijiang River. WP are medium to small in size and well-proportioned in structure, with large ears, thicker skin and larger skeletons. WP have the characteristics of high fecundity, strong feeding ability, strong resistance to disease and stress, rich intramuscular fat, and resistance to rough feeding. We sequenced WP pig from Nanling county.

The **DYP** (**HBP**), a group of Huai pig populations, are one of the main pig breeds in the Jianghuai area. The central production area of DYP (HBP) is in Dingyuan County and Chuzhou City [1]. It is mainly distributed in Hefei, Huoshan and Shouxian, Lu'an (Huoshou black pig is a branch of Dingyuan pig, which gradually differentiated due to regional reasons), Huainan, Bengbu and other areas north of the Yangtze River are also distributed. The DYP (HBP) have black hair, large body and small head, slender and uniform face, medium-large drooping ears, shallow facial folds, mainly distributed longitudinally, without groove back. The DYP (HBP) has characteristics of strong adaptability, high fecundity, resistance to rough feeding, high slaughter rate, good meat quality, and more suet oil. The animals studied were sampled in the Dingyuan county and Huoqiu county, the geographic center of origin of the population.

The **WSP** is a special local excellent meat and fat breed that is unique to Anhui Province [1]. It is native to Kecun area, Yi County, Huangshan City, southern Anhui Province. It is also distributed in neighboring places such as Jingdezhen and Chizhou. The physique of WSP is medium, thin and loose. The coat is black and white, the skin is pink, and the lower extremities are white. The WSP has an earlier matures, high fecundity, good meat quality, and resistance to rough feeding. The animals studied were sampled in the Yi County, the geographic center of origin of the breed.

REFERENCE

1. China National Commission of Animal Genetic Resource. Animal Genetic Resource in China. Pig [M]. China Agriculture Press, 2011.

# Supplementary Figures and Tables

## Supplementary Figures

**
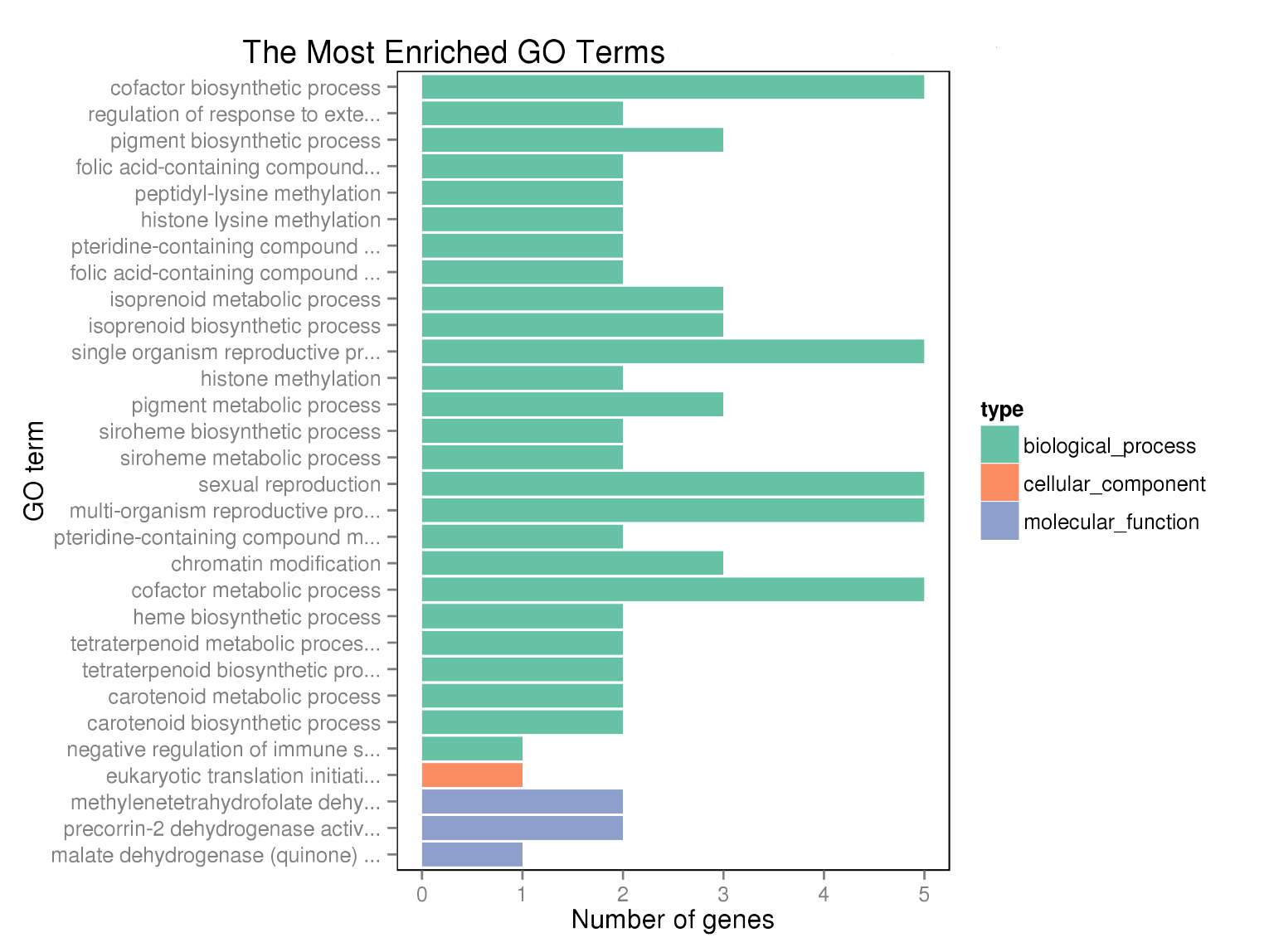
Figure S1.** Go analysis of 188 genes.

**Figure S2.** KEGG analysis of 188 gene
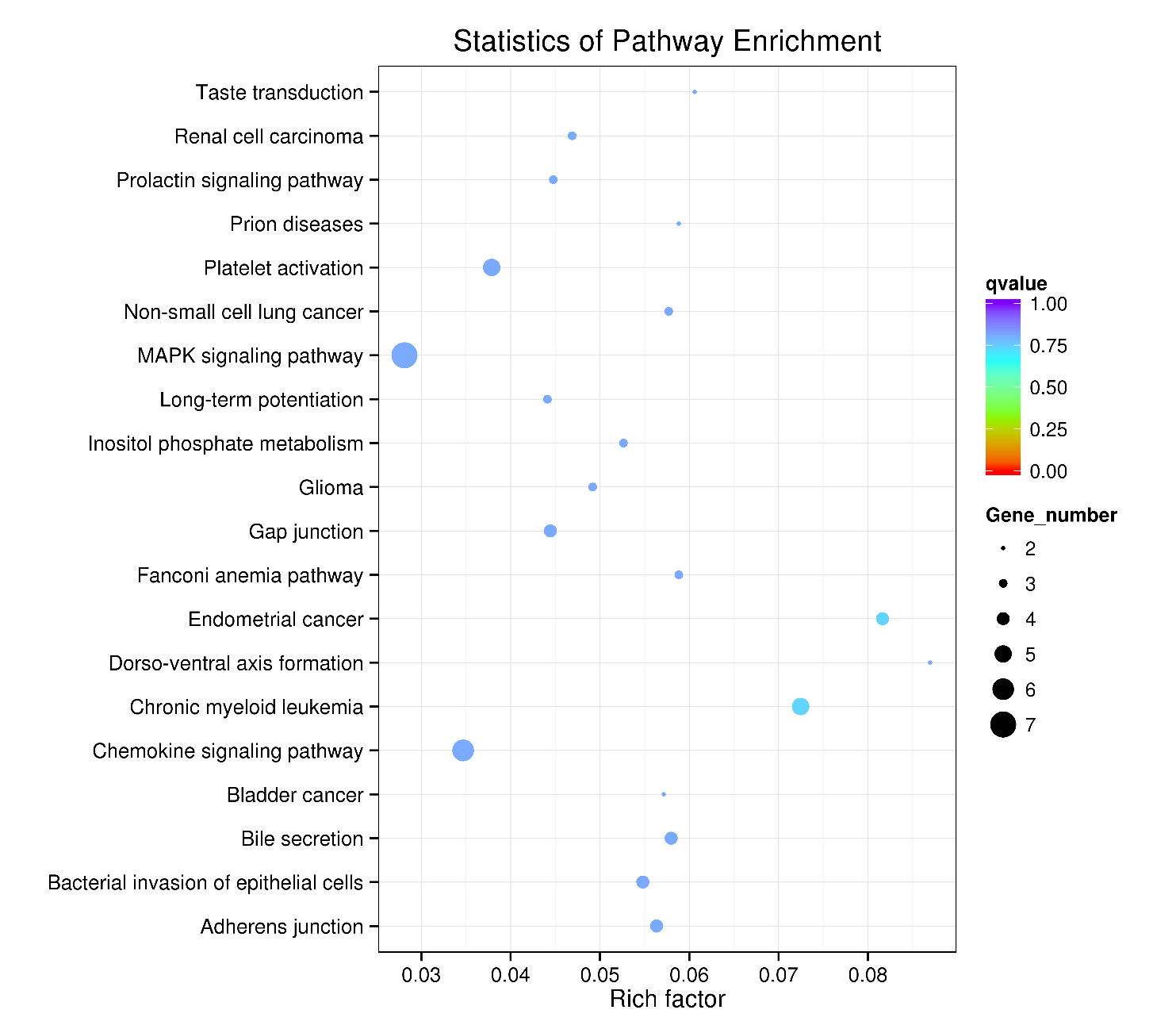
.


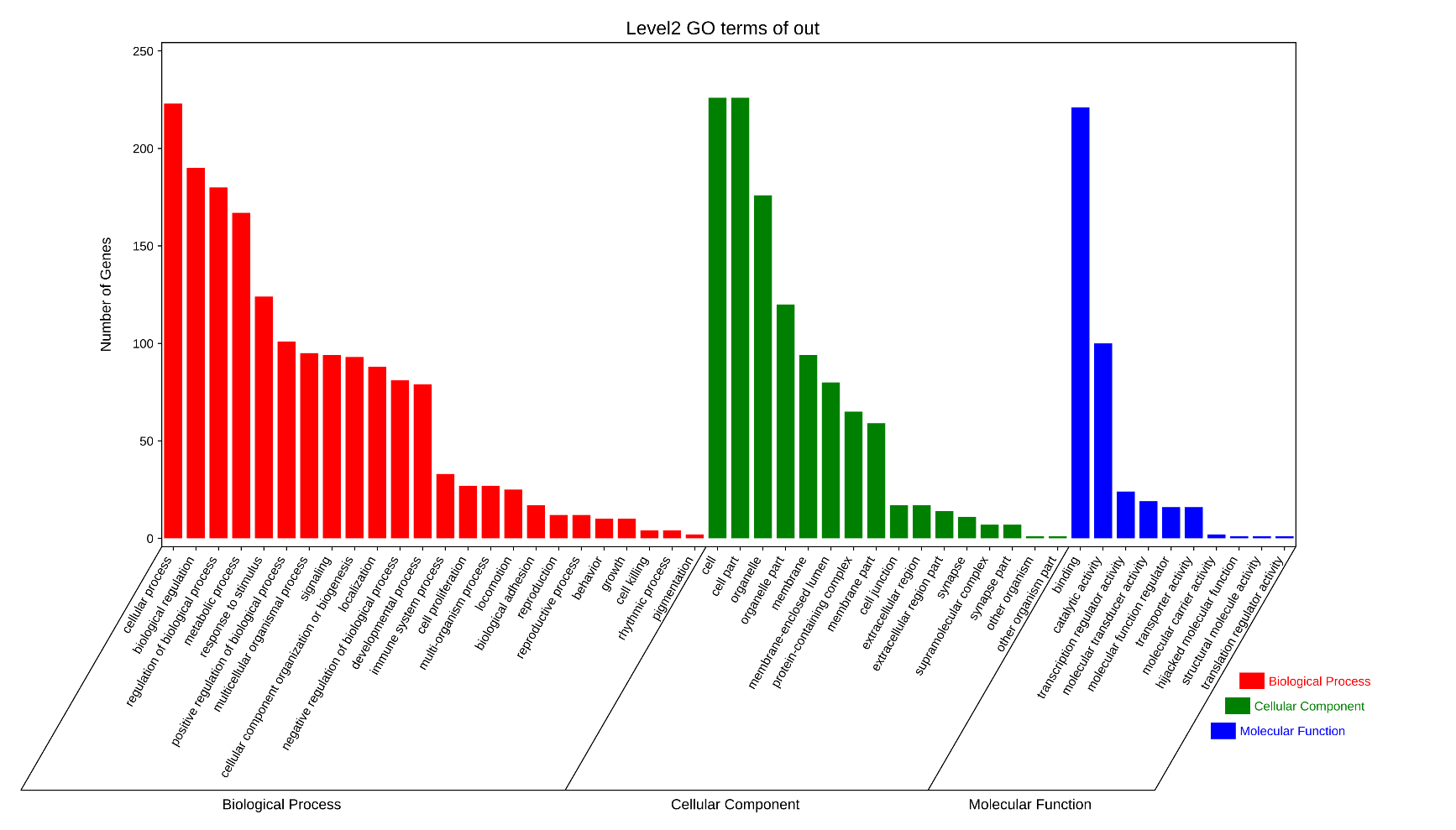


**Figure S3.** Go analysis of 331 genes.


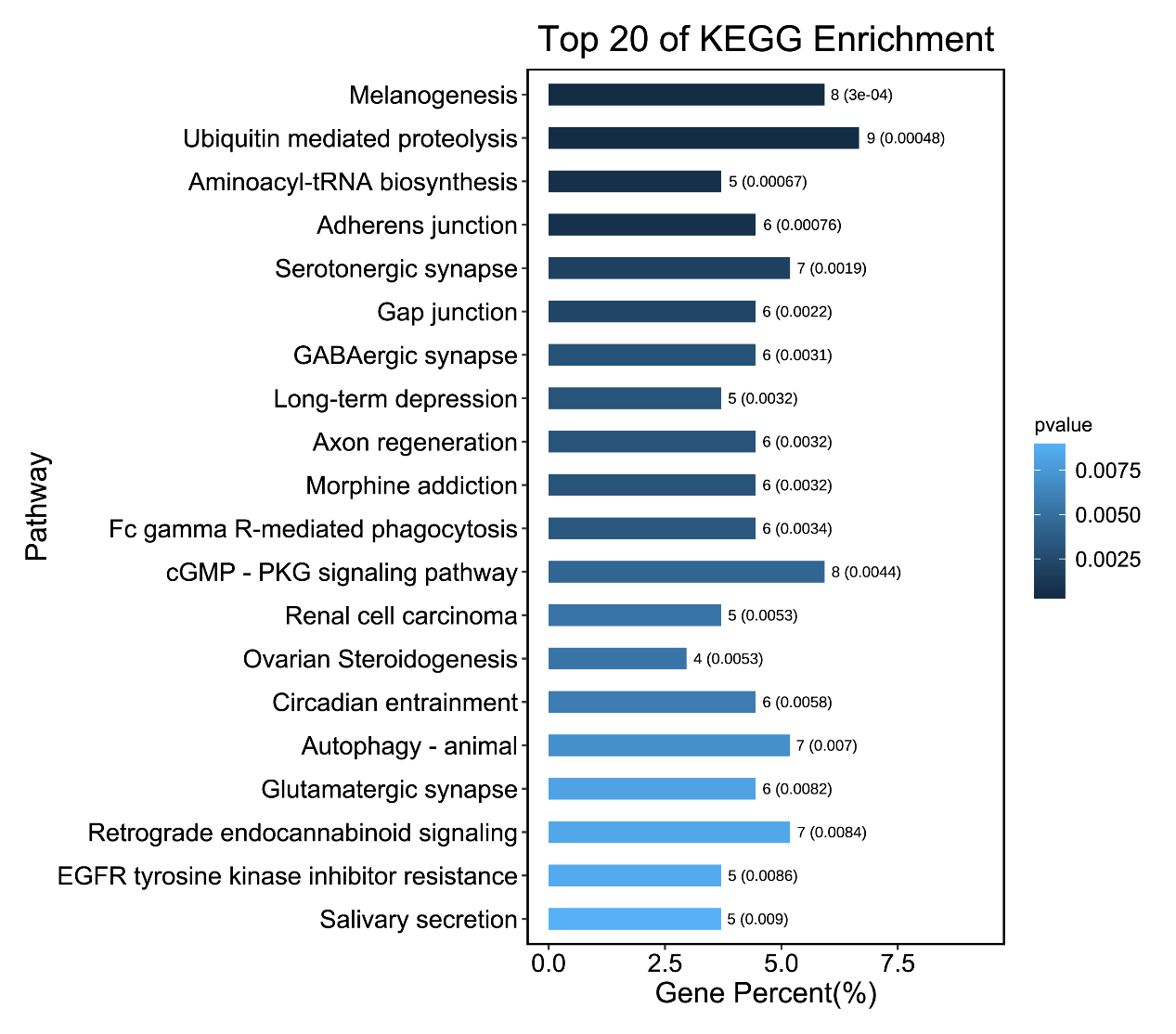
**Figure S4.** KEGG analysis of 331 genes.

## Supplementary Tables

| **Table S1.** The geographic origins and abbreviations of 150 pigs | | | | | |
| --- | --- | --- | --- | --- | --- |
| Breeds | Abb. | Samples | Origin | Longitude | Latitude |
| Wannan Spotted pig | WSP | 25 | Yi county | 117°56'E | 29°93'N |
| Wannan Black pig | WBP | 25 | Jixi county | 118°57'E | 30°07'N |
| Anqing six-end-white pig | ASP | 25 | Anqing | 116°33'E | 30°19'N |
| Wei pig | WP | 25 | Nanling county | 118°20'E | 30°56'N |
| Dingyuan pig | DYP | 25 | Dingyuan county | 117°68'E | 32°52'N |
| Huoshou Black pig | HBP | 25 | Huoqiu county | 116°27'E | 32°35'N |

**Table S2.** The detail information of downloaded sequencing data

| Breed | Accession No. | High-quality data (Gb) | References |
| --- | --- | --- | --- |
| Asian wild boar | SRS387324 | 16.28 | Li et al., 2013 |
|  | SRS387323 | 16.30 | Li et al., 2013 |
|  | SRS387320 | 12.00 | Li et al., 2013 |
|  | SRS465721 | 56.67 | Ai et al., 2015 |
|  | SRS465720 | 54.76 | Ai et al., 2015 |
|  | SRS465719 | 55.92 | Ai et al., 2015 |
|  | SRS465718 | 71.18 | Ai et al., 2015 |
|  | SRS465717 | 72.68 | Ai et al., 2015 |
|  | SRS465716 | 66.13 | Ai et al., 2015 |
|  | ERR173222 | 19.29 | Groenen et al., 2012 |
|  | ERR173221 | 9.31 | Groenen et al., 2012 |
|  | ERR173220 | 19.83 | Groenen et al., 2012 |
|  | ERR173219 | 9.83 | Groenen et al., 2012 |
| Landrace | ERR173186 | 19.98 | Groenen et al., 2012 |
|  | ERR173187 | 19.98 | Groenen et al., 2012 |
|  | ERR173188 | 19.96 | Groenen et al., 2012 |
| Duroc | SAMN06554454 | 14.68 | Zhao et al., 2018 |
|  | SAMN06554538 | 12.08 | Zhao et al., 2018 |
|  | SAMN06560339 | 11.12 | Zhao et al., 2018 |
|  | SAMN06562573 | 35.06 | Zhao et al., 2018 |
|  | SAMN06562586 | 10.35 | Zhao et al., 2018 |
|  | SAMN06562588 | 12.71 | Zhao et al., 2018 |
|  | SAMN06562603 | 13.61 | Zhao et al., 2018 |
|  | SAMN06562961 | 32.77 | Zhao et al., 2018 |
|  | SAMN06562976 | 16.13 | Zhao et al., 2018 |
|  | SAMN06562981 | 32.45 | Zhao et al., 2018 |
|  | SAMN06562994 | 30.10 | Zhao et al., 2018 |
|  | SAMN06563568 | 21.22 | Zhao et al., 2018 |
|  | SAMN06578935 | 27.30 | Zhao et al., 2018 |
|  | SAMN06606756 | 20.23 | Zhao et al., 2018 |
|  | SAMN06611050 | 17.99 | Zhao et al., 2018 |
|  | SAMN06611257 | 26.71 | Zhao et al., 2018 |
|  | SAMN06617789 | 28.02 | Zhao et al., 2018 |
|  | SAMN06618362 | 28.59 | Zhao et al., 2018 |
|  | SAMN09111834 | 25.78 | Zhao et al., 2018 |
| Pietrain | ERR875322 | 30.05 | Groenen et al., 2012 |
|  | ERR875326 | 30.18 | Groenen et al., 2012 |
| Yorkshire | ERR173180 | 18.34 | Groenen et al., 2012 |
|  | ERR173181 | 27.01 | Groenen et al., 2012 |
|  | ERR173182 | 17.56 | Groenen et al., 2012 |

**Table S3.** Summary statistic of resequencing data

| Sample | sex | Raw Base(bp) | Clean Base(bp) | Effective Rate(%) | Error Rate(%) | Q20(%) | Q30(%) | GC Content(%) |
| --- | --- | --- | --- | --- | --- | --- | --- | --- |
| HBP1 | Female | 35,700,207,000 | 35,642,108,100 | 99.84 | 0.03 | 96.72 | 91.86 | 43.56 |
| HBP2 | Male | 30,899,187,600 | 30,851,546,400 | 99.85 | 0.03 | 95.9 | 90.01 | 42.8 |
| HBP3 | Female | 30,143,800,200 | 30,100,876,200 | 99.86 | 0.03 | 96.71 | 91.87 | 42.67 |
| HBP4 | Male | 35,486,926,800 | 35,434,778,400 | 99.85 | 0.03 | 96.56 | 91.55 | 43.2 |
| HBP5 | Male | 31,616,021,400 | 31,562,326,500 | 99.83 | 0.03 | 96.85 | 92.22 | 43.42 |
| HBP6 | Female | 32,922,356,400 | 32,873,345,400 | 99.85 | 0.03 | 96.25 | 90.66 | 43.17 |
| HBP7 | Male | 31,754,851,800 | 31,720,179,000 | 99.89 | 0.03 | 96.88 | 92.25 | 43.28 |
| HBP8 | Male | 34,508,241,300 | 34,456,751,700 | 99.85 | 0.03 | 96.88 | 92.26 | 43.34 |
| HBP9 | Male | 28,475,372,100 | 28,444,845,900 | 99.89 | 0.04 | 95.31 | 88.7 | 43.48 |
| HBP10 | Female | 27,932,767,500 | 27,873,116,700 | 99.79 | 0.04 | 94.48 | 87.44 | 42.62 |
| HBP11 | Male | 30,177,642,600 | 30,145,067,700 | 99.89 | 0.03 | 96.96 | 92.41 | 43.38 |
| HBP12 | Male | 44,041,665,900 | 43,975,527,600 | 99.85 | 0.03 | 95.75 | 89.61 | 43.17 |
| HBP13 | Female | 29,652,163,200 | 29,626,143,600 | 99.91 | 0.03 | 96.17 | 90.42 | 43.26 |
| HBP14 | Female | 28,998,863,100 | 28,970,510,700 | 99.9 | 0.03 | 96.38 | 90.91 | 43.13 |
| HBP15 | Female | 29,622,998,700 | 29,563,206,900 | 99.8 | 0.03 | 96.97 | 92.44 | 42.95 |
| HBP16 | Female | 28,176,975,600 | 28,141,002,300 | 99.87 | 0.03 | 96.97 | 92.42 | 43.45 |
| HBP17 | Male | 27,469,770,600 | 27,428,281,800 | 99.85 | 0.03 | 96.78 | 92.02 | 43.39 |
| HBP18 | Female | 33,544,454,700 | 33,498,084,000 | 99.86 | 0.03 | 96.8 | 91.7 | 43.23 |
| HBP19 | Female | 30,032,295,600 | 29,979,278,400 | 99.82 | 0.04 | 94.6 | 87.47 | 42.61 |
| HBP20 | Female | 30,535,414,200 | 30,504,176,400 | 99.9 | 0.03 | 96.38 | 90.96 | 43.47 |
| HBP21 | Female | 38,896,537,500 | 38,850,612,900 | 99.88 | 0.03 | 96.47 | 91.25 | 42.72 |
| HBP22 | Female | 30,832,860,300 | 30,799,440,600 | 99.89 | 0.03 | 96.86 | 92.17 | 43.34 |
| HBP23 | Female | 38,055,698,100 | 38,015,393,400 | 99.89 | 0.04 | 95.46 | 89 | 43.27 |
| HBP24 | Female | 25,965,282,000 | 25,929,402,000 | 99.86 | 0.03 | 96.27 | 90.69 | 43.42 |
| HBP25 | Male | 36,004,575,900 | 35,956,438,500 | 99.87 | 0.03 | 96.85 | 92.12 | 43 |
| ASP1 | Female | 31,454,498,700 | 31,426,692,900 | 99.91 | 0.06 | 93.9 | 85 | 42.36 |
| ASP2 | Male | 28,423,930,500 | 28,389,414,000 | 99.88 | 0.04 | 94.45 | 86.72 | 42.34 |
| ASP3 | Female | 28,865,928,000 | 28,840,587,600 | 99.91 | 0.04 | 93.72 | 85.01 | 42.25 |
| ASP4 | Female | 28,109,637,900 | 28,082,657,700 | 99.9 | 0.06 | 93.6 | 85 | 43.23 |
| ASP5 | Female | 27,349,062,600 | 27,322,325,100 | 99.9 | 0.04 | 94.03 | 85.76 | 43.06 |
| ASP6 | Female | 27,990,069,000 | 27,963,496,800 | 99.91 | 0.04 | 94.31 | 86.42 | 43.08 |
| ASP7 | Male | 26,712,895,800 | 26,693,237,100 | 99.93 | 0.04 | 94.16 | 85.94 | 42.41 |
| ASP8 | Female | 27,198,526,500 | 27,165,855,600 | 99.88 | 0.06 | 93.69 | 85 | 42.34 |
| ASP9 | Male | 25,980,980,100 | 25,963,353,300 | 99.93 | 0.04 | 93.66 | 85 | 42.53 |
| ASP10 | Female | 27,380,404,800 | 27,352,414,800 | 99.9 | 0.04 | 94.11 | 86.02 | 42.36 |
| ASP11 | Female | 25,898,778,900 | 25,840,782,000 | 99.78 | 0.06 | 93.72 | 85 | 42.31 |
| ASP12 | Female | 26,831,145,900 | 26,812,146,900 | 99.93 | 0.04 | 93.84 | 85.24 | 42.41 |
| ASP13 | Female | 32,161,918,800 | 32,115,021,900 | 99.85 | 0.03 | 96.12 | 90.38 | 42.41 |
| ASP14 | Male | 25,955,334,600 | 25,919,311,800 | 99.86 | 0.03 | 96.19 | 90.47 | 42.2 |
| ASP15 | Male | 34,603,158,600 | 34,558,435,200 | 99.87 | 0.03 | 96.21 | 90.58 | 42.38 |
| ASP16 | Male | 30,531,011,700 | 30,482,231,400 | 99.84 | 0.03 | 96.05 | 90.29 | 43.07 |
| ASP17 | Male | 29,296,422,900 | 29,253,483,900 | 99.85 | 0.03 | 96.13 | 90.45 | 42.95 |
| ASP18 | Male | 29,639,970,900 | 29,594,805,300 | 99.85 | 0.03 | 96.2 | 90.6 | 43.14 |
| ASP19 | Male | 29,112,006,000 | 29,074,831,500 | 99.87 | 0.03 | 96.34 | 90.86 | 42.37 |
| ASP20 | Male | 39,669,701,100 | 39,605,599,500 | 99.84 | 0.03 | 96.68 | 91.53 | 43.05 |
| ASP21 | Male | 34,453,380,900 | 34,401,426,900 | 99.85 | 0.03 | 96.5 | 91.21 | 43.23 |
| ASP22 | Male | 34,279,285,800 | 34,224,028,500 | 99.84 | 0.03 | 96.62 | 91.44 | 43.13 |
| ASP23 | Male | 34,631,467,500 | 34,586,558,100 | 99.87 | 0.03 | 96.68 | 91.55 | 42.89 |
| ASP24 | Male | 28,795,039,800 | 28,753,998,600 | 99.86 | 0.03 | 96.76 | 91.75 | 43.17 |
| ASP25 | Male | 35,788,818,300 | 35,738,116,200 | 99.86 | 0.03 | 96.79 | 91.78 | 43.05 |
| WSP1 | Female | 26,202,078,600 | 26,171,092,200 | 99.88 | 0.03 | 96.31 | 90.75 | 42.31 |
| WSP2 | Female | 30,562,651,500 | 30,522,599,400 | 99.87 | 0.03 | 96.27 | 90.69 | 42.34 |
| WSP3 | Male | 32,699,005,500 | 32,642,172,900 | 99.83 | 0.03 | 96.29 | 90.75 | 43.83 |
| WSP4 | Male | 30,460,097,700 | 30,413,282,400 | 99.85 | 0.03 | 96.68 | 91.62 | 43.66 |
| WSP5 | Male | 26,871,635,700 | 26,827,940,400 | 99.84 | 0.03 | 96.89 | 92.04 | 43.93 |
| WSP6 | Male | 28,691,794,200 | 28,654,569,000 | 99.87 | 0.03 | 96.6 | 91.4 | 42.95 |
| WSP7 | Female | 31,978,301,100 | 31,942,902,900 | 99.89 | 0.03 | 96.69 | 91.52 | 43.08 |
| WSP8 | Male | 26,528,506,800 | 26,490,820,500 | 99.86 | 0.03 | 96.5 | 91.15 | 42.98 |
| WSP9 | Female | 30,898,908,900 | 30,853,429,200 | 99.85 | 0.03 | 96.69 | 91.56 | 42.87 |
| WSP10 | Female | 37,211,355,900 | 37,167,745,200 | 99.88 | 0.03 | 96.67 | 91.53 | 42.91 |
| WSP11 | Female | 29,269,823,700 | 29,237,273,700 | 99.89 | 0.03 | 96.5 | 91.15 | 43.07 |
| WSP12 | Female | 31,377,994,500 | 31,344,967,200 | 99.89 | 0.03 | 96.34 | 90.77 | 42.88 |
| WSP13 | Female | 27,773,017,200 | 27,730,928,400 | 99.85 | 0.03 | 96.59 | 91.34 | 43.11 |
| WSP14 | Male | 27,138,414,000 | 27,105,798,300 | 99.88 | 0.03 | 95.65 | 89.42 | 42.25 |
| WSP15 | Male | 28,254,399,600 | 28,217,563,200 | 99.87 | 0.03 | 96.12 | 90.44 | 42.89 |
| WSP16 | Male | 27,370,127,100 | 27,338,081,400 | 99.88 | 0.03 | 96.22 | 90.59 | 42.73 |
| WSP17 | Female | 26,527,642,800 | 26,499,286,500 | 99.89 | 0.03 | 96.16 | 90.46 | 42.75 |
| WSP18 | Female | 28,001,004,300 | 27,973,028,700 | 99.9 | 0.03 | 95.77 | 89.71 | 42.28 |
| WSP19 | Male | 27,409,871,400 | 27,376,987,500 | 99.88 | 0.03 | 96.06 | 90.21 | 42.19 |
| WSP20 | Male | 26,991,239,100 | 26,961,056,400 | 99.89 | 0.03 | 95.89 | 89.93 | 42.39 |
| WSP21 | Female | 26,900,411,700 | 26,870,408,100 | 99.89 | 0.03 | 96.04 | 90.21 | 42.28 |
| WSP22 | Female | 29,131,166,400 | 29,080,647,600 | 99.83 | 0.03 | 97.31 | 93.05 | 42.59 |
| WSP23 | Female | 27,241,891,200 | 27,216,783,900 | 99.91 | 0.03 | 96.78 | 91.75 | 42.95 |
| WSP24 | Female | 33,904,560,900 | 33,862,763,100 | 99.88 | 0.03 | 96.72 | 91.6 | 43.03 |
| WSP25 | Female | 26,359,048,200 | 26,326,405,800 | 99.88 | 0.03 | 96.57 | 91.28 | 43.01 |
| DYP1 | Female | 32,429,200,800 | 32,385,122,700 | 99.86 | 0.03 | 96.57 | 91.31 | 42.98 |
| DYP2 | Female | 29,566,030,200 | 29,523,533,700 | 99.86 | 0.03 | 96.52 | 91.24 | 43.76 |
| DYP3 | Male | 32,260,852,800 | 32,214,062,700 | 99.85 | 0.03 | 96.78 | 91.81 | 43.66 |
| DYP4 | Female | 26,693,702,400 | 26,664,693,600 | 99.89 | 0.03 | 96.84 | 91.91 | 43.55 |
| DYP5 | Male | 33,906,552,900 | 33,862,324,200 | 99.87 | 0.03 | 96.37 | 90.88 | 43 |
| DYP6 | Male | 32,392,292,400 | 32,348,713,500 | 99.87 | 0.03 | 96.56 | 91.26 | 42.88 |
| DYP7 | Female | 29,164,531,200 | 29,129,482,500 | 99.88 | 0.03 | 96.62 | 91.44 | 43.05 |
| DYP8 | Female | 32,662,288,800 | 32,627,034,600 | 99.89 | 0.03 | 96.39 | 90.92 | 42.87 |
| DYP9 | Female | 28,683,631,500 | 28,652,237,400 | 99.89 | 0.03 | 96.64 | 91.3 | 42.72 |
| DYP10 | Female | 29,663,970,900 | 29,624,373,300 | 99.87 | 0.03 | 96.81 | 91.83 | 43.07 |
| DYP11 | Female | 30,221,277,300 | 30,182,826,000 | 99.87 | 0.03 | 96.29 | 90.72 | 43.08 |
| DYP12 | Female | 26,485,821,000 | 26,453,593,800 | 99.88 | 0.03 | 96.34 | 90.77 | 43.11 |
| DYP13 | Female | 36,646,133,700 | 36,583,256,700 | 99.83 | 0.03 | 96.64 | 91.48 | 42.91 |
| DYP14 | Female | 31,492,723,500 | 31,444,932,600 | 99.85 | 0.03 | 96.72 | 91.66 | 43.75 |
| DYP15 | Female | 25,978,230,600 | 25,946,352,300 | 99.88 | 0.03 | 97.44 | 93.38 | 43.17 |
| DYP16 | Female | 29,720,075,700 | 29,674,286,700 | 99.85 | 0.03 | 96.73 | 91.68 | 43.51 |
| DYP17 | Female | 30,691,910,400 | 30,661,262,400 | 99.9 | 0.03 | 96.74 | 91.67 | 42.9 |
| DYP18 | Female | 36,027,654,600 | 35,964,039,600 | 99.82 | 0.03 | 96.38 | 90.91 | 43.11 |
| DYP19 | Female | 29,919,256,800 | 29,873,322,600 | 99.85 | 0.03 | 96.84 | 91.92 | 43.11 |
| DYP20 | Male | 41,313,184,800 | 41,263,972,500 | 99.88 | 0.03 | 96.46 | 91.06 | 43.06 |
| DYP21 | Female | 30,835,918,500 | 30,802,208,700 | 99.89 | 0.03 | 96.41 | 90.95 | 42.95 |
| DYP22 | Female | 36,144,948,600 | 36,085,895,100 | 99.84 | 0.03 | 96.29 | 90.72 | 42.91 |
| DYP23 | Female | 37,703,317,500 | 37,650,927,600 | 99.86 | 0.03 | 96.46 | 91.04 | 43.05 |
| DYP24 | Female | 32,907,562,800 | 32,848,075,800 | 99.82 | 0.03 | 96.44 | 91.05 | 43.28 |
| DYP25 | Female | 34,753,756,500 | 34,712,381,700 | 99.88 | 0.03 | 96.56 | 91.27 | 42.89 |
| WBP1 | Female | 35,504,699,100 | 35,463,322,800 | 99.88 | 0.03 | 96.62 | 91.45 | 43.73 |
| WBP2 | Male | 39,667,780,200 | 39,625,240,800 | 99.89 | 0.03 | 96.49 | 91.16 | 43.75 |
| WBP3 | Male | 34,686,723,300 | 34,632,287,100 | 99.84 | 0.03 | 96.61 | 91.41 | 43.62 |
| WBP4 | Female | 39,198,335,700 | 39,158,093,400 | 99.9 | 0.03 | 96.62 | 91.42 | 42.78 |
| WBP5 | Female | 33,144,978,000 | 33,098,723,700 | 99.86 | 0.03 | 96.43 | 90.97 | 43 |
| WBP6 | Male | 34,531,073,100 | 34,498,226,700 | 99.9 | 0.03 | 96.47 | 91.06 | 43.03 |
| WBP7 | Female | 32,215,461,600 | 32,165,172,000 | 99.84 | 0.03 | 96.49 | 91.14 | 42.96 |
| WBP8 | Male | 29,315,667,000 | 29,274,508,200 | 99.86 | 0.03 | 96.79 | 91.81 | 43 |
| WBP9 | Female | 31,285,157,100 | 31,255,437,600 | 99.91 | 0.04 | 95.56 | 89.16 | 43.02 |
| WBP10 | Male | 35,009,279,100 | 34,958,995,200 | 99.86 | 0.03 | 96.65 | 91.46 | 43.11 |
| WBP11 | Female | 28,507,110,300 | 28,475,463,000 | 99.89 | 0.03 | 96.27 | 90.63 | 43.12 |
| WBP12 | Male | 39,147,016,200 | 39,097,456,500 | 99.87 | 0.03 | 96.57 | 91.34 | 42.97 |
| WBP13 | Male | 31,868,365,500 | 31,816,794,000 | 99.84 | 0.03 | 96.65 | 91.52 | 43.75 |
| WBP14 | Male | 31,188,969,600 | 31,137,370,500 | 99.83 | 0.03 | 96.66 | 91.56 | 43.76 |
| WBP15 | Female | 32,807,751,000 | 32,755,990,800 | 99.84 | 0.03 | 96.78 | 91.81 | 44.11 |
| WBP16 | Male | 30,453,790,500 | 30,406,933,800 | 99.85 | 0.03 | 96.74 | 91.69 | 42.91 |
| WBP17 | Female | 32,073,783,300 | 32,018,767,500 | 99.83 | 0.03 | 96.44 | 91.02 | 42.94 |
| WBP18 | Female | 27,133,171,500 | 27,078,219,900 | 99.8 | 0.03 | 97.57 | 93.66 | 42.75 |
| WBP19 | Female | 32,465,796,600 | 32,400,250,500 | 99.8 | 0.03 | 96.32 | 90.78 | 42.98 |
| WBP20 | Female | 38,159,860,200 | 38,088,544,800 | 99.81 | 0.03 | 96.69 | 91.59 | 42.95 |
| WBP21 | Female | 32,643,195,300 | 32,596,963,200 | 99.86 | 0.03 | 96.6 | 91.37 | 42.98 |
| WBP22 | Female | 37,424,541,300 | 37,371,484,200 | 99.86 | 0.03 | 96.31 | 90.71 | 43.03 |
| WBP23 | Male | 26,772,694,500 | 26,734,699,200 | 99.86 | 0.03 | 96.55 | 91.28 | 43.09 |
| WBP24 | Male | 40,228,094,100 | 40,165,712,700 | 99.84 | 0.03 | 96.64 | 91.43 | 42.83 |
| WBP25 | Female | 30,037,797,600 | 29,978,095,200 | 99.8 | 0.03 | 96.58 | 91.63 | 42.48 |
| WP1 | Male | 28,047,710,700 | 28,002,011,700 | 99.84 | 0.03 | 97.3 | 93.02 | 43.19 |
| WP2 | Female | 27,433,068,000 | 27,408,021,300 | 99.91 | 0.03 | 96.36 | 90.81 | 43.63 |
| WP3 | Female | 26,018,602,800 | 25,985,069,700 | 99.87 | 0.03 | 97.17 | 92.66 | 42.64 |
| WP4 | Male | 35,802,969,900 | 35,753,225,700 | 99.86 | 0.03 | 96.59 | 91.31 | 42.78 |
| WP5 | Male | 36,362,629,500 | 36,307,057,500 | 99.85 | 0.03 | 96.65 | 91.47 | 43.07 |
| WP6 | Male | 29,310,889,200 | 29,272,515,000 | 99.87 | 0.03 | 96.88 | 91.99 | 43.23 |
| WP7 | Female | 34,937,565,000 | 34,881,719,100 | 99.84 | 0.03 | 96.63 | 91.42 | 43.01 |
| WP8 | Female | 30,525,851,400 | 30,484,634,700 | 99.86 | 0.03 | 96.57 | 91.31 | 43.14 |
| WP9 | Female | 31,348,135,800 | 31,286,535,300 | 99.8 | 0.03 | 97.46 | 93.41 | 42.83 |
| WP10 | Male | 32,635,532,700 | 32,227,619,700 | 98.75 | 0.03 | 97.22 | 92.95 | 43.17 |
| WP11 | Male | 36,968,646,300 | 36,532,455,600 | 98.82 | 0.03 | 97.27 | 93.03 | 42.93 |
| WP12 | Male | 32,723,311,800 | 32,316,188,400 | 98.76 | 0.03 | 97.14 | 92.78 | 44.3 |
| WP13 | Female | 33,681,280,500 | 33,266,857,200 | 98.77 | 0.03 | 97.21 | 92.94 | 43.83 |
| WP14 | Female | 31,129,609,800 | 30,747,192,600 | 98.77 | 0.03 | 97.2 | 92.93 | 43.73 |
| WP15 | Male | 29,331,950,100 | 28,977,105,600 | 98.79 | 0.03 | 97.29 | 93.13 | 43.28 |
| WP16 | Female | 37,244,152,800 | 36,809,127,300 | 98.83 | 0.03 | 97.4 | 93.29 | 43.08 |
| WP17 | Male | 31,580,778,600 | 31,188,531,900 | 98.76 | 0.03 | 97.09 | 92.64 | 43.03 |
| WP18 | Male | 27,863,365,200 | 27,531,161,100 | 98.81 | 0.03 | 97.25 | 93.03 | 43.09 |
| WP19 | Female | 31,955,002,200 | 31,575,077,100 | 98.81 | 0.03 | 97.25 | 93 | 43.08 |
| WP20 | Male | 31,815,479,100 | 31,760,630,100 | 99.83 | 0.03 | 96.69 | 91.62 | 43.73 |
| WP21 | Female | 26,555,601,900 | 26,513,324,400 | 99.84 | 0.03 | 97.69 | 93.91 | 42.87 |
| WP22 | Male | 28,642,346,700 | 28,600,141,800 | 99.85 | 0.03 | 96.72 | 91.67 | 43.44 |
| WP23 | Female | 26,024,630,400 | 25,990,215,900 | 99.87 | 0.03 | 96.77 | 91.69 | 42.8 |
| WP24 | Female | 27,226,198,800 | 27,189,436,500 | 99.86 | 0.03 | 96.35 | 90.84 | 43.73 |
| WP25 | Male | 30,968,524,200 | 30,924,763,200 | 99.86 | 0.03 | 96.68 | 91.54 | 43.56 |

**Table S4.** The mapping and average depth information of sequencing data

| Sample | Clean_reads | mapped_reads | mapping_rate | Average_depth | Coverage_1X | Coverage_4X |
| --- | --- | --- | --- | --- | --- | --- |
| HBP1 | 237,614,054 | 233,974,857 | 98.47% | 12.05 | 99.22% | 96.84% |
| HBP2 | 205,676,976 | 202,620,563 | 98.51% | 10.25 | 98.81% | 95.85% |
| HBP3 | 200,672,508 | 198,092,763 | 98.71% | 10.43 | 98.81% | 96.23% |
| HBP4 | 236,231,856 | 232,833,520 | 98.56% | 12.00 | 98.89% | 97.49% |
| HBP5 | 210,415,510 | 207,381,273 | 98.56% | 10.65 | 98.83% | 96.28% |
| HBP6 | 219,155,636 | 215,976,545 | 98.55% | 10.89 | 98.86% | 96.47% |
| HBP7 | 211,467,860 | 208,400,002 | 98.55% | 10.83 | 98.77% | 96.40% |
| HBP8 | 229,711,678 | 226,375,992 | 98.55% | 11.43 | 98.83% | 97.02% |
| HBP9 | 189,632,306 | 186,086,282 | 98.13% | 9.83 | 99.07% | 93.92% |
| HBP10 | 185,820,778 | 182,437,846 | 98.18% | 10.10 | 98.77% | 95.74% |
| HBP11 | 200,967,118 | 198,268,910 | 98.66% | 10.36 | 98.87% | 95.86% |
| HBP12 | 293,170,184 | 288,863,104 | 98.53% | 15.24 | 98.98% | 98.33% |
| HBP13 | 197,507,624 | 194,730,600 | 98.59% | 10.23 | 98.87% | 95.83% |
| HBP14 | 193,136,738 | 190,411,792 | 98.59% | 9.80 | 99.16% | 94.10% |
| HBP15 | 197,088,046 | 194,496,906 | 98.69% | 10.11 | 99.13% | 94.74% |
| HBP16 | 187,606,682 | 184,929,156 | 98.57% | 9.71 | 98.80% | 94.63% |
| HBP17 | 182,855,212 | 180,229,797 | 98.56% | 9.61 | 99.06% | 93.36% |
| HBP18 | 223,320,560 | 220,356,340 | 98.67% | 10.83 | 99.20% | 95.78% |
| HBP19 | 199,861,856 | 196,332,146 | 98.23% | 10.70 | 99.21% | 95.73% |
| HBP20 | 203,361,176 | 200,293,162 | 98.49% | 10.20 | 98.84% | 95.50% |
| HBP21 | 259,004,086 | 255,568,509 | 98.67% | 13.31 | 99.24% | 97.64% |
| HBP22 | 205,329,604 | 202,321,376 | 98.53% | 10.49 | 99.12% | 95.20% |
| HBP23 | 253,435,956 | 249,187,595 | 98.32% | 13.24 | 98.88% | 97.84% |
| HBP24 | 172,862,680 | 170,212,570 | 98.47% | 9.05 | 98.92% | 91.61% |
| HBP25 | 239,709,590 | 236,338,340 | 98.59% | 12.04 | 98.92% | 97.51% |
| ASP1 | 209,511,286 | 207,402,658 | 98.99% | 11.18 | 99.16% | 96.33% |
| ASP2 | 189,262,760 | 186,528,563 | 98.56% | 10.29 | 99.06% | 95.28% |
| ASP3 | 192,270,584 | 189,373,074 | 98.49% | 10.27 | 99.10% | 95.23% |
| ASP4 | 187,217,718 | 184,832,229 | 98.73% | 10.20 | 99.11% | 94.94% |
| ASP5 | 182,148,834 | 179,011,087 | 98.28% | 9.96 | 99.15% | 94.60% |
| ASP6 | 186,423,312 | 183,450,684 | 98.41% | 10.05 | 99.13% | 94.66% |
| ASP7 | 177,954,914 | 175,277,736 | 98.50% | 9.66 | 99.01% | 93.90% |
| ASP8 | 181,105,704 | 178,632,905 | 98.63% | 9.90 | 99.02% | 94.48% |
| ASP9 | 173,089,022 | 170,392,239 | 98.44% | 9.46 | 99.01% | 93.31% |
| ASP10 | 182,349,432 | 179,586,159 | 98.48% | 9.93 | 98.96% | 94.44% |
| ASP11 | 172,271,880 | 169,697,517 | 98.51% | 9.41 | 99.01% | 93.42% |
| ASP12 | 178,747,646 | 176,149,126 | 98.55% | 9.70 | 98.73% | 94.97% |
| ASP13 | 214,100,146 | 211,225,612 | 98.66% | 10.80 | 98.74% | 96.60% |
| ASP14 | 172,795,412 | 170,636,188 | 98.75% | 9.08 | 98.69% | 93.72% |
| ASP15 | 230,389,568 | 227,341,540 | 98.68% | 11.47 | 99.21% | 96.66% |
| ASP16 | 203,214,876 | 200,092,259 | 98.46% | 10.39 | 99.08% | 95.13% |
| ASP17 | 195,023,226 | 192,234,578 | 98.57% | 9.95 | 98.79% | 95.47% |
| ASP18 | 197,298,702 | 194,450,468 | 98.56% | 9.99 | 99.03% | 94.39% |
| ASP19 | 193,832,210 | 191,397,233 | 98.74% | 9.89 | 98.69% | 95.34% |
| ASP20 | 264,037,330 | 260,029,276 | 98.48% | 13.63 | 98.91% | 98.04% |
| ASP21 | 229,342,846 | 225,821,783 | 98.46% | 11.91 | 98.88% | 97.42% |
| ASP22 | 228,160,190 | 224,584,213 | 98.43% | 11.95 | 98.85% | 97.43% |
| ASP23 | 230,577,054 | 227,243,349 | 98.55% | 12.02 | 98.90% | 97.53% |
| ASP24 | 191,693,324 | 188,620,018 | 98.40% | 10.16 | 98.62% | 95.53% |
| ASP25 | 238,254,108 | 234,862,574 | 98.58% | 12.42 | 98.76% | 97.50% |
| WSP1 | 174,473,948 | 172,157,681 | 98.67% | 9.11 | 98.79% | 93.91% |
| WSP2 | 203,483,996 | 200,907,816 | 98.73% | 10.33 | 99.09% | 95.23% |
| WSP3 | 217,614,486 | 213,629,904 | 98.17% | 11.52 | 99.00% | 96.09% |
| WSP4 | 202,755,216 | 199,385,384 | 98.34% | 10.66 | 98.64% | 95.98% |
| WSP5 | 178,852,936 | 175,920,888 | 98.36% | 9.58 | 98.56% | 93.84% |
| WSP6 | 191,030,460 | 188,094,329 | 98.46% | 10.17 | 98.66% | 95.64% |
| WSP7 | 212,952,686 | 209,868,776 | 98.55% | 11.18 | 99.05% | 96.03% |
| WSP8 | 176,605,470 | 173,833,767 | 98.43% | 9.37 | 98.59% | 94.04% |
| WSP9 | 205,689,528 | 202,613,202 | 98.50% | 10.84 | 98.68% | 96.45% |
| WSP10 | 247,784,968 | 244,131,397 | 98.53% | 12.82 | 99.10% | 97.25% |
| WSP11 | 194,915,158 | 191,970,521 | 98.49% | 10.25 | 98.66% | 95.72% |
| WSP12 | 208,966,448 | 205,808,243 | 98.49% | 10.98 | 98.73% | 96.63% |
| WSP13 | 184,872,856 | 181,988,531 | 98.44% | 9.89 | 98.92% | 94.12% |
| WSP14 | 180,705,322 | 178,065,407 | 98.54% | 9.36 | 98.89% | 93.12% |
| WSP15 | 188,117,088 | 185,315,809 | 98.51% | 9.65 | 98.88% | 93.63% |
| WSP16 | 182,253,876 | 179,664,268 | 98.58% | 9.32 | 98.60% | 94.05% |
| WSP17 | 176,661,910 | 174,174,608 | 98.59% | 9.07 | 98.61% | 93.44% |
| WSP18 | 186,486,858 | 183,730,626 | 98.52% | 9.68 | 98.64% | 94.90% |
| WSP19 | 182,513,250 | 179,126,478 | 98.14% | 9.50 | 98.91% | 93.40% |
| WSP20 | 179,740,376 | 177,267,720 | 98.62% | 9.41 | 98.89% | 93.21% |
| WSP21 | 179,136,054 | 176,697,119 | 98.64% | 9.39 | 98.90% | 93.21% |
| WSP22 | 193,870,984 | 191,401,193 | 98.73% | 9.73 | 98.65% | 95.09% |
| WSP23 | 181,445,226 | 178,839,967 | 98.56% | 9.65 | 98.69% | 94.94% |
| WSP24 | 225,751,754 | 222,341,950 | 98.49% | 11.75 | 98.66% | 97.11% |
| WSP25 | 175,509,372 | 172,815,178 | 98.46% | 9.43 | 98.56% | 94.19% |
| DYP1 | 215,900,818 | 212,765,348 | 98.55% | 11.33 | 99.01% | 96.21% |
| DYP2 | 196,823,558 | 193,425,856 | 98.27% | 10.37 | 98.75% | 95.70% |
| DYP3 | 214,760,418 | 211,188,065 | 98.34% | 11.27 | 98.77% | 96.76% |
| DYP4 | 177,764,624 | 174,929,171 | 98.40% | 9.54 | 98.74% | 94.26% |
| DYP5 | 225,748,828 | 222,309,704 | 98.48% | 11.69 | 98.82% | 97.26% |
| DYP6 | 215,658,090 | 212,283,121 | 98.44% | 11.23 | 98.77% | 96.94% |
| DYP7 | 194,196,550 | 191,281,079 | 98.50% | 10.27 | 98.71% | 95.87% |
| DYP8 | 217,513,564 | 214,228,687 | 98.49% | 11.33 | 98.82% | 97.08% |
| DYP9 | 191,014,916 | 188,438,905 | 98.65% | 9.92 | 98.98% | 94.40% |
| DYP10 | 197,495,822 | 194,296,109 | 98.38% | 10.39 | 98.70% | 95.99% |
| DYP11 | 201,218,840 | 197,882,767 | 98.34% | 10.58 | 98.74% | 96.34% |
| DYP12 | 176,357,292 | 173,694,885 | 98.49% | 9.41 | 98.68% | 94.31% |
| DYP13 | 243,888,378 | 240,142,585 | 98.46% | 12.53 | 98.82% | 97.66% |
| DYP14 | 209,632,884 | 206,194,542 | 98.36% | 11.01 | 98.75% | 96.50% |
| DYP15 | 172,975,682 | 170,456,210 | 98.54% | 8.86 | 98.62% | 92.65% |
| DYP16 | 197,828,578 | 194,616,850 | 98.38% | 10.25 | 98.78% | 95.71% |
| DYP17 | 204,408,416 | 201,525,454 | 98.59% | 10.80 | 98.74% | 96.45% |
| DYP18 | 239,760,264 | 236,119,790 | 98.48% | 12.33 | 98.82% | 97.53% |
| DYP19 | 199,155,484 | 196,389,156 | 98.61% | 10.53 | 98.72% | 96.15% |
| DYP20 | 275,093,150 | 270,948,295 | 98.49% | 14.07 | 99.21% | 97.84% |
| DYP21 | 205,348,058 | 202,357,549 | 98.54% | 10.82 | 99.12% | 95.85% |
| DYP22 | 240,572,634 | 236,835,189 | 98.45% | 12.43 | 98.81% | 97.59% |
| DYP23 | 251,006,184 | 246,833,392 | 98.34% | 12.94 | 99.20% | 97.47% |
| DYP24 | 218,987,172 | 215,238,044 | 98.29% | 11.38 | 98.73% | 96.97% |
| DYP25 | 231,415,878 | 227,878,969 | 98.47% | 11.88 | 98.81% | 97.36% |
| WBP1 | 236,422,152 | 232,259,920 | 98.24% | 12.22 | 98.80% | 97.29% |
| WBP2 | 264,168,272 | 259,485,630 | 98.23% | 13.66 | 99.14% | 97.51% |
| WBP3 | 230,881,914 | 226,894,892 | 98.27% | 12.08 | 99.08% | 96.67% |
| WBP4 | 261,053,956 | 257,122,385 | 98.49% | 13.52 | 98.80% | 97.83% |
| WBP5 | 220,658,158 | 217,099,706 | 98.39% | 11.35 | 98.70% | 96.86% |
| WBP6 | 229,988,178 | 226,275,445 | 98.39% | 11.89 | 98.75% | 97.18% |
| WBP7 | 214,434,480 | 210,828,272 | 98.32% | 11.13 | 98.72% | 96.74% |
| WBP8 | 195,163,388 | 192,241,036 | 98.50% | 10.32 | 98.69% | 95.85% |
| WBP9 | 208,369,584 | 204,630,416 | 98.21% | 10.89 | 98.69% | 96.40% |
| WBP10 | 233,059,968 | 229,264,933 | 98.37% | 11.91 | 99.05% | 96.65% |
| WBP11 | 189,836,420 | 186,784,815 | 98.39% | 10.00 | 98.69% | 95.43% |
| WBP12 | 260,649,710 | 256,548,710 | 98.43% | 13.44 | 99.14% | 97.54% |
| WBP13 | 212,111,960 | 208,318,661 | 98.21% | 11.18 | 99.01% | 95.81% |
| WBP14 | 207,582,470 | 204,008,445 | 98.28% | 10.96 | 99.02% | 95.55% |
| WBP15 | 218,373,272 | 214,515,004 | 98.23% | 11.30 | 98.69% | 96.49% |
| WBP16 | 202,712,892 | 199,642,246 | 98.49% | 10.76 | 99.00% | 95.61% |
| WBP17 | 213,458,450 | 210,092,190 | 98.42% | 11.15 | 99.05% | 96.11% |
| WBP18 | 180,521,466 | 178,217,162 | 98.72% | 9.20 | 98.61% | 93.84% |
| WBP19 | 216,001,670 | 212,609,898 | 98.43% | 11.30 | 99.06% | 96.21% |
| WBP20 | 253,923,632 | 250,055,175 | 98.48% | 13.02 | 98.78% | 97.63% |
| WBP21 | 217,313,088 | 213,785,661 | 98.38% | 11.41 | 99.09% | 96.35% |
| WBP22 | 249,143,228 | 245,142,419 | 98.39% | 12.86 | 99.14% | 97.30% |
| WBP23 | 178,231,328 | 175,497,347 | 98.47% | 9.54 | 98.63% | 94.48% |
| WBP24 | 267,771,418 | 263,710,977 | 98.48% | 13.54 | 98.80% | 97.84% |
| WBP25 | 199,853,968 | 196,959,900 | 98.55% | 10.68 | 98.73% | 96.46% |
| WP1 | 186,680,078 | 183,880,231 | 98.50% | 9.70 | 99.06% | 93.86% |
| WP2 | 182,720,142 | 179,618,790 | 98.30% | 9.90 | 98.78% | 95.21% |
| WP3 | 173,233,798 | 171,159,414 | 98.80% | 9.14 | 98.69% | 93.80% |
| WP4 | 238,354,838 | 234,676,973 | 98.46% | 12.57 | 99.24% | 97.33% |
| WP5 | 242,047,050 | 238,219,086 | 98.42% | 12.52 | 98.83% | 97.67% |
| WP6 | 195,150,100 | 192,220,307 | 98.50% | 10.42 | 99.13% | 95.24% |
| WP7 | 232,544,794 | 229,173,238 | 98.55% | 12.12 | 98.84% | 97.35% |
| WP8 | 203,230,898 | 200,174,482 | 98.50% | 10.84 | 99.18% | 95.96% |
| WP9 | 208,576,902 | 205,919,324 | 98.73% | 10.63 | 99.21% | 95.85% |
| WP10 | 214,850,798 | 211,587,106 | 98.48% | 11.38 | 98.81% | 97.08% |
| WP11 | 243,549,704 | 240,200,417 | 98.62% | 12.94 | 99.24% | 97.54% |
| WP12 | 215,441,256 | 212,175,320 | 98.48% | 11.53 | 98.87% | 96.78% |
| WP13 | 221,779,048 | 218,177,653 | 98.38% | 11.69 | 98.82% | 97.06% |
| WP14 | 204,981,284 | 201,811,809 | 98.45% | 10.97 | 99.10% | 95.77% |
| WP15 | 193,180,704 | 190,197,103 | 98.46% | 10.41 | 99.17% | 95.27% |
| WP16 | 245,394,182 | 242,210,137 | 98.70% | 12.84 | 99.26% | 97.49% |
| WP17 | 207,923,546 | 204,867,029 | 98.53% | 10.97 | 98.85% | 96.80% |
| WP18 | 183,541,074 | 180,942,566 | 98.58% | 9.89 | 98.80% | 95.39% |
| WP19 | 210,500,514 | 207,623,585 | 98.63% | 11.07 | 98.85% | 96.86% |
| WP20 | 211,737,534 | 207,982,529 | 98.23% | 11.04 | 99.23% | 96.04% |
| WP21 | 176,755,496 | 174,521,276 | 98.74% | 9.06 | 99.01% | 92.27% |
| WP22 | 190,667,612 | 187,670,850 | 98.43% | 10.10 | 99.12% | 94.73% |
| WP23 | 173,268,106 | 170,802,503 | 98.58% | 9.42 | 98.79% | 94.60% |
| WP24 | 181,262,910 | 178,223,162 | 98.32% | 9.67 | 98.78% | 94.68% |
| WP25 | 206,165,088 | 203,051,037 | 98.49% | 10.94 | 99.16% | 95.81% |

**Table S5.** The selection regions of Anhui pig population versus Asian wild boar

| CHROM | BIN_START | BIN_END | log2(θπ ratio) | Fst |
| --- | --- | --- | --- | --- |
| 1 | 41580001 | 41620000 | 0.957381404 | 0.44497 |
| 1 | 41600001 | 41640000 | 1.750350057 | 0.66421 |
| 1 | 41700001 | 41740000 | 0.896152809 | 0.504204 |
| 1 | 41720001 | 41760000 | 1.434590176 | 0.565746 |
| 1 | 53860001 | 53900000 | 1.034389772 | 0.502666 |
| 1 | 73860001 | 73900000 | 1.409078277 | 0.437136 |
| 1 | 73900001 | 73940000 | 1.211582837 | 0.494092 |
| 1 | 73920001 | 73960000 | 0.994376669 | 0.452073 |
| 1 | 74040001 | 74080000 | 0.982583295 | 0.505532 |
| 1 | 77180001 | 77220000 | 1.067248205 | 0.40379 |
| 1 | 79040001 | 79080000 | 2.334143398 | 0.605013 |
| 1 | 79060001 | 79100000 | 2.271018666 | 0.444372 |
| 1 | 93160001 | 93200000 | 0.95208017 | 0.48598 |
| 1 | 93180001 | 93220000 | 1.456203294 | 0.460275 |
| 1 | 93200001 | 93240000 | 1.126851513 | 0.437878 |
| 1 | 93300001 | 93340000 | 0.993996903 | 0.444442 |
| 1 | 93320001 | 93360000 | 1.520475634 | 0.439832 |
| 1 | 93340001 | 93380000 | 1.583737629 | 0.518731 |
| 1 | 93360001 | 93400000 | 1.232666173 | 0.5143 |
| 1 | 93380001 | 93420000 | 1.378313794 | 0.410918 |
| 1 | 93400001 | 93440000 | 1.866313031 | 0.430548 |
| 1 | 93420001 | 93460000 | 1.767207359 | 0.469999 |
| 1 | 93440001 | 93480000 | 1.788657109 | 0.531571 |
| 1 | 93460001 | 93500000 | 1.294707839 | 0.46356 |
| 1 | 94140001 | 94180000 | 1.436982728 | 0.435564 |
| 1 | 94420001 | 94460000 | 1.190605933 | 0.516601 |
| 1 | 101620001 | 101660000 | 0.948967983 | 0.498268 |
| 1 | 101640001 | 101680000 | 0.985956002 | 0.481933 |
| 1 | 105300001 | 105340000 | 1.051937847 | 0.603874 |
| 1 | 105320001 | 105360000 | 1.0339862 | 0.523675 |
| 1 | 108440001 | 108480000 | 1.340631517 | 0.528388 |
| 1 | 108460001 | 108500000 | 0.913541512 | 0.422647 |
| 1 | 110000001 | 110040000 | 1.484339923 | 0.423088 |
| 1 | 110020001 | 110060000 | 1.523021361 | 0.441788 |
| 1 | 118200001 | 118240000 | 1.120730753 | 0.398598 |
| 1 | 126640001 | 126680000 | 1.042023315 | 0.471438 |
| 1 | 126660001 | 126700000 | 1.021738354 | 0.407432 |
| 1 | 132420001 | 132460000 | 1.168439246 | 0.44036 |
| 1 | 132440001 | 132480000 | 1.190120455 | 0.499994 |
| 1 | 133120001 | 133160000 | 0.982944335 | 0.432675 |
| 1 | 136860001 | 136900000 | 1.87978486 | 0.422734 |
| 1 | 136880001 | 136920000 | 1.597830715 | 0.551457 |
| 1 | 149140001 | 149180000 | 0.994798931 | 0.592055 |
| 1 | 149160001 | 149200000 | 1.201764168 | 0.566672 |
| 1 | 149340001 | 149380000 | 1.127676401 | 0.496908 |
| 1 | 149360001 | 149400000 | 1.065848989 | 0.426245 |
| 1 | 155140001 | 155180000 | 1.889884781 | 0.436176 |
| 1 | 155160001 | 155200000 | 1.941694697 | 0.486011 |
| 1 | 169780001 | 169820000 | 1.023598682 | 0.541681 |
| 1 | 170500001 | 170540000 | 0.930270579 | 0.656566 |
| 1 | 176060001 | 176100000 | 1.090817317 | 0.421557 |
| 1 | 179920001 | 179960000 | 0.846387162 | 0.520543 |
| 1 | 179980001 | 180020000 | 1.080445756 | 0.623249 |
| 1 | 180000001 | 180040000 | 0.979786526 | 0.631922 |
| 1 | 180240001 | 180280000 | 1.12917638 | 0.437953 |
| 1 | 180260001 | 180300000 | 1.09939288 | 0.460971 |
| 1 | 180860001 | 180900000 | 1.466639586 | 0.637428 |
| 1 | 180880001 | 180920000 | 0.932109251 | 0.531167 |
| 1 | 184660001 | 184700000 | 0.925445171 | 0.421519 |
| 1 | 184680001 | 184720000 | 1.27289189 | 0.461283 |
| 1 | 188240001 | 188280000 | 0.964306951 | 0.497296 |
| 1 | 199640001 | 199680000 | 2.130200863 | 0.595516 |
| 1 | 199660001 | 199700000 | 1.870946049 | 0.480883 |
| 1 | 201060001 | 201100000 | 0.952273855 | 0.403142 |
| 1 | 202020001 | 202060000 | 1.582336892 | 0.429181 |
| 1 | 202180001 | 202220000 | 1.009369174 | 0.401463 |
| 1 | 202580001 | 202620000 | 1.037163122 | 0.404094 |
| 1 | 205840001 | 205880000 | 1.464866941 | 0.451976 |
| 1 | 211860001 | 211900000 | 1.043210515 | 0.404248 |
| 1 | 216700001 | 216740000 | 1.871648875 | 0.397843 |
| 1 | 216720001 | 216760000 | 1.895113053 | 0.413896 |
| 1 | 216740001 | 216780000 | 1.977757655 | 0.478676 |
| 1 | 216760001 | 216800000 | 0.940982422 | 0.399692 |
| 1 | 216920001 | 216960000 | 1.298483776 | 0.418894 |
| 1 | 216940001 | 216980000 | 2.118990495 | 0.61629 |
| 1 | 216960001 | 217000000 | 1.755390202 | 0.772094 |
| 1 | 216980001 | 217020000 | 1.118663368 | 0.836646 |
| 1 | 217000001 | 217040000 | 0.966780557 | 0.852748 |
| 1 | 217020001 | 217060000 | 0.82499231 | 0.825379 |
| 1 | 219200001 | 219240000 | 1.655735836 | 0.403577 |
| 1 | 220420001 | 220460000 | 0.831767858 | 0.481038 |
| 1 | 221420001 | 221460000 | 0.860946359 | 0.505716 |
| 1 | 221440001 | 221480000 | 1.150722006 | 0.481559 |
| 1 | 234140001 | 234180000 | 0.953899433 | 0.471287 |
| 1 | 234160001 | 234200000 | 0.829655707 | 0.469701 |
| 10 | 33460001 | 33500000 | 0.983592444 | 0.459854 |
| 11 | 19260001 | 19300000 | 0.887964766 | 0.444303 |
| 11 | 39440001 | 39480000 | 1.244933236 | 0.534128 |
| 11 | 39460001 | 39500000 | 1.860522155 | 0.625062 |
| 11 | 39480001 | 39520000 | 1.641764807 | 0.663586 |
| 13 | 38640001 | 38680000 | 0.918625487 | 0.485399 |
| 13 | 38660001 | 38700000 | 1.511366632 | 0.520419 |
| 13 | 38680001 | 38720000 | 1.074682999 | 0.524709 |
| 13 | 41060001 | 41100000 | 0.873756011 | 0.434891 |
| 13 | 60900001 | 60940000 | 1.4144294 | 0.568211 |
| 13 | 60920001 | 60960000 | 1.238237801 | 0.521371 |
| 13 | 86600001 | 86640000 | 1.530227854 | 0.469941 |
| 13 | 86620001 | 86660000 | 1.641766896 | 0.518873 |
| 13 | 86640001 | 86680000 | 1.549138081 | 0.494262 |
| 13 | 88100001 | 88140000 | 1.245526779 | 0.594441 |
| 13 | 88120001 | 88160000 | 1.043021786 | 0.544226 |
| 13 | 92300001 | 92340000 | 1.38220455 | 0.473326 |
| 13 | 92460001 | 92500000 | 0.924268998 | 0.515518 |
| 13 | 92480001 | 92520000 | 1.087533794 | 0.448906 |
| 13 | 92500001 | 92540000 | 0.973714435 | 0.477621 |
| 13 | 92520001 | 92560000 | 1.223726623 | 0.502631 |
| 13 | 92540001 | 92580000 | 1.448594517 | 0.504581 |
| 13 | 92560001 | 92600000 | 1.268722673 | 0.495706 |
| 13 | 92840001 | 92880000 | 0.890686895 | 0.66666 |
| 13 | 96720001 | 96760000 | 0.930615523 | 0.453627 |
| 13 | 96740001 | 96780000 | 1.053522811 | 0.469591 |
| 13 | 104880001 | 104920000 | 2.080082669 | 0.418257 |
| 13 | 107420001 | 107460000 | 1.061282429 | 0.424498 |
| 13 | 107740001 | 107780000 | 1.017805635 | 0.411261 |
| 13 | 108340001 | 108380000 | 1.674178784 | 0.684218 |
| 13 | 108360001 | 108400000 | 1.928019726 | 0.71865 |
| 13 | 108380001 | 108420000 | 1.868683646 | 0.700047 |
| 13 | 108400001 | 108440000 | 1.573858851 | 0.597503 |
| 13 | 108420001 | 108460000 | 1.457264074 | 0.578393 |
| 13 | 108440001 | 108480000 | 1.45123467 | 0.637879 |
| 13 | 108460001 | 108500000 | 1.20290445 | 0.553264 |
| 13 | 110420001 | 110460000 | 0.868760595 | 0.408091 |
| 13 | 110440001 | 110480000 | 0.939963654 | 0.405727 |
| 13 | 110580001 | 110620000 | 0.859555419 | 0.479264 |
| 13 | 110620001 | 110660000 | 0.920210146 | 0.418513 |
| 13 | 118760001 | 118800000 | 1.207323388 | 0.456683 |
| 13 | 119940001 | 119980000 | 0.949379541 | 0.642588 |
| 13 | 125380001 | 125420000 | 1.768047256 | 0.60877 |
| 13 | 125400001 | 125440000 | 2.021829665 | 0.618453 |
| 13 | 128340001 | 128380000 | 0.845515433 | 0.494387 |
| 13 | 140820001 | 140860000 | 1.263244567 | 0.526827 |
| 13 | 140840001 | 140880000 | 2.493920865 | 0.659635 |
| 13 | 140860001 | 140900000 | 2.347279911 | 0.654221 |
| 13 | 140880001 | 140920000 | 1.734829274 | 0.630884 |
| 13 | 148800001 | 148840000 | 1.104437575 | 0.520415 |
| 13 | 162400001 | 162440000 | 1.010495273 | 0.455056 |
| 13 | 165200001 | 165240000 | 1.035685643 | 0.433932 |
| 14 | 48140001 | 48180000 | 1.208062311 | 0.473845 |
| 14 | 48160001 | 48200000 | 1.424942793 | 0.438339 |
| 14 | 48180001 | 48220000 | 1.008655763 | 0.406215 |
| 14 | 48200001 | 48240000 | 0.938054441 | 0.45418 |
| 14 | 48220001 | 48260000 | 1.122880209 | 0.494305 |
| 14 | 48240001 | 48280000 | 1.09778285 | 0.51513 |
| 14 | 48260001 | 48300000 | 0.934479131 | 0.51382 |
| 14 | 48360001 | 48400000 | 1.238315942 | 0.447717 |
| 14 | 48640001 | 48680000 | 1.3046376 | 0.408769 |
| 14 | 48660001 | 48700000 | 1.46035097 | 0.459173 |
| 14 | 49900001 | 49940000 | 0.92834368 | 0.444668 |
| 14 | 49920001 | 49960000 | 1.063245085 | 0.536084 |
| 14 | 50000001 | 50040000 | 0.979153708 | 0.491046 |
| 14 | 50020001 | 50060000 | 0.853983006 | 0.497302 |
| 14 | 50040001 | 50080000 | 1.108700235 | 0.481986 |
| 14 | 50060001 | 50100000 | 1.276270352 | 0.486004 |
| 14 | 50080001 | 50120000 | 1.38411125 | 0.483288 |
| 14 | 50100001 | 50140000 | 1.111616363 | 0.485082 |
| 14 | 50120001 | 50160000 | 0.878671744 | 0.516247 |
| 14 | 50140001 | 50180000 | 1.447608411 | 0.594352 |
| 14 | 50160001 | 50200000 | 2.04319826 | 0.658125 |
| 14 | 50180001 | 50220000 | 2.364898278 | 0.692261 |
| 14 | 50200001 | 50240000 | 2.068097829 | 0.719874 |
| 14 | 50220001 | 50260000 | 1.695333265 | 0.738301 |
| 14 | 50340001 | 50380000 | 1.019170205 | 0.654818 |
| 14 | 50360001 | 50400000 | 1.58637648 | 0.739791 |
| 14 | 50380001 | 50420000 | 1.261630201 | 0.688934 |
| 14 | 50400001 | 50440000 | 1.139456375 | 0.651375 |
| 14 | 50420001 | 50460000 | 1.472984617 | 0.695014 |
| 14 | 50440001 | 50480000 | 1.285386341 | 0.643091 |
| 14 | 50460001 | 50500000 | 1.268198512 | 0.650683 |
| 14 | 50480001 | 50520000 | 1.522637007 | 0.725788 |
| 14 | 50500001 | 50540000 | 1.508055307 | 0.717524 |
| 14 | 50520001 | 50560000 | 1.409637008 | 0.678959 |
| 14 | 50540001 | 50580000 | 1.172259974 | 0.685894 |
| 14 | 50560001 | 50600000 | 0.829277219 | 0.606942 |
| 14 | 50660001 | 50700000 | 1.077868834 | 0.405946 |
| 14 | 56480001 | 56520000 | 1.264549082 | 0.424397 |
| 14 | 56500001 | 56540000 | 1.334176718 | 0.416467 |
| 14 | 66040001 | 66080000 | 1.56140567 | 0.415182 |
| 14 | 66060001 | 66100000 | 1.507961841 | 0.405806 |
| 14 | 70820001 | 70860000 | 1.359919075 | 0.417841 |
| 14 | 70840001 | 70880000 | 1.691391714 | 0.45645 |
| 14 | 70860001 | 70900000 | 1.656456625 | 0.396066 |
| 14 | 76820001 | 76860000 | 1.188288321 | 0.437857 |
| 14 | 76840001 | 76880000 | 0.98720984 | 0.497126 |
| 14 | 76860001 | 76900000 | 1.1565627 | 0.395223 |
| 14 | 76960001 | 77000000 | 1.333916296 | 0.399598 |
| 14 | 82940001 | 82980000 | 1.350626782 | 0.43963 |
| 14 | 82960001 | 83000000 | 1.317937949 | 0.415069 |
| 14 | 101640001 | 101680000 | 0.942612757 | 0.531813 |
| 14 | 101660001 | 101700000 | 0.921645419 | 0.509876 |
| 14 | 119760001 | 119800000 | 1.429945791 | 0.441175 |
| 15 | 37100001 | 37140000 | 0.854088031 | 0.418575 |
| 15 | 62420001 | 62460000 | 1.413488407 | 0.642365 |
| 15 | 68100001 | 68140000 | 1.594077894 | 0.684794 |
| 15 | 68120001 | 68160000 | 2.694862066 | 0.801202 |
| 15 | 68140001 | 68180000 | 2.295125842 | 0.762724 |
| 15 | 68160001 | 68200000 | 2.145649163 | 0.734772 |
| 15 | 68180001 | 68220000 | 2.407027339 | 0.772575 |
| 15 | 68200001 | 68240000 | 1.345851588 | 0.602597 |
| 15 | 68220001 | 68260000 | 1.048260276 | 0.529614 |
| 15 | 70540001 | 70580000 | 1.076211714 | 0.473359 |
| 15 | 93560001 | 93600000 | 1.806129117 | 0.533051 |
| 15 | 93580001 | 93620000 | 2.064401134 | 0.580955 |
| 15 | 93600001 | 93640000 | 1.427144141 | 0.478305 |
| 15 | 93620001 | 93660000 | 1.332650649 | 0.515807 |
| 15 | 94580001 | 94620000 | 1.572202427 | 0.69994 |
| 15 | 94600001 | 94640000 | 1.088352073 | 0.553866 |
| 15 | 94680001 | 94720000 | 1.612658039 | 0.723027 |
| 15 | 94700001 | 94740000 | 0.859150857 | 0.540123 |
| 15 | 98720001 | 98760000 | 1.124565896 | 0.541612 |
| 15 | 98740001 | 98780000 | 1.363808444 | 0.439401 |
| 15 | 98760001 | 98800000 | 1.053640839 | 0.396755 |
| 15 | 105000001 | 105040000 | 0.948013905 | 0.423842 |
| 15 | 105080001 | 105120000 | 0.865139077 | 0.395973 |
| 15 | 105140001 | 105180000 | 1.202376381 | 0.427143 |
| 15 | 113520001 | 113560000 | 0.901762914 | 0.45538 |
| 15 | 113540001 | 113580000 | 0.859766064 | 0.486107 |
| 16 | 39060001 | 39100000 | 0.921684219 | 0.577565 |
| 16 | 54160001 | 54200000 | 1.096439622 | 0.620845 |
| 16 | 54180001 | 54220000 | 1.003323868 | 0.489252 |
| 17 | 31600001 | 31640000 | 1.417697824 | 0.423234 |
| 17 | 31620001 | 31660000 | 1.66244807 | 0.46956 |
| 18 | 18560001 | 18600000 | 1.7750857 | 0.699654 |
| 18 | 18580001 | 18620000 | 2.054295299 | 0.741222 |
| 18 | 18600001 | 18640000 | 0.853640543 | 0.553468 |
| 18 | 23740001 | 23780000 | 1.116622151 | 0.421484 |
| 18 | 23760001 | 23800000 | 1.516156804 | 0.483314 |
| 2 | 5280001 | 5320000 | 0.934790911 | 0.461742 |
| 2 | 18820001 | 18860000 | 0.841123738 | 0.509402 |
| 2 | 63080001 | 63120000 | 1.706852675 | 0.604353 |
| 2 | 63100001 | 63140000 | 1.761620907 | 0.607235 |
| 2 | 64280001 | 64320000 | 0.868453573 | 0.411116 |
| 2 | 64300001 | 64340000 | 0.999930004 | 0.421305 |
| 2 | 66620001 | 66660000 | 0.932900945 | 0.416482 |
| 2 | 66640001 | 66680000 | 1.212618394 | 0.436826 |
| 2 | 93760001 | 93800000 | 0.833625483 | 0.462201 |
| 2 | 93780001 | 93820000 | 0.965339232 | 0.479433 |
| 2 | 104280001 | 104320000 | 1.194347938 | 0.427674 |
| 2 | 109820001 | 109860000 | 1.41956772 | 0.40414 |
| 2 | 110980001 | 111020000 | 1.26212566 | 0.451209 |
| 2 | 111020001 | 111060000 | 1.395955022 | 0.432965 |
| 2 | 111040001 | 111080000 | 1.590313312 | 0.534747 |
| 2 | 111060001 | 111100000 | 1.836867244 | 0.561389 |
| 2 | 112100001 | 112140000 | 0.938886954 | 0.418202 |
| 3 | 42940001 | 42980000 | 1.044424065 | 0.402732 |
| 3 | 64300001 | 64340000 | 0.846710057 | 0.439864 |
| 3 | 83120001 | 83160000 | 1.501197487 | 0.48681 |
| 3 | 83140001 | 83180000 | 1.349384939 | 0.508172 |
| 3 | 83200001 | 83240000 | 1.394079846 | 0.424112 |
| 4 | 22020001 | 22060000 | 1.118939264 | 0.487005 |
| 4 | 50620001 | 50660000 | 1.648315663 | 0.519122 |
| 4 | 52560001 | 52600000 | 1.497057888 | 0.553746 |
| 4 | 52580001 | 52620000 | 1.610286425 | 0.628639 |
| 4 | 52600001 | 52640000 | 0.911090789 | 0.558898 |
| 4 | 52620001 | 52660000 | 0.834726984 | 0.43122 |
| 4 | 53620001 | 53660000 | 1.258088779 | 0.419084 |
| 4 | 53640001 | 53680000 | 1.04704874 | 0.450911 |
| 4 | 53660001 | 53700000 | 0.831172184 | 0.499109 |
| 4 | 54180001 | 54220000 | 1.083178127 | 0.441222 |
| 4 | 54200001 | 54240000 | 1.065292172 | 0.557287 |
| 4 | 54220001 | 54260000 | 0.923804161 | 0.554236 |
| 4 | 68300001 | 68340000 | 1.386573599 | 0.422655 |
| 4 | 80100001 | 80140000 | 1.349251348 | 0.416634 |
| 4 | 80120001 | 80160000 | 1.926989311 | 0.518925 |
| 4 | 80140001 | 80180000 | 1.969235969 | 0.528478 |
| 4 | 91900001 | 91940000 | 0.861122732 | 0.447254 |
| 4 | 101300001 | 101340000 | 1.445336186 | 0.459254 |
| 5 | 18660001 | 18700000 | 1.860938801 | 0.567668 |
| 5 | 18680001 | 18720000 | 1.083397313 | 0.56119 |
| 5 | 29600001 | 29640000 | 1.059452438 | 0.412813 |
| 5 | 29760001 | 29800000 | 1.433900281 | 0.512438 |
| 5 | 29780001 | 29820000 | 1.948809339 | 0.576417 |
| 5 | 29800001 | 29840000 | 1.906541738 | 0.471056 |
| 5 | 29820001 | 29860000 | 1.505673962 | 0.397222 |
| 5 | 30520001 | 30560000 | 1.122663383 | 0.442196 |
| 5 | 32180001 | 32220000 | 0.881141282 | 0.49663 |
| 5 | 50920001 | 50960000 | 1.034958107 | 0.452099 |
| 5 | 51420001 | 51460000 | 1.114795946 | 0.437115 |
| 5 | 51440001 | 51480000 | 1.183417757 | 0.405397 |
| 5 | 52200001 | 52240000 | 0.843631948 | 0.546184 |
| 5 | 52220001 | 52260000 | 0.882635179 | 0.648092 |
| 5 | 52960001 | 53000000 | 0.830863403 | 0.624727 |
| 5 | 54060001 | 54100000 | 0.902045282 | 0.40306 |
| 5 | 54220001 | 54260000 | 1.059052021 | 0.450178 |
| 5 | 54460001 | 54500000 | 1.468237454 | 0.488125 |
| 5 | 54480001 | 54520000 | 1.583593587 | 0.507271 |
| 5 | 54500001 | 54540000 | 1.244053528 | 0.41251 |
| 5 | 61160001 | 61200000 | 0.920846555 | 0.53777 |
| 5 | 61180001 | 61220000 | 2.182042824 | 0.581317 |
| 5 | 89620001 | 89660000 | 1.1035945 | 0.406646 |
| 5 | 89640001 | 89680000 | 1.282365706 | 0.427955 |
| 6 | 47980001 | 48020000 | 1.29113408 | 0.734712 |
| 6 | 48000001 | 48040000 | 1.056005511 | 0.739996 |
| 6 | 52320001 | 52360000 | 0.875783059 | 0.504607 |
| 6 | 69220001 | 69260000 | 1.257994406 | 0.413286 |
| 6 | 69240001 | 69280000 | 1.342676314 | 0.413701 |
| 6 | 104180001 | 104220000 | 1.164726439 | 0.425938 |
| 6 | 114520001 | 114560000 | 0.98375678 | 0.446388 |
| 6 | 122020001 | 122060000 | 1.225461262 | 0.435819 |
| 6 | 122040001 | 122080000 | 1.394861496 | 0.59147 |
| 6 | 122060001 | 122100000 | 1.174754797 | 0.48748 |
| 6 | 123060001 | 123100000 | 0.825798357 | 0.414511 |
| 6 | 123260001 | 123300000 | 1.567954376 | 0.530176 |
| 6 | 123280001 | 123320000 | 1.645597312 | 0.660625 |
| 6 | 123300001 | 123340000 | 1.564915833 | 0.615515 |
| 6 | 126660001 | 126700000 | 0.913485585 | 0.528105 |
| 6 | 129520001 | 129560000 | 1.19427115 | 0.641241 |
| 6 | 129540001 | 129580000 | 1.133734033 | 0.59481 |
| 6 | 129560001 | 129600000 | 0.857418761 | 0.48741 |
| 6 | 130580001 | 130620000 | 1.180583951 | 0.474658 |
| 6 | 135260001 | 135300000 | 0.829633218 | 0.465461 |
| 6 | 135280001 | 135320000 | 1.258077297 | 0.64104 |
| 7 | 57000001 | 57040000 | 1.109056966 | 0.542882 |
| 7 | 60160001 | 60200000 | 0.893241667 | 0.451869 |
| 7 | 72700001 | 72740000 | 1.322421661 | 0.566431 |
| 7 | 72720001 | 72760000 | 1.144939815 | 0.512556 |
| 7 | 74980001 | 75020000 | 0.893880623 | 0.453416 |
| 7 | 91080001 | 91120000 | 1.023488069 | 0.407347 |
| 7 | 91100001 | 91140000 | 1.023411803 | 0.397976 |
| 7 | 95040001 | 95080000 | 1.151150465 | 0.401718 |
| 7 | 100900001 | 100940000 | 1.00288082 | 0.484257 |
| 7 | 100920001 | 100960000 | 0.985372204 | 0.45891 |
| 8 | 25720001 | 25760000 | 1.966510391 | 0.539082 |
| 8 | 25740001 | 25780000 | 1.861896021 | 0.612067 |
| 8 | 45980001 | 46020000 | 1.758747014 | 0.520357 |
| 8 | 46000001 | 46040000 | 2.179570983 | 0.616824 |
| 8 | 46020001 | 46060000 | 1.804162479 | 0.540659 |
| 8 | 46040001 | 46080000 | 1.551436162 | 0.481812 |
| 8 | 46060001 | 46100000 | 1.457715137 | 0.433446 |
| 8 | 46080001 | 46120000 | 1.414933684 | 0.462927 |
| 8 | 46100001 | 46140000 | 1.582279257 | 0.456086 |
| 8 | 46120001 | 46160000 | 1.924750178 | 0.440577 |
| 8 | 46560001 | 46600000 | 1.599315572 | 0.45285 |
| 8 | 46580001 | 46620000 | 1.724282642 | 0.43135 |
| 8 | 49460001 | 49500000 | 2.072803754 | 0.691728 |
| 8 | 49480001 | 49520000 | 2.349265237 | 0.706136 |
| 8 | 49500001 | 49540000 | 1.48774363 | 0.570114 |
| 8 | 49700001 | 49740000 | 0.990644624 | 0.474023 |
| 8 | 49720001 | 49760000 | 1.418153725 | 0.596033 |
| 8 | 51160001 | 51200000 | 0.839970865 | 0.427009 |
| 8 | 51720001 | 51760000 | 0.967989224 | 0.592104 |
| 8 | 61040001 | 61080000 | 0.980928341 | 0.422164 |
| 8 | 61060001 | 61100000 | 1.830456821 | 0.522105 |
| 8 | 61080001 | 61120000 | 2.009660539 | 0.568812 |
| 8 | 61100001 | 61140000 | 2.025210599 | 0.562598 |
| 8 | 61120001 | 61160000 | 1.83003256 | 0.536008 |
| 8 | 61140001 | 61180000 | 1.233544344 | 0.420964 |
| 8 | 61220001 | 61260000 | 1.283438405 | 0.423621 |
| 8 | 61240001 | 61280000 | 1.563364632 | 0.516474 |
| 8 | 61260001 | 61300000 | 1.951249913 | 0.536672 |
| 8 | 61280001 | 61320000 | 2.157613306 | 0.54798 |
| 8 | 61300001 | 61340000 | 2.009205094 | 0.567589 |
| 8 | 61320001 | 61360000 | 1.679086068 | 0.485753 |
| 8 | 61340001 | 61380000 | 1.680041431 | 0.404339 |
| 8 | 61380001 | 61420000 | 1.315287656 | 0.417898 |
| 8 | 61400001 | 61440000 | 1.251807241 | 0.530048 |
| 8 | 61420001 | 61460000 | 0.985481126 | 0.550816 |
| 8 | 61440001 | 61480000 | 0.864344985 | 0.539962 |
| 8 | 61460001 | 61500000 | 1.306803684 | 0.544818 |
| 8 | 61480001 | 61520000 | 1.252794103 | 0.561065 |
| 8 | 61500001 | 61540000 | 1.250540792 | 0.518161 |
| 8 | 61520001 | 61560000 | 1.016745759 | 0.489573 |
| 8 | 61540001 | 61580000 | 0.875311176 | 0.453894 |
| 8 | 63940001 | 63980000 | 0.913447192 | 0.415308 |
| 8 | 64240001 | 64280000 | 1.306518163 | 0.549895 |
| 8 | 64260001 | 64300000 | 1.5919601 | 0.585069 |
| 8 | 64280001 | 64320000 | 1.041487196 | 0.510071 |
| 8 | 67040001 | 67080000 | 0.868369174 | 0.503841 |
| 8 | 67060001 | 67100000 | 1.410517012 | 0.642412 |
| 8 | 67080001 | 67120000 | 1.383003074 | 0.551873 |
| 8 | 67100001 | 67140000 | 1.002194488 | 0.489218 |
| 8 | 67140001 | 67180000 | 0.846234637 | 0.419714 |
| 8 | 67180001 | 67220000 | 1.003595847 | 0.474484 |
| 8 | 67540001 | 67580000 | 1.568363545 | 0.40324 |
| 8 | 67700001 | 67740000 | 0.852272048 | 0.455404 |
| 8 | 68040001 | 68080000 | 1.16478117 | 0.496655 |
| 8 | 68060001 | 68100000 | 1.226321782 | 0.416605 |
| 8 | 68660001 | 68700000 | 0.943158137 | 0.398542 |
| 8 | 70220001 | 70260000 | 1.384764734 | 0.493138 |
| 8 | 70240001 | 70280000 | 1.664745195 | 0.601588 |
| 8 | 82860001 | 82900000 | 1.652098522 | 0.670894 |
| 8 | 82880001 | 82920000 | 1.058770069 | 0.56123 |
| 8 | 82920001 | 82960000 | 1.972985019 | 0.542366 |
| 8 | 85300001 | 85340000 | 1.142684792 | 0.53782 |
| 8 | 85320001 | 85360000 | 1.429236747 | 0.527098 |
| 8 | 85520001 | 85560000 | 1.105693031 | 0.469237 |
| 8 | 94360001 | 94400000 | 0.833566165 | 0.397089 |
| 8 | 94380001 | 94420000 | 1.260951757 | 0.417239 |
| 8 | 108360001 | 108400000 | 0.90893111 | 0.412686 |
| 8 | 115080001 | 115120000 | 0.85964734 | 0.495619 |
| 8 | 115100001 | 115140000 | 0.99829126 | 0.469366 |
| 8 | 116920001 | 116960000 | 0.865481972 | 0.414172 |
| 9 | 47580001 | 47620000 | 1.481031041 | 0.452172 |
| 9 | 57500001 | 57540000 | 0.973970143 | 0.43675 |
| 9 | 75800001 | 75840000 | 0.82890002 | 0.397344 |
| 9 | 75820001 | 75860000 | 0.984204964 | 0.471982 |
| 9 | 75840001 | 75880000 | 1.106844424 | 0.575475 |
| 9 | 75860001 | 75900000 | 1.106010258 | 0.524493 |
| 9 | 85700001 | 85740000 | 1.079348753 | 0.484546 |
| 9 | 91440001 | 91480000 | 1.182616552 | 0.461346 |
| 9 | 91460001 | 91500000 | 1.316173477 | 0.461015 |
| 9 | 101800001 | 101840000 | 1.191731792 | 0.457546 |
| 9 | 116720001 | 116760000 | 1.235191782 | 0.40538 |

**Table S6.** The selected genes harbored in selection regions of Anhui pig population versus Asian wild boar

| GeneID | Genename | GeneFunc |
| --- | --- | --- |
| NM_001001260.1 | PGLYRP1 | peptidoglycan recognition protein 1 |
| NM_001004026.1 | CSN3 | casein kappa |
| NM_001078686.1 | ODAM | odontogenic%2C ameloblast asssociated |
| NM_001105289.1 | COL5A2 | collagen type V alpha 2 chain |
| NM_001112688.1 | CIDEB | cell death-inducing DFFA-like effector b |
| NM_001164021.1 | SLC5A1 | solute carrier family 5 member 1 |
| NM_001167636.1 | NSDHL | NAD(P) dependent steroid dehydrogenase-like |
| NM_001190251.1 | REG4 | regenerating family member 4 |
| NM_001243297.1 | COL3A1 | collagen type III alpha 1 chain |
| NM_001243516.1 | AP3M1 | adaptor related protein complex 3 mu 1 subunit |
| NM_001244511.1 | PRR13 | proline rich 13%2C transcript variant 1 |
| NM_001244932.1 | SUMF1 | sulfatase modifying factor 1 |
| NM_001285967.1 | RB1 | RB transcriptional corepressor 1 |
| NM_001315597.1 | LEMD3 | LEM domain containing 3 |
| NM_213734.1 | APTX | aprataxin |
| NM_213872.1 | RLN2 | relaxin 2 |
| NM_214387.1 | NR0B1 | nuclear receptor subfamily 0 group B member 1 |
| NM_214435.2 | MSTN | myostatin |
| XM_001924990.5 | ATG14 | autophagy related 14 |
| XM_001925479.7 | SEC63 | SEC63 homolog%2C protein translocation regulator%2C transcript variant X1 |
| XM_001926013.5 | INSL6 | insulin like 6 |
| XM_001926562.4 | ADAM30 | ADAM metallopeptidase domain 30 |
| XM_001926818.5 | L2HGDH | L-2-hydroxyglutarate dehydrogenase%2C transcript variant X1 |
| XM_001926857.5 | PPM1F | protein phosphatase%2C Mg2+/Mn2+ dependent 1F%2C transcript variant X1 |
| XM_001926951.4 | TBX18 | T-box 18 |
| XM_001927253.5 | DRG1 | developmentally regulated GTP binding protein 1 |
| XM_001927439.5 | PDK3 | pyruvate dehydrogenase kinase 3%2C transcript variant X1 |
| XM_001927718.4 | NOP9 | NOP9 nucleolar protein |
| XM_001927733.6 | LTB4R2 | leukotriene B4 receptor 2%2C transcript variant X1 |
| XM_001928314.6 | ATP6V1D | ATPase H+ transporting V1 subunit D |
| XM_001928339.4 | EIF2S1 | eukaryotic translation initiation factor 2 subunit alpha |
| XM_001928598.6 | TBR1 | T-box%2C brain 1 |
| XM_001929437.4 | SMARCB1 | SWI/SNF related%2C matrix associated%2C actin dependent regulator of chromatin%2C subfamily b%2C member 1%2C transcript variant X1 |
| XM_001929445.5 | MMP11 | matrix metallopeptidase 11%2C transcript variant X1 |
| XM_001929468.4 | CHCHD10 | coiled-coil-helix-coiled-coil-helix domain containing 10 |
| XM_001929470.5 | VPREB3 | V-set pre-B cell surrogate light chain 3 |
| XM_001929542.5 | SDF2L1 | stromal cell derived factor 2 like 1 |
| XM_001929566.5 | SERPIND1 | serpin family D member 1 |
| XM_001929569.6 | PI4KA | phosphatidylinositol 4-kinase alpha%2C transcript variant X1 |
| XM_003121929.6 | DOCK8 | dedicator of cytokinesis 8 |
| XM_003125515.5 | EIF3H | eukaryotic translation initiation factor 3 subunit H%2C transcript variant X1 |
| XM_003126194.6 | TARBP2 | TARBP2%2C RISC loading complex RNA binding subunit%2C transcript variant X1 |
| XM_003126433.5 | CMAS | cytidine monophosphate N-acetylneuraminic acid synthetase |
| XM_003126445.6 | SLCO1C1 | solute carrier organic anion transporter family member 1C1%2C transcript variant X1 |
| XM_003130084.4 | SNX19 | sorting nexin 19%2C transcript variant X1 |
| XM_003132532.3 | TMEM212 | transmembrane protein 212 |
| XM_003132552.4 | CCDC39 | coiled-coil domain containing 39%2C transcript variant X3 |
| XM_003132656.6 | TMEM39A | transmembrane protein 39A |
| XM_003132993.4 | SNAP29 | synaptosome associated protein 29 |
| XM_003132994.4 | CRKL | CRK like proto-oncogene%2C adaptor protein |
| XM_003132997.4 | LZTR1 | leucine zipper like transcription regulator 1 |
| XM_003132998.4 | THAP7 | THAP domain containing 7%2C transcript variant X1 |
| XM_003133210.6 | GMFG | glia maturation factor gamma%2C transcript variant X1 |
| XM_003133545.5 | C15H2orf88 | chromosome 15 C2orf88 homolog%2C transcript variant X3 |
| XM_003134688.6 | TMEM209 | transmembrane protein 209%2C transcript variant X1 |
| XM_003135049.4 | CHST7 | carbohydrate sulfotransferase 7 |
| XM_003355124.2 | LOC100626877 | olfactory receptor 10R2-like |
| XM_003355574.4 | SMIM10L1 | small integral membrane protein 10 like 1 |
| XM_003358668.4 | ACTRT3 | actin related protein T3 |
| XM_003358669.4 | MYNN | myoneurin%2C transcript variant X1 |
| XM_003359240.4 | ADK | adenosine kinase%2C transcript variant X1 |
| XM_003484018.4 | SSMEM1 | serine rich single-pass membrane protein 1 |
| XM_005654102.3 | LOC100153925 | mitotic-spindle organizing protein 2B%2C transcript variant X1 |
| XM_005655662.3 | LOC100621352 | sulfotransferase 6B1-like |
| XM_005656504.3 | GRIA2 | glutamate ionotropic receptor AMPA type subunit 2%2C transcript variant X7 |
| XM_005656513.3 | RUFY3 | RUN and FYVE domain containing 3%2C transcript variant X1 |
| XM_005660117.3 | PLGRKT | plasminogen receptor with a C-terminal lysine%2C transcript variant X1 |
| XM_005664262.3 | UBE2N | ubiquitin conjugating enzyme E2 N |
| XM_005664266.3 | MRPL42 | mitochondrial ribosomal protein L42%2C transcript variant X2 |
| XM_005666234.3 | DHRS1 | dehydrogenase/reductase 1%2C transcript variant X1 |
| XM_005669930.3 | MBNL1 | muscleblind like splicing regulator 1%2C transcript variant X3 |
| XM_005670867.3 | SFI1 | SFI1 centrin binding protein |
| XM_005670911.3 | LOC100523213 | immunoglobulin lambda-like polypeptide 5%2C transcript variant X1 |
| XM_005670915.3 | PPIL2 | peptidylprolyl isomerase like 2%2C transcript variant X3 |
| XM_005670922.3 | TMEM191C | transmembrane protein 191C%2C transcript variant X1 |
| XM_005673970.3 | CETN2 | centrin 2 |
| XM_013981430.2 | SMPD4 | sphingomyelin phosphodiesterase 4%2C transcript variant X1 |
| XM_013981913.2 | LOC100514009 | histone-lysine N-methyltransferase SETMAR%2C transcript variant X2 |
| XM_013983166.2 | ZNF70 | zinc finger protein 70%2C transcript variant X2 |
| XM_013983173.2 | LOC100155534 | proline-rich receptor-like protein kinase PERK2%2C transcript variant X1 |
| XM_013984188.2 | PSMD14 | proteasome 26S subunit%2C non-ATPase 14%2C transcript variant X2 |
| XM_013986189.2 | WNK3 | WNK lysine deficient protein kinase 3%2C transcript variant X5 |
| XM_013986501.2 | ZNF185 | zinc finger protein 185 with LIM domain%2C transcript variant X6 |
| XM_013986853.2 | MAP3K12 | mitogen-activated protein kinase kinase kinase 12%2C transcript variant X2 |
| XM_013988351.2 | PIK3C2G | phosphatidylinositol-4-phosphate 3-kinase catalytic subunit type 2 gamma%2C transcript variant X2 |
| XM_013988558.2 | SETBP1 | SET binding protein 1%2C transcript variant X2 |
| XM_013989320.2 | SLC4A4 | solute carrier family 4 member 4%2C transcript variant X1 |
| XM_013993522.2 | MLLT3 | MLLT3%2C super elongation complex subunit%2C transcript variant X1 |
| XM_013993593.2 | VLDLR | very low density lipoprotein receptor%2C transcript variant X1 |
| XM_013996640.2 | SLC7A13 | solute carrier family 7 member 13 |
| XM_013999331.2 | FUBP1 | far upstream element binding protein 1%2C transcript variant X1 |
| XM_013999339.2 | DNAJB4 | DnaJ heat shock protein family (Hsp40) member B4%2C transcript variant X1 |
| XM_021062686.1 | SORD | sorbitol dehydrogenase%2C transcript variant X1 |
| XM_021062982.1 | ARHGEF12 | Rho guanine nucleotide exchange factor 12%2C transcript variant X1 |
| XM_021063096.1 | FOCAD | focadhesin%2C transcript variant X2 |
| XM_021063404.1 | ISPD | isoprenoid synthase domain containing%2C transcript variant X1 |
| XM_021065362.1 | PCDH9 | protocadherin 9%2C transcript variant X1 |
| XM_021067261.1 | FBXO34 | F-box protein 34%2C transcript variant X1 |
| XM_021068253.1 | BCL6 | B-cell CLL/lymphoma 6%2C transcript variant X5 |
| XM_021068991.1 | ARHGEF3 | Rho guanine nucleotide exchange factor 3%2C transcript variant X1 |
| XM_021069707.1 | LEKR1 | leucine%2C glutamate and lysine rich 1%2C transcript variant X2 |
| XM_021069763.1 | LRRC34 | leucine rich repeat containing 34%2C transcript variant X1 |
| XM_021069802.1 | FNDC3B | fibronectin type III domain containing 3B%2C transcript variant X3 |
| XM_021069813.1 | LOC110256334 | uncharacterized LOC110256334 |
| XM_021069816.1 | LOC100153543 | multiple epidermal growth factor-like domains protein 6 |
| XM_021070329.1 | ARHGAP31 | Rho GTPase activating protein 31%2C transcript variant X1 |
| XM_021070691.1 | CRYBG3 | crystallin beta-gamma domain containing 3 |
| XM_021071138.1 | MECOM | MDS1 and EVI1 complex locus%2C transcript variant X1 |
| XM_021071922.1 | MAPK1 | mitogen-activated protein kinase 1%2C transcript variant X1 |
| XM_021072282.1 | YPEL1 | yippee like 1%2C transcript variant X2 |
| XM_021072739.1 | TOP3B | topoisomerase (DNA) III beta |
| XM_021072792.1 | HIC2 | HIC ZBTB transcriptional repressor 2 |
| XM_021073419.1 | CCDC74B | coiled-coil domain containing 74B |
| XM_021073445.1 | DCC | DCC netrin 1 receptor%2C transcript variant X1 |
| XM_021073686.1 | C14H22orf15 | chromosome 14 C22orf15 homolog |
| XM_021073687.1 | LOC110256815 | uncharacterized LOC110256815 |
| XM_021073780.1 | ZNF407 | zinc finger protein 407%2C transcript variant X1 |
| XM_021073913.1 | PRR14L | proline rich 14 like%2C transcript variant X2 |
| XM_021073967.1 | MIA2 | melanoma inhibitory activity 2%2C transcript variant X1 |
| XM_021073983.1 | EIF4ENIF1 | eukaryotic translation initiation factor 4E nuclear import factor 1%2C transcript variant X1 |
| XM_021074028.1 | AIFM3 | apoptosis inducing factor%2C mitochondria associated 3%2C transcript variant X1 |
| XM_021074039.1 | P2RX6 | purinergic receptor P2X 6%2C transcript variant X1 |
| XM_021074050.1 | LOC100510930 | tubulin alpha-3 chain%2C transcript variant X1 |
| XM_021074310.1 | PTPRD | protein tyrosine phosphatase%2C receptor type D%2C transcript variant X3 |
| XM_021074380.1 | CTNNA3 | catenin alpha 3%2C transcript variant X1 |
| XM_021074433.1 | LOC100154312 | coiled-coil domain-containing protein 116 |
| XM_021075699.1 | TRAK2 | trafficking kinesin protein 2%2C transcript variant X2 |
| XM_021075700.1 | STRADB | STE20-related kinase adaptor beta%2C transcript variant X4 |
| XM_021075702.1 | C2CD6 | C2 calcium dependent domain containing 6%2C transcript variant X1 |
| XM_021076633.1 | DOCK2 | dedicator of cytokinesis 2%2C transcript variant X1 |
| XM_021076863.1 | PDE4D | phosphodiesterase 4D%2C transcript variant X1 |
| XM_021077210.1 | KDM2A | lysine demethylase 2A%2C transcript variant X1 |
| XM_021078736.1 | CPA2 | carboxypeptidase A2 |
| XM_021079051.1 | WASL | Wiskott-Aldrich syndrome like%2C transcript variant X1 |
| XM_021080387.1 | SLC9A7 | solute carrier family 9 member A7%2C transcript variant X1 |
| XM_021080587.1 | RAI2 | retinoic acid induced 2%2C transcript variant X2 |
| XM_021080777.1 | FAM120C | family with sequence similarity 120C |
| XM_021082806.1 | JAK2 | Janus kinase 2%2C transcript variant X3 |
| XM_021083170.1 | TTC17 | tetratricopeptide repeat domain 17%2C transcript variant X2 |
| XM_021083673.1 | LOC100518417 | adhesion G protein-coupled receptor E2-like%2C transcript variant X2 |
| XM_021083730.1 | CD274 | CD274 molecule%2C transcript variant X3 |
| XM_021083737.1 | LOC100520720 | zinc finger protein 14-like |
| XM_021083741.1 | LOC106508100 | zinc finger protein 791-like%2C transcript variant X1 |
| XM_021085108.1 | LOC110259628 | olfactory receptor-like protein OLF4 |
| XM_021088177.1 | FAM120A | constitutive coactivator of PPAR-gamma-like protein 1%2C transcript variant X1 |
| XM_021088199.1 | CTNNA2 | catenin alpha 2%2C transcript variant X1 |
| XM_021088208.1 | FANCL | Fanconi anemia complementation group L%2C transcript variant X1 |
| XM_021090636.1 | SPIDR | scaffolding protein involved in DNA repair%2C transcript variant X1 |
| XM_021090713.1 | SCML4 | sex comb on midleg like 4 (Drosophila)%2C transcript variant X1 |
| XM_021090817.1 | LOC100624085 | olfactory receptor 10K1-like |
| XM_021091087.1 | SLCO1B3 | solute carrier organic anion transporter family member 1B3%2C transcript variant X1 |
| XM_021091183.1 | PCBP2 | poly(rC) binding protein 2%2C transcript variant X2 |
| XM_021091238.1 | REV3L | REV3 like%2C DNA directed polymerase zeta catalytic subunit%2C transcript variant X1 |
| XM_021091955.1 | MSRB3 | methionine sulfoxide reductase B3%2C transcript variant X1 |
| XM_021091960.1 | IRAK3 | interleukin 1 receptor associated kinase 3 |
| XM_021092211.1 | ETNK1 | ethanolamine kinase 1 |
| XM_021092351.1 | LOC100154902 | taste receptor type 2 member 20-like |
| XM_021094087.1 | USP3 | ubiquitin specific peptidase 3%2C transcript variant X1 |
| XM_021094362.1 | VPS13C | vacuolar protein sorting 13 homolog C%2C transcript variant X1 |
| XM_021094364.1 | LRFN1 | leucine rich repeat and fibronectin type III domain containing 1 |
| XM_021094366.1 | SAMD4B | sterile alpha motif domain containing 4B%2C transcript variant X1 |
| XM_021094599.1 | HIF3A | hypoxia inducible factor 3 alpha subunit%2C transcript variant X1 |
| XM_021095277.1 | RERE | arginine-glutamic acid dipeptide repeats |
| XM_021096140.1 | METTL4 | methyltransferase like 4%2C transcript variant X1 |
| XM_021096361.1 | PRKACB | protein kinase cAMP-activated catalytic subunit beta%2C transcript variant X1 |
| XM_021096440.1 | NEXN | nexilin F-actin binding protein%2C transcript variant X2 |
| XM_021098041.1 | LOC106506784 | basic salivary proline-rich protein 2-like%2C transcript variant X2 |
| XM_021098316.1 | FMN1 | formin 1 |
| XM_021099012.1 | SCAPER | S-phase cyclin A associated protein in the ER%2C transcript variant X1 |
| XM_021099082.1 | NEO1 | neogenin 1%2C transcript variant X1 |
| XM_021099391.1 | PLEK2 | pleckstrin 2%2C transcript variant X1 |
| XM_021099431.1 | SIPA1L1 | signal induced proliferation associated 1 like 1%2C transcript variant X7 |
| XM_021099557.1 | ADCK1 | aarF domain containing kinase 1%2C transcript variant X1 |
| XM_021100455.1 | SMAD1 | SMAD family member 1%2C transcript variant X7 |
| XM_021100487.1 | GLRB | glycine receptor beta%2C transcript variant X3 |
| XM_021100595.1 | DCK | deoxycytidine kinase%2C transcript variant X1 |
| XM_021100671.1 | INPP4B | inositol polyphosphate-4-phosphatase type II B%2C transcript variant X5 |
| XM_021101415.1 | LOC110262119 | mucin-5AC-like |
| XM_021101416.1 | CABS1 | calcium binding protein%2C spermatid associated 1 |
| XM_021101423.1 | ADAMTS3 | ADAM metallopeptidase with thrombospondin type 1 motif 3 |
| XM_021101430.1 | MTHFD2L | methylenetetrahydrofolate dehydrogenase (NADP+ dependent) 2-like |
| XM_021101431.1 | EPGN | epithelial mitogen |
| XM_021101439.1 | SOS2 | SOS Ras/Rho guanine nucleotide exchange factor 2 |
| XM_021101533.1 | MAP4K5 | mitogen-activated protein kinase kinase kinase kinase 5%2C transcript variant X1 |
| XM_021101542.1 | LOC100523624 | methylmalonic aciduria type A protein%2C mitochondrial%2C transcript variant X1 |
| XM_021102398.1 | SLC25A13 | solute carrier family 25 member 13%2C transcript variant X1 |
| XM_021102528.1 | MAGI2 | membrane associated guanylate kinase%2C WW and PDZ domain containing 2%2C transcript variant X1 |
| XM_021102607.1 | RABGAP1L | RAB GTPase activating protein 1 like%2C transcript variant X1 |

**Table S7.** GO analysis of the selected genes of Anhui pig population versus Asian wild boar

| GO accession | Description | pValue | Gene_names |
| --- | --- | --- | --- |
| GO:0051188 | cofactor biosynthetic process | 0.0038423 | NM_001167636.1,XM_005666234.3,XM_021101430.1,XM_021074028.1,XM_021100595.1 |
| GO:0032101 | regulation of response to external stimulus | 0.0041145 | XM_001929445.5,XM_005670922.3 |
| GO:0004488 | methylenetetrahydrofolate dehydrogenase (NADP+) activity | 0.0056504 | XM_005666234.3,XM_021101430.1 |
| GO:0046148 | pigment biosynthetic process | 0.0066919 | XM_001926818.5,XM_021074028.1,NM_001167636.1 |
| GO:0009396 | folic acid-containing compound biosynthetic process | 0.0079944 | XM_021101430.1,XM_005666234.3 |
| GO:0018022 | peptidyl-lysine methylation | 0.0081087 | XM_013981913.2,XM_005673970.3 |
| GO:0034968 | histone lysine methylation | 0.0081087 | XM_013981913.2,XM_005673970.3 |
| GO:0042559 | pteridine-containing compound biosynthetic process | 0.0090359 | XM_005666234.3,XM_021101430.1 |
| GO:0006760 | folic acid-containing compound metabolic process | 0.0092844 | XM_021101430.1,XM_005666234.3 |
| GO:0006720 | isoprenoid metabolic process | 0.0096794 | XM_021063404.1,XM_021074028.1,XM_001926818.5 |
| GO:0008299 | isoprenoid biosynthetic process | 0.0096794 | XM_021063404.1,XM_021074028.1,XM_001926818.5 |
| GO:0044702 | single organism reproductive process | 0.0097286 | XM_021073445.1,XM_003132997.4,XM_021101416.1,XM_021095277.1,XM_021094364.1 |
| GO:0016571 | histone methylation | 0.0098702 | XM_005673970.3,XM_013981913.2 |
| GO:0042440 | pigment metabolic process | 0.010368 | XM_001926818.5,XM_021074028.1,NM_001167636.1 |
| GO:0019354 | siroheme biosynthetic process | 0.011861 | NM_001167636.1,XM_021074028.1 |
| GO:0043115 | precorrin-2 dehydrogenase activity | 0.011861 | NM_001167636.1,XM_021074028.1 |
| GO:0046156 | siroheme metabolic process | 0.011861 | XM_021074028.1,NM_001167636.1 |
| GO:0019953 | sexual reproduction | 0.011978 | XM_003132997.4,XM_021101416.1,XM_021073445.1,XM_021095277.1,XM_021094364.1 |
| GO:0044703 | multi-organism reproductive process | 0.012474 | XM_021095277.1,XM_021094364.1,XM_003132997.4,XM_021101416.1,XM_021073445.1 |
| GO:0042558 | pteridine-containing compound metabolic process | 0.012537 | XM_005666234.3,XM_021101430.1 |
| GO:0016568 | chromatin modification | 0.013426 | XM_001929437.4,XM_013981913.2,XM_005673970.3 |
| GO:0051186 | cofactor metabolic process | 0.013797 | XM_021100595.1,XM_021074028.1,NM_001167636.1,XM_021101430.1,XM_005666234.3 |
| GO:0006783 | heme biosynthetic process | 0.01421 | NM_001167636.1,XM_021074028.1 |
| GO:0005850 | eukaryotic translation initiation factor 2 complex | 0.014945 | XM_001928339.4 |
| GO:0016108 | tetraterpenoid metabolic process | 0.016339 | XM_001926818.5,XM_021074028.1 |
| GO:0016109 | tetraterpenoid biosynthetic process | 0.016339 | XM_001926818.5,XM_021074028.1 |
| GO:0016116 | carotenoid metabolic process | 0.016339 | XM_001926818.5,XM_021074028.1 |
| GO:0016117 | carotenoid biosynthetic process | 0.016339 | XM_021074028.1,XM_001926818.5 |
| GO:0008924 | malate dehydrogenase (quinone) activity | 0.017004 | XM_001926818.5 |
| GO:0002683 | negative regulation of immune system process | 0.017284 | XM_001929445.5 |
| GO:0002698 | negative regulation of immune effector process | 0.017284 | XM_001929445.5 |
| GO:0002832 | negative regulation of response to biotic stimulus | 0.017284 | XM_001929445.5 |
| GO:0030683 | evasion or tolerance by virus of host immune response | 0.017284 | XM_001929445.5 |
| GO:0031348 | negative regulation of defense response | 0.017284 | XM_001929445.5 |
| GO:0043901 | negative regulation of multi-organism process | 0.017284 | XM_001929445.5 |
| GO:0050687 | negative regulation of defense response to virus | 0.017284 | XM_001929445.5 |
| GO:0018193 | peptidyl-amino acid modification | 0.017441 | XM_005670915.3,XM_005673970.3,XM_013981913.2 |
| GO:0034237 | protein kinase A regulatory subunit binding | 0.017497 | XM_003133545.5 |
| GO:0051018 | protein kinase A binding | 0.017497 | XM_003133545.5 |
| GO:0016646 | oxidoreductase activity, acting on the CH-NH group of donors, NAD or NADP as acceptor | 0.017721 | XM_005666234.3,XM_021101430.1 |
| GO:0030570 | pectate lyase activity | 0.017913 | XM_003121929.6 |
| GO:0042168 | heme metabolic process | 0.018762 | NM_001167636.1,XM_021074028.1 |
| GO:0018205 | peptidyl-lysine modification | 0.019097 | XM_005673970.3,XM_013981913.2 |
| GO:0022414 | reproductive process | 0.019166 | XM_021073445.1,XM_003132997.4,XM_021101416.1,XM_021095277.1,XM_021094364.1 |
| GO:0016628 | oxidoreductase activity, acting on the CH-CH group of donors, NAD or NADP as acceptor | 0.021621 | NM_001167636.1,XM_021074028.1 |
| GO:0008745 | N-acetylmuramoyl-L-alanine amidase activity | 0.022207 | NM_001001260.1 |
| GO:0008610 | lipid biosynthetic process | 0.022512 | XM_021073419.1,NM_001167636.1,XM_021063404.1,XM_001926818.5,XM_021074028.1 |
| GO:0002200 | somatic diversification of immune receptors | 0.022917 | XM_013993522.2 |
| GO:0002562 | somatic diversification of immune receptors via germline recombination within a single locus | 0.022917 | XM_013993522.2 |
| GO:0016444 | somatic cell DNA recombination | 0.022917 | XM_013993522.2 |
| GO:0033151 | V(D)J recombination | 0.022917 | XM_013993522.2 |
| GO:0042578 | phosphoric ester hydrolase activity | 0.023144 | XM_013981430.2,XM_021076863.1,XM_021099082.1,XM_021074310.1,XM_021101542.1,XM_021069802.1,XM_013981913.2,NM_214387.1,XM_021073445.1 |
| GO:0008948 | oxaloacetate decarboxylase activity | 0.023643 | XM_021101542.1,XM_021094362.1 |
| GO:0035725 | sodium ion transmembrane transport | 0.023643 | XM_021094362.1,XM_021101542.1 |
| GO:0071436 | sodium ion export | 0.023643 | XM_021094362.1,XM_021101542.1 |
| GO:0050518 | 2-C-methyl-D-erythritol 4-phosphate cytidylyltransferase activity | 0.024079 | XM_021063404.1 |
| GO:0000128 | flocculation | 0.024456 | XM_001929569.6 |
| GO:0098630 | aggregation of unicellular organisms | 0.024456 | XM_001929569.6 |
| GO:0098743 | cell aggregation | 0.024456 | XM_001929569.6 |
| GO:0009108 | coenzyme biosynthetic process | 0.02499 | XM_021100595.1,XM_021101430.1,XM_005666234.3 |
| GO:0002520 | immune system development | 0.026716 | XM_013993522.2 |
| GO:0016645 | oxidoreductase activity, acting on the CH-NH group of donors | 0.026894 | XM_021101430.1,XM_005666234.3 |
| GO:0051015 | actin filament binding | 0.026939 | XM_021088199.1,XM_021074380.1 |
| GO:0050795 | regulation of behavior | 0.027162 | XM_005670922.3 |
| GO:0050920 | regulation of chemotaxis | 0.027162 | XM_005670922.3 |
| GO:0002697 | regulation of immune effector process | 0.028325 | XM_001929445.5 |
| GO:0050688 | regulation of defense response to virus | 0.028325 | XM_001929445.5 |
| GO:0050690 | regulation of defense response to virus by virus | 0.028325 | XM_001929445.5 |
| GO:0006027 | glycosaminoglycan catabolic process | 0.029121 | NM_001001260.1 |
| GO:0009253 | peptidoglycan catabolic process | 0.029121 | NM_001001260.1 |
| GO:0000226 | microtubule cytoskeleton organization | 0.030063 | XM_021100671.1,XM_003132993.4,XM_005670867.3,XM_021075699.1 |
| GO:0050290 | sphingomyelin phosphodiesterase D activity | 0.030917 | XM_013981430.2 |
| GO:0003953 | NAD+ nucleosidase activity | 0.031154 | XM_003121929.6 |
| GO:0016837 | carbon-oxygen lyase activity, acting on polysaccharides | 0.031439 | XM_003121929.6 |
| GO:0007339 | binding of sperm to zona pellucida | 0.031498 | XM_021094364.1,XM_021073445.1 |
| GO:0009988 | cell-cell recognition | 0.031498 | XM_021094364.1,XM_021073445.1 |
| GO:0035036 | sperm-egg recognition | 0.031498 | XM_021094364.1,XM_021073445.1 |
| GO:0072519 | parasitism | 0.03344 | XM_021076633.1 |
| GO:0016278 | lysine N-methyltransferase activity | 0.033829 | XM_005673970.3,XM_013981913.2 |
| GO:0016279 | protein-lysine N-methyltransferase activity | 0.033829 | XM_013981913.2,XM_005673970.3 |
| GO:0018024 | histone-lysine N-methyltransferase activity | 0.033829 | XM_013981913.2,XM_005673970.3 |
| GO:0042054 | histone methyltransferase activity | 0.033829 | XM_005673970.3,XM_013981913.2 |
| GO:0002831 | regulation of response to biotic stimulus | 0.03395 | XM_001929445.5 |
| GO:0008037 | cell recognition | 0.034474 | XM_021094364.1,XM_021073445.1 |
| GO:0008942 | nitrite reductase [NAD(P)H] activity | 0.034569 | XM_021074028.1 |
| GO:0046857 | oxidoreductase activity, acting on other nitrogenous compounds as donors, with NAD or NADP as acceptor | 0.034569 | XM_021074028.1 |
| GO:0071705 | nitrogen compound transport | 0.035738 | XM_005670922.3,XM_021075699.1,XM_021070329.1,XM_013996640.2 |
| GO:0006721 | terpenoid metabolic process | 0.035979 | XM_021074028.1,XM_001926818.5 |
| GO:0016114 | terpenoid biosynthetic process | 0.035979 | XM_001926818.5,XM_021074028.1 |
| GO:0005231 | excitatory extracellular ligand-gated ion channel activity | 0.037603 | XM_005656504.3,XM_021074039.1 |
| GO:0006779 | porphyrin-containing compound biosynthetic process | 0.037663 | XM_021074028.1,NM_001167636.1 |
| GO:0000003 | reproduction | 0.038273 | XM_021073445.1,XM_021101416.1,XM_003132997.4,XM_021094364.1,XM_021099431.1,XM_021095277.1 |
| GO:0031514 | motile cilium | 0.038447 | XM_021075699.1,XM_021102607.1,XM_005670922.3 |
| GO:0032102 | negative regulation of response to external stimulus | 0.038979 | XM_001929445.5 |
| GO:0006479 | protein methylation | 0.039018 | XM_013981913.2,XM_005673970.3 |
| GO:0008213 | protein alkylation | 0.039018 | XM_013981913.2,XM_005673970.3 |
| GO:0016569 | covalent chromatin modification | 0.039409 | XM_005673970.3,XM_013981913.2 |
| GO:0016570 | histone modification | 0.039409 | XM_013981913.2,XM_005673970.3 |
| GO:0008608 | attachment of spindle microtubules to kinetochore | 0.039961 | XM_003132993.4,XM_005670867.3 |
| GO:0042398 | cellular modified amino acid biosynthetic process | 0.040609 | XM_021101430.1,XM_005666234.3 |
| GO:0051704 | multi-organism process | 0.040987 | XM_021076633.1,XM_021100487.1,NM_001243297.1,XM_013993522.2,XM_021101416.1,XM_003132997.4,XM_001929445.5,XM_021094364.1,XM_021095277.1,XM_021073780.1,XM_021102528.1,XM_021096440.1,XM_021073445.1,XM_001929569.6 |
| GO:0019049 | evasion or tolerance of host defenses by virus | 0.041099 | XM_001929445.5 |
| GO:0007338 | single fertilization | 0.041685 | XM_021073445.1,XM_021094364.1 |
| GO:0009566 | fertilization | 0.041685 | XM_021094364.1,XM_021073445.1 |
| GO:0071949 | FAD binding | 0.04283 | XM_001926818.5,XM_021074028.1 |
| GO:0000270 | peptidoglycan metabolic process | 0.042993 | NM_001001260.1 |
| GO:0016661 | oxidoreductase activity, acting on other nitrogenous compounds as donors | 0.043121 | XM_021074028.1 |
| GO:0098809 | nitrite reductase activity | 0.043121 | XM_021074028.1 |
| GO:0005576 | extracellular region | 0.043901 | XM_013993522.2,XM_003132997.4,XM_003132993.4,XM_001929445.5,XM_001926013.5,NM_001004026.1,NM_001243297.1,XM_005673970.3,XM_003358668.4,XM_021073445.1,XM_021094364.1,XM_021083730.1,XM_003121929.6,XM_021074039.1,NM_213872.1 |
| GO:0005929 | cilium | 0.044218 | XM_005670922.3,XM_021075699.1,XM_021102607.1 |
| GO:0016615 | malate dehydrogenase activity | 0.044583 | XM_001926818.5 |
| GO:0006325 | chromatin organization | 0.044622 | XM_013981913.2,XM_005673970.3,XM_001929437.4 |
| GO:0016625 | oxidoreductase activity, acting on the aldehyde or oxo group of donors, iron-sulfur protein as acceptor | 0.044803 | XM_013986853.2 |
| GO:0005887 | integral component of plasma membrane | 0.044963 | XM_021099431.1,XM_021074039.1,XM_021073780.1 |
| GO:0007283 | spermatogenesis | 0.045045 | XM_021095277.1,XM_021101416.1 |
| GO:0048232 | male gamete generation | 0.045045 | XM_021095277.1,XM_021101416.1 |
| GO:0048583 | regulation of response to stimulus | 0.045209 | XM_001929445.5,XM_021101439.1,XM_021068991.1,XM_021062982.1,XM_005670922.3,XM_021099431.1 |
| GO:0031226 | intrinsic component of plasma membrane | 0.045921 | XM_021073780.1,XM_021074039.1,XM_021099431.1 |
| GO:0005230 | extracellular ligand-gated ion channel activity | 0.047889 | XM_021074039.1,XM_021100487.1,XM_005656504.3 |
| GO:0009317 | acetyl-CoA carboxylase complex | 0.048328 | XM_021073419.1 |
| GO:0005581 | collagen trimer | 0.04888 | NM_001243297.1,NM_001105289.1 |
| GO:0034453 | microtubule anchoring | 0.049324 | XM_005670867.3,XM_003132993.4 |
| GO:0008131 | primary amine oxidase activity | 0.049362 | XM_001924990.5 |
| GO:0005637 | nuclear inner membrane | 0.049405 | NM_001315597.1 |
| GO:0005639 | integral component of nuclear inner membrane | 0.049405 | NM_001315597.1 |
| GO:0031229 | intrinsic component of nuclear inner membrane | 0.049405 | NM_001315597.1 |
| GO:0044453 | nuclear membrane part | 0.049405 | NM_001315597.1 |
| GO:0016791 | phosphatase activity | 0.049503 | XM_013981913.2,XM_021069802.1,XM_021099082.1,XM_021074310.1,XM_021101542.1,NM_214387.1,XM_021073445.1 |
| GO:0033743 | peptide-methionine (R)-S-oxide reductase activity | 0.050279 | XM_021091955.1 |
| GO:0051056 | regulation of small GTPase mediated signal transduction | 0.050451 | XM_021099431.1,XM_021062982.1,XM_021101439.1,XM_021068991.1 |
| GO:0019902 | phosphatase binding | 0.051616 | XM_003484018.4 |
| GO:0020012 | evasion or tolerance of host immune response | 0.052178 | XM_001929445.5 |
| GO:0051805 | evasion or tolerance of immune response of other organism involved in symbiotic interaction | 0.052178 | XM_001929445.5 |
| GO:0052564 | response to immune response of other organism involved in symbiotic interaction | 0.052178 | XM_001929445.5 |
| GO:0052572 | response to host immune response | 0.052178 | XM_001929445.5 |
| GO:0007276 | gamete generation | 0.057472 | XM_021095277.1,XM_003132997.4,XM_021101416.1 |
| GO:0003743 | translation initiation factor activity | 0.058223 | XM_001928339.4,XM_003130084.4 |
| GO:0005201 | extracellular matrix structural constituent | 0.060403 | NM_001243297.1,NM_001105289.1 |
| GO:0031347 | regulation of defense response | 0.060535 | XM_001929445.5 |
| GO:0006778 | porphyrin-containing compound metabolic process | 0.060818 | XM_021074028.1,NM_001167636.1 |
| GO:0016901 | oxidoreductase activity, acting on the CH-OH group of donors, quinone or similar compound as acceptor | 0.061322 | XM_001926818.5 |
| GO:0016846 | carbon-sulfur lyase activity | 0.062693 | XM_021091955.1 |
| GO:0005819 | spindle | 0.062889 | XM_003132993.4,XM_021075699.1,XM_005670867.3 |
| GO:0001614 | purinergic nucleotide receptor activity | 0.063931 | XM_021074039.1 |
| GO:0004931 | extracellular ATP-gated cation channel activity | 0.063931 | XM_021074039.1 |
| GO:0014074 | response to purine-containing compound | 0.063931 | XM_021074039.1 |
| GO:0016502 | nucleotide receptor activity | 0.063931 | XM_021074039.1 |
| GO:0033198 | response to ATP | 0.063931 | XM_021074039.1 |
| GO:0035381 | ATP-gated ion channel activity | 0.063931 | XM_021074039.1 |
| GO:0035586 | purinergic receptor activity | 0.063931 | XM_021074039.1 |
| GO:0046683 | response to organophosphorus | 0.063931 | XM_021074039.1 |
| GO:0006732 | coenzyme metabolic process | 0.065794 | XM_005666234.3,XM_021101430.1,XM_021100595.1 |
| GO:0070567 | cytidylyltransferase activity | 0.06747 | XM_021063404.1 |
| GO:0006575 | cellular modified amino acid metabolic process | 0.069964 | XM_005666234.3,XM_021101430.1 |
| GO:0008170 | N-methyltransferase activity | 0.070046 | XM_013981913.2,XM_005673970.3 |
| GO:0000228 | nuclear chromosome | 0.070168 | XM_005670867.3,XM_001929437.4,XM_003132993.4 |
| GO:0048609 | multicellular organismal reproductive process | 0.071342 | XM_021101416.1,XM_003132997.4,XM_021095277.1 |
| GO:0004140 | dephospho-CoA kinase activity | 0.073112 | XM_021100595.1 |
| GO:0042126 | nitrate metabolic process | 0.074057 | XM_021074028.1 |
| GO:0042128 | nitrate assimilation | 0.074057 | XM_021074028.1 |
| GO:0016972 | thiol oxidase activity | 0.074924 | XM_021072282.1 |
| GO:0016053 | organic acid biosynthetic process | 0.075331 | XM_021073419.1,XM_005666234.3,XM_021101430.1 |
| GO:0046394 | carboxylic acid biosynthetic process | 0.075331 | XM_005666234.3,XM_021101430.1,XM_021073419.1 |
| GO:0030682 | evasion or tolerance of host defense response | 0.075652 | XM_001929445.5 |
| GO:0051807 | evasion or tolerance of defense response of other organism involved in symbiotic interaction | 0.075652 | XM_001929445.5 |
| GO:0072686 | mitotic spindle | 0.077566 | XM_005670867.3,XM_003132993.4 |
| GO:0030001 | metal ion transport | 0.077636 | XM_021071922.1,XM_021074028.1,XM_001927253.5,XM_021094362.1,XM_021101542.1,XM_021088199.1,XM_021094364.1 |
| GO:0003993 | acid phosphatase activity | 0.079377 | XM_021099082.1,XM_021074310.1,XM_021073445.1,XM_021069802.1 |
| GO:0032504 | multicellular organism reproduction | 0.07953 | XM_021095277.1,XM_003132997.4,XM_021101416.1 |
| GO:0008146 | sulfotransferase activity | 0.079704 | XM_005655662.3,XM_003135049.4 |
| GO:0010243 | response to organonitrogen compound | 0.080654 | XM_021074039.1 |
| GO:0006303 | double-strand break repair via nonhomologous end joining | 0.081035 | XM_005656513.3 |
| GO:0008276 | protein methyltransferase activity | 0.081165 | XM_013981913.2,XM_005673970.3 |
| GO:0016773 | phosphotransferase activity, alcohol group as acceptor | 0.081367 | XM_021099557.1,XM_021071922.1,XM_021082806.1,XM_021075700.1,XM_021100595.1,XM_021096361.1,XM_013986189.2,XM_021075702.1,XM_013986853.2,XM_013988351.2,XM_021101533.1,XM_021091960.1,XM_001929569.6 |
| GO:0003723 | RNA binding | 0.081626 | XM_021102528.1,XM_021079051.1,XM_003130084.4,XM_021094366.1,XM_021101542.1,XM_001928339.4,XM_021091183.1,XM_013999331.2,XM_001927718.4 |
| GO:0007266 | Rho protein signal transduction | 0.084124 | XM_021068991.1,XM_021101439.1,XM_021062982.1 |
| GO:0035023 | regulation of Rho protein signal transduction | 0.084124 | XM_021068991.1,XM_021101439.1,XM_021062982.1 |
| GO:0005816 | spindle pole body | 0.086231 | XM_021075699.1 |
| GO:0005823 | central plaque of spindle pole body | 0.086231 | XM_021075699.1 |
| GO:0044450 | microtubule organizing center part | 0.086231 | XM_021075699.1 |
| GO:1902531 | regulation of intracellular signal transduction | 0.08694 | XM_021062982.1,XM_021099431.1,XM_021068991.1,XM_021101439.1 |
| GO:0003989 | acetyl-CoA carboxylase activity | 0.08764 | XM_021073419.1 |
| GO:0016421 | CoA carboxylase activity | 0.08764 | XM_021073419.1 |
| GO:0016885 | ligase activity, forming carbon-carbon bonds | 0.08764 | XM_021073419.1 |
| GO:1901698 | response to nitrogen compound | 0.089255 | XM_021074039.1 |
| GO:0014070 | response to organic cyclic compound | 0.089353 | XM_021074039.1,NM_214387.1 |
| GO:0003779 | actin binding | 0.089866 | XM_021079051.1,XM_003133210.6,XM_021074380.1,XM_021088199.1,NM_001285967.1 |
| GO:0006812 | cation transport | 0.090493 | XM_021074028.1,XM_021071922.1,XM_021080387.1,XM_021094362.1,XM_021101542.1,XM_001927253.5,XM_021074039.1,XM_021088199.1,XM_021094364.1 |
| GO:0005088 | Ras guanyl-nucleotide exchange factor activity | 0.090944 | XM_021101439.1,XM_021068991.1,XM_021062982.1 |
| GO:0005089 | Rho guanyl-nucleotide exchange factor activity | 0.090944 | XM_021062982.1,XM_021101439.1,XM_021068991.1 |
| GO:0019752 | carboxylic acid metabolic process | 0.091366 | XM_005666234.3,XM_021101430.1,XM_021073780.1,XM_021073419.1,XM_001926818.5,XM_021074028.1 |
| GO:1901607 | alpha-amino acid biosynthetic process | 0.091832 | XM_005666234.3,XM_021101430.1 |
| GO:0016782 | transferase activity, transferring sulfur-containing groups | 0.092163 | XM_005655662.3,XM_003135049.4 |
| GO:0016614 | oxidoreductase activity, acting on CH-OH group of donors | 0.093446 | NM_001167636.1,XM_001926818.5,XM_021074028.1 |
| GO:0030150 | protein import into mitochondrial matrix | 0.093566 | XM_021102607.1 |
| GO:0006403 | RNA localization | 0.095424 | XM_021075699.1,XM_005670922.3 |
| GO:0015931 | nucleobase-containing compound transport | 0.095424 | XM_005670922.3,XM_021075699.1 |
| GO:0050657 | nucleic acid transport | 0.095424 | XM_005670922.3,XM_021075699.1 |
| GO:0050658 | RNA transport | 0.095424 | XM_005670922.3,XM_021075699.1 |
| GO:0051236 | establishment of RNA localization | 0.095424 | XM_021075699.1,XM_005670922.3 |
| GO:0031965 | nuclear membrane | 0.095674 | NM_001315597.1 |
| GO:0019033 | viral tegument | 0.096737 | XM_013989320.2 |
| GO:0000726 | non-recombinational repair | 0.097505 | XM_005656513.3 |
| GO:0016670 | oxidoreductase activity, acting on a sulfur group of donors, oxygen as acceptor | 0.097611 | XM_021072282.1 |
| GO:0044413 | avoidance of host defenses | 0.098061 | XM_001929445.5 |
| GO:0044415 | evasion or tolerance of host defenses | 0.098061 | XM_001929445.5 |
| GO:0051832 | avoidance of defenses of other organism involved in symbiotic interaction | 0.098061 | XM_001929445.5 |
| GO:0051834 | evasion or tolerance of defenses of other organism involved in symbiotic interaction | 0.098061 | XM_001929445.5 |
| GO:0052173 | response to defenses of other organism involved in symbiotic interaction | 0.098061 | XM_001929445.5 |
| GO:0052200 | response to host defenses | 0.098061 | XM_001929445.5 |
| GO:0075136 | response to host | 0.098061 | XM_001929445.5 |
| GO:0016671 | oxidoreductase activity, acting on a sulfur group of donors, disulfide as acceptor | 0.098453 | XM_021091955.1 |
| GO:0046872 | metal ion binding | 0.10021 | XM_021065362.1,XM_013993593.2,XM_001924990.5,XM_021069816.1,XM_021083737.1,NM_213734.1,XM_021102398.1,XM_001926562.4,XM_021072792.1,XM_013981913.2,XM_021078736.1,XM_021099431.1,XM_021073445.1,XM_021068253.1,XM_021083741.1,XM_021071138.1,XM_021071922.1,XM_021088208.1,XM_021073780.1,XM_021101423.1,XM_005656513.3,XM_005673970.3,XM_021077210.1,XM_005669930.3,XM_013983166.2,XM_001929445.5,XM_021101416.1,XM_021074310.1,XM_021099082.1,XM_021069802.1,XM_021083673.1,XM_013986501.2,XM_021095277.1,XM_021072282.1,XM_021094087.1,XM_003358669.4 |
| GO:0048870 | cell motility | 0.10425 | XM_005670922.3,XM_021102607.1,XM_021075699.1 |
| GO:0051674 | localization of cell | 0.10425 | XM_005670922.3,XM_021102607.1,XM_021075699.1 |
| GO:0043169 | cation binding | 0.10497 | XM_021102398.1,NM_213734.1,XM_021072792.1,XM_001926562.4,XM_001924990.5,XM_013993593.2,XM_021065362.1,XM_021083737.1,XM_021069816.1,XM_021071138.1,XM_021088208.1,XM_021071922.1,XM_021099431.1,XM_021078736.1,XM_013981913.2,XM_021083741.1,XM_021073445.1,XM_021068253.1,XM_005669930.3,XM_021077210.1,XM_005673970.3,XM_021074310.1,XM_021101416.1,XM_021099082.1,XM_001929445.5,XM_013983166.2,XM_021073780.1,XM_005656513.3,XM_021101423.1,XM_021094087.1,XM_003358669.4,XM_013986501.2,XM_021095277.1,XM_021083673.1,XM_021069802.1,XM_021072282.1 |
| GO:0003682 | chromatin binding | 0.10539 | XM_021088199.1,XM_021095277.1 |
| GO:0007224 | smoothened signaling pathway | 0.10556 | XM_005673970.3 |
| GO:0035091 | phosphatidylinositol binding | 0.10558 | XM_013988351.2,XM_003130084.4 |
| GO:0015937 | coenzyme A biosynthetic process | 0.10565 | XM_021100595.1 |
| GO:0033866 | nucleoside bisphosphate biosynthetic process | 0.10565 | XM_021100595.1 |
| GO:0034030 | ribonucleoside bisphosphate biosynthetic process | 0.10565 | XM_021100595.1 |
| GO:0034033 | purine nucleoside bisphosphate biosynthetic process | 0.10565 | XM_021100595.1 |
| GO:0043436 | oxoacid metabolic process | 0.10627 | XM_005666234.3,XM_021101430.1,XM_021073419.1,XM_021073780.1,XM_001926818.5,XM_021074028.1 |
| GO:0016788 | hydrolase activity, acting on ester bonds | 0.10719 | XM_021076863.1,XM_021079051.1,XM_021099082.1,XM_021074310.1,XM_021101542.1,XM_013981430.2,XM_021073445.1,NM_214387.1,XM_021069802.1,XM_013981913.2,XM_021091238.1,XM_001924990.5 |
| GO:0006082 | organic acid metabolic process | 0.10748 | XM_021074028.1,XM_001926818.5,XM_021073419.1,XM_021073780.1,XM_005666234.3,XM_021101430.1 |
| GO:0006996 | organelle organization | 0.10837 | XM_005673970.3,XM_003132993.4,XM_005670867.3,XM_013981913.2,NM_001285967.1,XM_005670922.3,XM_021100671.1,XM_021072739.1,XM_021102607.1,XM_001929437.4,XM_021075699.1 |
| GO:0043900 | regulation of multi-organism process | 0.10878 | XM_001929445.5 |
| GO:0005694 | chromosome | 0.10925 | XM_021095277.1,XM_005673970.3,XM_005670867.3,XM_001929437.4,XM_003132993.4 |
| GO:0008652 | cellular amino acid biosynthetic process | 0.11173 | XM_021101430.1,XM_005666234.3 |
| GO:0048038 | quinone binding | 0.11209 | XM_001924990.5 |
| GO:0008135 | translation factor activity, RNA binding | 0.11281 | XM_001928339.4,XM_003130084.4 |
| GO:1990542 | mitochondrial transmembrane transport | 0.1138 | XM_021102607.1 |
| GO:0043414 | macromolecule methylation | 0.11514 | XM_013981913.2,XM_005673970.3 |
| GO:0000778 | condensed nuclear chromosome kinetochore | 0.1154 | XM_005670867.3,XM_003132993.4 |
| GO:0000780 | condensed nuclear chromosome, centromeric region | 0.1154 | XM_005670867.3,XM_003132993.4 |
| GO:0000942 | condensed nuclear chromosome outer kinetochore | 0.1154 | XM_005670867.3,XM_003132993.4 |
| GO:0042729 | DASH complex | 0.1154 | XM_005670867.3,XM_003132993.4 |
| GO:0044711 | single-organism biosynthetic process | 0.12137 | XM_021101430.1,XM_021063404.1,XM_021100595.1,XM_021074028.1,XM_005666234.3,NM_001167636.1,XM_021073419.1,XM_001926818.5 |
| GO:0000077 | DNA damage checkpoint | 0.12146 | XM_021073419.1 |
| GO:0031570 | DNA integrity checkpoint | 0.12146 | XM_021073419.1 |
| GO:0007265 | Ras protein signal transduction | 0.12209 | XM_021062982.1,XM_021101439.1,XM_021068991.1 |
| GO:0046578 | regulation of Ras protein signal transduction | 0.12209 | XM_021101439.1,XM_021068991.1,XM_021062982.1 |
| GO:0006631 | fatty acid metabolic process | 0.12395 | XM_021073419.1,XM_021074028.1 |
| GO:2001057 | reactive nitrogen species metabolic process | 0.12493 | XM_021074028.1 |
| GO:0042995 | cell projection | 0.12653 | XM_021075699.1,XM_021102607.1,XM_005670922.3 |
| GO:0006099 | tricarboxylic acid cycle | 0.12667 | XM_001926818.5 |
| GO:0006101 | citrate metabolic process | 0.12667 | XM_001926818.5 |
| GO:0048308 | organelle inheritance | 0.12704 | XM_005670922.3 |
| GO:0048309 | endoplasmic reticulum inheritance | 0.12704 | XM_005670922.3 |
| GO:0015936 | coenzyme A metabolic process | 0.12814 | XM_021100595.1 |
| GO:0033865 | nucleoside bisphosphate metabolic process | 0.12814 | XM_021100595.1 |
| GO:0033875 | ribonucleoside bisphosphate metabolic process | 0.12814 | XM_021100595.1 |
| GO:0034032 | purine nucleoside bisphosphate metabolic process | 0.12814 | XM_021100595.1 |
| GO:0015276 | ligand-gated ion channel activity | 0.13144 | XM_005656504.3,XM_021074039.1,XM_021100487.1 |
| GO:0022834 | ligand-gated channel activity | 0.13144 | XM_021074039.1,XM_021100487.1,XM_005656504.3 |
| GO:0006026 | aminoglycan catabolic process | 0.13257 | NM_001001260.1 |
| GO:0031012 | extracellular matrix | 0.13363 | XM_005673970.3,NM_001243297.1,XM_001929445.5,XM_021101423.1 |
| GO:0008677 | 2-dehydropantoate 2-reductase activity | 0.13364 | XM_021074028.1 |
| GO:0045735 | nutrient reservoir activity | 0.13426 | XM_021077210.1 |
| GO:0009607 | response to biotic stimulus | 0.1344 | XM_001929445.5,XM_001926013.5,NM_001243297.1 |
| GO:0016641 | oxidoreductase activity, acting on the CH-NH2 group of donors, oxygen as acceptor | 0.13473 | XM_001924990.5 |
| GO:0044743 | intracellular protein transmembrane import | 0.13567 | XM_021102607.1 |
| GO:0065002 | intracellular protein transmembrane transport | 0.13567 | XM_021102607.1 |
| GO:0004659 | prenyltransferase activity | 0.13739 | NM_214435.2 |
| GO:0016811 | hydrolase activity, acting on carbon-nitrogen (but not peptide) bonds, in linear amides | 0.14076 | NM_001001260.1 |
| GO:0009060 | aerobic respiration | 0.14088 | XM_001926818.5 |
| GO:0015075 | ion transmembrane transporter activity | 0.14118 | XM_001927253.5,XM_021088199.1,XM_021074039.1,XM_021074310.1,XM_013996640.2,XM_021101542.1,XM_021080387.1,XM_021094362.1,XM_021071922.1,XM_005656504.3,XM_021100487.1,XM_013989320.2 |
| GO:0016799 | hydrolase activity, hydrolyzing N-glycosyl compounds | 0.14154 | XM_003121929.6 |
| GO:0009719 | response to endogenous stimulus | 0.14164 | NM_214387.1,XM_021074039.1 |
| GO:0005488 | binding | 0.1417 | XM_005656513.3,XM_013988558.2,XM_013988351.2,NM_001167636.1,XM_021099082.1,XM_021091183.1,XM_021063096.1,XM_021079051.1,XM_001929445.5,XM_013986853.2,XM_013983166.2,XM_005669930.3,XM_021101439.1,XM_001929569.6,XM_001927253.5,XM_021095277.1,XM_013986501.2,XM_021091238.1,XM_021094599.1,XM_021069802.1,XM_003130084.4,XM_021094362.1,XM_021100487.1,XM_021102607.1,XM_021083737.1,XM_021069816.1,XM_021090713.1,XM_001924990.5,XM_013984188.2,XM_021099557.1,XM_003132997.4,XM_013986189.2,XM_021076633.1,XM_021102398.1,XM_021069763.1,XM_013999331.2,XM_021083170.1,XM_021078736.1,XM_021073419.1,XM_013981913.2,XM_003133545.5,XM_021088208.1,XM_013993522.2,XM_003126445.6,XM_021075700.1,XM_021082806.1,XM_021071922.1,XM_021096361.1,XM_003132993.4,XM_021091087.1,XM_021101423.1,XM_003121929.6,XM_021073780.1,XM_005670911.3,XM_021101542.1,XM_021101416.1,XM_021074310.1,XM_021094366.1,XM_005673970.3,XM_021077210.1,XM_001926818.5,XM_021072282.1,XM_021072739.1,XM_003132998.4,XM_021083730.1,XM_021083673.1,NM_001285967.1,XM_003358669.4,XM_021074380.1,XM_001926013.5,XM_021094087.1,XM_021062982.1,XM_021101533.1,XM_003125515.5,XM_021091960.1,XM_003133210.6,XM_021096440.1,XM_021088199.1,XM_013993593.2,NM_001105289.1,XM_021065362.1,XM_005664262.3,XM_021072792.1,XM_021100595.1,XM_001926562.4,XM_021067261.1,XM_001927718.4,NM_213734.1,XM_003132994.4,XM_021102528.1,XM_021083741.1,XM_021073445.1,XM_021068253.1,XM_021099431.1,XM_021094364.1,NM_213872.1,XM_001928339.4,XM_021074028.1,XM_003484018.4,NM_214435.2,XM_021071138.1,XM_021101431.1,NM_001243297.1 |
| GO:0006811 | ion transport | 0.14482 | XM_021100487.1,XM_013989320.2,XM_021080387.1,XM_021094362.1,XM_021074028.1,XM_021071922.1,XM_013996640.2,XM_021101542.1,XM_021074039.1,XM_021094364.1,XM_021088199.1,XM_001927253.5 |
| GO:0005215 | transporter activity | 0.14483 | XM_021101542.1,XM_021074310.1,XM_021080387.1,XM_005656504.3,NM_001164021.1,XM_021088199.1,XM_021074039.1,XM_003126445.6,XM_013996640.2,XM_021071922.1,XM_021094362.1,XM_013989320.2,XM_021100487.1,XM_021091087.1,XM_001927253.5,XM_001928314.6 |
| GO:0005747 | mitochondrial respiratory chain complex I | 0.14489 | XM_021078736.1 |
| GO:0030964 | NADH dehydrogenase complex | 0.14489 | XM_021078736.1 |
| GO:0045271 | respiratory chain complex I | 0.14489 | XM_021078736.1 |
| GO:0072350 | tricarboxylic acid metabolic process | 0.14674 | XM_001926818.5 |
| GO:0005852 | eukaryotic translation initiation factor 3 complex | 0.14933 | XM_003130084.4 |
| GO:0003824 | catalytic activity | 0.15028 | XM_001924990.5,XM_021099557.1,XM_013986189.2,XM_021091955.1,XM_021076633.1,NM_001001260.1,XM_005670922.3,XM_021078736.1,XM_013981913.2,XM_021073419.1,XM_001926857.5,XM_021088208.1,XM_021096361.1,XM_021082806.1,XM_021071922.1,XM_021075700.1,XM_013981430.2,XM_005666234.3,XM_013988351.2,NM_001167636.1,XM_021099082.1,XM_021074050.1,XM_013983166.2,XM_013986853.2,XM_021079051.1,XM_001929445.5,XM_021101439.1,NM_214387.1,XM_001929569.6,XM_005670915.3,XM_021091238.1,XM_021096140.1,XM_021069802.1,XM_021094362.1,XM_021063404.1,XM_021101533.1,XM_021091960.1,XM_005655662.3,XM_021088199.1,XM_021100595.1,XM_001926562.4,XM_021076863.1,XM_021068991.1,XM_003359240.4,XM_021073445.1,XM_021099431.1,XM_021074028.1,XM_003484018.4,NM_214435.2,XM_021101423.1,XM_003121929.6,XM_021073780.1,XM_021074310.1,XM_021101542.1,XM_021101430.1,XM_005673970.3,XM_001926818.5,XM_021072739.1,XM_021072282.1,XM_021083730.1,XM_001928314.6,XM_003135049.4,XM_003358669.4,XM_021075702.1,XM_021094087.1 |
| GO:0016853 | isomerase activity | 0.15126 | XM_021072739.1,XM_005670915.3,XM_021088199.1 |
| GO:0003854 | 3-beta-hydroxy-delta5-steroid dehydrogenase activity | 0.15143 | NM_001167636.1 |
| GO:0016229 | steroid dehydrogenase activity | 0.15143 | NM_001167636.1 |
| GO:0033764 | steroid dehydrogenase activity, acting on the CH-OH group of donors, NAD or NADP as acceptor | 0.15143 | NM_001167636.1 |
| GO:0044255 | cellular lipid metabolic process | 0.15288 | XM_021073419.1,XM_021063404.1,XM_001926818.5,XM_021074028.1 |
| GO:0000413 | protein peptidyl-prolyl isomerization | 0.15294 | XM_005670915.3 |
| GO:0003755 | peptidyl-prolyl cis-trans isomerase activity | 0.15294 | XM_005670915.3 |
| GO:0016859 | cis-trans isomerase activity | 0.15294 | XM_005670915.3 |
| GO:0018208 | peptidyl-proline modification | 0.15294 | XM_005670915.3 |
| GO:0000940 | condensed chromosome outer kinetochore | 0.15301 | XM_005670867.3,XM_003132993.4 |
| GO:0003857 | 3-hydroxyacyl-CoA dehydrogenase activity | 0.15317 | XM_021074028.1 |
| GO:0016831 | carboxy-lyase activity | 0.15322 | XM_021101542.1,XM_021094362.1 |
| GO:0005200 | structural constituent of cytoskeleton | 0.15413 | XM_021075699.1 |
| GO:0008237 | metallopeptidase activity | 0.15427 | XM_021078736.1,XM_001926562.4,XM_021101423.1,XM_001929445.5 |
| GO:0000922 | spindle pole | 0.15574 | XM_021075699.1 |
| GO:0071806 | protein transmembrane transport | 0.15747 | XM_021102607.1 |
| GO:0004672 | protein kinase activity | 0.15774 | XM_021101533.1,XM_021091960.1,XM_013986853.2,XM_021075702.1,XM_013986189.2,XM_021096361.1,XM_021082806.1,XM_021075700.1,XM_021071922.1,XM_021099557.1 |
| GO:0022891 | substrate-specific transmembrane transporter activity | 0.1584 | XM_021088199.1,XM_021074039.1,XM_001927253.5,XM_005656504.3,XM_021100487.1,XM_013989320.2,XM_013996640.2,XM_021074310.1,XM_021101542.1,XM_021080387.1,XM_021094362.1,XM_021071922.1 |
| GO:0008168 | methyltransferase activity | 0.15854 | XM_021096140.1,XM_005673970.3,XM_013981913.2,XM_021101439.1 |
| GO:0022402 | cell cycle process | 0.15929 | XM_021073419.1,XM_021099431.1,XM_003132993.4,XM_021075699.1,XM_005670867.3 |
| GO:0000794 | condensed nuclear chromosome | 0.16072 | XM_005670867.3,XM_003132993.4 |
| GO:0008121 | ubiquinol-cytochrome-c reductase activity | 0.16076 | XM_021074310.1 |
| GO:0016681 | oxidoreductase activity, acting on diphenols and related substances as donors, cytochrome as acceptor | 0.16076 | XM_021074310.1 |
| GO:0022857 | transmembrane transporter activity | 0.16097 | XM_021088199.1,XM_021074039.1,XM_001928314.6,XM_001927253.5,XM_005656504.3,XM_021091087.1,XM_021100487.1,XM_013989320.2,XM_021074310.1,XM_013996640.2,XM_021101542.1,XM_021080387.1,XM_021094362.1,XM_021071922.1 |
| GO:0006338 | chromatin remodeling | 0.16112 | XM_001929437.4 |
| GO:0000777 | condensed chromosome kinetochore | 0.16142 | XM_003132993.4,XM_005670867.3 |
| GO:0000779 | condensed chromosome, centromeric region | 0.16142 | XM_005670867.3,XM_003132993.4 |
| GO:0007017 | microtubule-based process | 0.16216 | XM_021100671.1,XM_005670867.3,XM_021075699.1,XM_003132993.4 |
| GO:0006468 | protein phosphorylation | 0.1627 | XM_021099557.1,XM_013986853.2,XM_021096361.1,XM_013986189.2,XM_021075700.1,XM_021071922.1,XM_021082806.1,XM_021091960.1,XM_021101533.1 |
| GO:0008641 | small protein activating enzyme activity | 0.16364 | XM_021074028.1 |
| GO:0044459 | plasma membrane part | 0.16524 | XM_021099431.1,XM_021074039.1,XM_021073780.1 |
| GO:0016627 | oxidoreductase activity, acting on the CH-CH group of donors | 0.1658 | NM_001167636.1,XM_021074028.1 |
| GO:1902589 | single-organism organelle organization | 0.16622 | XM_003132993.4,XM_005670867.3,XM_021075699.1,XM_005673970.3,XM_013981913.2,XM_021100671.1 |
| GO:0019438 | aromatic compound biosynthetic process | 0.16752 | XM_021073419.1,XM_021094599.1,XM_021099431.1,XM_021095277.1,XM_021068253.1,XM_001929569.6,NM_214387.1,XM_021083741.1,XM_021094362.1,XM_021074028.1,XM_021100455.1,XM_013993522.2,NM_001167636.1,XM_021100671.1,XM_001928598.6,XM_005666234.3,XM_021101423.1,XM_005656513.3,XM_021083737.1,XM_005673970.3,XM_021101430.1,XM_021100595.1,XM_013983166.2,XM_021094366.1,XM_001926951.4 |
| GO:0071941 | nitrogen cycle metabolic process | 0.16888 | XM_021074028.1 |
| GO:0015979 | photosynthesis | 0.16919 | NM_001243297.1,XM_021091183.1 |
| GO:0008081 | phosphoric diester hydrolase activity | 0.1693 | XM_013981430.2,XM_021076863.1 |
| GO:0008757 | S-adenosylmethionine-dependent methyltransferase activity | 0.1698 | XM_005673970.3,XM_013981913.2 |
| GO:0098803 | respiratory chain complex | 0.17082 | XM_021078736.1 |
| GO:0010033 | response to organic substance | 0.17139 | NM_214387.1,XM_021074039.1 |
| GO:0033014 | tetrapyrrole biosynthetic process | 0.17193 | XM_021074028.1,NM_001167636.1 |
| GO:0051276 | chromosome organization | 0.17337 | XM_013981913.2,XM_005673970.3,XM_001929437.4,XM_021072739.1 |
| GO:0044283 | small molecule biosynthetic process | 0.17364 | XM_005666234.3,XM_021101430.1,XM_021073419.1 |
| GO:0098798 | mitochondrial protein complex | 0.17455 | XM_021102607.1,XM_021078736.1 |
| GO:0006464 | cellular protein modification process | 0.17581 | XM_021096361.1,XM_021082806.1,XM_021075700.1,XM_021071922.1,XM_003358669.4,XM_021094087.1,NM_214387.1,XM_013981913.2,XM_005670915.3,XM_013986853.2,XM_013986189.2,XM_021074310.1,XM_021099557.1,XM_005673970.3,XM_021101533.1,XM_021091960.1 |
| GO:0036211 | protein modification process | 0.17581 | XM_021101533.1,XM_021091960.1,XM_013986189.2,XM_013986853.2,XM_021074310.1,XM_021099557.1,XM_005673970.3,NM_214387.1,XM_013981913.2,XM_005670915.3,XM_021096361.1,XM_021082806.1,XM_021075700.1,XM_021071922.1,XM_003358669.4,XM_021094087.1 |
| GO:0034220 | ion transmembrane transport | 0.17589 | XM_021101542.1,XM_013996640.2,XM_021094362.1,XM_021074039.1 |
| GO:0000746 | conjugation | 0.17638 | XM_021073780.1 |
| GO:0018130 | heterocycle biosynthetic process | 0.17671 | XM_021101423.1,XM_005656513.3,XM_021083737.1,NM_001167636.1,XM_001928598.6,XM_021100671.1,XM_005666234.3,XM_013983166.2,XM_021100595.1,XM_021094366.1,XM_001926951.4,XM_005673970.3,XM_021101430.1,XM_021068253.1,XM_001929569.6,NM_214387.1,XM_021083741.1,XM_021073419.1,XM_021094599.1,XM_021099431.1,XM_021095277.1,XM_021094362.1,XM_021074028.1,XM_021100455.1,XM_013993522.2 |
| GO:0098813 | nuclear chromosome segregation | 0.17712 | XM_005670867.3,XM_003132993.4 |
| GO:0000087 | mitotic M phase | 0.18024 | NM_214387.1 |
| GO:0098763 | mitotic cell cycle phase | 0.18024 | NM_214387.1 |
| GO:0032259 | methylation | 0.18045 | XM_013981913.2,XM_005673970.3 |
| GO:0016741 | transferase activity, transferring one-carbon groups | 0.18123 | XM_021101439.1,XM_021096140.1,XM_005673970.3,XM_013981913.2 |
| GO:0005742 | mitochondrial outer membrane translocase complex | 0.18289 | XM_021102607.1 |
| GO:0009896 | positive regulation of catabolic process | 0.18371 | XM_001924990.5,XM_005670922.3 |
| GO:0010508 | positive regulation of autophagy | 0.18371 | XM_001924990.5,XM_005670922.3 |
| GO:0031331 | positive regulation of cellular catabolic process | 0.18371 | XM_001924990.5,XM_005670922.3 |
| GO:0006626 | protein targeting to mitochondrion | 0.18413 | XM_021102607.1 |
| GO:0030474 | spindle pole body duplication | 0.18482 | XM_021075699.1 |
| GO:0051300 | spindle pole body organization | 0.18482 | XM_021075699.1 |
| GO:0050660 | flavin adenine dinucleotide binding | 0.18565 | XM_001926818.5,XM_021074028.1 |
| GO:0006814 | sodium ion transport | 0.18634 | XM_021101542.1,XM_021094362.1 |
| GO:0009966 | regulation of signal transduction | 0.18695 | XM_021068991.1,XM_021101439.1,XM_021099431.1,XM_021062982.1 |
| GO:0016830 | carbon-carbon lyase activity | 0.18695 | XM_021101542.1,XM_021094362.1 |
| GO:0004616 | phosphogluconate dehydrogenase (decarboxylating) activity | 0.18772 | NM_001167636.1 |
| GO:0044455 | mitochondrial membrane part | 0.18831 | XM_021102607.1,XM_021078736.1 |
| GO:0048585 | negative regulation of response to stimulus | 0.19011 | XM_001929445.5 |
| GO:0022836 | gated channel activity | 0.19036 | XM_005656504.3,XM_021100487.1,XM_021074039.1,XM_021071922.1 |
| GO:0098799 | outer mitochondrial membrane protein complex | 0.19058 | XM_021102607.1 |
| GO:0005746 | mitochondrial respiratory chain | 0.19205 | XM_021078736.1 |
| GO:0016829 | lyase activity | 0.19297 | XM_021101542.1,XM_021091955.1,XM_021094362.1,XM_003121929.6 |
| GO:0010646 | regulation of cell communication | 0.19374 | XM_021099431.1,XM_021062982.1,XM_021101439.1,XM_021068991.1 |
| GO:0023051 | regulation of signaling | 0.19495 | XM_021062982.1,XM_021099431.1,XM_021068991.1,XM_021101439.1 |
| GO:0031323 | regulation of cellular metabolic process | 0.1952 | NM_214387.1,XM_021068253.1,XM_001929569.6,XM_021083741.1,XM_021073419.1,XM_021099431.1,XM_021095277.1,XM_005670922.3,XM_021094599.1,XM_021094362.1,XM_013993522.2,XM_021100455.1,XM_005656513.3,XM_021101423.1,XM_021083737.1,XM_001924990.5,XM_001928598.6,XM_021100671.1,XM_021094366.1,XM_001926951.4,XM_005673970.3 |
| GO:1901362 | organic cyclic compound biosynthetic process | 0.19648 | XM_021074028.1,XM_021094362.1,XM_021100455.1,XM_013993522.2,XM_021068253.1,NM_214387.1,XM_001929569.6,XM_021083741.1,XM_021073419.1,XM_021099431.1,XM_021095277.1,XM_021094599.1,XM_021094366.1,XM_013983166.2,XM_021100595.1,XM_001926951.4,XM_005673970.3,XM_021101430.1,XM_005656513.3,XM_021101423.1,XM_021083737.1,NM_001167636.1,XM_005666234.3,XM_021100671.1,XM_001928598.6 |
| GO:0008324 | cation transmembrane transporter activity | 0.1988 | XM_001927253.5,XM_021074039.1,XM_021088199.1,XM_021071922.1,XM_021094362.1,XM_021080387.1,XM_021101542.1,XM_021074310.1 |
| GO:0007029 | endoplasmic reticulum organization | 0.20169 | XM_005670922.3 |
| GO:0015886 | heme transport | 0.203 | XM_021070329.1 |
| GO:0051181 | cofactor transport | 0.203 | XM_021070329.1 |
| GO:1901678 | iron coordination entity transport | 0.203 | XM_021070329.1 |
| GO:0004222 | metalloendopeptidase activity | 0.20404 | XM_001929445.5,XM_001926562.4,XM_021101423.1 |
| GO:0080134 | regulation of response to stress | 0.20494 | XM_001929445.5 |
| GO:0019866 | organelle inner membrane | 0.20545 | NM_001315597.1,XM_021078736.1 |
| GO:0098542 | defense response to other organism | 0.20601 | XM_001929445.5,NM_001243297.1 |
| GO:0015298 | solute:cation antiporter activity | 0.20677 | XM_021080387.1 |
| GO:0015299 | solute:proton antiporter activity | 0.20677 | XM_021080387.1 |
| GO:0005085 | guanyl-nucleotide exchange factor activity | 0.20797 | XM_021068991.1,XM_021101439.1,XM_021062982.1 |
| GO:0007610 | behavior | 0.20847 | XM_005670922.3 |
| GO:0070585 | protein localization to mitochondrion | 0.20946 | XM_021102607.1 |
| GO:0072655 | establishment of protein localization to mitochondrion | 0.20946 | XM_021102607.1 |
| GO:0022892 | substrate-specific transporter activity | 0.20986 | XM_021074039.1,XM_021088199.1,XM_001927253.5,XM_021100487.1,XM_013989320.2,XM_005656504.3,XM_021080387.1,XM_021094362.1,XM_021071922.1,XM_021074310.1,XM_013996640.2,XM_021101542.1 |
| GO:0044454 | nuclear chromosome part | 0.21015 | XM_005670867.3,XM_003132993.4 |
| GO:0033013 | tetrapyrrole metabolic process | 0.21053 | NM_001167636.1,XM_021074028.1 |
| GO:0016877 | ligase activity, forming carbon-sulfur bonds | 0.21067 | XM_021074028.1 |
| GO:0000279 | M phase | 0.21101 | NM_214387.1 |
| GO:0022403 | cell cycle phase | 0.21101 | NM_214387.1 |
| GO:0044848 | biological phase | 0.21101 | NM_214387.1 |
| GO:0016740 | transferase activity | 0.21153 | XM_021101533.1,XM_021091960.1,XM_013988351.2,XM_005655662.3,XM_021100595.1,XM_013986189.2,XM_013986853.2,XM_013983166.2,XM_021099557.1,XM_005673970.3,XM_021076633.1,XM_001929569.6,XM_021101439.1,XM_021068991.1,XM_013981913.2,XM_021096140.1,XM_005670915.3,XM_021075700.1,XM_021082806.1,XM_021071922.1,XM_021075702.1,XM_021096361.1,XM_021088208.1,XM_003358669.4,XM_003135049.4,XM_021063404.1,NM_214435.2 |
| GO:0017048 | Rho GTPase binding | 0.21375 | XM_021076633.1 |
| GO:0015077 | monovalent inorganic cation transmembrane transporter activity | 0.21403 | XM_021074310.1,XM_021101542.1,XM_021080387.1,XM_021094362.1,XM_021071922.1 |
| GO:0006405 | RNA export from nucleus | 0.21551 | XM_021075699.1 |
| GO:0005234 | extracellular-glutamate-gated ion channel activity | 0.2164 | XM_005656504.3 |
| GO:0051536 | iron-sulfur cluster binding | 0.21755 | XM_013986853.2,XM_021074028.1 |
| GO:0051540 | metal cluster binding | 0.21755 | XM_013986853.2,XM_021074028.1 |
| GO:0008509 | anion transmembrane transporter activity | 0.21778 | XM_013989320.2,XM_013996640.2 |
| GO:0004675 | transmembrane receptor protein serine/threonine kinase activity | 0.21823 | XM_021075702.1 |
| GO:0004181 | metallocarboxypeptidase activity | 0.21848 | XM_021078736.1 |
| GO:0006694 | steroid biosynthetic process | 0.2187 | NM_001167636.1 |
| GO:0044281 | small molecule metabolic process | 0.21937 | XM_021101430.1,XM_021100595.1,XM_021074028.1,XM_005666234.3,XM_021073780.1,XM_021073419.1,XM_001926818.5,XM_003359240.4 |
| GO:0030203 | glycosaminoglycan metabolic process | 0.21952 | NM_001001260.1 |
| GO:0051168 | nuclear export | 0.22052 | XM_021075699.1 |
| GO:0007049 | cell cycle | 0.22175 | NM_001285967.1,XM_021073419.1,XM_021099431.1,XM_003132993.4,XM_005670867.3,XM_021075699.1 |
| GO:1901605 | alpha-amino acid metabolic process | 0.2221 | XM_021101430.1,XM_005666234.3 |
| GO:0051537 | 2 iron, 2 sulfur cluster binding | 0.22263 | XM_021074028.1 |
| GO:0015081 | sodium ion transmembrane transporter activity | 0.22367 | XM_021101542.1,XM_021094362.1 |
| GO:0070469 | respiratory chain | 0.22511 | XM_021078736.1 |
| GO:0005543 | phospholipid binding | 0.22691 | XM_013988351.2,XM_003130084.4 |
| GO:0055085 | transmembrane transport | 0.22791 | XM_021074039.1,XM_021088199.1,XM_021102607.1,NM_001164021.1,XM_021091087.1,XM_021080387.1,XM_021094362.1,XM_003126445.6,XM_013996640.2,XM_021101542.1 |
| GO:0000793 | condensed chromosome | 0.22999 | XM_005670867.3,XM_003132993.4 |
| GO:0016310 | phosphorylation | 0.23087 | XM_021091960.1,XM_021101533.1,XM_021099557.1,XM_013986189.2,XM_021096361.1,XM_013986853.2,XM_021071922.1,XM_021082806.1,XM_021075700.1 |
| GO:0006355 | regulation of transcription, DNA-templated | 0.23107 | XM_021083737.1,XM_005656513.3,XM_021101423.1,XM_021100671.1,XM_001928598.6,XM_001926951.4,XM_021094366.1,XM_005673970.3,XM_021083741.1,XM_001929569.6,XM_021068253.1,NM_214387.1,XM_021095277.1,XM_021099431.1,XM_021094599.1,XM_021073419.1,XM_013993522.2,XM_021100455.1,XM_021094362.1 |
| GO:1903506 | regulation of nucleic acid-templated transcription | 0.23107 | XM_021094599.1,XM_021099431.1,XM_021095277.1,XM_021073419.1,XM_021083741.1,XM_021068253.1,NM_214387.1,XM_001929569.6,XM_021100455.1,XM_013993522.2,XM_021094362.1,XM_021100671.1,XM_001928598.6,XM_021083737.1,XM_021101423.1,XM_005656513.3,XM_005673970.3,XM_001926951.4,XM_021094366.1 |
| GO:2001141 | regulation of RNA biosynthetic process | 0.23107 | XM_021083741.1,XM_021068253.1,XM_001929569.6,NM_214387.1,XM_021095277.1,XM_021099431.1,XM_021094599.1,XM_021073419.1,XM_021100455.1,XM_013993522.2,XM_021094362.1,XM_021083737.1,XM_005656513.3,XM_021101423.1,XM_021100671.1,XM_001928598.6,XM_001926951.4,XM_021094366.1,XM_005673970.3 |
| GO:0015630 | microtubule cytoskeleton | 0.23131 | XM_005670867.3,XM_021075699.1,XM_003132993.4 |
| GO:0006839 | mitochondrial transport | 0.23136 | XM_021102607.1 |
| GO:0000776 | kinetochore | 0.2316 | XM_003132993.4,XM_005670867.3 |
| GO:0051252 | regulation of RNA metabolic process | 0.23172 | XM_005673970.3,XM_001926951.4,XM_021094366.1,XM_021100671.1,XM_001928598.6,XM_021083737.1,XM_021101423.1,XM_005656513.3,XM_021100455.1,XM_013993522.2,XM_021094362.1,XM_021094599.1,XM_021099431.1,XM_021095277.1,XM_021073419.1,XM_021083741.1,XM_021068253.1,XM_001929569.6,NM_214387.1 |
| GO:1990204 | oxidoreductase complex | 0.23183 | XM_021078736.1 |
| GO:0002682 | regulation of immune system process | 0.23226 | XM_001929445.5 |
| GO:0010506 | regulation of autophagy | 0.23571 | XM_001924990.5,XM_005670922.3 |
| GO:0055114 | oxidation-reduction process | 0.23613 | XM_001926818.5,XM_021072282.1,XM_001924990.5,XM_005666234.3,NM_001167636.1,XM_021074310.1,XM_021062686.1,XM_013986853.2,XM_021074028.1,XM_021091955.1,XM_021101430.1 |
| GO:0051607 | defense response to virus | 0.23717 | XM_001929445.5 |
| GO:0040012 | regulation of locomotion | 0.23854 | XM_005670922.3 |
| GO:0008092 | cytoskeletal protein binding | 0.2395 | XM_021079051.1,XM_003133210.6,XM_021102607.1,NM_001285967.1,XM_021088199.1,XM_021074380.1 |
| GO:0019219 | regulation of nucleobase-containing compound metabolic process | 0.24043 | XM_021083737.1,XM_021101423.1,XM_005656513.3,XM_021100671.1,XM_001928598.6,XM_001926951.4,XM_021094366.1,XM_005673970.3,XM_021083741.1,NM_214387.1,XM_021068253.1,XM_001929569.6,XM_021094599.1,XM_021099431.1,XM_021095277.1,XM_021073419.1,XM_013993522.2,XM_021100455.1,XM_021094362.1 |
| GO:0007010 | cytoskeleton organization | 0.24137 | XM_005670867.3,XM_021075699.1,XM_003132993.4,XM_021100671.1,NM_001285967.1 |
| GO:0040011 | locomotion | 0.24197 | XM_005670922.3,XM_021075699.1,XM_021102607.1 |
| GO:0008202 | steroid metabolic process | 0.24375 | NM_001167636.1 |
| GO:0015074 | DNA integration | 0.24538 | XM_021094366.1 |
| GO:0031023 | microtubule organizing center organization | 0.24547 | XM_021075699.1 |
| GO:0008235 | metalloexopeptidase activity | 0.24597 | XM_021078736.1 |
| GO:0010556 | regulation of macromolecule biosynthetic process | 0.24773 | XM_021068253.1,XM_001929569.6,NM_214387.1,XM_021083741.1,XM_021073419.1,XM_021095277.1,XM_021099431.1,XM_021094599.1,XM_021094362.1,XM_021100455.1,XM_013993522.2,XM_005656513.3,XM_021101423.1,XM_021083737.1,XM_021100671.1,XM_001928598.6,XM_021094366.1,XM_001926951.4,XM_005673970.3 |
| GO:0031326 | regulation of cellular biosynthetic process | 0.24773 | XM_021073419.1,XM_021099431.1,XM_021095277.1,XM_021094599.1,XM_001929569.6,XM_021068253.1,NM_214387.1,XM_021083741.1,XM_021094362.1,XM_021100455.1,XM_013993522.2,XM_021100671.1,XM_001928598.6,XM_005656513.3,XM_021101423.1,XM_021083737.1,XM_005673970.3,XM_021094366.1,XM_001926951.4 |
| GO:2000112 | regulation of cellular macromolecule biosynthetic process | 0.24773 | XM_005673970.3,XM_001926951.4,XM_021094366.1,XM_021100671.1,XM_001928598.6,XM_021083737.1,XM_005656513.3,XM_021101423.1,XM_013993522.2,XM_021100455.1,XM_021094362.1,XM_021099431.1,XM_021095277.1,XM_021094599.1,XM_021073419.1,XM_021083741.1,XM_021068253.1,NM_214387.1,XM_001929569.6 |
| GO:0009889 | regulation of biosynthetic process | 0.24806 | XM_005673970.3,XM_001926951.4,XM_021094366.1,XM_001928598.6,XM_021100671.1,XM_021083737.1,XM_005656513.3,XM_021101423.1,XM_013993522.2,XM_021100455.1,XM_021094362.1,XM_021099431.1,XM_021095277.1,XM_021094599.1,XM_021073419.1,XM_021083741.1,XM_021068253.1,NM_214387.1,XM_001929569.6 |
| GO:0016616 | oxidoreductase activity, acting on the CH-OH group of donors, NAD or NADP as acceptor | 0.24937 | NM_001167636.1,XM_021074028.1 |
| GO:0017004 | cytochrome complex assembly | 0.24955 | XM_021070329.1 |
| GO:0051171 | regulation of nitrogen compound metabolic process | 0.24998 | XM_021094362.1,XM_013993522.2,XM_021100455.1,NM_214387.1,XM_021068253.1,XM_001929569.6,XM_021083741.1,XM_021073419.1,XM_021094599.1,XM_021099431.1,XM_021095277.1,XM_021094366.1,XM_001926951.4,XM_005673970.3,XM_021101423.1,XM_005656513.3,XM_021083737.1,XM_001928598.6,XM_021100671.1 |
| GO:0019222 | regulation of metabolic process | 0.25332 | XM_021083741.1,XM_021068253.1,XM_001929569.6,NM_214387.1,XM_005670922.3,XM_021094599.1,XM_021099431.1,XM_021095277.1,XM_021073419.1,XM_013993522.2,XM_021100455.1,XM_021094362.1,XM_021083737.1,XM_021101423.1,XM_005656513.3,XM_021100671.1,XM_001928598.6,XM_001924990.5,XM_001926951.4,XM_021094366.1,XM_005673970.3 |
| GO:0022890 | inorganic cation transmembrane transporter activity | 0.25349 | XM_021080387.1,XM_021094362.1,XM_001927253.5,XM_021071922.1,XM_021074310.1,XM_021101542.1,XM_021088199.1 |
| GO:0032787 | monocarboxylic acid metabolic process | 0.25477 | XM_021073419.1,XM_021074028.1 |
| GO:0005635 | nuclear envelope | 0.25487 | NM_001315597.1 |
| GO:0015297 | antiporter activity | 0.25529 | XM_021080387.1 |
| GO:0016638 | oxidoreductase activity, acting on the CH-NH2 group of donors | 0.25738 | XM_001924990.5 |
| GO:0004114 | 3',5'-cyclic-nucleotide phosphodiesterase activity | 0.25797 | XM_021076863.1 |
| GO:0009605 | response to external stimulus | 0.25878 | XM_001929445.5,XM_005670922.3,NM_001243297.1 |
| GO:0010468 | regulation of gene expression | 0.25948 | XM_021099431.1,XM_021095277.1,XM_021094599.1,XM_021073419.1,XM_021083741.1,XM_021068253.1,XM_001929569.6,NM_214387.1,XM_021100455.1,XM_013993522.2,XM_021094362.1,XM_001928598.6,XM_021100671.1,XM_021083737.1,XM_005656513.3,XM_021101423.1,XM_005673970.3,XM_001926951.4,XM_021094366.1 |
| GO:0031967 | organelle envelope | 0.25967 | NM_001315597.1,XM_021102607.1,XM_021078736.1 |
| GO:0007059 | chromosome segregation | 0.26014 | XM_003132993.4,XM_005670867.3 |
| GO:0006813 | potassium ion transport | 0.26202 | XM_021071922.1,XM_021074028.1 |
| GO:0050909 | sensory perception of taste | 0.26228 | XM_021092351.1 |
| GO:0005516 | calmodulin binding | 0.26251 | XM_021100487.1 |
| GO:0005801 | cis-Golgi network | 0.26358 | XM_021099431.1 |
| GO:0000041 | transition metal ion transport | 0.26638 | XM_021094364.1,XM_001927253.5 |
| GO:0015171 | amino acid transmembrane transporter activity | 0.26657 | XM_013996640.2 |
| GO:0006302 | double-strand break repair | 0.26756 | XM_005656513.3 |
| GO:0006629 | lipid metabolic process | 0.2685 | XM_021063404.1,NM_001167636.1,XM_021073419.1,XM_021074028.1,XM_001926818.5 |
| GO:0000786 | nucleosome | 0.27156 | XM_021095277.1 |
| GO:0016667 | oxidoreductase activity, acting on a sulfur group of donors | 0.2719 | XM_021091955.1,XM_021072282.1 |
| GO:0016679 | oxidoreductase activity, acting on diphenols and related substances as donors | 0.27226 | XM_021074310.1 |
| GO:1901136 | carbohydrate derivative catabolic process | 0.27311 | NM_001001260.1 |
| GO:0002252 | immune effector process | 0.27317 | XM_001929445.5 |
| GO:0043167 | ion binding | 0.27357 | XM_021073445.1,XM_021068253.1,XM_021083741.1,XM_013981913.2,XM_021099431.1,XM_021078736.1,XM_021082806.1,XM_021074028.1,XM_021075700.1,XM_021071922.1,XM_021096361.1,XM_021088208.1,XM_021071138.1,XM_021069816.1,XM_021101533.1,XM_021091960.1,XM_021083737.1,XM_021065362.1,XM_001924990.5,XM_013993593.2,XM_021100595.1,XM_013986189.2,XM_001926562.4,XM_021099557.1,XM_021072792.1,NM_213734.1,XM_021102398.1,XM_021072282.1,XM_001927253.5,XM_001926818.5,XM_021069802.1,XM_013986501.2,XM_021095277.1,XM_021083673.1,XM_003130084.4,XM_003358669.4,XM_021094087.1,XM_005656513.3,XM_021101423.1,XM_021073780.1,XM_013988351.2,XM_001929445.5,XM_013983166.2,XM_013986853.2,XM_021101542.1,XM_021101416.1,XM_021074310.1,XM_021099082.1,XM_021077210.1,XM_005673970.3,XM_005669930.3 |
| GO:0098655 | cation transmembrane transport | 0.27565 | XM_021094362.1,XM_021101542.1,XM_021074039.1 |
| GO:1901700 | response to oxygen-containing compound | 0.27621 | XM_021074039.1 |
| GO:0006265 | DNA topological change | 0.27664 | XM_021072739.1 |
| GO:0019012 | virion | 0.27791 | XM_005656513.3,XM_001929445.5,NM_001285967.1,XM_013989320.2,XM_005670922.3,XM_021095277.1 |
| GO:0042221 | response to chemical | 0.28069 | XM_005670922.3,XM_021074039.1,NM_214387.1 |
| GO:0044421 | extracellular region part | 0.28176 | XM_003358668.4,XM_003132993.4,XM_005673970.3,NM_001243297.1 |
| GO:0004180 | carboxypeptidase activity | 0.28534 | XM_021078736.1 |
| GO:0004725 | protein tyrosine phosphatase activity | 0.28659 | NM_214387.1,XM_021074310.1 |
| GO:0003333 | amino acid transmembrane transport | 0.28688 | XM_013996640.2 |
| GO:0006865 | amino acid transport | 0.28688 | XM_013996640.2 |
| GO:0043207 | response to external biotic stimulus | 0.28809 | XM_001929445.5,NM_001243297.1 |
| GO:0051707 | response to other organism | 0.28809 | NM_001243297.1,XM_001929445.5 |
| GO:0006633 | fatty acid biosynthetic process | 0.2912 | XM_021073419.1 |
| GO:0016903 | oxidoreductase activity, acting on the aldehyde or oxo group of donors | 0.29173 | XM_013986853.2 |
| GO:0032993 | protein-DNA complex | 0.2919 | XM_021095277.1 |
| GO:1903825 | organic acid transmembrane transport | 0.29217 | XM_013996640.2 |
| GO:0043412 | macromolecule modification | 0.29339 | XM_005670915.3,XM_013981913.2,NM_214387.1,XM_021094087.1,XM_003358669.4,XM_021071922.1,XM_021082806.1,XM_021075700.1,XM_021096361.1,XM_021101533.1,XM_021091960.1,XM_005673970.3,XM_021099557.1,XM_021074310.1,XM_013986853.2,XM_013986189.2 |
| GO:0004112 | cyclic-nucleotide phosphodiesterase activity | 0.29414 | XM_021076863.1 |
| GO:0016874 | ligase activity | 0.29558 | XM_021073780.1,XM_021073419.1,XM_021074028.1 |
| GO:0019899 | enzyme binding | 0.29572 | XM_021076633.1,XM_003484018.4 |
| GO:0044815 | DNA packaging complex | 0.2964 | XM_021095277.1 |
| GO:0005578 | proteinaceous extracellular matrix | 0.29685 | NM_001243297.1,XM_005673970.3 |
| GO:0098656 | anion transmembrane transport | 0.29833 | XM_013996640.2 |
| GO:0007606 | sensory perception of chemical stimulus | 0.29981 | XM_021092351.1 |
| GO:0098519 | nucleotide phosphatase activity, acting on free nucleotides | 0.30038 | XM_021101542.1 |
| GO:0044271 | cellular nitrogen compound biosynthetic process | 0.30203 | XM_021071138.1,XM_013993522.2,XM_021100455.1,XM_021074028.1,XM_021094362.1,XM_021095277.1,XM_021099431.1,XM_021094599.1,XM_021073419.1,XM_021083741.1,XM_001929569.6,XM_021068253.1,NM_214387.1,XM_021101430.1,XM_005673970.3,XM_001926951.4,XM_021094366.1,XM_013983166.2,XM_021100595.1,XM_005666234.3,XM_001928598.6,XM_021100671.1,NM_001167636.1,XM_021083737.1,XM_005656513.3,XM_021101423.1 |
| GO:0005509 | calcium ion binding | 0.3039 | XM_021065362.1,XM_005673970.3,XM_021083673.1,XM_021102398.1,XM_013993593.2,XM_021069816.1,XM_021101416.1 |
| GO:0016772 | transferase activity, transferring phosphorus-containing groups | 0.30498 | XM_001929569.6,XM_021096361.1,XM_021075702.1,XM_021082806.1,XM_021071922.1,XM_021075700.1,XM_003358669.4,XM_021063404.1,XM_021101533.1,XM_021091960.1,XM_013988351.2,XM_013986853.2,XM_021100595.1,XM_013986189.2,XM_013983166.2,XM_021099557.1 |
| GO:0080090 | regulation of primary metabolic process | 0.30515 | XM_021101423.1,XM_005656513.3,XM_021083737.1,XM_001928598.6,XM_021100671.1,XM_021094366.1,XM_001926951.4,XM_005673970.3,XM_001929569.6,XM_021068253.1,NM_214387.1,XM_021083741.1,XM_021073419.1,XM_021094599.1,XM_021095277.1,XM_021099431.1,XM_021094362.1,XM_021100455.1,XM_013993522.2 |
| GO:0019013 | viral nucleocapsid | 0.30517 | XM_021095277.1 |
| GO:0006520 | cellular amino acid metabolic process | 0.30528 | XM_021101430.1,XM_005666234.3,XM_021073780.1 |
| GO:0005179 | hormone activity | 0.30634 | NM_213872.1,XM_001926013.5 |
| GO:0004620 | phospholipase activity | 0.30694 | XM_013981430.2 |
| GO:0004970 | ionotropic glutamate receptor activity | 0.30938 | XM_005656504.3 |
| GO:0008066 | glutamate receptor activity | 0.30938 | XM_005656504.3 |
| GO:0032501 | multicellular organismal process | 0.31062 | XM_021101416.1,XM_013993522.2,XM_003132997.4,XM_021092351.1,XM_003132993.4,XM_021095277.1,NM_001243297.1 |
| GO:0005515 | protein binding | 0.31122 | XM_003358669.4,XM_021094362.1,XM_001926013.5,XM_021074380.1,XM_021100487.1,XM_021062982.1,XM_021083673.1,XM_021094599.1,XM_021083730.1,XM_021069802.1,NM_001285967.1,XM_021099082.1,XM_021074310.1,XM_021094366.1,XM_021063096.1,XM_021079051.1,XM_021077210.1,XM_005673970.3,XM_013988351.2,XM_005670911.3,XM_003126445.6,XM_021088208.1,XM_003132993.4,XM_021101431.1,XM_003484018.4,XM_021071138.1,NM_214435.2,NM_001243297.1,XM_021091087.1,XM_021102528.1,XM_021068253.1,XM_021083170.1,XM_021073445.1,XM_021094364.1,XM_021099431.1,XM_013981913.2,XM_003133545.5,NM_213872.1,XM_021072792.1,XM_003132997.4,XM_021076633.1,XM_021067261.1,XM_003132994.4,XM_021069763.1,NM_213734.1,XM_021091960.1,XM_021102607.1,XM_003125515.5,XM_021096440.1,XM_021090713.1,XM_021069816.1,XM_003133210.6,XM_013993593.2,NM_001105289.1,XM_021088199.1,XM_005664262.3,XM_013984188.2 |
| GO:0006935 | chemotaxis | 0.31213 | XM_005670922.3 |
| GO:0042330 | taxis | 0.31213 | XM_005670922.3 |
| GO:0000785 | chromatin | 0.31262 | XM_021095277.1 |
| GO:0005886 | plasma membrane | 0.3139 | XM_021074039.1,XM_021073780.1,NM_001285967.1,XM_021099431.1 |
| GO:0035556 | intracellular signal transduction | 0.31476 | XM_021099391.1,XM_021072792.1,XM_013983166.2,XM_021067261.1,XM_021062982.1,XM_021068991.1,XM_021101439.1,XM_005656513.3,XM_021099431.1 |
| GO:0060255 | regulation of macromolecule metabolic process | 0.31492 | XM_021083741.1,XM_021068253.1,NM_214387.1,XM_001929569.6,XM_021094599.1,XM_021095277.1,XM_021099431.1,XM_021073419.1,XM_021100455.1,XM_013993522.2,XM_021094362.1,XM_021083737.1,XM_021101423.1,XM_005656513.3,XM_021100671.1,XM_001928598.6,XM_001926951.4,XM_021094366.1,XM_005673970.3 |
| GO:0000075 | cell cycle checkpoint | 0.31492 | XM_021073419.1 |
| GO:0030955 | potassium ion binding | 0.31533 | XM_021071922.1 |
| GO:0031420 | alkali metal ion binding | 0.31533 | XM_021071922.1 |
| GO:0031329 | regulation of cellular catabolic process | 0.31616 | XM_005670922.3,XM_001924990.5 |
| GO:0003916 | DNA topoisomerase activity | 0.31819 | XM_021072739.1 |
| GO:0009288 | bacterial-type flagellum | 0.32079 | XM_005670922.3 |
| GO:0008408 | 3'-5' exonuclease activity | 0.32205 | XM_021091238.1 |
| GO:0031975 | envelope | 0.32259 | XM_021102607.1,NM_001315597.1,XM_021078736.1 |
| GO:0072330 | monocarboxylic acid biosynthetic process | 0.32298 | XM_021073419.1 |
| GO:0006796 | phosphate-containing compound metabolic process | 0.32544 | XM_021101533.1,XM_021091960.1,NM_214387.1,XM_021074310.1,XM_021099557.1,XM_021100595.1,XM_021096361.1,XM_013986853.2,XM_013986189.2,XM_021071922.1,XM_021082806.1,XM_021075700.1 |
| GO:0004721 | phosphoprotein phosphatase activity | 0.32576 | NM_214387.1,XM_021074310.1 |
| GO:0009894 | regulation of catabolic process | 0.32606 | XM_005670922.3,XM_001924990.5 |
| GO:0006793 | phosphorus metabolic process | 0.32724 | NM_214387.1,XM_021091960.1,XM_021101533.1,XM_021082806.1,XM_021071922.1,XM_021075700.1,XM_021096361.1,XM_021100595.1,XM_013986853.2,XM_013986189.2,XM_021099557.1,XM_021074310.1 |
| GO:0006470 | protein dephosphorylation | 0.32883 | NM_214387.1,XM_021074310.1 |
| GO:0015672 | monovalent inorganic cation transport | 0.33065 | XM_021071922.1,XM_021074028.1,XM_021094362.1,XM_021101542.1 |
| GO:0046873 | metal ion transmembrane transporter activity | 0.33121 | XM_021101542.1,XM_001927253.5,XM_021094362.1,XM_021071922.1,XM_021088199.1 |
| GO:0009615 | response to virus | 0.33537 | XM_001929445.5 |
| GO:0043933 | macromolecular complex subunit organization | 0.33685 | XM_005673970.3,XM_013981913.2,XM_003132993.4,XM_001929437.4,XM_021075699.1,XM_021070329.1 |
| GO:0044764 | multi-organism cellular process | 0.3369 | XM_021102528.1,XM_001929445.5,XM_001929569.6,XM_021096440.1,XM_021073780.1 |
| GO:0005342 | organic acid transmembrane transporter activity | 0.33716 | XM_013996640.2 |
| GO:0008514 | organic anion transmembrane transporter activity | 0.33716 | XM_013996640.2 |
| GO:0046943 | carboxylic acid transmembrane transporter activity | 0.33716 | XM_013996640.2 |
| GO:0016301 | kinase activity | 0.34027 | XM_021099557.1,XM_003358669.4,XM_021071922.1,XM_021075700.1,XM_021082806.1,XM_013986189.2,XM_021100595.1,XM_021096361.1,XM_021075702.1,XM_013986853.2,XM_021091960.1,XM_021101533.1 |
| GO:0006022 | aminoglycan metabolic process | 0.3454 | NM_001001260.1 |
| GO:0051028 | mRNA transport | 0.34682 | XM_005670922.3 |
| GO:0048037 | cofactor binding | 0.34818 | XM_001924990.5,NM_001167636.1,XM_001926818.5,XM_021074028.1 |
| GO:0007600 | sensory perception | 0.34852 | XM_021092351.1 |
| GO:0050877 | neurological system process | 0.34852 | XM_021092351.1 |
| GO:0044765 | single-organism transport | 0.35108 | XM_021094364.1,XM_001927253.5,XM_021091087.1,XM_021100487.1,XM_013989320.2,XM_013996640.2,XM_003126445.6,XM_021070329.1,XM_005670867.3,XM_021094362.1,XM_021071922.1,XM_021074028.1,XM_021088199.1,XM_021074039.1,XM_021102607.1,NM_001164021.1,XM_021101542.1,XM_021080387.1 |
| GO:1901564 | organonitrogen compound metabolic process | 0.35177 | XM_021100595.1,XM_021074028.1,XM_021101430.1,XM_021071138.1,XM_003359240.4,NM_001001260.1,XM_001924990.5,XM_005666234.3,NM_001167636.1,XM_021073780.1 |
| GO:0016765 | transferase activity, transferring alkyl or aryl (other than methyl) groups | 0.35209 | NM_214435.2 |
| GO:0006820 | anion transport | 0.35228 | XM_013989320.2,XM_013996640.2 |
| GO:0004842 | ubiquitin-protein transferase activity | 0.35272 | XM_021088208.1,XM_005670915.3,XM_021076633.1 |
| GO:0019787 | ubiquitin-like protein transferase activity | 0.35272 | XM_021088208.1,XM_021076633.1,XM_005670915.3 |
| GO:0044427 | chromosomal part | 0.35289 | XM_021095277.1,XM_005670867.3,XM_003132993.4 |
| GO:0033993 | response to lipid | 0.35406 | NM_214387.1 |
| GO:0043401 | steroid hormone mediated signaling pathway | 0.35406 | NM_214387.1 |
| GO:0048545 | response to steroid hormone | 0.35406 | NM_214387.1 |
| GO:0071383 | cellular response to steroid hormone stimulus | 0.35406 | NM_214387.1 |
| GO:0071396 | cellular response to lipid | 0.35406 | NM_214387.1 |
| GO:0071407 | cellular response to organic cyclic compound | 0.35406 | NM_214387.1 |
| GO:0006351 | transcription, DNA-templated | 0.35511 | XM_021095277.1,XM_021099431.1,XM_021094599.1,XM_021073419.1,XM_021083741.1,XM_021068253.1,NM_214387.1,XM_001929569.6,XM_013993522.2,XM_021100455.1,XM_021094362.1,XM_001928598.6,XM_021100671.1,XM_021083737.1,XM_005656513.3,XM_021101423.1,XM_005673970.3,XM_001926951.4,XM_021094366.1,XM_013983166.2 |
| GO:0097659 | nucleic acid-templated transcription | 0.35511 | XM_021094362.1,XM_013993522.2,XM_021100455.1,XM_021068253.1,NM_214387.1,XM_001929569.6,XM_021083741.1,XM_021073419.1,XM_021094599.1,XM_021095277.1,XM_021099431.1,XM_013983166.2,XM_021094366.1,XM_001926951.4,XM_005673970.3,XM_021101423.1,XM_005656513.3,XM_021083737.1,XM_021100671.1,XM_001928598.6 |
| GO:0016311 | dephosphorylation | 0.35707 | NM_214387.1,XM_021074310.1 |
| GO:0005634 | nucleus | 0.3598 | XM_001926951.4,XM_003132997.4,XM_001929437.4,XM_021101423.1,XM_021090713.1,XM_001928598.6,XM_021100671.1,NM_001315597.1,XM_021088208.1,XM_005670867.3,XM_003132993.4,XM_021094362.1,XM_021068253.1,XM_021099431.1,XM_021095277.1,XM_013981913.2,NM_001285967.1 |
| GO:0016298 | lipase activity | 0.35983 | XM_013981430.2 |
| GO:1901565 | organonitrogen compound catabolic process | 0.36003 | NM_001001260.1 |
| GO:0019199 | transmembrane receptor protein kinase activity | 0.36106 | XM_021075702.1 |
| GO:0008270 | zinc ion binding | 0.36252 | XM_021077210.1,XM_021094087.1,XM_001929445.5,XM_001926562.4,XM_013983166.2,XM_021088208.1,XM_021072792.1,XM_003358669.4,XM_013981913.2,XM_021099431.1,XM_021095277.1,XM_021078736.1,XM_013986501.2,XM_021072282.1,XM_021101423.1 |
| GO:0005507 | copper ion binding | 0.36471 | XM_001924990.5 |
| GO:0005198 | structural molecule activity | 0.36611 | NM_001243297.1,XM_021100487.1,XM_013989320.2,XM_021071138.1,XM_003132997.4,NM_001105289.1,XM_005656513.3,XM_021075699.1 |
| GO:0009617 | response to bacterium | 0.36618 | NM_001243297.1 |
| GO:0042742 | defense response to bacterium | 0.36618 | NM_001243297.1 |
| GO:0032774 | RNA biosynthetic process | 0.36683 | XM_013993522.2,XM_021100455.1,XM_021094362.1,XM_021094599.1,XM_021099431.1,XM_021095277.1,XM_021073419.1,XM_021083741.1,XM_021068253.1,XM_001929569.6,NM_214387.1,XM_005673970.3,XM_001926951.4,XM_013983166.2,XM_021094366.1,XM_021100671.1,XM_001928598.6,XM_021083737.1,XM_021101423.1,XM_005656513.3 |
| GO:0044237 | cellular metabolic process | 0.36873 | XM_021100595.1,XM_013986189.2,XM_021099557.1,XM_003132997.4,XM_001926951.4,XM_021101533.1,XM_021091960.1,XM_021083737.1,XM_001924990.5,XM_021100671.1,XM_021082806.1,XM_021075700.1,XM_021071922.1,XM_021074028.1,XM_021096361.1,XM_013993522.2,NM_001243297.1,XM_021071138.1,XM_021068253.1,XM_003359240.4,XM_021083741.1,XM_021073419.1,XM_013981913.2,XM_021099431.1,XM_005670922.3,XM_021079051.1,XM_021094366.1,XM_013986853.2,XM_013983166.2,XM_021074310.1,XM_021091183.1,XM_005673970.3,XM_021101430.1,XM_005656513.3,XM_021101423.1,XM_021073780.1,NM_001167636.1,XM_005666234.3,XM_001928598.6,XM_021094362.1,XM_003358669.4,XM_021100455.1,XM_021063404.1,XM_021094087.1,XM_021072739.1,XM_001929569.6,NM_214387.1,XM_001926818.5,XM_021095277.1,XM_021096140.1,XM_021094599.1,XM_005670915.3 |
| GO:0008289 | lipid binding | 0.36884 | XM_003130084.4,XM_013988351.2,XM_003121929.6 |
| GO:0016407 | acetyltransferase activity | 0.37354 | XM_021068991.1 |
| GO:0003676 | nucleic acid binding | 0.3772 | XM_021073419.1,XM_021099431.1,XM_021095277.1,XM_021091238.1,XM_021072739.1,XM_001929569.6,XM_003132998.4,XM_021102528.1,XM_021083741.1,XM_021101439.1,XM_021071138.1,XM_003130084.4,XM_001928339.4,XM_013993522.2,XM_021073780.1,XM_005656513.3,XM_021090713.1,XM_013988558.2,XM_021083737.1,XM_005673970.3,XM_021077210.1,XM_013999331.2,XM_001927718.4,XM_021094366.1,XM_021079051.1,XM_013983166.2,XM_021101542.1,XM_003132997.4,XM_021072792.1,XM_021091183.1 |
| GO:0006928 | movement of cell or subcellular component | 0.38052 | XM_021075699.1,XM_021102607.1,XM_005670922.3 |
| GO:0002064 | epithelial cell development | 0.38121 | XM_003132997.4 |
| GO:0002065 | columnar/cuboidal epithelial cell differentiation | 0.38121 | XM_003132997.4 |
| GO:0002066 | columnar/cuboidal epithelial cell development | 0.38121 | XM_003132997.4 |
| GO:0005213 | structural constituent of chorion | 0.38121 | XM_003132997.4 |
| GO:0007292 | female gamete generation | 0.38121 | XM_003132997.4 |
| GO:0007304 | chorion-containing eggshell formation | 0.38121 | XM_003132997.4 |
| GO:0030703 | eggshell formation | 0.38121 | XM_003132997.4 |
| GO:0030707 | ovarian follicle cell development | 0.38121 | XM_003132997.4 |
| GO:0048477 | oogenesis | 0.38121 | XM_003132997.4 |
| GO:0009755 | hormone-mediated signaling pathway | 0.38208 | NM_214387.1 |
| GO:0032870 | cellular response to hormone stimulus | 0.38208 | NM_214387.1 |
| GO:0044423 | virion part | 0.38303 | XM_005656513.3,NM_001285967.1,XM_013989320.2,XM_021095277.1,XM_005670922.3 |
| GO:0010256 | endomembrane system organization | 0.38475 | XM_005670922.3 |
| GO:0019048 | modulation by virus of host morphology or physiology | 0.38505 | XM_001929445.5 |
| GO:0009116 | nucleoside metabolic process | 0.38692 | XM_003359240.4,XM_021100595.1 |
| GO:0044428 | nuclear part | 0.39019 | NM_001315597.1,XM_021088208.1,XM_001929437.4,XM_005670867.3,XM_021094362.1,XM_003132993.4 |
| GO:0034654 | nucleobase-containing compound biosynthetic process | 0.39067 | XM_021083737.1,XM_021101423.1,XM_005656513.3,XM_021100671.1,XM_001928598.6,XM_001926951.4,XM_013983166.2,XM_021100595.1,XM_021094366.1,XM_005673970.3,XM_021083741.1,NM_214387.1,XM_021068253.1,XM_001929569.6,XM_021094599.1,XM_021095277.1,XM_021099431.1,XM_021073419.1,XM_021100455.1,XM_013993522.2,XM_021094362.1 |
| GO:1901657 | glycosyl compound metabolic process | 0.39144 | XM_021100595.1,XM_003359240.4 |
| GO:0071495 | cellular response to endogenous stimulus | 0.39315 | NM_214387.1 |
| GO:0030855 | epithelial cell differentiation | 0.3941 | XM_003132997.4 |
| GO:1902578 | single-organism localization | 0.39516 | XM_013989320.2,XM_021100487.1,XM_021091087.1,XM_021071922.1,XM_021074028.1,XM_021094362.1,XM_005670867.3,XM_013996640.2,XM_003126445.6,XM_021070329.1,XM_021094364.1,XM_001927253.5,NM_001164021.1,XM_021080387.1,XM_021101542.1,XM_021074039.1,XM_021088199.1,XM_021102607.1 |
| GO:0031981 | nuclear lumen | 0.39541 | XM_005670867.3,XM_001929437.4,XM_003132993.4,XM_021094362.1 |
| GO:0019028 | viral capsid | 0.39581 | NM_001285967.1,XM_005670922.3,XM_021095277.1,XM_005656513.3 |
| GO:0003674 | molecular_function | 0.4011 | XM_003132993.4,XM_021071922.1,XM_013993522.2,XM_013996640.2,XM_003126445.6,XM_001926857.5,XM_021088208.1,XM_021091087.1,XM_013981430.2,XM_021083170.1,XM_001927733.6,XM_003133545.5,XM_013981913.2,XM_013986189.2,XM_021091955.1,XM_021099557.1,XM_013999331.2,XM_021069763.1,XM_021102398.1,XM_005656504.3,XM_021090713.1,XM_021083737.1,XM_021102607.1,XM_021100671.1,XM_021094362.1,XM_003130084.4,XM_021085108.1,NM_214387.1,XM_001929569.6,XM_005670915.3,XM_021094599.1,XM_021091238.1,XM_013986501.2,XM_021095277.1,XM_013986853.2,XM_013983166.2,XM_021074050.1,XM_005656513.3,NM_001167636.1,XM_021074039.1,XM_001928598.6,XM_013988351.2,XM_001928339.4,XM_013989320.2,XM_021101431.1,XM_003484018.4,XM_021071138.1,NM_214435.2,XM_021068991.1,XM_003359240.4,XM_021102528.1,XM_021099431.1,XM_021100595.1,XM_001926562.4,XM_021072792.1,XM_003132994.4,XM_001927718.4,XM_021067261.1,XM_003133210.6,XM_021101533.1,XM_003125515.5,XM_005664262.3,XM_021065362.1,XM_013993593.2,NM_001105289.1,XM_005655662.3,XM_003132998.4,XM_021072282.1,XM_021072739.1,NM_001285967.1,XM_021083730.1,XM_021101542.1,NM_001164021.1,XM_021077210.1,XM_021101430.1,XM_021101423.1,XM_005670911.3,XM_003121929.6,XM_021096361.1,XM_021075700.1,XM_021082806.1,NM_001001260.1,XM_021073419.1,XM_005670922.3,XM_021078736.1,XM_021080387.1,XM_003132997.4,XM_021076633.1,XM_021069816.1,XM_013984188.2,XM_001924990.5,XM_003355124.2,XM_021100455.1,XM_021100487.1,XM_021063404.1,XM_021090817.1,XM_001927253.5,XM_021101439.1,XM_021069802.1,XM_021096140.1,XM_001929445.5,XM_021063096.1,XM_021079051.1,XM_021099082.1,XM_021091183.1,XM_005669930.3,XM_013988558.2,XM_005666234.3,XM_021074028.1,NM_001243297.1,XM_021092351.1,XM_021068253.1,XM_021073445.1,XM_021083741.1,NM_213872.1,XM_021094364.1,XM_021076863.1,XM_001926951.4,NM_213734.1,XM_021096440.1,XM_021091960.1,XM_021088199.1,XM_021075702.1,XM_003358669.4,XM_003135049.4,XM_021062982.1,XM_001926013.5,XM_021094087.1,XM_021074380.1,XM_001926818.5,XM_001928314.6,XM_021083673.1,XM_021094366.1,XM_021074310.1,XM_021101416.1,XM_005673970.3,XM_021075699.1,XM_021073780.1 |
| GO:0000775 | chromosome, centromeric region | 0.40129 | XM_003132993.4,XM_005670867.3 |
| GO:0003008 | system process | 0.4023 | XM_021092351.1 |
| GO:0004527 | exonuclease activity | 0.40334 | XM_001924990.5,XM_021091238.1 |
| GO:0017016 | Ras GTPase binding | 0.40678 | XM_021076633.1 |
| GO:0031267 | small GTPase binding | 0.40678 | XM_021076633.1 |
| GO:0051020 | GTPase binding | 0.40678 | XM_021076633.1 |
| GO:0070647 | protein modification by small protein conjugation or removal | 0.40754 | XM_021094087.1,XM_005670915.3 |
| GO:0031325 | positive regulation of cellular metabolic process | 0.40801 | XM_001924990.5,XM_005670922.3 |
| GO:0007281 | germ cell development | 0.40855 | XM_003132997.4 |
| GO:0042025 | host cell nucleus | 0.4129 | XM_021075699.1 |
| GO:0040007 | growth | 0.4146 | NM_214435.2 |
| GO:0016491 | oxidoreductase activity | 0.41513 | XM_021074028.1,XM_021091955.1,XM_013986853.2,XM_021074310.1,XM_021101430.1,XM_021072282.1,XM_001926818.5,NM_001167636.1,XM_001924990.5,XM_005666234.3 |
| GO:0031966 | mitochondrial membrane | 0.41585 | XM_021102607.1,XM_021078736.1 |
| GO:0043565 | sequence-specific DNA binding | 0.41802 | XM_005656513.3,XM_021090713.1,XM_013993522.2,XM_021073419.1,XM_021095277.1 |
| GO:0098687 | chromosomal region | 0.41977 | XM_003132993.4,XM_005670867.3 |
| GO:0019079 | viral genome replication | 0.42025 | XM_021102528.1 |
| GO:0005815 | microtubule organizing center | 0.42155 | XM_021075699.1 |
| GO:0098800 | inner mitochondrial membrane protein complex | 0.42187 | XM_021078736.1 |
| GO:0033647 | host intracellular organelle | 0.42301 | XM_021075699.1 |
| GO:0033648 | host intracellular membrane-bounded organelle | 0.42301 | XM_021075699.1 |
| GO:0071310 | cellular response to organic substance | 0.42477 | NM_214387.1 |
| GO:0043233 | organelle lumen | 0.42553 | XM_021094362.1,XM_003132993.4,XM_005670867.3,XM_001929437.4 |
| GO:0070013 | intracellular organelle lumen | 0.42553 | XM_001929437.4,XM_005670867.3,XM_003132993.4,XM_021094362.1 |
| GO:0044003 | modification by symbiont of host morphology or physiology | 0.42592 | XM_001929445.5 |
| GO:0009308 | amine metabolic process | 0.42791 | XM_001924990.5 |
| GO:0009893 | positive regulation of metabolic process | 0.42803 | XM_001924990.5,XM_005670922.3 |
| GO:0031974 | membrane-enclosed lumen | 0.42896 | XM_005670867.3,XM_001929437.4,XM_021094362.1,XM_003132993.4 |
| GO:0051817 | modification of morphology or physiology of other organism involved in symbiotic interaction | 0.4305 | XM_001929445.5 |
| GO:0050662 | coenzyme binding | 0.43408 | NM_001167636.1,XM_001926818.5,XM_021074028.1 |
| GO:0022412 | cellular process involved in reproduction in multicellular organism | 0.43508 | XM_003132997.4 |
| GO:0015078 | hydrogen ion transmembrane transporter activity | 0.43514 | XM_021080387.1,XM_021074310.1 |
| GO:0035821 | modification of morphology or physiology of other organism | 0.4373 | XM_001929445.5 |
| GO:0045333 | cellular respiration | 0.43738 | XM_001926818.5 |
| GO:0009725 | response to hormone | 0.44083 | NM_214387.1 |
| GO:0005740 | mitochondrial envelope | 0.44093 | XM_021102607.1,XM_021078736.1 |
| GO:0005741 | mitochondrial outer membrane | 0.44145 | XM_021102607.1 |
| GO:0031968 | organelle outer membrane | 0.44145 | XM_021102607.1 |
| GO:0015093 | ferrous iron transmembrane transporter activity | 0.44979 | XM_001927253.5 |
| GO:0015684 | ferrous iron transport | 0.44979 | XM_001927253.5 |
| GO:0031300 | intrinsic component of organelle membrane | 0.45316 | NM_001315597.1 |
| GO:0031301 | integral component of organelle membrane | 0.45316 | NM_001315597.1 |
| GO:0070887 | cellular response to chemical stimulus | 0.4537 | NM_214387.1 |
| GO:0016705 | oxidoreductase activity, acting on paired donors, with incorporation or reduction of molecular oxygen | 0.45414 | XM_021074028.1,XM_001926818.5 |
| GO:0005249 | voltage-gated potassium channel activity | 0.45856 | XM_021071922.1 |
| GO:0060429 | epithelium development | 0.45911 | XM_003132997.4 |
| GO:0033646 | host intracellular part | 0.45975 | XM_021075699.1 |
| GO:0043656 | intracellular region of host | 0.45975 | XM_021075699.1 |
| GO:0007005 | mitochondrion organization | 0.46179 | XM_021102607.1 |
| GO:0016810 | hydrolase activity, acting on carbon-nitrogen (but not peptide) bonds | 0.4624 | NM_001001260.1 |
| GO:0006826 | iron ion transport | 0.46932 | XM_001927253.5 |
| GO:0046483 | heterocycle metabolic process | 0.46943 | NM_001167636.1,XM_021073780.1,XM_001928598.6,XM_005666234.3,XM_021101423.1,XM_005656513.3,XM_005673970.3,XM_021101430.1,XM_013983166.2,XM_021079051.1,XM_021094366.1,XM_021094599.1,XM_021095277.1,XM_021096140.1,XM_001929569.6,XM_021072739.1,NM_214387.1,XM_021094362.1,XM_003358669.4,XM_021100455.1,XM_021100671.1,XM_021083737.1,XM_021100595.1,XM_001926951.4,XM_003132997.4,XM_021073419.1,XM_021099431.1,XM_021068253.1,XM_021083741.1,XM_003359240.4,XM_021074028.1,XM_013993522.2 |
| GO:0006725 | cellular aromatic compound metabolic process | 0.47068 | XM_021083737.1,XM_021100671.1,XM_001926951.4,XM_003132997.4,XM_021100595.1,XM_003359240.4,XM_021083741.1,XM_021068253.1,XM_021099431.1,XM_021073419.1,XM_013993522.2,XM_021074028.1,XM_021101423.1,XM_005656513.3,XM_001928598.6,XM_005666234.3,NM_001167636.1,XM_021073780.1,XM_013983166.2,XM_021079051.1,XM_021094366.1,XM_021101430.1,XM_005673970.3,XM_021072739.1,XM_001929569.6,NM_214387.1,XM_021094599.1,XM_021096140.1,XM_021095277.1,XM_021100455.1,XM_003358669.4,XM_021094362.1 |
| GO:0005381 | iron ion transmembrane transporter activity | 0.47283 | XM_001927253.5 |
| GO:0016043 | cellular component organization | 0.47376 | XM_005670867.3,XM_021070329.1,XM_003132993.4,XM_005673970.3,XM_001929437.4,XM_021102607.1,XM_021075699.1,XM_021072739.1,XM_021099431.1,XM_021100671.1,XM_005670922.3,NM_001285967.1,XM_013981913.2 |
| GO:0005615 | extracellular space | 0.4762 | XM_003132993.4,XM_003358668.4 |
| GO:0048731 | system development | 0.47757 | XM_013993522.2 |
| GO:0071944 | cell periphery | 0.47777 | XM_021099431.1,NM_001285967.1,XM_021074039.1,XM_021073780.1,XM_003132997.4 |
| GO:0015980 | energy derivation by oxidation of organic compounds | 0.47879 | XM_001926818.5 |
| GO:0007264 | small GTPase mediated signal transduction | 0.47936 | XM_021062982.1,XM_021099431.1,XM_021068991.1,XM_021101439.1 |
| GO:0044429 | mitochondrial part | 0.48031 | XM_021078736.1,XM_021102607.1 |
| GO:0019898 | extrinsic component of membrane | 0.48033 | XM_021102607.1 |
| GO:0015711 | organic anion transport | 0.48256 | XM_013996640.2 |
| GO:0015849 | organic acid transport | 0.48256 | XM_013996640.2 |
| GO:0046942 | carboxylic acid transport | 0.48256 | XM_013996640.2 |
| GO:0015291 | secondary active transmembrane transporter activity | 0.48297 | XM_021080387.1 |
| GO:0042600 | chorion | 0.4839 | XM_003132997.4 |
| GO:0001071 | nucleic acid binding transcription factor activity | 0.49292 | XM_021073419.1,XM_021100671.1,XM_001928598.6,XM_021095277.1,XM_005656513.3,XM_021100455.1,XM_001926951.4 |
| GO:0003700 | transcription factor activity, sequence-specific DNA binding | 0.49292 | XM_005656513.3,XM_001926951.4,XM_021100455.1,XM_021073419.1,XM_021095277.1,XM_001928598.6,XM_021100671.1 |
| GO:0016835 | carbon-oxygen lyase activity | 0.49313 | XM_003121929.6 |
| GO:0006310 | DNA recombination | 0.4942 | XM_013993522.2,XM_003132997.4 |
| GO:0003006 | developmental process involved in reproduction | 0.49646 | XM_003132997.4 |
| GO:0016567 | protein ubiquitination | 0.49758 | XM_005670915.3 |
| GO:0016579 | protein deubiquitination | 0.49979 | XM_021094087.1 |
| GO:0070646 | protein modification by small protein removal | 0.49979 | XM_021094087.1 |
| GO:0042451 | purine nucleoside biosynthetic process | 0.5013 | XM_021100595.1 |
| GO:0046129 | purine ribonucleoside biosynthetic process | 0.5013 | XM_021100595.1 |
| GO:0051287 | NAD binding | 0.5019 | XM_021074028.1 |
| GO:1901360 | organic cyclic compound metabolic process | 0.50304 | XM_021073419.1,XM_021099431.1,XM_021068253.1,XM_003359240.4,XM_021083741.1,XM_021074028.1,XM_013993522.2,XM_021100671.1,XM_021083737.1,XM_021100595.1,XM_001926951.4,XM_003132997.4,XM_021094599.1,XM_021096140.1,XM_021095277.1,XM_001929569.6,XM_021072739.1,NM_214387.1,XM_021094362.1,XM_003358669.4,XM_021100455.1,NM_001167636.1,XM_021073780.1,XM_001928598.6,XM_005666234.3,XM_021101423.1,XM_005656513.3,XM_005673970.3,XM_021101430.1,XM_013983166.2,XM_021079051.1,XM_021094366.1 |
| GO:0044403 | symbiosis, encompassing mutualism through parasitism | 0.50649 | XM_001929445.5,XM_021096440.1,XM_021102528.1,XM_021076633.1 |
| GO:0044419 | interspecies interaction between organisms | 0.50753 | XM_021102528.1,XM_001929445.5,XM_021096440.1,XM_021076633.1 |
| GO:0002376 | immune system process | 0.50825 | XM_001929445.5,XM_013993522.2 |
| GO:0032446 | protein modification by small protein conjugation | 0.50934 | XM_005670915.3 |
| GO:0043038 | amino acid activation | 0.51291 | XM_021073780.1 |
| GO:0043039 | tRNA aminoacylation | 0.51291 | XM_021073780.1 |
| GO:0022804 | active transmembrane transporter activity | 0.51293 | XM_001928314.6,XM_021080387.1 |
| GO:0043093 | FtsZ-dependent cytokinesis | 0.51428 | XM_021099431.1 |
| GO:0036459 | ubiquitinyl hydrolase activity | 0.51459 | XM_021094087.1 |
| GO:0046914 | transition metal ion binding | 0.5186 | XM_021077210.1,XM_021072792.1,XM_001929445.5,XM_013983166.2,XM_001926562.4,XM_001924990.5,XM_021101423.1,XM_021094087.1,XM_021088208.1,XM_003358669.4,XM_021099431.1,XM_021095277.1,XM_021078736.1,XM_013986501.2,XM_013981913.2,XM_021072282.1 |
| GO:0016875 | ligase activity, forming carbon-oxygen bonds | 0.51953 | XM_021073780.1 |
| GO:0016876 | ligase activity, forming aminoacyl-tRNA and related compounds | 0.51953 | XM_021073780.1 |
| GO:0016070 | RNA metabolic process | 0.52005 | XM_021100455.1,XM_013993522.2,XM_021074028.1,XM_021094362.1,XM_021099431.1,XM_021095277.1,XM_021094599.1,XM_021073419.1,XM_021083741.1,XM_001929569.6,XM_021068253.1,NM_214387.1,XM_005673970.3,XM_001926951.4,XM_021094366.1,XM_021079051.1,XM_013983166.2,XM_021100671.1,XM_001928598.6,XM_021073780.1,XM_021083737.1,XM_005656513.3,XM_021101423.1 |
| GO:0032505 | reproduction of a single-celled organism | 0.52303 | XM_021099431.1 |
| GO:0006457 | protein folding | 0.52303 | XM_005670915.3 |
| GO:0009888 | tissue development | 0.52426 | XM_003132997.4 |
| GO:0033643 | host cell part | 0.52553 | XM_021075699.1 |
| GO:0098662 | inorganic cation transmembrane transport | 0.52739 | XM_021101542.1,XM_021094362.1 |
| GO:0044267 | cellular protein metabolic process | 0.52917 | XM_021071138.1,XM_021094087.1,XM_021082806.1,XM_021071922.1,XM_021075700.1,XM_021096361.1,XM_003358669.4,XM_013981913.2,XM_005670915.3,NM_214387.1,XM_005673970.3,XM_013986853.2,XM_013986189.2,XM_021099557.1,XM_021074310.1,XM_021091960.1,XM_021101533.1 |
| GO:0098660 | inorganic ion transmembrane transport | 0.53004 | XM_021094362.1,XM_021101542.1 |
| GO:0048468 | cell development | 0.53235 | XM_003132997.4 |
| GO:0000917 | barrier septum assembly | 0.53238 | XM_021099431.1 |
| GO:0032506 | cytokinetic process | 0.53238 | XM_021099431.1 |
| GO:0090529 | cell septum assembly | 0.53238 | XM_021099431.1 |
| GO:1902410 | mitotic cytokinetic process | 0.53238 | XM_021099431.1 |
| GO:0005261 | cation channel activity | 0.53239 | XM_021071922.1,XM_021074039.1 |
| GO:0044249 | cellular biosynthetic process | 0.53302 | XM_021083737.1,XM_005656513.3,XM_021101423.1,XM_005666234.3,XM_001928598.6,XM_021100671.1,NM_001167636.1,XM_001926951.4,XM_021094366.1,XM_021100595.1,XM_013983166.2,XM_021101430.1,XM_005673970.3,XM_021083741.1,XM_001926818.5,XM_021068253.1,NM_214387.1,XM_001929569.6,XM_021095277.1,XM_021099431.1,XM_021094599.1,XM_021073419.1,XM_021100455.1,XM_013993522.2,XM_021074028.1,XM_021094362.1,XM_021071138.1,XM_021063404.1 |
| GO:0009163 | nucleoside biosynthetic process | 0.53537 | XM_021100595.1 |
| GO:0042455 | ribonucleoside biosynthetic process | 0.53537 | XM_021100595.1 |
| GO:0008083 | growth factor activity | 0.53548 | NM_214435.2 |
| GO:0018995 | host | 0.53865 | XM_021075699.1 |
| GO:0043657 | host cell | 0.53865 | XM_021075699.1 |
| GO:0044215 | other organism | 0.53865 | XM_021075699.1 |
| GO:0044216 | other organism cell | 0.53865 | XM_021075699.1 |
| GO:0044217 | other organism part | 0.53865 | XM_021075699.1 |
| GO:1901659 | glycosyl compound biosynthetic process | 0.54131 | XM_021100595.1 |
| GO:0048646 | anatomical structure formation involved in morphogenesis | 0.54456 | XM_003132997.4 |
| GO:0044877 | macromolecular complex binding | 0.54744 | XM_021095277.1,XM_021088199.1,XM_021074380.1 |
| GO:0005743 | mitochondrial inner membrane | 0.54872 | XM_021078736.1 |
| GO:0019954 | asexual reproduction | 0.54977 | XM_021099431.1 |
| GO:0000281 | mitotic cytokinesis | 0.55371 | XM_021099431.1 |
| GO:0061640 | cytoskeleton-dependent cytokinesis | 0.55371 | XM_021099431.1 |
| GO:0003724 | RNA helicase activity | 0.55515 | XM_021101542.1 |
| GO:0006807 | nitrogen compound metabolic process | 0.55625 | XM_005656513.3,XM_021101423.1,XM_021073780.1,NM_001167636.1,XM_005666234.3,XM_001928598.6,XM_021094366.1,XM_021079051.1,XM_013983166.2,XM_005673970.3,XM_021101430.1,NM_214387.1,XM_001929569.6,XM_021072739.1,XM_021096140.1,XM_021095277.1,XM_021094599.1,XM_021094362.1,XM_021100455.1,XM_003358669.4,XM_021083737.1,XM_001924990.5,XM_021100671.1,XM_021100595.1,XM_003132997.4,XM_001926951.4,XM_021068253.1,NM_001001260.1,XM_003359240.4,XM_021083741.1,XM_021073419.1,XM_021099431.1,XM_021074028.1,XM_013993522.2,XM_021071138.1 |
| GO:0005216 | ion channel activity | 0.55927 | XM_021100487.1,XM_021074039.1,XM_005656504.3,XM_021071922.1 |
| GO:0022838 | substrate-specific channel activity | 0.55927 | XM_021071922.1,XM_005656504.3,XM_021074039.1,XM_021100487.1 |
| GO:0019538 | protein metabolic process | 0.56207 | XM_021075700.1,XM_021082806.1,XM_021071922.1,XM_021096361.1,XM_003358669.4,XM_021071138.1,XM_021094087.1,NM_214387.1,XM_013981913.2,XM_021083730.1,XM_021078736.1,XM_005670915.3,XM_001929445.5,XM_013986853.2,XM_013986189.2,XM_001926562.4,XM_021099557.1,XM_021074310.1,XM_005673970.3,XM_021101423.1,XM_021101533.1,XM_021091960.1 |
| GO:0071103 | DNA conformation change | 0.56963 | XM_021072739.1 |
| GO:0005096 | GTPase activator activity | 0.57047 | XM_021099431.1 |
| GO:1901576 | organic substance biosynthetic process | 0.57124 | NM_001167636.1,XM_005666234.3,XM_001928598.6,XM_021100671.1,XM_005656513.3,XM_021101423.1,XM_021083737.1,XM_005673970.3,XM_021101430.1,XM_021094366.1,XM_013983166.2,XM_021100595.1,XM_001926951.4,XM_021073419.1,XM_021099431.1,XM_021095277.1,XM_021094599.1,XM_021068253.1,XM_001929569.6,NM_214387.1,XM_021083741.1,XM_001926818.5,XM_021063404.1,XM_021071138.1,XM_021074028.1,XM_021094362.1,XM_013993522.2,XM_021100455.1 |
| GO:0006810 | transport | 0.57199 | XM_021074039.1,XM_021088199.1,XM_021102607.1,XM_021075699.1,NM_001164021.1,XM_021080387.1,XM_021101542.1,XM_021094364.1,XM_005670922.3,XM_021099431.1,XM_001927253.5,XM_021091087.1,XM_021100487.1,XM_013989320.2,XM_021094362.1,XM_021071922.1,XM_021074028.1,XM_003126445.6,XM_021070329.1,XM_013996640.2,XM_005670867.3 |
| GO:0051726 | regulation of cell cycle | 0.57235 | NM_001285967.1 |
| GO:0071840 | cellular component organization or biogenesis | 0.5734 | XM_003132993.4,XM_021070329.1,XM_005670867.3,XM_005673970.3,XM_021072739.1,XM_021075699.1,XM_021102607.1,XM_001929437.4,XM_013981913.2,NM_001285967.1,XM_021100671.1,XM_005670922.3,XM_021099431.1 |
| GO:0015267 | channel activity | 0.57424 | XM_021071922.1,XM_021074039.1,XM_021100487.1,XM_005656504.3 |
| GO:0022803 | passive transmembrane transporter activity | 0.57424 | XM_021071922.1,XM_005656504.3,XM_021100487.1,XM_021074039.1 |
| GO:0006914 | autophagy | 0.5761 | XM_005670922.3,XM_001924990.5 |
| GO:0005577 | fibrinogen complex | 0.58415 | XM_003132993.4 |
| GO:0030168 | platelet activation | 0.58415 | XM_003132993.4 |
| GO:0034641 | cellular nitrogen compound metabolic process | 0.58471 | XM_001926951.4,XM_003132997.4,XM_021100595.1,XM_021083737.1,XM_021100671.1,XM_013993522.2,XM_021074028.1,XM_021071138.1,XM_021083741.1,XM_003359240.4,XM_021068253.1,XM_021099431.1,XM_021073419.1,XM_013983166.2,XM_021094366.1,XM_021079051.1,XM_021101430.1,XM_005673970.3,XM_021101423.1,XM_005656513.3,XM_001928598.6,XM_005666234.3,NM_001167636.1,XM_021073780.1,XM_003358669.4,XM_021100455.1,XM_021094362.1,XM_001929569.6,NM_214387.1,XM_021072739.1,XM_021094599.1,XM_021095277.1,XM_021096140.1 |
| GO:0004190 | aspartic-type endopeptidase activity | 0.58654 | XM_003484018.4 |
| GO:0070001 | aspartic-type peptidase activity | 0.58654 | XM_003484018.4 |
| GO:0001076 | transcription factor activity, RNA polymerase II transcription factor binding | 0.58769 | XM_021094362.1 |
| GO:0001104 | RNA polymerase II transcription cofactor activity | 0.58769 | XM_021094362.1 |
| GO:0051234 | establishment of localization | 0.58792 | XM_021075699.1,XM_021102607.1,XM_021074039.1,XM_021088199.1,XM_021080387.1,XM_021101542.1,NM_001164021.1,XM_001927253.5,XM_021099431.1,XM_005670922.3,XM_021094364.1,XM_021074028.1,XM_021071922.1,XM_021094362.1,XM_005670867.3,XM_021070329.1,XM_003126445.6,XM_013996640.2,XM_013989320.2,XM_021100487.1,XM_021091087.1 |
| GO:0005267 | potassium channel activity | 0.58852 | XM_021071922.1 |
| GO:0022843 | voltage-gated cation channel activity | 0.59229 | XM_021071922.1 |
| GO:0004674 | protein serine/threonine kinase activity | 0.59339 | XM_013986189.2,XM_021075702.1 |
| GO:1901566 | organonitrogen compound biosynthetic process | 0.59391 | XM_021101430.1,XM_021071138.1,XM_005666234.3,NM_001167636.1,XM_021100595.1,XM_021074028.1 |
| GO:0016592 | mediator complex | 0.5945 | XM_021094362.1 |
| GO:0070838 | divalent metal ion transport | 0.59748 | XM_001927253.5 |
| GO:0048522 | positive regulation of cellular process | 0.5986 | XM_001924990.5,XM_005670922.3 |
| GO:0030695 | GTPase regulator activity | 0.60233 | XM_021099431.1 |
| GO:0008138 | protein tyrosine/serine/threonine phosphatase activity | 0.6061 | XM_021074310.1 |
| GO:0000910 | cytokinesis | 0.60734 | XM_021099431.1 |
| GO:0015079 | potassium ion transmembrane transporter activity | 0.61009 | XM_021071922.1 |
| GO:0008855 | exodeoxyribonuclease VII activity | 0.61168 | XM_001924990.5 |
| GO:0044238 | primary metabolic process | 0.61276 | XM_021101430.1,XM_005673970.3,XM_021074310.1,XM_013983166.2,XM_013986853.2,XM_021094366.1,XM_001929445.5,XM_021079051.1,XM_001928598.6,XM_005666234.3,NM_001167636.1,XM_021073780.1,XM_021101423.1,XM_005656513.3,XM_021094087.1,XM_021063404.1,XM_003358669.4,XM_021100455.1,XM_021094362.1,XM_021094599.1,XM_005670915.3,XM_021095277.1,XM_021096140.1,XM_021083730.1,XM_001926818.5,XM_001929569.6,NM_214387.1,XM_021072739.1,XM_001926951.4,XM_021099557.1,XM_003132997.4,XM_001926562.4,XM_021100595.1,XM_013986189.2,XM_021100671.1,XM_021083737.1,XM_021091960.1,XM_021101533.1,XM_021071138.1,XM_013993522.2,XM_021096361.1,XM_021082806.1,XM_021071922.1,XM_021075700.1,XM_021074028.1,XM_021099431.1,XM_021078736.1,XM_013981913.2,XM_021073419.1,XM_021083741.1,XM_003359240.4,XM_021068253.1 |
| GO:0009152 | purine ribonucleotide biosynthetic process | 0.61342 | XM_021100595.1 |
| GO:0009260 | ribonucleotide biosynthetic process | 0.61342 | XM_021100595.1 |
| GO:0046390 | ribose phosphate biosynthetic process | 0.61342 | XM_021100595.1 |
| GO:0003677 | DNA binding | 0.61764 | XM_021077210.1,XM_005673970.3,XM_021072792.1,XM_003132997.4,XM_013983166.2,XM_021073780.1,XM_021090713.1,XM_013988558.2,XM_005656513.3,XM_021071138.1,XM_013993522.2,XM_021091238.1,XM_021095277.1,XM_021099431.1,XM_021073419.1,XM_021101439.1,XM_021072739.1,XM_001929569.6 |
| GO:0006139 | nucleobase-containing compound metabolic process | 0.61861 | XM_021101423.1,XM_005656513.3,XM_021083737.1,XM_021073780.1,XM_021100671.1,XM_001928598.6,XM_013983166.2,XM_021100595.1,XM_021094366.1,XM_021079051.1,XM_001926951.4,XM_003132997.4,XM_005673970.3,XM_021072739.1,XM_021068253.1,XM_001929569.6,NM_214387.1,XM_021083741.1,XM_003359240.4,XM_021073419.1,XM_021094599.1,XM_021096140.1,XM_021099431.1,XM_021095277.1,XM_021094362.1,XM_021074028.1,XM_021100455.1,XM_013993522.2,XM_003358669.4 |
| GO:0044710 | single-organism metabolic process | 0.61903 | XM_003358669.4,XM_021074028.1,XM_021063404.1,XM_001926818.5,XM_003359240.4,XM_021072282.1,XM_013981913.2,XM_021073419.1,XM_021074310.1,XM_021062686.1,XM_021100595.1,XM_013986853.2,XM_013983166.2,XM_021091955.1,XM_021101430.1,XM_005673970.3,XM_005656513.3,XM_001924990.5,XM_005666234.3,NM_001167636.1,XM_021073780.1 |
| GO:0055086 | nucleobase-containing small molecule metabolic process | 0.62052 | XM_021100595.1,XM_003359240.4 |
| GO:0004529 | exodeoxyribonuclease activity | 0.62153 | XM_001924990.5 |
| GO:0016895 | exodeoxyribonuclease activity, producing 5'-phosphomonoesters | 0.62153 | XM_001924990.5 |
| GO:0001775 | cell activation | 0.6219 | XM_003132993.4 |
| GO:0071704 | organic substance metabolic process | 0.62194 | XM_021082806.1,XM_021071922.1,XM_021075700.1,XM_021074028.1,XM_021096361.1,XM_013993522.2,XM_021071138.1,XM_021068253.1,NM_001001260.1,XM_003359240.4,XM_021083741.1,XM_021073419.1,XM_013981913.2,XM_021099431.1,XM_021078736.1,XM_021100595.1,XM_001926562.4,XM_013986189.2,XM_021099557.1,XM_003132997.4,XM_001926951.4,XM_021091960.1,XM_021101533.1,XM_021083737.1,XM_001924990.5,XM_021100671.1,XM_021094362.1,XM_003358669.4,XM_021100455.1,XM_021063404.1,XM_021094087.1,NM_214387.1,XM_001929569.6,XM_021072739.1,XM_001926818.5,XM_021095277.1,XM_021096140.1,XM_021083730.1,XM_021094599.1,XM_005670915.3,XM_001929445.5,XM_021094366.1,XM_021079051.1,XM_013983166.2,XM_013986853.2,XM_021074310.1,XM_005673970.3,XM_021101430.1,XM_005656513.3,XM_021101423.1,XM_021073780.1,NM_001167636.1,XM_005666234.3,XM_001928598.6 |
| GO:0008238 | exopeptidase activity | 0.62319 | XM_021078736.1 |
| GO:0006164 | purine nucleotide biosynthetic process | 0.62351 | XM_021100595.1 |
| GO:0030312 | external encapsulating structure | 0.62578 | XM_003132997.4 |
| GO:0046915 | transition metal ion transmembrane transporter activity | 0.62578 | XM_001927253.5 |
| GO:0008047 | enzyme activator activity | 0.62662 | XM_021099431.1 |
| GO:0009058 | biosynthetic process | 0.62694 | XM_021094366.1,XM_013983166.2,XM_021100595.1,XM_001926951.4,XM_005673970.3,XM_021101430.1,XM_005656513.3,XM_021101423.1,XM_021083737.1,NM_001167636.1,XM_005666234.3,XM_021100671.1,XM_001928598.6,XM_021074028.1,XM_021094362.1,XM_021100455.1,XM_013993522.2,XM_021063404.1,XM_021071138.1,XM_001929569.6,XM_021068253.1,NM_214387.1,XM_021083741.1,XM_001926818.5,XM_021073419.1,XM_021095277.1,XM_021099431.1,XM_021094599.1 |
| GO:0007596 | blood coagulation | 0.63238 | XM_003132993.4 |
| GO:0043228 | non-membrane-bounded organelle | 0.63396 | XM_021095277.1,XM_005670922.3,XM_001929437.4,XM_021075699.1,XM_021071138.1,XM_005673970.3,XM_005670867.3,XM_003132993.4 |
| GO:0048856 | anatomical structure development | 0.6341 | XM_013993522.2,XM_003132997.4 |
| GO:0042626 | ATPase activity, coupled to transmembrane movement of substances | 0.63438 | XM_001928314.6 |
| GO:0043492 | ATPase activity, coupled to movement of substances | 0.63438 | XM_001928314.6 |
| GO:0005739 | mitochondrion | 0.63611 | XM_021078736.1,XM_021102607.1 |
| GO:0015399 | primary active transmembrane transporter activity | 0.63636 | XM_001928314.6 |
| GO:0015405 | P-P-bond-hydrolysis-driven transmembrane transporter activity | 0.63636 | XM_001928314.6 |
| GO:0044260 | cellular macromolecule metabolic process | 0.63741 | XM_005673970.3,XM_021074310.1,XM_021079051.1,XM_021094366.1,XM_013983166.2,XM_013986853.2,XM_001928598.6,XM_021073780.1,XM_005656513.3,XM_021101423.1,XM_021094087.1,XM_021100455.1,XM_003358669.4,XM_021094362.1,XM_021095277.1,XM_021094599.1,XM_005670915.3,XM_021072739.1,XM_001929569.6,NM_214387.1,XM_021099557.1,XM_003132997.4,XM_001926951.4,XM_013986189.2,XM_021100671.1,XM_021101533.1,XM_021091960.1,XM_021083737.1,XM_021071138.1,XM_013993522.2,XM_021082806.1,XM_021074028.1,XM_021075700.1,XM_021071922.1,XM_021096361.1,XM_021099431.1,XM_021073419.1,XM_013981913.2,XM_021083741.1,XM_021068253.1 |
| GO:0030674 | protein binding, bridging | 0.63746 | XM_003132993.4 |
| GO:0060090 | binding, bridging | 0.63746 | XM_003132993.4 |
| GO:0051701 | interaction with host | 0.64002 | XM_001929445.5 |
| GO:0006915 | apoptotic process | 0.64137 | NM_001112688.1 |
| GO:0012501 | programmed cell death | 0.64137 | NM_001112688.1 |
| GO:0072522 | purine-containing compound biosynthetic process | 0.64191 | XM_021100595.1 |
| GO:0009405 | pathogenesis | 0.64345 | XM_013993522.2,XM_021100487.1 |
| GO:0072511 | divalent inorganic cation transport | 0.64346 | XM_001927253.5 |
| GO:0009987 | cellular process | 0.64373 | XM_021073780.1,XM_021101423.1,XM_021075699.1,XM_001929437.4,XM_005673970.3,NM_001164021.1,XM_021101430.1,XM_021094366.1,XM_021101542.1,XM_021074310.1,NM_001285967.1,XM_021083673.1,XM_021072739.1,XM_001926818.5,XM_021062982.1,XM_021094087.1,XM_021070329.1,XM_003358669.4,XM_021088199.1,XM_021096440.1,XM_021101533.1,XM_021091960.1,XM_021067261.1,XM_021076863.1,XM_021100595.1,XM_021072792.1,XM_001926951.4,XM_021099431.1,XM_021094364.1,XM_021068253.1,XM_021073445.1,XM_021092351.1,XM_021102528.1,XM_003359240.4,XM_021083741.1,XM_021068991.1,NM_001112688.1,NM_001243297.1,XM_021071138.1,XM_021074028.1,XM_021074039.1,NM_001167636.1,XM_005666234.3,XM_001928598.6,XM_005656513.3,XM_021079051.1,XM_001929445.5,XM_013983166.2,XM_013986853.2,XM_021091183.1,XM_021096140.1,XM_021095277.1,XM_005670915.3,XM_021094599.1,NM_214387.1,XM_001929569.6,XM_021101439.1,XM_021090817.1,XM_021063404.1,XM_021085108.1,XM_003355124.2,XM_021094362.1,XM_021100455.1,XM_001924990.5,XM_021100671.1,XM_021102607.1,XM_021083737.1,XM_021080387.1,XM_013986189.2,XM_003132997.4,XM_021099557.1,XM_021073419.1,XM_013981913.2,XM_005670922.3,XM_001927733.6,XM_021091087.1,XM_021082806.1,XM_021075700.1,XM_021071922.1,XM_003132993.4,XM_021096361.1,XM_005670867.3,XM_013996640.2,XM_021099391.1,XM_013993522.2,XM_003126445.6 |
| GO:0005244 | voltage-gated ion channel activity | 0.64414 | XM_021071922.1 |
| GO:0022832 | voltage-gated channel activity | 0.64414 | XM_021071922.1 |
| GO:0006952 | defense response | 0.64639 | XM_001929445.5,NM_001243297.1 |
| GO:0016747 | transferase activity, transferring acyl groups other than amino-acyl groups | 0.6475 | XM_021068991.1 |
| GO:0006352 | DNA-templated transcription, initiation | 0.64778 | XM_005673970.3 |
| GO:0006357 | regulation of transcription from RNA polymerase II promoter | 0.65011 | XM_021094362.1 |
| GO:0098772 | molecular function regulator | 0.65102 | XM_021101439.1,XM_021068991.1,XM_021074039.1,XM_021062982.1,XM_021099431.1 |
| GO:0097159 | organic cyclic compound binding | 0.65134 | XM_001929569.6,XM_021072739.1,XM_001927253.5,XM_003132998.4,XM_001926818.5,XM_021101439.1,XM_021095277.1,XM_021091238.1,XM_003130084.4,XM_005656513.3,XM_013988558.2,XM_021073780.1,XM_021094366.1,XM_021079051.1,XM_013986853.2,XM_013983166.2,XM_021101542.1,XM_021091183.1,XM_021077210.1,XM_005673970.3,XM_021102528.1,XM_021083741.1,XM_021073419.1,XM_021099431.1,XM_021082806.1,XM_021071922.1,XM_021075700.1,XM_021074028.1,XM_021096361.1,XM_013993522.2,XM_001928339.4,XM_021071138.1,XM_021090713.1,XM_021091960.1,XM_021101533.1,XM_021083737.1,XM_021100595.1,XM_013986189.2,XM_003132997.4,XM_021099557.1,XM_021072792.1,XM_013999331.2,XM_001927718.4 |
| GO:1901363 | heterocyclic compound binding | 0.65134 | XM_021102528.1,XM_021083741.1,XM_021073419.1,XM_021099431.1,XM_021075700.1,XM_021082806.1,XM_021074028.1,XM_021071922.1,XM_021096361.1,XM_001928339.4,XM_013993522.2,XM_021071138.1,XM_021090713.1,XM_021101533.1,XM_021091960.1,XM_021083737.1,XM_013986189.2,XM_021100595.1,XM_003132997.4,XM_021099557.1,XM_021072792.1,XM_013999331.2,XM_001927718.4,XM_021072739.1,XM_001929569.6,XM_001927253.5,XM_003132998.4,XM_001926818.5,XM_021101439.1,XM_021095277.1,XM_021091238.1,XM_003130084.4,XM_005656513.3,XM_013988558.2,XM_021073780.1,XM_021079051.1,XM_021094366.1,XM_013983166.2,XM_013986853.2,XM_021101542.1,XM_021091183.1,XM_005673970.3,XM_021077210.1 |
| GO:0007599 | hemostasis | 0.65658 | XM_003132993.4 |
| GO:0050817 | coagulation | 0.65658 | XM_003132993.4 |
| GO:0050878 | regulation of body fluid levels | 0.65658 | XM_003132993.4 |
| GO:0048518 | positive regulation of biological process | 0.65729 | XM_005670922.3,XM_001924990.5 |
| GO:0043170 | macromolecule metabolic process | 0.65888 | XM_021100671.1,XM_021083737.1,XM_021101533.1,XM_021091960.1,XM_013986189.2,XM_001926562.4,XM_001926951.4,XM_003132997.4,XM_021099557.1,XM_013981913.2,XM_021073419.1,XM_021099431.1,XM_021078736.1,NM_001001260.1,XM_021068253.1,XM_021083741.1,XM_021071138.1,XM_021096361.1,XM_021071922.1,XM_021082806.1,XM_021075700.1,XM_021074028.1,XM_013993522.2,XM_021073780.1,XM_001928598.6,XM_021101423.1,XM_005656513.3,XM_005673970.3,XM_013983166.2,XM_013986853.2,XM_001929445.5,XM_021094366.1,XM_021079051.1,XM_021074310.1,XM_021094599.1,XM_005670915.3,XM_021095277.1,XM_021083730.1,XM_021072739.1,NM_214387.1,XM_001929569.6,XM_021094087.1,XM_021094362.1,XM_003358669.4,XM_021100455.1 |
| GO:0030414 | peptidase inhibitor activity | 0.65959 | XM_021074039.1 |
| GO:0061134 | peptidase regulator activity | 0.65959 | XM_021074039.1 |
| GO:0043231 | intracellular membrane-bounded organelle | 0.66136 | XM_021101423.1,XM_021090713.1,XM_021102607.1,XM_001929437.4,XM_001928598.6,XM_021100671.1,XM_001926951.4,XM_003132997.4,XM_021068253.1,XM_013981913.2,NM_001285967.1,XM_021095277.1,XM_021099431.1,XM_021078736.1,XM_003132993.4,XM_021094362.1,NM_001315597.1,XM_021088208.1,XM_005670867.3 |
| GO:0016796 | exonuclease activity, active with either ribo- or deoxyribonucleic acids and producing 5'-phosphomonoesters | 0.66366 | XM_001924990.5 |
| GO:0000152 | nuclear ubiquitin ligase complex | 0.66418 | XM_021088208.1 |
| GO:0005680 | anaphase-promoting complex | 0.66418 | XM_021088208.1 |
| GO:0031461 | cullin-RING ubiquitin ligase complex | 0.66418 | XM_021088208.1 |
| GO:0043227 | membrane-bounded organelle | 0.6648 | XM_013981913.2,NM_001285967.1,XM_021099431.1,XM_021078736.1,XM_021095277.1,XM_021068253.1,XM_003132993.4,XM_021094362.1,NM_001315597.1,XM_021088208.1,XM_005670867.3,XM_001928598.6,XM_021100671.1,XM_021090713.1,XM_021101423.1,XM_001929437.4,XM_021102607.1,XM_001926951.4,XM_003132997.4 |
| GO:0008219 | cell death | 0.66616 | NM_001112688.1 |
| GO:0016265 | death | 0.66616 | NM_001112688.1 |
| GO:0051179 | localization | 0.66875 | XM_013989320.2,XM_021091087.1,XM_021100487.1,XM_005670867.3,XM_003126445.6,XM_013996640.2,XM_021070329.1,XM_021074028.1,XM_021071922.1,XM_021094362.1,XM_021099431.1,XM_005670922.3,XM_021094364.1,XM_001927253.5,NM_001164021.1,XM_021101542.1,XM_021080387.1,XM_021088199.1,XM_021074039.1,XM_021075699.1,XM_021102607.1 |
| GO:0044707 | single-multicellular organism process | 0.66963 | XM_013993522.2,XM_003132993.4,XM_021092351.1,NM_001243297.1 |
| GO:0016032 | viral process | 0.67039 | XM_021102528.1,XM_021096440.1,XM_001929445.5 |
| GO:0006399 | tRNA metabolic process | 0.6707 | XM_021073780.1,XM_021074028.1 |
| GO:0042278 | purine nucleoside metabolic process | 0.67088 | XM_021100595.1 |
| GO:0046128 | purine ribonucleoside metabolic process | 0.67088 | XM_021100595.1 |
| GO:0032403 | protein complex binding | 0.67097 | XM_021088199.1,XM_021074380.1 |
| GO:0006259 | DNA metabolic process | 0.67278 | XM_013983166.2,XM_021094366.1,XM_013993522.2,XM_003358669.4,XM_003132997.4,XM_021072739.1,XM_005656513.3,XM_021099431.1 |
| GO:0043604 | amide biosynthetic process | 0.67279 | XM_021101430.1,XM_005666234.3,XM_021071138.1 |
| GO:0006974 | cellular response to DNA damage stimulus | 0.67282 | XM_021073419.1,XM_003358669.4,XM_005656513.3,XM_013983166.2 |
| GO:0000151 | ubiquitin ligase complex | 0.67437 | XM_021088208.1 |
| GO:0051258 | protein polymerization | 0.67609 | XM_003132993.4 |
| GO:0043623 | cellular protein complex assembly | 0.67799 | XM_003132993.4,XM_021070329.1 |
| GO:0006091 | generation of precursor metabolites and energy | 0.68029 | XM_001926818.5 |
| GO:0009611 | response to wounding | 0.68358 | XM_003132993.4 |
| GO:0042060 | wound healing | 0.68358 | XM_003132993.4 |
| GO:0006508 | proteolysis | 0.68854 | XM_021094087.1,XM_021078736.1,XM_021083730.1,XM_021101423.1,XM_001926562.4,XM_001929445.5 |
| GO:1901135 | carbohydrate derivative metabolic process | 0.68868 | XM_003359240.4,XM_021100595.1,NM_001001260.1 |
| GO:0005622 | intracellular | 0.6892 | XM_021101423.1,XM_021075699.1,XM_001929437.4,XM_001928598.6,XM_021079051.1,XM_005673970.3,NM_214387.1,NM_001285967.1,XM_021095277.1,XM_003130084.4,XM_021094362.1,NM_001315597.1,XM_021100455.1,XM_021062982.1,XM_003133210.6,XM_021090713.1,XM_021102607.1,XM_021083737.1,XM_021100671.1,XM_003132997.4,XM_001926951.4,XM_021068253.1,XM_021083741.1,XM_021073419.1,XM_013981913.2,XM_021078736.1,XM_021099431.1,XM_003132993.4,XM_021088208.1,XM_005670867.3,XM_001928339.4,NM_001112688.1,XM_021071138.1 |
| GO:0007155 | cell adhesion | 0.68977 | XM_021065362.1,XM_021074380.1,XM_021088199.1 |
| GO:0005102 | receptor binding | 0.69341 | XM_003132993.4,NM_213872.1,NM_214435.2,XM_001926013.5 |
| GO:0060589 | nucleoside-triphosphatase regulator activity | 0.69531 | XM_021099431.1 |
| GO:0090304 | nucleic acid metabolic process | 0.69613 | XM_021073419.1,XM_021095277.1,XM_021099431.1,XM_021094599.1,XM_021072739.1,XM_001929569.6,XM_021068253.1,NM_214387.1,XM_021083741.1,XM_021074028.1,XM_021094362.1,XM_021100455.1,XM_013993522.2,XM_003358669.4,XM_021073780.1,XM_001928598.6,XM_021100671.1,XM_005656513.3,XM_021101423.1,XM_021083737.1,XM_005673970.3,XM_021079051.1,XM_021094366.1,XM_013983166.2,XM_003132997.4,XM_001926951.4 |
| GO:0022610 | biological adhesion | 0.6994 | XM_021074380.1,XM_021088199.1,XM_021065362.1 |
| GO:0048193 | Golgi vesicle transport | 0.69988 | XM_005670867.3 |
| GO:0005623 | cell | 0.70014 | XM_001926951.4,XM_003132997.4,XM_021083737.1,XM_021102607.1,XM_003133210.6,XM_021090713.1,XM_021100671.1,XM_001928339.4,XM_005670867.3,XM_021088208.1,XM_003132993.4,XM_021071138.1,NM_001112688.1,XM_021083741.1,XM_021068253.1,XM_005670922.3,XM_021078736.1,XM_021099431.1,XM_013981913.2,XM_021073419.1,XM_021079051.1,XM_005673970.3,XM_001929437.4,XM_021075699.1,XM_021101423.1,XM_001928598.6,XM_021073780.1,XM_021074039.1,XM_021100455.1,NM_001315597.1,XM_021094362.1,XM_003130084.4,XM_021062982.1,NM_214387.1,XM_021095277.1,NM_001285967.1 |
| GO:0044464 | cell part | 0.70014 | NM_214387.1,XM_021095277.1,NM_001285967.1,NM_001315597.1,XM_021100455.1,XM_021094362.1,XM_003130084.4,XM_021062982.1,XM_021075699.1,XM_001929437.4,XM_021101423.1,XM_001928598.6,XM_021074039.1,XM_021073780.1,XM_021079051.1,XM_005673970.3,XM_021083741.1,XM_021068253.1,XM_005670922.3,XM_021099431.1,XM_021078736.1,XM_013981913.2,XM_021073419.1,XM_001928339.4,XM_021088208.1,XM_005670867.3,XM_003132993.4,XM_021071138.1,NM_001112688.1,XM_021083737.1,XM_021102607.1,XM_021090713.1,XM_003133210.6,XM_021100671.1,XM_001926951.4,XM_003132997.4 |
| GO:0009119 | ribonucleoside metabolic process | 0.70037 | XM_021100595.1 |
| GO:0031090 | organelle membrane | 0.70434 | XM_021078736.1,XM_021102607.1,NM_001315597.1 |
| GO:0007275 | multicellular organismal development | 0.7081 | XM_013993522.2,NM_001243297.1 |
| GO:0009055 | electron carrier activity | 0.70987 | XM_013986853.2 |
| GO:0033554 | cellular response to stress | 0.71193 | XM_013983166.2,XM_005656513.3,XM_003358669.4,XM_021073419.1 |
| GO:0007156 | homophilic cell adhesion via plasma membrane adhesion molecules | 0.71287 | XM_021065362.1 |
| GO:0070011 | peptidase activity, acting on L-amino acid peptides | 0.714 | XM_021078736.1,XM_021083730.1,XM_003484018.4,XM_001926562.4,XM_021101423.1,XM_001929445.5 |
| GO:0030154 | cell differentiation | 0.71572 | XM_003132997.4 |
| GO:0016482 | cytoplasmic transport | 0.71778 | XM_021075699.1,XM_021102607.1 |
| GO:0009165 | nucleotide biosynthetic process | 0.71903 | XM_021100595.1 |
| GO:1901293 | nucleoside phosphate biosynthetic process | 0.71903 | XM_021100595.1 |
| GO:1902494 | catalytic complex | 0.71947 | XM_021088208.1,XM_021078736.1,XM_021073419.1 |
| GO:0043603 | cellular amide metabolic process | 0.72033 | XM_021101430.1,XM_021071138.1,XM_005666234.3 |
| GO:0072509 | divalent inorganic cation transmembrane transporter activity | 0.7217 | XM_001927253.5 |
| GO:0098742 | cell-cell adhesion via plasma-membrane adhesion molecules | 0.72827 | XM_021065362.1 |
| GO:0016151 | nickel cation binding | 0.73024 | XM_003358669.4 |
| GO:0043232 | intracellular non-membrane-bounded organelle | 0.73294 | XM_003132993.4,XM_001929437.4,XM_021075699.1,XM_005670867.3,XM_005673970.3,XM_021095277.1,XM_021071138.1 |
| GO:0044767 | single-organism developmental process | 0.73554 | NM_001243297.1,XM_013993522.2,XM_003132997.4 |
| GO:0017038 | protein import | 0.73593 | XM_021102607.1 |
| GO:0000989 | transcription factor activity, transcription factor binding | 0.73627 | XM_021094362.1 |
| GO:0003712 | transcription cofactor activity | 0.73627 | XM_021094362.1 |
| GO:0006913 | nucleocytoplasmic transport | 0.73708 | XM_021075699.1 |
| GO:0004017 | adenylate kinase activity | 0.74153 | XM_003358669.4 |
| GO:0016787 | hydrolase activity | 0.74173 | XM_001926562.4,XM_021076863.1,XM_021079051.1,XM_001929445.5,XM_021074310.1,XM_021099082.1,XM_021074050.1,XM_021101542.1,XM_021101423.1,XM_001924990.5,XM_003121929.6,XM_021094087.1,XM_003484018.4,XM_013981430.2,NM_001001260.1,XM_021072282.1,XM_021073445.1,NM_214387.1,XM_013981913.2,XM_021069802.1,XM_001928314.6,XM_021091238.1,XM_021078736.1,XM_021099431.1,XM_021083730.1 |
| GO:0098609 | cell-cell adhesion | 0.74179 | XM_021065362.1 |
| GO:0019867 | outer membrane | 0.74184 | XM_021102607.1 |
| GO:0004536 | deoxyribonuclease activity | 0.74188 | XM_001924990.5 |
| GO:0009653 | anatomical structure morphogenesis | 0.74304 | XM_003132997.4 |
| GO:0051169 | nuclear transport | 0.74332 | XM_021075699.1 |
| GO:0009150 | purine ribonucleotide metabolic process | 0.74505 | XM_021100595.1 |
| GO:0009259 | ribonucleotide metabolic process | 0.74505 | XM_021100595.1 |
| GO:0016021 | integral component of membrane | 0.74938 | XM_021099082.1,XM_021080387.1,XM_021073967.1,XM_021074039.1,XM_021073780.1,XM_003126445.6,XM_021070329.1,XM_013996640.2,NM_001315597.1,XM_003132532.3,XM_021071922.1,XM_003355124.2,XM_021085108.1,NM_214435.2,XM_003484018.4,XM_001926013.5,XM_021090817.1,XM_013989320.2,XM_021091087.1,XM_001927733.6,XM_021073445.1,XM_021072739.1,XM_001927253.5,XM_021092351.1,XM_021099431.1,XM_021083673.1,XM_021094364.1,XM_021091238.1,NM_001285967.1 |
| GO:0004518 | nuclease activity | 0.75096 | XM_021091238.1,XM_001924990.5 |
| GO:0016820 | hydrolase activity, acting on acid anhydrides, catalyzing transmembrane movement of substances | 0.75117 | XM_001928314.6 |
| GO:0098805 | whole membrane | 0.75119 | XM_021102607.1 |
| GO:0006461 | protein complex assembly | 0.75152 | XM_003132993.4,XM_021070329.1,XM_021075699.1 |
| GO:0070271 | protein complex biogenesis | 0.75152 | XM_021070329.1,XM_021075699.1,XM_003132993.4 |
| GO:0006163 | purine nucleotide metabolic process | 0.75173 | XM_021100595.1 |
| GO:0034622 | cellular macromolecular complex assembly | 0.75228 | XM_021070329.1,XM_003132993.4 |
| GO:0019693 | ribose phosphate metabolic process | 0.75494 | XM_021100595.1 |
| GO:0032502 | developmental process | 0.75568 | NM_001243297.1,XM_013993522.2,XM_003132997.4 |
| GO:0044699 | single-organism process | 0.75597 | XM_021073780.1,XM_021075699.1,XM_021101430.1,NM_001164021.1,XM_005673970.3,XM_021074310.1,XM_021101416.1,XM_021101542.1,XM_021083673.1,NM_001285967.1,XM_001926818.5,XM_021072282.1,XM_021062982.1,XM_021070329.1,XM_003358669.4,XM_021088199.1,XM_021091960.1,XM_021067261.1,XM_021072792.1,XM_021062686.1,XM_021076863.1,XM_021100595.1,XM_021094364.1,XM_021099431.1,XM_021068991.1,XM_003359240.4,XM_021092351.1,XM_021073445.1,NM_001243297.1,XM_013989320.2,NM_001112688.1,XM_021074028.1,XM_005666234.3,NM_001167636.1,XM_021074039.1,XM_005656513.3,XM_013983166.2,XM_013986853.2,XM_021095277.1,XM_021101439.1,XM_001927253.5,NM_214387.1,XM_021085108.1,XM_021100487.1,XM_021063404.1,XM_021090817.1,XM_021094362.1,XM_003355124.2,XM_021100671.1,XM_001924990.5,XM_021102607.1,XM_003132997.4,XM_021080387.1,XM_021091955.1,XM_005670922.3,XM_013981913.2,XM_021073419.1,XM_001927733.6,XM_021091087.1,XM_021099391.1,XM_013996640.2,XM_013993522.2,XM_003126445.6,XM_005670867.3,XM_003132993.4,XM_021071922.1 |
| GO:0008233 | peptidase activity | 0.75693 | XM_003484018.4,XM_021083730.1,XM_021078736.1,XM_021101423.1,XM_001926562.4,XM_001929445.5 |
| GO:0044430 | cytoskeletal part | 0.75699 | XM_005670867.3,XM_021075699.1,XM_003132993.4 |
| GO:0010467 | gene expression | 0.75782 | XM_021073419.1,XM_021099431.1,XM_021095277.1,XM_021094599.1,XM_001929569.6,XM_021068253.1,NM_214387.1,XM_021083741.1,XM_021071138.1,XM_021074028.1,XM_021094362.1,XM_013993522.2,XM_021100455.1,XM_021100671.1,XM_001928598.6,XM_005656513.3,XM_021101423.1,XM_021083737.1,XM_005673970.3,XM_021094366.1,XM_013983166.2,XM_001926951.4 |
| GO:0031224 | intrinsic component of membrane | 0.75865 | XM_003132532.3,XM_021071922.1,XM_003355124.2,XM_021070329.1,NM_001315597.1,XM_013996640.2,XM_003126445.6,XM_021091087.1,XM_021090817.1,XM_013989320.2,XM_001926013.5,XM_021085108.1,XM_003484018.4,NM_214435.2,XM_001927253.5,XM_021092351.1,XM_021073445.1,XM_021072739.1,XM_001927733.6,NM_001285967.1,XM_021091238.1,XM_021094364.1,XM_021083673.1,XM_021099431.1,XM_021080387.1,XM_021099082.1,XM_021073967.1,XM_021073780.1,XM_021074039.1 |
| GO:0006950 | response to stress | 0.75945 | XM_021073419.1,NM_001243297.1,XM_003358669.4,XM_001929445.5,XM_005656513.3,XM_003132993.4,XM_013983166.2 |
| GO:0005524 | ATP binding | 0.76327 | XM_013986853.2,XM_021100595.1,XM_021096361.1,XM_013986189.2,XM_021071922.1,XM_021082806.1,XM_021075700.1,XM_021074028.1,XM_021101542.1,XM_021099557.1,XM_021073780.1,XM_021099431.1,XM_021101533.1,XM_021091960.1 |
| GO:0072521 | purine-containing compound metabolic process | 0.76389 | XM_021100595.1 |
| GO:0016776 | phosphotransferase activity, phosphate group as acceptor | 0.76436 | XM_003358669.4 |
| GO:0065003 | macromolecular complex assembly | 0.7653 | XM_021070329.1,XM_021075699.1,XM_003132993.4 |
| GO:0051301 | cell division | 0.76566 | XM_021099431.1 |
| GO:0032559 | adenyl ribonucleotide binding | 0.76701 | XM_021071922.1,XM_021082806.1,XM_021074028.1,XM_021075700.1,XM_021100595.1,XM_021096361.1,XM_013986189.2,XM_013986853.2,XM_021099557.1,XM_021101542.1,XM_021073780.1,XM_021099431.1,XM_021091960.1,XM_021101533.1 |
| GO:0003899 | DNA-directed RNA polymerase activity | 0.77189 | XM_013983166.2 |
| GO:0019201 | nucleotide kinase activity | 0.77418 | XM_003358669.4 |
| GO:0030554 | adenyl nucleotide binding | 0.77677 | XM_021099431.1,XM_021073780.1,XM_021101533.1,XM_021091960.1,XM_021101542.1,XM_021099557.1,XM_021096361.1,XM_021100595.1,XM_013986189.2,XM_013986853.2,XM_021071922.1,XM_021074028.1,XM_021082806.1,XM_021075700.1 |
| GO:0043168 | anion binding | 0.78018 | XM_021091960.1,XM_021101533.1,XM_021073780.1,XM_013988351.2,XM_013986853.2,XM_013986189.2,XM_021100595.1,XM_021101542.1,XM_021099557.1,XM_001927253.5,XM_001926818.5,XM_021099431.1,XM_021096361.1,XM_021075700.1,XM_021082806.1,XM_021074028.1,XM_003130084.4,XM_021071922.1 |
| GO:0004175 | endopeptidase activity | 0.78141 | XM_003484018.4,XM_021101423.1,XM_001926562.4,XM_001929445.5 |
| GO:0043226 | organelle | 0.78313 | XM_021100671.1,XM_001928598.6,XM_001929437.4,XM_021102607.1,XM_021075699.1,XM_021101423.1,XM_021090713.1,XM_005673970.3,XM_001926951.4,XM_003132997.4,XM_005670922.3,XM_021095277.1,XM_021099431.1,XM_021078736.1,XM_013981913.2,NM_001285967.1,XM_021068253.1,XM_021071138.1,NM_001315597.1,XM_021088208.1,XM_005670867.3,XM_021094362.1,XM_003132993.4 |
| GO:0034645 | cellular macromolecule biosynthetic process | 0.7833 | XM_021073419.1,XM_021099431.1,XM_021095277.1,XM_021094599.1,XM_001929569.6,XM_021068253.1,NM_214387.1,XM_021083741.1,XM_021071138.1,XM_021094362.1,XM_013993522.2,XM_021100455.1,XM_021100671.1,XM_001928598.6,XM_005656513.3,XM_021101423.1,XM_021083737.1,XM_005673970.3,XM_021094366.1,XM_013983166.2,XM_001926951.4 |
| GO:0000988 | transcription factor activity, protein binding | 0.78488 | XM_021094362.1 |
| GO:0071822 | protein complex subunit organization | 0.78582 | XM_021070329.1,XM_021075699.1,XM_003132993.4 |
| GO:0008152 | metabolic process | 0.79141 | XM_013993522.2,XM_021096361.1,XM_021074028.1,XM_021082806.1,XM_021075700.1,XM_021071922.1,XM_021071138.1,NM_001243297.1,XM_003359240.4,XM_021083741.1,NM_001001260.1,XM_021068253.1,XM_005670922.3,XM_021099431.1,XM_021078736.1,XM_013981913.2,XM_021073419.1,XM_001926951.4,XM_021062686.1,XM_021099557.1,XM_003132997.4,XM_001926562.4,XM_021100595.1,XM_013986189.2,XM_021091955.1,XM_021083737.1,XM_021091960.1,XM_021101533.1,XM_021100671.1,XM_001924990.5,XM_021100455.1,XM_003358669.4,XM_021094362.1,XM_021094087.1,XM_021063404.1,XM_001926818.5,XM_001929569.6,NM_214387.1,XM_021072739.1,XM_021072282.1,XM_021094599.1,XM_005670915.3,XM_021096140.1,XM_021095277.1,XM_021083730.1,XM_021091183.1,XM_021074310.1,XM_013986853.2,XM_013983166.2,XM_021094366.1,XM_001929445.5,XM_021079051.1,XM_021101430.1,XM_005673970.3,XM_021101423.1,XM_005656513.3,XM_001928598.6,XM_005666234.3,NM_001167636.1,XM_021073780.1 |
| GO:0034660 | ncRNA metabolic process | 0.79225 | XM_021073780.1,XM_021074028.1 |
| GO:0009059 | macromolecule biosynthetic process | 0.79397 | XM_005673970.3,XM_001926951.4,XM_013983166.2,XM_021094366.1,XM_001928598.6,XM_021100671.1,XM_021083737.1,XM_021101423.1,XM_005656513.3,XM_021071138.1,XM_013993522.2,XM_021100455.1,XM_021094362.1,XM_021094599.1,XM_021099431.1,XM_021095277.1,XM_021073419.1,XM_021083741.1,XM_021068253.1,NM_214387.1,XM_001929569.6 |
| GO:0019031 | viral envelope | 0.79985 | XM_005656513.3 |
| GO:0036338 | viral membrane | 0.79985 | XM_005656513.3 |
| GO:0004857 | enzyme inhibitor activity | 0.80001 | XM_021074039.1 |
| GO:0008033 | tRNA processing | 0.80024 | XM_021074028.1 |
| GO:0035639 | purine ribonucleoside triphosphate binding | 0.80377 | XM_021075700.1,XM_021071922.1,XM_021074028.1,XM_021082806.1,XM_021100595.1,XM_013986189.2,XM_021096361.1,XM_013986853.2,XM_021099557.1,XM_021101542.1,XM_021073780.1,XM_021099431.1,XM_001927253.5,XM_021101533.1,XM_021091960.1 |
| GO:0042623 | ATPase activity, coupled | 0.80467 | XM_001928314.6 |
| GO:0005856 | cytoskeleton | 0.8053 | XM_005670867.3,XM_021075699.1,XM_003132993.4 |
| GO:0006281 | DNA repair | 0.80541 | XM_005656513.3,XM_013983166.2,XM_003358669.4 |
| GO:0007166 | cell surface receptor signaling pathway | 0.80641 | XM_005673970.3 |
| GO:0001883 | purine nucleoside binding | 0.80698 | XM_001927253.5,XM_021091960.1,XM_021101533.1,XM_021073780.1,XM_021099431.1,XM_021100595.1,XM_013986853.2,XM_021096361.1,XM_013986189.2,XM_021071922.1,XM_021075700.1,XM_021082806.1,XM_021074028.1,XM_021099557.1,XM_021101542.1 |
| GO:0032549 | ribonucleoside binding | 0.80698 | XM_021101533.1,XM_021091960.1,XM_001927253.5,XM_021099431.1,XM_021073780.1,XM_021099557.1,XM_021101542.1,XM_021100595.1,XM_021096361.1,XM_013986189.2,XM_013986853.2,XM_021074028.1,XM_021075700.1,XM_021071922.1,XM_021082806.1 |
| GO:0032550 | purine ribonucleoside binding | 0.80698 | XM_001927253.5,XM_021091960.1,XM_021101533.1,XM_021073780.1,XM_021099431.1,XM_021074028.1,XM_021075700.1,XM_021071922.1,XM_021082806.1,XM_021100595.1,XM_013986853.2,XM_021096361.1,XM_013986189.2,XM_021099557.1,XM_021101542.1 |
| GO:0048869 | cellular developmental process | 0.80823 | XM_003132997.4 |
| GO:0019205 | nucleobase-containing compound kinase activity | 0.80876 | XM_003358669.4 |
| GO:0009057 | macromolecule catabolic process | 0.80933 | NM_001001260.1 |
| GO:0001882 | nucleoside binding | 0.80995 | XM_021100595.1,XM_013986189.2,XM_021096361.1,XM_013986853.2,XM_021074028.1,XM_021071922.1,XM_021075700.1,XM_021082806.1,XM_021101542.1,XM_021099557.1,XM_021073780.1,XM_021099431.1,XM_001927253.5,XM_021101533.1,XM_021091960.1 |
| GO:0032555 | purine ribonucleotide binding | 0.81083 | XM_021099431.1,XM_021073780.1,XM_021101533.1,XM_021091960.1,XM_001927253.5,XM_021099557.1,XM_021101542.1,XM_021074028.1,XM_021082806.1,XM_021075700.1,XM_021071922.1,XM_021096361.1,XM_021100595.1,XM_013986853.2,XM_013986189.2 |
| GO:0000278 | mitotic cell cycle | 0.81223 | XM_021099431.1 |
| GO:1903047 | mitotic cell cycle process | 0.81223 | XM_021099431.1 |
| GO:0043229 | intracellular organelle | 0.81759 | XM_005673970.3,XM_001926951.4,XM_003132997.4,XM_021100671.1,XM_001928598.6,XM_021102607.1,XM_021075699.1,XM_001929437.4,XM_021090713.1,XM_021101423.1,XM_021071138.1,NM_001315597.1,XM_021088208.1,XM_005670867.3,XM_021094362.1,XM_003132993.4,XM_021099431.1,XM_021095277.1,XM_021078736.1,XM_013981913.2,NM_001285967.1,XM_021068253.1 |
| GO:0017076 | purine nucleotide binding | 0.81906 | XM_021099557.1,XM_021101542.1,XM_021096361.1,XM_021100595.1,XM_013986189.2,XM_013986853.2,XM_021082806.1,XM_021074028.1,XM_021075700.1,XM_021071922.1,XM_021101533.1,XM_021091960.1,XM_001927253.5,XM_021099431.1,XM_021073780.1 |
| GO:0006366 | transcription from RNA polymerase II promoter | 0.82072 | XM_021094362.1 |
| GO:0016798 | hydrolase activity, acting on glycosyl bonds | 0.8246 | XM_003121929.6 |
| GO:0022607 | cellular component assembly | 0.82479 | XM_021075699.1,XM_021070329.1,XM_003132993.4,XM_021099431.1 |
| GO:0048519 | negative regulation of biological process | 0.82552 | XM_001929445.5 |
| GO:0032553 | ribonucleotide binding | 0.82778 | XM_021099431.1,XM_021073780.1,XM_021101533.1,XM_021091960.1,XM_001927253.5,XM_021101542.1,XM_021099557.1,XM_021071922.1,XM_021082806.1,XM_021074028.1,XM_021075700.1,XM_021096361.1,XM_021100595.1,XM_013986189.2,XM_013986853.2 |
| GO:0000166 | nucleotide binding | 0.83218 | XM_021096361.1,XM_021075700.1,XM_021082806.1,XM_021071922.1,XM_021074028.1,XM_021091238.1,XM_021099431.1,XM_001927253.5,XM_001926818.5,XM_013986853.2,XM_013986189.2,XM_021100595.1,XM_021099557.1,XM_021101542.1,XM_021073780.1,XM_021101533.1,XM_021091960.1 |
| GO:1901265 | nucleoside phosphate binding | 0.83218 | XM_021096361.1,XM_021074028.1,XM_021082806.1,XM_021071922.1,XM_021075700.1,XM_001927253.5,XM_001926818.5,XM_021091238.1,XM_021099431.1,XM_021100595.1,XM_013986853.2,XM_013986189.2,XM_021101542.1,XM_021099557.1,XM_021101533.1,XM_021091960.1,XM_021073780.1 |
| GO:0009056 | catabolic process | 0.83357 | XM_001924990.5,XM_005670922.3,NM_001001260.1 |
| GO:0071702 | organic substance transport | 0.83442 | XM_021075699.1,XM_021102607.1,XM_021070329.1,XM_013996640.2,XM_021099431.1,XM_005670922.3 |
| GO:0043234 | protein complex | 0.83478 | NM_001243297.1,XM_003132993.4,XM_021094362.1,XM_003130084.4,XM_001928339.4,XM_021088208.1,XM_005670867.3,XM_021073419.1,NM_001105289.1,XM_021078736.1,XM_021095277.1,XM_021102607.1 |
| GO:0016746 | transferase activity, transferring acyl groups | 0.83664 | XM_021068991.1 |
| GO:0005575 | cellular_component | 0.83746 | XM_021101423.1,XM_021073967.1,XM_001929437.4,XM_021075699.1,XM_021073780.1,XM_003121929.6,XM_021101542.1,NM_001164021.1,XM_005673970.3,XM_021072739.1,XM_001929542.5,NM_001285967.1,XM_021083673.1,XM_021083730.1,XM_021075702.1,XM_021070329.1,NM_001315597.1,XM_021062982.1,XM_001926013.5,XM_003133210.6,XM_021065362.1,NM_001105289.1,XM_021088199.1,XM_001926951.4,XM_021092351.1,XM_003358668.4,XM_021068253.1,XM_021073445.1,XM_021083741.1,NM_213872.1,XM_021094364.1,XM_021099431.1,XM_001928339.4,NM_001243297.1,NM_001112688.1,XM_013989320.2,XM_021071138.1,NM_214435.2,XM_003484018.4,XM_013988558.2,XM_005656513.3,XM_021074039.1,XM_001928598.6,XM_021079051.1,XM_001929445.5,XM_021099082.1,XM_001927253.5,NM_214387.1,XM_021091238.1,XM_021095277.1,XM_021094362.1,XM_003132532.3,XM_003130084.4,XM_003355124.2,XM_021100455.1,XM_021100487.1,XM_021090817.1,XM_021085108.1,XM_021090713.1,XM_021083737.1,XM_021102607.1,XM_021100671.1,XM_021080387.1,XM_003132997.4,XM_005656504.3,XM_001927733.6,XM_013981913.2,XM_021073419.1,XM_005670922.3,XM_021078736.1,XM_003132993.4,XM_021071922.1,XM_013996640.2,XM_013993522.2,XM_003126445.6,XM_021088208.1,XM_005670867.3,XM_021091087.1,NM_001004026.1 |
| GO:0034062 | RNA polymerase activity | 0.83751 | XM_013983166.2 |
| GO:0009117 | nucleotide metabolic process | 0.84112 | XM_021100595.1 |
| GO:0044446 | intracellular organelle part | 0.84258 | XM_021094362.1,XM_003132993.4,NM_001315597.1,XM_005670867.3,XM_021088208.1,XM_021078736.1,XM_021095277.1,XM_021075699.1,XM_021102607.1,XM_001929437.4 |
| GO:0016779 | nucleotidyltransferase activity | 0.84438 | XM_021063404.1,XM_013983166.2 |
| GO:0006753 | nucleoside phosphate metabolic process | 0.84445 | XM_021100595.1 |
| GO:0030234 | enzyme regulator activity | 0.84861 | XM_021074039.1,XM_021099431.1 |
| GO:0090407 | organophosphate biosynthetic process | 0.84925 | XM_021100595.1 |
| GO:0036094 | small molecule binding | 0.85006 | XM_021099431.1,XM_021091238.1,XM_001926818.5,XM_001927253.5,XM_021082806.1,XM_021074028.1,XM_021075700.1,XM_021071922.1,XM_021096361.1,XM_021073780.1,XM_021101533.1,XM_021091960.1,XM_021101542.1,XM_021099557.1,XM_013986189.2,XM_013986853.2,XM_021100595.1 |
| GO:0044425 | membrane part | 0.85013 | XM_021074039.1,XM_021073780.1,XM_021073967.1,XM_021102607.1,XM_021080387.1,XM_021099082.1,NM_001285967.1,XM_021094364.1,XM_021083673.1,XM_021091238.1,XM_021078736.1,XM_021099431.1,XM_021092351.1,XM_001927253.5,XM_021072739.1,XM_021073445.1,XM_001927733.6,XM_021091087.1,XM_021090817.1,XM_013989320.2,XM_001926013.5,XM_003484018.4,XM_021085108.1,NM_214435.2,XM_003355124.2,XM_021071922.1,XM_003132532.3,XM_013996640.2,XM_021070329.1,XM_003126445.6,NM_001315597.1 |
| GO:0044763 | single-organism cellular process | 0.8528 | XM_021073780.1,XM_021074039.1,NM_001167636.1,XM_005666234.3,XM_005656513.3,XM_021075699.1,XM_005673970.3,NM_001164021.1,XM_021101430.1,XM_013983166.2,XM_021101542.1,NM_001285967.1,XM_021083673.1,NM_214387.1,XM_001926818.5,XM_021101439.1,XM_021090817.1,XM_021062982.1,XM_021063404.1,XM_021085108.1,XM_003355124.2,XM_021094362.1,XM_021070329.1,XM_003358669.4,XM_021088199.1,XM_021100671.1,XM_021091960.1,XM_021102607.1,XM_021067261.1,XM_021100595.1,XM_021080387.1,XM_021076863.1,XM_003132997.4,XM_021072792.1,XM_021073419.1,XM_013981913.2,XM_021099431.1,XM_021094364.1,XM_005670922.3,XM_021073445.1,XM_021092351.1,XM_001927733.6,XM_003359240.4,XM_021068991.1,NM_001112688.1,XM_021091087.1,XM_021074028.1,XM_003132993.4,XM_005670867.3,XM_021099391.1,XM_013996640.2,XM_003126445.6 |
| GO:0044422 | organelle part | 0.85287 | XM_021094362.1,XM_003132993.4,XM_005670867.3,XM_021088208.1,NM_001315597.1,XM_021075699.1,XM_021102607.1,XM_001929437.4,XM_021095277.1,XM_021078736.1 |
| GO:0044424 | intracellular part | 0.85778 | XM_021068253.1,XM_021099431.1,XM_021078736.1,XM_021095277.1,XM_013981913.2,NM_001285967.1,XM_021073419.1,NM_001315597.1,XM_001928339.4,XM_005670867.3,XM_021088208.1,XM_021094362.1,XM_003132993.4,XM_003130084.4,XM_021071138.1,XM_021062982.1,XM_021102607.1,XM_001929437.4,XM_021075699.1,XM_021090713.1,XM_021101423.1,XM_021100671.1,XM_001928598.6,XM_001926951.4,XM_003132997.4,XM_021079051.1,XM_005673970.3 |
| GO:0004386 | helicase activity | 0.8614 | XM_021101542.1 |
| GO:0044444 | cytoplasmic part | 0.86197 | XM_001928339.4,XM_021075699.1,XM_021102607.1,XM_003130084.4,XM_021078736.1,XM_021071138.1,XM_021073419.1 |
| GO:0097367 | carbohydrate derivative binding | 0.86231 | XM_021101542.1,XM_021099557.1,XM_021074028.1,XM_021071922.1,XM_021075700.1,XM_021082806.1,XM_021100595.1,XM_021096361.1,XM_013986189.2,XM_013986853.2,XM_021101533.1,XM_021091960.1,XM_001927253.5,XM_021099431.1,XM_021073780.1 |
| GO:0005840 | ribosome | 0.86462 | XM_021071138.1 |
| GO:0072594 | establishment of protein localization to organelle | 0.86643 | XM_021102607.1 |
| GO:0005737 | cytoplasm | 0.86729 | XM_021102607.1,XM_021075699.1,XM_021073419.1,XM_021078736.1,XM_021099431.1,XM_003130084.4,XM_021079051.1,XM_001928339.4,XM_021062982.1,XM_021071138.1 |
| GO:0003735 | structural constituent of ribosome | 0.86735 | XM_021071138.1 |
| GO:0019058 | viral life cycle | 0.87027 | XM_021102528.1 |
| GO:0046907 | intracellular transport | 0.87187 | XM_005670867.3,XM_021102607.1,XM_021075699.1,XM_021099431.1 |
| GO:0065008 | regulation of biological quality | 0.87205 | XM_001929445.5,XM_003132993.4 |
| GO:0005654 | nucleoplasm | 0.87596 | XM_021094362.1 |
| GO:0044451 | nucleoplasm part | 0.87596 | XM_021094362.1 |
| GO:1902582 | single-organism intracellular transport | 0.87894 | XM_005670867.3,XM_021102607.1 |
| GO:0006605 | protein targeting | 0.88753 | XM_021102607.1 |
| GO:0033365 | protein localization to organelle | 0.8943 | XM_021102607.1 |
| GO:0034470 | ncRNA processing | 0.89534 | XM_021074028.1 |
| GO:1902580 | single-organism cellular localization | 0.89819 | XM_021102607.1 |
| GO:0044248 | cellular catabolic process | 0.8987 | XM_001924990.5,XM_005670922.3 |
| GO:0044085 | cellular component biogenesis | 0.89935 | XM_021099431.1,XM_003132993.4,XM_021075699.1,XM_021070329.1 |
| GO:1901137 | carbohydrate derivative biosynthetic process | 0.90672 | XM_021100595.1 |
| GO:0008236 | serine-type peptidase activity | 0.90833 | XM_021083730.1 |
| GO:0017171 | serine hydrolase activity | 0.90833 | XM_021083730.1 |
| GO:0005525 | GTP binding | 0.91403 | XM_021101542.1,XM_001927253.5 |
| GO:0051649 | establishment of localization in cell | 0.91552 | XM_021102607.1,XM_005670867.3,XM_021075699.1,XM_021099431.1 |
| GO:0032991 | macromolecular complex | 0.9161 | XM_021095277.1,XM_021078736.1,NM_001105289.1,XM_021073419.1,XM_021102607.1,XM_021071138.1,NM_001243297.1,XM_021088208.1,XM_005670867.3,XM_001928339.4,XM_003130084.4,XM_021094362.1,XM_003132993.4 |
| GO:0016020 | membrane | 0.91785 | NM_001164021.1,XM_021099082.1,XM_021101542.1,XM_021074039.1,XM_021073780.1,XM_021073967.1,XM_021101423.1,XM_013988558.2,XM_001926013.5,XM_021085108.1,XM_021100487.1,XM_021090817.1,XM_021070329.1,NM_001315597.1,XM_021075702.1,XM_021094362.1,XM_003132532.3,XM_003355124.2,XM_021091238.1,XM_021083673.1,XM_021083730.1,XM_001929542.5,NM_001285967.1,XM_001927253.5,XM_021072739.1,XM_005656504.3,XM_021080387.1,XM_021088199.1,XM_021065362.1,XM_021102607.1,NM_214435.2,XM_003484018.4,XM_021091087.1,XM_013989320.2,XM_013996640.2,XM_003126445.6,XM_005670867.3,XM_021071922.1,XM_021094364.1,XM_021078736.1,XM_021099431.1,XM_001927733.6,XM_021092351.1,XM_021073445.1 |
| GO:0032561 | guanyl ribonucleotide binding | 0.91802 | XM_021101542.1,XM_001927253.5 |
| GO:0050789 | regulation of biological process | 0.91806 | XM_021092351.1,XM_021068253.1,XM_021068991.1,XM_021083741.1,XM_001927733.6,XM_021073419.1,XM_005670922.3,XM_021099431.1,XM_003132993.4,XM_013993522.2,XM_021099391.1,XM_021083737.1,XM_021091960.1,XM_021100671.1,XM_001924990.5,XM_021076863.1,XM_001926951.4,XM_021072792.1,XM_021067261.1,XM_001929569.6,NM_214387.1,XM_021101439.1,NM_001285967.1,XM_021083673.1,XM_021094599.1,XM_021095277.1,XM_021094362.1,XM_003355124.2,XM_021100455.1,XM_021070329.1,XM_021062982.1,XM_021090817.1,XM_021085108.1,XM_021101423.1,XM_005656513.3,XM_001928598.6,XM_013983166.2,XM_001929445.5,XM_021094366.1,XM_005673970.3 |
| GO:0019001 | guanyl nucleotide binding | 0.91891 | XM_001927253.5,XM_021101542.1 |
| GO:0016887 | ATPase activity | 0.92271 | XM_001928314.6,XM_021101542.1 |
| GO:0050794 | regulation of cellular process | 0.92336 | XM_013983166.2,XM_021094366.1,XM_005673970.3,XM_021101423.1,XM_005656513.3,XM_001928598.6,XM_021094362.1,XM_003355124.2,XM_021070329.1,XM_021100455.1,XM_021062982.1,XM_021090817.1,XM_021085108.1,NM_214387.1,XM_001929569.6,XM_021101439.1,NM_001285967.1,XM_021094599.1,XM_021083673.1,XM_021095277.1,XM_021076863.1,XM_021072792.1,XM_001926951.4,XM_021067261.1,XM_021083737.1,XM_021091960.1,XM_021100671.1,XM_001924990.5,XM_003132993.4,XM_021099391.1,XM_013993522.2,XM_021092351.1,XM_021068253.1,XM_021068991.1,XM_021083741.1,XM_001927733.6,XM_021073419.1,XM_005670922.3,XM_021099431.1 |
| GO:1990234 | transferase complex | 0.92486 | XM_021088208.1 |
| GO:0098588 | bounding membrane of organelle | 0.93012 | XM_021102607.1 |
| GO:1901575 | organic substance catabolic process | 0.93274 | NM_001001260.1 |
| GO:0051641 | cellular localization | 0.94002 | XM_021099431.1,XM_005670867.3,XM_021102607.1,XM_021075699.1 |
| GO:0065007 | biological regulation | 0.9422 | XM_001924990.5,XM_021100671.1,XM_021091960.1,XM_021083737.1,XM_021067261.1,XM_001926951.4,XM_021072792.1,XM_021076863.1,XM_021099431.1,XM_005670922.3,XM_021073419.1,XM_021083741.1,XM_001927733.6,XM_021068991.1,XM_021068253.1,XM_021092351.1,XM_013993522.2,XM_021099391.1,XM_003132993.4,XM_001928598.6,XM_005656513.3,XM_021101423.1,XM_005673970.3,XM_021094366.1,XM_001929445.5,XM_013983166.2,XM_021095277.1,XM_021094599.1,XM_021083673.1,NM_001285967.1,XM_021101439.1,XM_001929569.6,NM_214387.1,XM_021085108.1,XM_021090817.1,XM_021062982.1,XM_021070329.1,XM_021100455.1,XM_003355124.2,XM_021094362.1 |
| GO:0046983 | protein dimerization activity | 0.94399 | XM_021094599.1 |
| GO:0008150 | biological_process | 0.94627 | XM_021062982.1,XM_021074380.1,XM_001926013.5,XM_021094087.1,XM_003358669.4,XM_021070329.1,NM_001285967.1,XM_021083730.1,XM_021083673.1,XM_021072739.1,XM_021072282.1,XM_001926818.5,XM_005673970.3,NM_001164021.1,XM_021101430.1,XM_021094366.1,XM_021101542.1,XM_021101416.1,XM_021074310.1,XM_021073780.1,XM_021101423.1,XM_001929437.4,XM_021075699.1,NM_001112688.1,XM_013989320.2,NM_001243297.1,NM_214435.2,XM_021071138.1,XM_021074028.1,XM_021099431.1,XM_021094364.1,XM_021073445.1,XM_021068253.1,XM_021092351.1,XM_021102528.1,XM_003359240.4,XM_021083741.1,XM_021068991.1,XM_021067261.1,XM_021076863.1,XM_021100595.1,XM_001926562.4,XM_021062686.1,XM_001926951.4,XM_021072792.1,XM_021065362.1,XM_021088199.1,XM_021096440.1,XM_021091960.1,XM_021101533.1,XM_021090817.1,XM_021063404.1,XM_021100487.1,XM_021085108.1,XM_003355124.2,XM_021094362.1,XM_021100455.1,XM_021096140.1,XM_021095277.1,XM_005670915.3,XM_021094599.1,XM_001929569.6,NM_214387.1,XM_001927253.5,XM_021101439.1,XM_001929445.5,XM_021079051.1,XM_013983166.2,XM_013986853.2,XM_021091183.1,XM_021074039.1,NM_001167636.1,XM_005666234.3,XM_001928598.6,XM_005656513.3,XM_021091087.1,XM_021071922.1,XM_021082806.1,XM_021075700.1,XM_003132993.4,XM_021096361.1,XM_005670867.3,XM_021099391.1,XM_013996640.2,XM_003126445.6,XM_013993522.2,XM_021073419.1,XM_013981913.2,XM_021078736.1,XM_005670922.3,NM_001001260.1,XM_001927733.6,XM_021076633.1,XM_021091955.1,XM_013986189.2,XM_021080387.1,XM_021099557.1,XM_003132997.4,XM_001924990.5,XM_021100671.1,XM_021102607.1,XM_021083737.1 |
| GO:0019637 | organophosphate metabolic process | 0.95097 | XM_021100595.1 |
| GO:0098796 | membrane protein complex | 0.95131 | XM_021102607.1,XM_021078736.1 |
| GO:0033036 | macromolecule localization | 0.95196 | XM_021102607.1,XM_021075699.1,XM_021099431.1,XM_005670922.3 |
| GO:0006412 | translation | 0.95805 | XM_021071138.1 |
| GO:0003924 | GTPase activity | 0.95824 | XM_021074050.1 |
| GO:0016817 | hydrolase activity, acting on acid anhydrides | 0.95908 | XM_001928314.6,XM_021099431.1,XM_021072282.1,XM_021101542.1,XM_021074050.1 |
| GO:0006260 | DNA replication | 0.96165 | XM_021099431.1 |
| GO:0043043 | peptide biosynthetic process | 0.96228 | XM_021071138.1 |
| GO:0016192 | vesicle-mediated transport | 0.96267 | XM_005670867.3 |
| GO:0030529 | ribonucleoprotein complex | 0.9636 | XM_021071138.1 |
| GO:0012505 | endomembrane system | 0.96417 | NM_001315597.1 |
| GO:0006886 | intracellular protein transport | 0.9644 | XM_021102607.1,XM_021099431.1 |
| GO:0006518 | peptide metabolic process | 0.9675 | XM_021071138.1 |
| GO:0016818 | hydrolase activity, acting on acid anhydrides, in phosphorus-containing anhydrides | 0.97373 | XM_021099431.1,XM_001928314.6,XM_021074050.1,XM_021101542.1 |
| GO:0007165 | signal transduction | 0.9763 | XM_003355124.2,XM_003132993.4,XM_021070329.1,XM_021099391.1,XM_021090817.1,XM_021062982.1,XM_021085108.1,NM_214387.1,XM_021092351.1,XM_001927733.6,XM_021068991.1,XM_021101439.1,XM_021099431.1,XM_021083673.1,XM_013983166.2,XM_021076863.1,XM_021072792.1,XM_005673970.3,XM_021067261.1,XM_005656513.3,XM_021091960.1 |
| GO:0044700 | single organism signaling | 0.97767 | XM_021099431.1,XM_021083673.1,NM_214387.1,XM_021092351.1,XM_001927733.6,XM_021101439.1,XM_021068991.1,XM_021090817.1,XM_021062982.1,XM_021085108.1,XM_003355124.2,XM_003132993.4,XM_021099391.1,XM_021070329.1,XM_005656513.3,XM_021091960.1,XM_005673970.3,XM_021067261.1,XM_021076863.1,XM_013983166.2,XM_021072792.1 |
| GO:0034613 | cellular protein localization | 0.97796 | XM_021102607.1,XM_021099431.1 |
| GO:0070727 | cellular macromolecule localization | 0.97796 | XM_021099431.1,XM_021102607.1 |
| GO:0023052 | signaling | 0.97848 | XM_021067261.1,XM_005673970.3,XM_021072792.1,XM_013983166.2,XM_021076863.1,XM_021091960.1,XM_005656513.3,XM_021085108.1,XM_021090817.1,XM_021062982.1,XM_021099391.1,XM_021070329.1,XM_003355124.2,XM_003132993.4,XM_021099431.1,XM_021083673.1,XM_001927733.6,XM_021068991.1,XM_021101439.1,NM_214387.1,XM_021092351.1 |
| GO:0007154 | cell communication | 0.98189 | XM_021072792.1,XM_013983166.2,XM_021076863.1,XM_021067261.1,XM_005673970.3,XM_021091960.1,XM_005656513.3,XM_021099391.1,XM_021070329.1,XM_003355124.2,XM_003132993.4,XM_021085108.1,XM_021090817.1,XM_021062982.1,XM_001927733.6,XM_021101439.1,XM_021068991.1,NM_214387.1,XM_021092351.1,XM_021099431.1,XM_021083673.1 |
| GO:0050896 | response to stimulus | 0.98223 | XM_005656513.3,XM_021091960.1,XM_021074039.1,XM_013983166.2,XM_021076863.1,XM_001929445.5,XM_021072792.1,XM_005673970.3,XM_021067261.1,XM_021092351.1,NM_214387.1,XM_021101439.1,XM_021068991.1,XM_001927733.6,XM_021073419.1,XM_021083673.1,XM_005670922.3,XM_021099431.1,XM_003132993.4,XM_003355124.2,XM_003358669.4,XM_021099391.1,XM_021070329.1,XM_021062982.1,NM_001243297.1,XM_021090817.1,XM_001926013.5,XM_021085108.1 |
| GO:0006396 | RNA processing | 0.98302 | XM_021074028.1 |
| GO:0015031 | protein transport | 0.98561 | XM_021099431.1,XM_021102607.1 |
| GO:0045184 | establishment of protein localization | 0.98749 | XM_021102607.1,XM_021099431.1 |
| GO:0017111 | nucleoside-triphosphatase activity | 0.98807 | XM_021101542.1,XM_021074050.1,XM_001928314.6 |
| GO:0004888 | transmembrane signaling receptor activity | 0.98837 | XM_021074039.1,XM_021083673.1,XM_021092351.1,XM_001927733.6,XM_021090817.1,XM_005656504.3,XM_021085108.1,XM_021075702.1,XM_003355124.2 |
| GO:0016462 | pyrophosphatase activity | 0.98863 | XM_021101542.1,XM_021074050.1,XM_001928314.6 |
| GO:0051716 | cellular response to stimulus | 0.99014 | XM_021091960.1,XM_005656513.3,XM_021072792.1,XM_021076863.1,XM_013983166.2,XM_021067261.1,XM_005673970.3,XM_001927733.6,XM_021101439.1,XM_021068991.1,NM_214387.1,XM_021092351.1,XM_021099431.1,XM_021083673.1,XM_021073419.1,XM_021070329.1,XM_021099391.1,XM_003358669.4,XM_003355124.2,XM_003132993.4,XM_021085108.1,XM_021090817.1,XM_021062982.1 |
| GO:0038023 | signaling receptor activity | 0.99097 | XM_001927733.6,XM_021092351.1,XM_021083673.1,XM_021074039.1,XM_003355124.2,XM_021075702.1,XM_005656504.3,XM_021085108.1,XM_021090817.1 |
| GO:0008104 | protein localization | 0.99118 | XM_021099431.1,XM_021102607.1 |
| GO:0004872 | receptor activity | 0.99401 | XM_021083673.1,XM_021074039.1,XM_001927733.6,XM_021092351.1,XM_021085108.1,XM_005656504.3,XM_021090817.1,XM_021075702.1,XM_003355124.2 |
| GO:0004871 | signal transducer activity | 0.9966 | XM_021092351.1,XM_001927733.6,XM_021074039.1,XM_021083673.1,XM_021075702.1,XM_003355124.2,XM_021090817.1,XM_021085108.1,XM_005656504.3 |
| GO:0060089 | molecular transducer activity | 0.9978 | XM_001927733.6,XM_021092351.1,XM_021083673.1,XM_021074039.1,XM_003355124.2,XM_021075702.1,XM_005656504.3,XM_021085108.1,XM_021090817.1 |
| GO:0004930 | G-protein coupled receptor activity | 0.99851 | XM_021090817.1,XM_021085108.1,XM_021083673.1,XM_003355124.2,XM_021092351.1,XM_001927733.6 |
| GO:0007186 | G-protein coupled receptor signaling pathway | 0.99947 | XM_001927733.6,XM_021092351.1,XM_003355124.2,XM_021083673.1,XM_021085108.1,XM_021090817.1 |

**Table S8.** KEGG analysis of the selected genes Anhui pig population versus Asian wild boar

| #Term | ID | P-Value | Input |
| --- | --- | --- | --- |
| Chronic myeloid leukemia | ssc05220 | 0.005917408 | XM_021071922.1|NM_001285967.1|XM_003132994.4|XM_021071138.1|XM_021101439.1 |
| Endometrial cancer | ssc05213 | 0.009234192 | XM_021071922.1|XM_021074380.1|XM_021101439.1|XM_021088199.1 |
| Bile secretion | ssc04976 | 0.027004717 | NM_001164021.1|XM_021091087.1|XM_013989320.2|XM_021096361.1 |
| Adherens junction | ssc04520 | 0.029446245 | XM_021071922.1|XM_021079051.1|XM_021088199.1|XM_021074380.1 |
| Bacterial invasion of epithelial cells | ssc05100 | 0.0320144 | XM_003132994.4|XM_021079051.1|XM_021088199.1|XM_021074380.1 |
| Fanconi anemia pathway | ssc03460 | 0.051637372 | XM_021072739.1|XM_021091238.1|XM_021088208.1 |
| Non-small cell lung cancer | ssc05223 | 0.054010745 | XM_021071922.1|NM_001285967.1|XM_021101439.1 |
| Dorso-ventral axis formation | ssc04320 | 0.057140414 | XM_021071922.1|XM_021101439.1 |
| Gap junction | ssc04540 | 0.059003762 | XM_021074050.1|XM_021071922.1|XM_021096361.1|XM_021101439.1 |
| Chemokine signaling pathway | ssc04062 | 0.061326008 | XM_021071922.1|XM_021101439.1|XM_021082806.1|XM_003132994.4|XM_021096361.1|XM_021079051.1 |
| Platelet activation | ssc04611 | 0.063010983 | XM_021096361.1|NM_001105289.1|XM_021071922.1|XM_021062982.1|NM_001243297.1 |
| Inositol phosphate metabolism | ssc00562 | 0.066638907 | XM_013988351.2|XM_021100671.1|XM_001929569.6 |
| Glioma | ssc05214 | 0.077618795 | XM_021071922.1|NM_001285967.1|XM_021101439.1 |
| Renal cell carcinoma | ssc05211 | 0.086337983 | XM_021071922.1|XM_003132994.4|XM_021101439.1 |
| Prolactin signaling pathway | ssc04917 | 0.095451158 | XM_021082806.1|XM_021071922.1|XM_021101439.1 |
| Long-term potentiation | ssc04720 | 0.098573023 | XM_021071922.1|XM_021096361.1|XM_005656504.3 |
| Taste transduction | ssc04742 | 0.102559902 | XM_021092351.1|XM_021096361.1 |
| MAPK signaling pathway | ssc04010 | 0.105674932 | XM_021071922.1|XM_021101439.1|XM_013986853.2|XM_003132994.4|XM_021096361.1|XM_021071138.1|XM_021101533.1 |
| Prion diseases | ssc05020 | 0.107522301 | XM_021071922.1|XM_021096361.1 |
| Bladder cancer | ssc05219 | 0.112547469 | XM_021071922.1|NM_001285967.1 |
| Neurotrophin signaling pathway | ssc04722 | 0.122645382 | XM_021071922.1|XM_003132994.4|XM_021101439.1|XM_021091960.1 |
| Phosphatidylinositol signaling system | ssc04070 | 0.135438892 | XM_013988351.2|XM_021100671.1|XM_001929569.6 |
| Pathways in cancer | ssc05200 | 0.138992943 | NM_001285967.1|XM_021101439.1|XM_021071922.1|XM_021088199.1|XM_003132994.4|XM_021071138.1|XM_021073445.1|XM_021074380.1 |
| Protein digestion and absorption | ssc04974 | 0.138999671 | XM_021078736.1|NM_001105289.1|NM_001243297.1 |
| Fc gamma R-mediated phagocytosis | ssc04666 | 0.142591931 | XM_021071922.1|XM_003132994.4|XM_021079051.1 |
| Prostate cancer | ssc05215 | 0.157260013 | XM_021071922.1|NM_001285967.1|XM_021101439.1 |
| ErbB signaling pathway | ssc04012 | 0.157260013 | XM_021071922.1|XM_003132994.4|XM_021101439.1 |
| GnRH signaling pathway | ssc04912 | 0.160997862 | XM_021071922.1|XM_021096361.1|XM_021101439.1 |
| Insulin signaling pathway | ssc04910 | 0.165096437 | XM_021071922.1|XM_003132994.4|XM_021096361.1|XM_021101439.1 |
| Cocaine addiction | ssc05030 | 0.171126553 | XM_021096361.1|XM_005656504.3 |
| Retrograde endocannabinoid signaling | ssc04723 | 0.195750123 | XM_021071922.1|XM_021096361.1|XM_005656504.3 |
| Focal adhesion | ssc04510 | 0.198636742 | XM_021071922.1|XM_003132994.4|NM_001105289.1|XM_021101439.1|NM_001243297.1 |
| Estrogen signaling pathway | ssc04915 | 0.203710971 | XM_021071922.1|XM_021096361.1|XM_021101439.1 |
| Circadian entrainment | ssc04713 | 0.20771937 | XM_021071922.1|XM_021096361.1|XM_005656504.3 |
| Regulation of actin cytoskeleton | ssc04810 | 0.209571789 | XM_021071922.1|XM_003132994.4|XM_021079051.1|XM_021062982.1|XM_021101439.1 |
| Acute myeloid leukemia | ssc05221 | 0.21623666 | XM_021071922.1|XM_021101439.1 |
| Amoebiasis | ssc05146 | 0.244504476 | NM_001105289.1|XM_021096361.1|NM_001243297.1 |
| Long-term depression | ssc04730 | 0.244943393 | XM_021071922.1|XM_005656504.3 |
| One carbon pool by folate | ssc00670 | 0.257494184 | XM_021101430.1 |
| Pancreatic cancer | ssc05212 | 0.268014174 | XM_021071922.1|NM_001285967.1 |
| Fc epsilon RI signaling pathway | ssc04664 | 0.268014174 | XM_021071922.1|XM_021101439.1 |
| Leishmaniasis | ssc05140 | 0.268014174 | XM_021082806.1|XM_021071922.1 |
| Cholinergic synapse | ssc04725 | 0.269571363 | XM_021082806.1|XM_021071922.1|XM_021096361.1 |
| Thyroid hormone signaling pathway | ssc04919 | 0.269571363 | XM_021071922.1|XM_003126445.6|XM_021096361.1 |
| Amphetamine addiction | ssc05031 | 0.279551341 | XM_021096361.1|XM_005656504.3 |
| Vascular smooth muscle contraction | ssc04270 | 0.286440985 | XM_021071922.1|XM_021062982.1|XM_021096361.1 |
| Arrhythmogenic right ventricular cardiomyopathy (ARVC) | ssc05412 | 0.291075079 | XM_021088199.1|XM_021074380.1 |
| Glycosaminoglycan biosynthesis - chondroitin sulfate / dermatan sulfate | ssc00532 | 0.291606811 | XM_003135049.4 |
| Steroid biosynthesis | ssc00100 | 0.291606811 | NM_001167636.1 |
| Axon guidance | ssc04360 | 0.294908486 | XM_021073445.1|XM_021071922.1|XM_021062982.1 |
| Colorectal cancer | ssc05210 | 0.296828723 | XM_021073445.1|XM_021071922.1 |
| Melanoma | ssc05218 | 0.302575265 | XM_021071922.1|NM_001285967.1 |
| Proximal tubule bicarbonate reclamation | ssc04964 | 0.302626732 | XM_013989320.2 |
| Glutamatergic synapse | ssc04724 | 0.311885421 | XM_021071922.1|XM_021096361.1|XM_005656504.3 |
| Butanoate metabolism | ssc00650 | 0.313475688 | XM_001926818.5 |
| B cell receptor signaling pathway | ssc04662 | 0.319760799 | XM_021071922.1|XM_021101439.1 |
| Hepatitis C | ssc05160 | 0.328888494 | XM_021071922.1|XM_021101439.1|XM_001928339.4 |
| TGF-beta signaling pathway | ssc04350 | 0.331161914 | XM_021071922.1|XM_021100455.1 |
| Protein export | ssc03060 | 0.334671245 | XM_001925479.7 |
| Tight junction | ssc04530 | 0.337389153 | XM_021102528.1|XM_021088199.1|XM_021074380.1 |
| Pentose and glucuronate interconversions | ssc00040 | 0.345023016 | XM_021062686.1 |
| Collecting duct acid secretion | ssc04966 | 0.345023016 | XM_001928314.6 |
| Thyroid cancer | ssc05216 | 0.345023016 | XM_021071922.1 |
| Signaling pathways regulating pluripotency of stem cells | ssc04550 | 0.350127621 | XM_021082806.1|XM_021071922.1|XM_021100455.1 |
| Measles | ssc05162 | 0.354368488 | XM_021082806.1|XM_003125515.5|XM_001928339.4 |
| FoxO signaling pathway | ssc04068 | 0.354368488 | XM_021071922.1|XM_021068253.1|XM_021101439.1 |
| Ubiquitin mediated proteolysis | ssc04120 | 0.358605922 | XM_005670915.3|XM_021088208.1|XM_005664262.3 |
| Proteoglycans in cancer | ssc05205 | 0.363212179 | XM_021071922.1|XM_021062982.1|XM_021101439.1|XM_021096361.1 |
| Homologous recombination | ssc03440 | 0.365247163 | XM_021072739.1 |
| Salmonella infection | ssc05132 | 0.370591271 | XM_021071922.1|XM_021079051.1 |
| Fructose and mannose metabolism | ssc00051 | 0.375124473 | XM_021062686.1 |
| Hippo signaling pathway | ssc04390 | 0.375512219 | XM_021088199.1|XM_021074380.1|XM_021100455.1 |
| ECM-receptor interaction | ssc04512 | 0.37615223 | NM_001105289.1|NM_001243297.1 |
| Progesterone-mediated oocyte maturation | ssc04914 | 0.392710484 | XM_021071922.1|XM_021096361.1 |
| GABAergic synapse | ssc04727 | 0.392710484 | XM_021096361.1|XM_021075699.1 |
| cAMP signaling pathway | ssc04024 | 0.394857867 | XM_021076863.1|XM_021071922.1|XM_021096361.1|XM_005656504.3 |
| Pancreatic secretion | ssc04972 | 0.403638679 | XM_021078736.1|XM_013989320.2 |
| Nicotine addiction | ssc05033 | 0.413124414 | XM_005656504.3 |
| Rap1 signaling pathway | ssc04015 | 0.419307734 | XM_021071922.1|XM_021099431.1|XM_003132994.4|XM_021102528.1 |
| Morphine addiction | ssc05032 | 0.419852587 | XM_021076863.1|XM_021096361.1 |
| Apoptosis | ssc04210 | 0.425207388 | XM_021096361.1|XM_021091960.1 |
| SNARE interactions in vesicular transport | ssc04130 | 0.440104012 | XM_003132993.4 |
| Melanogenesis | ssc04916 | 0.446363837 | XM_021071922.1|XM_021096361.1 |
| Transcriptional misregulation in cancer | ssc05202 | 0.450110747 | XM_013993522.2|XM_021068253.1|XM_021100455.1 |
| Regulation of autophagy | ssc04140 | 0.457399617 | XM_001924990.5 |
| Aldosterone-regulated sodium reabsorption | ssc04960 | 0.482349064 | XM_021071922.1 |
| Mineral absorption | ssc04978 | 0.490408645 | NM_001164021.1 |
| Endocrine and other factor-regulated calcium reabsorption | ssc04961 | 0.490408645 | XM_021096361.1 |
| Carbohydrate digestion and absorption | ssc04973 | 0.490408645 | NM_001164021.1 |
| Influenza A | ssc05164 | 0.497799019 | XM_021082806.1|XM_021071922.1|XM_001928339.4 |
| Vasopressin-regulated water reabsorption | ssc04962 | 0.498343082 | XM_021096361.1 |
| Nucleotide excision repair | ssc03420 | 0.506154311 | XM_005673970.3 |
| T cell receptor signaling pathway | ssc04660 | 0.507089149 | XM_021071922.1|XM_021101439.1 |
| Oocyte meiosis | ssc04114 | 0.507089149 | XM_021071922.1|XM_021096361.1 |
| Natural killer cell mediated cytotoxicity | ssc04650 | 0.511951828 | XM_021071922.1|XM_021101439.1 |
| Sphingolipid metabolism | ssc00600 | 0.513844242 | XM_013981430.2 |
| TNF signaling pathway | ssc04668 | 0.516782913 | XM_021071922.1|XM_021102528.1 |
| Tuberculosis | ssc05152 | 0.520853566 | XM_021082806.1|XM_021071922.1|XM_021062982.1 |
| Proteasome | ssc03050 | 0.521414754 | XM_013984188.2 |
| Hedgehog signaling pathway | ssc04340 | 0.528867694 | XM_021096361.1 |
| Serotonergic synapse | ssc04726 | 0.531084723 | XM_021071922.1|XM_021096361.1 |
| Type II diabetes mellitus | ssc04930 | 0.536204885 | XM_021071922.1 |
| Amino sugar and nucleotide sugar metabolism | ssc00520 | 0.543428119 | XM_003126433.5 |
| Lysosome | ssc04142 | 0.545095974 | NM_001243516.1|NM_001244932.1 |
| Toxoplasmosis | ssc05145 | 0.545095974 | XM_021082806.1|XM_021071922.1 |
| NOD-like receptor signaling pathway | ssc04621 | 0.550539161 | XM_021071922.1 |
| Lysine degradation | ssc00310 | 0.550539161 | XM_013981913.2 |
| Calcium signaling pathway | ssc04020 | 0.550676238 | XM_021074039.1|XM_001927733.6|XM_021096361.1 |
| Purine metabolism | ssc00230 | 0.554326746 | XM_021100595.1|XM_021076863.1|XM_003359240.4 |
| Ovarian steroidogenesis | ssc04913 | 0.557539749 | XM_021096361.1 |
| Leukocyte transendothelial migration | ssc04670 | 0.56331971 | XM_021088199.1|XM_021074380.1 |
| Dopaminergic synapse | ssc04728 | 0.56779323 | XM_021096361.1|XM_005656504.3 |
| Viral carcinogenesis | ssc05203 | 0.575854761 | XM_021071922.1|NM_001285967.1|XM_021096361.1 |
| Epstein-Barr virus infection | ssc05169 | 0.575854761 | XM_013984188.2|NM_001285967.1|XM_021096361.1 |
| Amyotrophic lateral sclerosis (ALS) | ssc05014 | 0.584471388 | XM_005656504.3 |
| PI3K-Akt signaling pathway | ssc04151 | 0.592690851 | XM_021082806.1|XM_021071922.1|NM_001105289.1|XM_021101439.1|NM_001243297.1 |
| VEGF signaling pathway | ssc04370 | 0.6035916 | XM_021071922.1 |
| Synaptic vesicle cycle | ssc04721 | 0.609767983 | XM_001928314.6 |
| Neuroactive ligand-receptor interaction | ssc04080 | 0.612712519 | XM_021074039.1|XM_001927733.6|XM_005656504.3|XM_021100487.1 |
| mTOR signaling pathway | ssc04150 | 0.615848393 | XM_021071922.1 |
| Adrenergic signaling in cardiomyocytes | ssc04261 | 0.654069249 | XM_021071922.1|XM_021096361.1 |
| Alcoholism | ssc05034 | 0.661527915 | XM_021071922.1|XM_021101439.1 |
| Adipocytokine signaling pathway | ssc04920 | 0.671690066 | XM_021082806.1 |
| Cell adhesion molecules (CAMs) | ssc04514 | 0.672473465 | XM_021083730.1|XM_021099082.1 |
| Phagosome | ssc04145 | 0.676057765 | XM_021074050.1|XM_001928314.6 |
| Thyroid hormone synthesis | ssc04918 | 0.681846457 | XM_021096361.1 |
| RNA transport | ssc03013 | 0.683130751 | XM_003125515.5|XM_001928339.4 |
| Ras signaling pathway | ssc04014 | 0.684962732 | XM_021071922.1|XM_021096361.1|XM_021101439.1 |
| Complement and coagulation cascades | ssc04610 | 0.686806535 | XM_001929566.5 |
| Pertussis | ssc05133 | 0.686806535 | XM_021071922.1 |
| Hepatitis B | ssc05161 | 0.69007709 | XM_021071922.1|NM_001285967.1 |
| Gastric acid secretion | ssc04971 | 0.691689493 | XM_021096361.1 |
| Salivary secretion | ssc04970 | 0.696496527 | XM_021096361.1 |
| Jak-STAT signaling pathway | ssc04630 | 0.696897842 | XM_021082806.1|XM_021101439.1 |
| Oxytocin signaling pathway | ssc04921 | 0.722948024 | XM_021071922.1|XM_021096361.1 |
| Protein processing in endoplasmic reticulum | ssc04141 | 0.726068135 | XM_001925479.7|XM_001928339.4 |
| Glycerophospholipid metabolism | ssc00564 | 0.732357074 | XM_021092211.1 |
| Dilated cardiomyopathy | ssc05414 | 0.736531648 | XM_021096361.1 |
| Insulin secretion | ssc04911 | 0.744686992 | XM_021096361.1 |
| Rheumatoid arthritis | ssc05323 | 0.748669761 | XM_001928314.6 |
| Small cell lung cancer | ssc05222 | 0.752590568 | NM_001285967.1 |
| Herpes simplex infection | ssc05168 | 0.782461267 | XM_021082806.1|XM_001928339.4 |
| Pyrimidine metabolism | ssc00240 | 0.798340361 | XM_021100595.1 |
| Toll-like receptor signaling pathway | ssc04620 | 0.798340361 | XM_021071922.1 |
| HIF-1 signaling pathway | ssc04066 | 0.807637583 | XM_021071922.1 |
| Chagas disease (American trypanosomiasis) | ssc05142 | 0.819372278 | XM_021071922.1 |
| Inflammatory mediator regulation of TRP channels | ssc04750 | 0.824969101 | XM_021096361.1 |
| Cell cycle | ssc04110 | 0.838216083 | NM_001285967.1 |
| AMPK signaling pathway | ssc04152 | 0.845679304 | XM_021075700.1 |
| Wnt signaling pathway | ssc04310 | 0.868163895 | XM_021096361.1 |
| MicroRNAs in cancer | ssc05206 | 0.875057228 | XM_003132994.4|XM_021101439.1 |
| Osteoclast differentiation | ssc04380 | 0.878149206 | XM_021071922.1 |
| Oxidative phosphorylation | ssc00190 | 0.880053861 | XM_001928314.6 |
| Metabolic pathways | ssc01100 | 0.882086214 | XM_013988351.2|XM_021062686.1|XM_021100671.1|XM_021092211.1|XM_021100595.1|NM_001167636.1|XM_003126433.5|XM_021091238.1|XM_003359240.4|XM_001929569.6|XM_001928314.6|XM_021101430.1|XM_021075699.1|XM_013981430.2 |
| Parkinson's disease | ssc05012 | 0.90824591 | XM_021096361.1 |
| HTLV-I infection | ssc05166 | 0.908624374 | NM_001285967.1|XM_021096361.1 |
| Non-alcoholic fatty liver disease (NAFLD) | ssc04932 | 0.920386276 | XM_001928339.4 |
| cGMP-PKG signaling pathway | ssc04022 | 0.928709601 | XM_021071922.1 |
| Alzheimer's disease | ssc05010 | 0.93716446 | XM_021071922.1 |
| Olfactory transduction | ssc04740 | 0.999965921 | XM_021085108.1|XM_021096361.1 |

**Table S9.** The selection regions of Anhui pig population versus commercial pig population

| CHROM | BIN_START | BIN_END | log2(θπ ratio) | Fst |
| --- | --- | --- | --- | --- |
| 1 | 231840001 | 231880000 | 0.002054302 | 0.252795 |
| 1 | 242020001 | 242060000 | 0.020214606 | 0.252869 |
| 1 | 41580001 | 41620000 | 0.674454384 | 0.253127 |
| 1 | 217100001 | 217140000 | 0.484238642 | 0.253418 |
| 1 | 47520001 | 47560000 | 0.104593388 | 0.253784 |
| 1 | 111960001 | 112000000 | 0.047619321 | 0.253965 |
| 1 | 242380001 | 242420000 | 0.18929034 | 0.254016 |
| 1 | 112960001 | 113000000 | 0.091269147 | 0.254489 |
| 1 | 117720001 | 117760000 | 0.105794449 | 0.255602 |
| 1 | 55400001 | 55440000 | 0.018781304 | 0.255635 |
| 1 | 208940001 | 208980000 | 0.077460302 | 0.255951 |
| 1 | 55280001 | 55320000 | 0.054161368 | 0.256062 |
| 1 | 55760001 | 55800000 | 0.062055292 | 0.256095 |
| 1 | 217060001 | 217100000 | 0.979600507 | 0.256367 |
| 1 | 149160001 | 149200000 | 0.307697697 | 0.256581 |
| 1 | 208900001 | 208940000 | 0.552588718 | 0.256581 |
| 1 | 94980001 | 95020000 | 0.144012368 | 0.256708 |
| 1 | 95000001 | 95040000 | 0.015570784 | 0.25741 |
| 1 | 112060001 | 112100000 | 0.009748683 | 0.258142 |
| 1 | 93340001 | 93380000 | 0.082643534 | 0.258887 |
| 1 | 41720001 | 41760000 | 0.96320546 | 0.259929 |
| 1 | 74560001 | 74600000 | 0.488687934 | 0.260245 |
| 1 | 58820001 | 58860000 | 0.047170904 | 0.260695 |
| 1 | 217040001 | 217080000 | 0.985626617 | 0.261408 |
| 1 | 231820001 | 231860000 | 0.119893389 | 0.262708 |
| 1 | 74540001 | 74580000 | 0.476216495 | 0.262853 |
| 1 | 50200001 | 50240000 | 0.074160765 | 0.263287 |
| 1 | 225320001 | 225360000 | 0.126714068 | 0.263414 |
| 1 | 41520001 | 41560000 | 0.122211554 | 0.264297 |
| 1 | 220360001 | 220400000 | 0.157158953 | 0.264582 |
| 1 | 221440001 | 221480000 | 0.142565172 | 0.265495 |
| 1 | 36280001 | 36320000 | 0.199443511 | 0.2656 |
| 1 | 257000001 | 257040000 | 0.16862367 | 0.265765 |
| 1 | 242100001 | 242140000 | 0.182890195 | 0.266007 |
| 1 | 73840001 | 73880000 | 0.202130945 | 0.266052 |
| 1 | 220280001 | 220320000 | 0.053497736 | 0.266135 |
| 1 | 235800001 | 235840000 | 0.39385764 | 0.26628 |
| 1 | 105280001 | 105320000 | 0.13617474 | 0.26681 |
| 1 | 38180001 | 38220000 | 0.013953182 | 0.267334 |
| 1 | 55260001 | 55300000 | 0.191916429 | 0.268279 |
| 1 | 86600001 | 86640000 | 0.249961362 | 0.268498 |
| 1 | 134740001 | 134780000 | 0.288015849 | 0.268547 |
| 1 | 56500001 | 56540000 | 0.077007551 | 0.268637 |
| 1 | 219020001 | 219060000 | 0.235521499 | 0.269097 |
| 1 | 134460001 | 134500000 | 0.029579845 | 0.269265 |
| 1 | 93360001 | 93400000 | 0.072231915 | 0.269335 |
| 1 | 242400001 | 242440000 | 0.349532216 | 0.269519 |
| 1 | 134720001 | 134760000 | 0.307533496 | 0.271014 |
| 1 | 233120001 | 233160000 | 0.038651448 | 0.27285 |
| 1 | 36220001 | 36260000 | 0.444754803 | 0.273093 |
| 1 | 65740001 | 65780000 | 0.194237887 | 0.27323 |
| 1 | 112480001 | 112520000 | 0.338137726 | 0.273336 |
| 1 | 44640001 | 44680000 | 0.157316264 | 0.273496 |
| 1 | 208880001 | 208920000 | 0.102497071 | 0.274069 |
| 1 | 42500001 | 42540000 | 0.575046902 | 0.274174 |
| 1 | 94320001 | 94360000 | 0.534844887 | 0.274265 |
| 1 | 36240001 | 36280000 | 0.674987475 | 0.274398 |
| 1 | 117100001 | 117140000 | 0.061801314 | 0.27479 |
| 1 | 149720001 | 149760000 | 0.0691548 | 0.275906 |
| 1 | 209100001 | 209140000 | 0.64050267 | 0.276576 |
| 1 | 74040001 | 74080000 | 0.27473083 | 0.277039 |
| 1 | 166160001 | 166200000 | 0.132474478 | 0.277101 |
| 1 | 112040001 | 112080000 | 0.442128197 | 0.277995 |
| 1 | 149700001 | 149740000 | 0.017093775 | 0.278001 |
| 1 | 232120001 | 232160000 | 0.233438867 | 0.27862 |
| 1 | 138820001 | 138860000 | 0.451147111 | 0.278916 |
| 1 | 62880001 | 62920000 | 0.083697995 | 0.279505 |
| 1 | 42540001 | 42580000 | 0.501190437 | 0.27998 |
| 1 | 95440001 | 95480000 | 0.12709021 | 0.280161 |
| 1 | 125940001 | 125980000 | 0.032589104 | 0.280522 |
| 1 | 60000001 | 60040000 | 0.613171701 | 0.280778 |
| 1 | 62040001 | 62080000 | 0.048665724 | 0.28089 |
| 1 | 38200001 | 38240000 | 0.399934741 | 0.28102 |
| 1 | 25280001 | 25320000 | 0.367816064 | 0.281565 |
| 1 | 93300001 | 93340000 | 0.04957821 | 0.28187 |
| 1 | 106660001 | 106700000 | 0.016558152 | 0.281972 |
| 1 | 217440001 | 217480000 | 0.169159784 | 0.282317 |
| 1 | 45200001 | 45240000 | 0.110830468 | 0.282531 |
| 1 | 106680001 | 106720000 | 0.214951407 | 0.283098 |
| 1 | 256940001 | 256980000 | 0.440891965 | 0.28441 |
| 1 | 146760001 | 146800000 | 0.121391487 | 0.284923 |
| 1 | 221120001 | 221160000 | 0.389905296 | 0.285108 |
| 1 | 111720001 | 111760000 | 0.133796551 | 0.28511 |
| 1 | 44500001 | 44540000 | 0.243447555 | 0.286961 |
| 1 | 221140001 | 221180000 | 0.316791895 | 0.288212 |
| 1 | 232180001 | 232220000 | 0.090625148 | 0.288382 |
| 1 | 36160001 | 36200000 | 0.010788095 | 0.288895 |
| 1 | 139180001 | 139220000 | 0.063438643 | 0.289174 |
| 1 | 149080001 | 149120000 | 0.099950811 | 0.289325 |
| 1 | 125520001 | 125560000 | 0.102264139 | 0.28961 |
| 1 | 209040001 | 209080000 | 0.118388258 | 0.29203 |
| 1 | 232100001 | 232140000 | 0.049100317 | 0.292608 |
| 1 | 166040001 | 166080000 | 0.03925334 | 0.292917 |
| 1 | 256960001 | 257000000 | 0.432908889 | 0.293438 |
| 1 | 55460001 | 55500000 | 0.198019265 | 0.293635 |
| 1 | 110000001 | 110040000 | 0.15504899 | 0.293641 |
| 1 | 118040001 | 118080000 | 0.140875902 | 0.293809 |
| 1 | 233140001 | 233180000 | 0.039904296 | 0.294563 |
| 1 | 93180001 | 93220000 | 0.07972942 | 0.294585 |
| 1 | 108360001 | 108400000 | 0.158892581 | 0.295787 |
| 1 | 44620001 | 44660000 | 0.334645807 | 0.296413 |
| 1 | 151900001 | 151940000 | 0.193800431 | 0.29688 |
| 1 | 232840001 | 232880000 | 0.105002915 | 0.297405 |
| 1 | 55160001 | 55200000 | 0.108706925 | 0.29834 |
| 1 | 233100001 | 233140000 | 0.146938509 | 0.299256 |
| 1 | 256980001 | 257020000 | 0.409986592 | 0.301148 |
| 1 | 209060001 | 209100000 | 0.202257169 | 0.301849 |
| 1 | 242060001 | 242100000 | 0.162437729 | 0.304976 |
| 1 | 232160001 | 232200000 | 0.172741596 | 0.305111 |
| 1 | 110020001 | 110060000 | 0.233306559 | 0.305636 |
| 1 | 50180001 | 50220000 | 0.154144088 | 0.306615 |
| 1 | 112460001 | 112500000 | 0.202740125 | 0.308562 |
| 1 | 69840001 | 69880000 | 0.011451787 | 0.309198 |
| 1 | 36260001 | 36300000 | 0.545980231 | 0.309368 |
| 1 | 230840001 | 230880000 | 0.280996034 | 0.309426 |
| 1 | 209080001 | 209120000 | 0.303137473 | 0.310209 |
| 1 | 221520001 | 221560000 | 0.216194535 | 0.310567 |
| 1 | 44520001 | 44560000 | 0.192650607 | 0.311533 |
| 1 | 104940001 | 104980000 | 0.068005901 | 0.312444 |
| 1 | 25300001 | 25340000 | 0.373548513 | 0.314791 |
| 1 | 65800001 | 65840000 | 0.33541254 | 0.315789 |
| 1 | 56100001 | 56140000 | 0.013846699 | 0.315888 |
| 1 | 36200001 | 36240000 | 0.418339718 | 0.316246 |
| 1 | 42520001 | 42560000 | 0.677471446 | 0.316513 |
| 1 | 55700001 | 55740000 | 0.058526892 | 0.317321 |
| 1 | 104920001 | 104960000 | 0.133311661 | 0.317671 |
| 1 | 149140001 | 149180000 | 0.106011513 | 0.317989 |
| 1 | 56040001 | 56080000 | 0.354952122 | 0.318142 |
| 1 | 125960001 | 126000000 | 0.85622295 | 0.31993 |
| 1 | 232040001 | 232080000 | 0.322474338 | 0.320752 |
| 1 | 180860001 | 180900000 | 1.126687215 | 0.320817 |
| 1 | 232140001 | 232180000 | 0.435455409 | 0.321051 |
| 1 | 93280001 | 93320000 | 0.159943899 | 0.321881 |
| 1 | 55440001 | 55480000 | 0.015533843 | 0.324216 |
| 1 | 242040001 | 242080000 | 0.458162219 | 0.324362 |
| 1 | 112440001 | 112480000 | 0.230962859 | 0.324532 |
| 1 | 221540001 | 221580000 | 0.40088917 | 0.324708 |
| 1 | 232960001 | 233000000 | 0.059592222 | 0.325496 |
| 1 | 111700001 | 111740000 | 0.582273884 | 0.325554 |
| 1 | 43820001 | 43860000 | 0.083769048 | 0.326816 |
| 1 | 105300001 | 105340000 | 0.942972807 | 0.327916 |
| 1 | 93260001 | 93300000 | 0.143716035 | 0.328164 |
| 1 | 138840001 | 138880000 | 0.947296074 | 0.328903 |
| 1 | 105320001 | 105360000 | 0.387711011 | 0.329741 |
| 1 | 77360001 | 77400000 | 0.140411497 | 0.331619 |
| 1 | 221760001 | 221800000 | 0.561723556 | 0.332423 |
| 1 | 139160001 | 139200000 | 0.03954488 | 0.332877 |
| 1 | 104960001 | 105000000 | 0.009555173 | 0.333491 |
| 1 | 126000001 | 126040000 | 1.008889838 | 0.335375 |
| 1 | 151940001 | 151980000 | 0.114305602 | 0.335811 |
| 1 | 112420001 | 112460000 | 0.101568869 | 0.336968 |
| 1 | 73960001 | 74000000 | 0.129652705 | 0.338405 |
| 1 | 125980001 | 126020000 | 0.815494079 | 0.339088 |
| 1 | 53840001 | 53880000 | 0.168786786 | 0.339692 |
| 1 | 111680001 | 111720000 | 0.525432809 | 0.340408 |
| 1 | 90280001 | 90320000 | 0.147352722 | 0.342481 |
| 1 | 133100001 | 133140000 | 0.224804708 | 0.345085 |
| 1 | 209660001 | 209700000 | 0.060556586 | 0.346162 |
| 1 | 112400001 | 112440000 | 0.156811709 | 0.346687 |
| 1 | 232060001 | 232100000 | 0.348585013 | 0.347269 |
| 1 | 237740001 | 237780000 | 0.046004876 | 0.350921 |
| 1 | 220380001 | 220420000 | 0.215590505 | 0.352756 |
| 1 | 210840001 | 210880000 | 0.177555796 | 0.352852 |
| 1 | 210220001 | 210260000 | 0.061652987 | 0.353146 |
| 1 | 230820001 | 230860000 | 0.002058574 | 0.354216 |
| 1 | 74060001 | 74100000 | 0.245971701 | 0.355648 |
| 1 | 221740001 | 221780000 | 0.400801794 | 0.356834 |
| 1 | 232080001 | 232120000 | 0.235477594 | 0.357281 |
| 1 | 151920001 | 151960000 | 0.12229397 | 0.360736 |
| 1 | 69860001 | 69900000 | 0.18026691 | 0.362327 |
| 1 | 133120001 | 133160000 | 0.373215589 | 0.362656 |
| 1 | 133080001 | 133120000 | 0.148868428 | 0.362819 |
| 1 | 55960001 | 56000000 | 0.169918702 | 0.364832 |
| 1 | 64260001 | 64300000 | 0.254189553 | 0.366713 |
| 1 | 69880001 | 69920000 | 0.155088351 | 0.368021 |
| 1 | 221580001 | 221620000 | 0.197889772 | 0.368634 |
| 1 | 221560001 | 221600000 | 0.29787452 | 0.369311 |
| 1 | 56060001 | 56100000 | 0.158117656 | 0.370473 |
| 1 | 106920001 | 106960000 | 0.142243246 | 0.371666 |
| 1 | 40900001 | 40940000 | 0.081021179 | 0.371787 |
| 1 | 133160001 | 133200000 | 0.090987986 | 0.372881 |
| 1 | 90240001 | 90280000 | 0.085963467 | 0.376112 |
| 1 | 90180001 | 90220000 | 0.081006132 | 0.37673 |
| 1 | 111660001 | 111700000 | 0.105377655 | 0.376822 |
| 1 | 74480001 | 74520000 | 0.1512932 | 0.378636 |
| 1 | 90260001 | 90300000 | 0.264302336 | 0.379898 |
| 1 | 139140001 | 139180000 | 0.015964313 | 0.38155 |
| 1 | 104980001 | 105020000 | 0.074750427 | 0.383356 |
| 1 | 151880001 | 151920000 | 0.48404781 | 0.384101 |
| 1 | 86540001 | 86580000 | 0.105198313 | 0.385259 |
| 1 | 86520001 | 86560000 | 0.128425057 | 0.385389 |
| 1 | 209000001 | 209040000 | 0.063045052 | 0.386174 |
| 1 | 133140001 | 133180000 | 0.268828431 | 0.386772 |
| 1 | 55980001 | 56020000 | 0.264377931 | 0.386954 |
| 1 | 133060001 | 133100000 | 0.197542267 | 0.388238 |
| 1 | 151860001 | 151900000 | 0.247436645 | 0.390977 |
| 1 | 56020001 | 56060000 | 0.462432146 | 0.391387 |
| 1 | 210240001 | 210280000 | 0.207690201 | 0.391988 |
| 1 | 106800001 | 106840000 | 0.342095497 | 0.393159 |
| 1 | 106940001 | 106980000 | 0.083308901 | 0.395353 |
| 1 | 55940001 | 55980000 | 0.266126989 | 0.398372 |
| 1 | 101260001 | 101300000 | 0.290109895 | 0.398526 |
| 1 | 86640001 | 86680000 | 0.377641996 | 0.399557 |
| 1 | 108520001 | 108560000 | 0.018343739 | 0.400481 |
| 1 | 220180001 | 220220000 | 0.047394453 | 0.402969 |
| 1 | 101280001 | 101320000 | 0.394466752 | 0.404937 |
| 1 | 74460001 | 74500000 | 0.128336803 | 0.408254 |
| 1 | 77160001 | 77200000 | 0.052493723 | 0.411534 |
| 1 | 90220001 | 90260000 | 0.092337505 | 0.417109 |
| 1 | 56080001 | 56120000 | 0.241468548 | 0.418575 |
| 1 | 56000001 | 56040000 | 0.455423452 | 0.420038 |
| 1 | 64220001 | 64260000 | 0.102486207 | 0.423789 |
| 1 | 90200001 | 90240000 | 0.127775117 | 0.423803 |
| 1 | 36180001 | 36220000 | 0.60651707 | 0.427232 |
| 1 | 220400001 | 220440000 | 0.368082759 | 0.427913 |
| 1 | 212240001 | 212280000 | 0.106354076 | 0.430345 |
| 1 | 86620001 | 86660000 | 0.271011106 | 0.432712 |
| 1 | 135760001 | 135800000 | 0.086197304 | 0.432797 |
| 1 | 135740001 | 135780000 | 0.11393728 | 0.435953 |
| 1 | 212200001 | 212240000 | 0.128798178 | 0.440329 |
| 1 | 73980001 | 74020000 | 0.038770836 | 0.442559 |
| 1 | 77180001 | 77220000 | 0.009369305 | 0.442675 |
| 1 | 212220001 | 212260000 | 0.224134408 | 0.445394 |
| 1 | 60040001 | 60080000 | 0.163939669 | 0.446494 |
| 1 | 220440001 | 220480000 | 0.292278462 | 0.450344 |
| 1 | 60020001 | 60060000 | 0.685466197 | 0.454665 |
| 1 | 133040001 | 133080000 | 0.179944796 | 0.461429 |
| 1 | 220420001 | 220460000 | 0.630258185 | 0.463458 |
| 1 | 104900001 | 104940000 | 0.069402881 | 0.469605 |
| 1 | 137580001 | 137620000 | 0.025398798 | 0.505152 |
| 1 | 137480001 | 137520000 | 0.049754438 | 0.516847 |
| 1 | 137500001 | 137540000 | 0.132180323 | 0.521623 |
| 1 | 137560001 | 137600000 | 0.155612516 | 0.543903 |
| 1 | 132860001 | 132900000 | 0.044134403 | 0.54678 |
| 2 | 104320001 | 104360000 | 0.301640406 | 0.260012 |
| 2 | 9360001 | 9400000 | 0.642155496 | 0.264514 |
| 2 | 34380001 | 34420000 | 0.348924194 | 0.273749 |
| 2 | 34340001 | 34380000 | 0.295689826 | 0.277061 |
| 2 | 104280001 | 104320000 | 0.917363636 | 0.278511 |
| 2 | 104300001 | 104340000 | 0.804138082 | 0.28532 |
| 2 | 34360001 | 34400000 | 0.300216016 | 0.291592 |
| 2 | 9340001 | 9380000 | 0.283328145 | 0.298707 |
| 2 | 71840001 | 71880000 | 0.050429179 | 0.30048 |
| 2 | 34420001 | 34460000 | 0.462307436 | 0.306929 |
| 2 | 71860001 | 71900000 | 0.045129758 | 0.311799 |
| 2 | 151840001 | 151880000 | 0.001186306 | 0.320919 |
| 2 | 34400001 | 34440000 | 0.22338893 | 0.32489 |
| 2 | 151780001 | 151820000 | 0.220866759 | 0.336391 |
| 2 | 151800001 | 151840000 | 0.433849936 | 0.341373 |
| 2 | 151820001 | 151860000 | 0.253031421 | 0.348044 |
| 2 | 81420001 | 81460000 | 0.150968937 | 0.409939 |
| 2 | 81400001 | 81440000 | 0.124111406 | 0.412622 |
| 3 | 35280001 | 35320000 | 0.050875444 | 0.255733 |
| 3 | 46300001 | 46340000 | 0.515234196 | 0.256718 |
| 3 | 46320001 | 46360000 | 0.712991905 | 0.259006 |
| 3 | 43140001 | 43180000 | 0.275340803 | 0.259628 |
| 3 | 129000001 | 129040000 | 0.264864483 | 0.272471 |
| 3 | 56400001 | 56440000 | 0.207168571 | 0.28915 |
| 3 | 99520001 | 99560000 | 0.234624097 | 0.291339 |
| 3 | 25140001 | 25180000 | 0.046988892 | 0.291632 |
| 3 | 23100001 | 23140000 | 0.119595469 | 0.295316 |
| 3 | 52180001 | 52220000 | 0.118082612 | 0.301974 |
| 3 | 25160001 | 25200000 | 0.042129364 | 0.302887 |
| 3 | 50140001 | 50180000 | 0.190214775 | 0.317384 |
| 3 | 99500001 | 99540000 | 0.406608046 | 0.318637 |
| 3 | 99480001 | 99520000 | 0.522354402 | 0.322201 |
| 3 | 23160001 | 23200000 | 0.206891569 | 0.323119 |
| 3 | 43120001 | 43160000 | 0.008700598 | 0.332355 |
| 3 | 22660001 | 22700000 | 0.045744327 | 0.345188 |
| 3 | 23120001 | 23160000 | 0.210276183 | 0.348349 |
| 3 | 23140001 | 23180000 | 0.220101962 | 0.352862 |
| 3 | 128980001 | 129020000 | 0.363694776 | 0.358928 |
| 3 | 42120001 | 42160000 | 0.096621539 | 0.360479 |
| 3 | 42140001 | 42180000 | 0.173614763 | 0.367172 |
| 3 | 52200001 | 52240000 | 0.37182352 | 0.372693 |
| 3 | 42040001 | 42080000 | 0.262999901 | 0.374998 |
| 3 | 42200001 | 42240000 | 0.022989057 | 0.383473 |
| 3 | 42060001 | 42100000 | 0.189516625 | 0.384105 |
| 3 | 38580001 | 38620000 | 0.362198033 | 0.384345 |
| 3 | 38540001 | 38580000 | 0.337973292 | 0.387907 |
| 3 | 50120001 | 50160000 | 0.347395354 | 0.388103 |
| 3 | 42160001 | 42200000 | 0.062504507 | 0.391957 |
| 3 | 42180001 | 42220000 | 0.118118641 | 0.400577 |
| 3 | 38360001 | 38400000 | 0.587611403 | 0.413533 |
| 3 | 38280001 | 38320000 | 0.256929611 | 0.415674 |
| 3 | 38560001 | 38600000 | 0.235589679 | 0.416339 |
| 3 | 38300001 | 38340000 | 0.146315891 | 0.416457 |
| 3 | 22680001 | 22720000 | 0.171478509 | 0.41713 |
| 3 | 38320001 | 38360000 | 0.33237973 | 0.42403 |
| 3 | 38340001 | 38380000 | 0.834369963 | 0.430717 |
| 3 | 38380001 | 38420000 | 0.428969953 | 0.436246 |
| 3 | 38600001 | 38640000 | 0.226882945 | 0.439684 |
| 3 | 38460001 | 38500000 | 0.794580747 | 0.439754 |
| 3 | 38440001 | 38480000 | 0.953723383 | 0.444566 |
| 3 | 22700001 | 22740000 | 0.287696081 | 0.44762 |
| 3 | 38480001 | 38520000 | 0.366864425 | 0.449651 |
| 3 | 51920001 | 51960000 | 0.040820419 | 0.450826 |
| 3 | 51940001 | 51980000 | 0.027085257 | 0.460996 |
| 3 | 38620001 | 38660000 | 0.03164023 | 0.467267 |
| 3 | 38420001 | 38460000 | 1.03439757 | 0.468903 |
| 3 | 38400001 | 38440000 | 0.827185804 | 0.494828 |
| 3 | 83200001 | 83240000 | 0.328800628 | 0.497043 |
| 3 | 83220001 | 83260000 | 0.182727299 | 0.533978 |
| 4 | 52420001 | 52460000 | 0.103602662 | 0.254899 |
| 4 | 88620001 | 88660000 | 0.020818031 | 0.255925 |
| 4 | 124580001 | 124620000 | 0.007961437 | 0.25603 |
| 4 | 52220001 | 52260000 | 0.684335257 | 0.256502 |
| 4 | 79980001 | 80020000 | 0.185822825 | 0.257121 |
| 4 | 113880001 | 113920000 | 0.62415232 | 0.258031 |
| 4 | 80840001 | 80880000 | 0.435321855 | 0.259512 |
| 4 | 113900001 | 113940000 | 0.599346946 | 0.261478 |
| 4 | 113920001 | 113960000 | 0.207640885 | 0.264733 |
| 4 | 80000001 | 80040000 | 0.215374666 | 0.267006 |
| 4 | 118140001 | 118180000 | 0.177485566 | 0.274722 |
| 4 | 52240001 | 52280000 | 1.001932141 | 0.279687 |
| 4 | 88640001 | 88680000 | 0.202626786 | 0.280553 |
| 4 | 91420001 | 91460000 | 0.145296598 | 0.280932 |
| 4 | 91360001 | 91400000 | 0.013158586 | 0.282497 |
| 4 | 118160001 | 118200000 | 0.174235496 | 0.284186 |
| 4 | 52260001 | 52300000 | 1.474203481 | 0.284724 |
| 4 | 90140001 | 90180000 | 0.167801506 | 0.294127 |
| 4 | 88380001 | 88420000 | 0.124910269 | 0.300238 |
| 4 | 84720001 | 84760000 | 0.031403098 | 0.303727 |
| 4 | 91400001 | 91440000 | 0.505763802 | 0.311795 |
| 4 | 91900001 | 91940000 | 0.805409099 | 0.316367 |
| 4 | 97980001 | 98020000 | 0.024412037 | 0.329871 |
| 4 | 98000001 | 98040000 | 0.113174638 | 0.333098 |
| 4 | 91380001 | 91420000 | 0.619037363 | 0.337366 |
| 4 | 89320001 | 89360000 | 0.204778705 | 0.346912 |
| 4 | 98020001 | 98060000 | 0.278120433 | 0.355276 |
| 5 | 61120001 | 61160000 | 0.247175066 | 0.255901 |
| 5 | 29860001 | 29900000 | 0.230986506 | 0.25599 |
| 5 | 89660001 | 89700000 | 0.666925838 | 0.259479 |
| 5 | 29660001 | 29700000 | 0.108049673 | 0.261972 |
| 5 | 29580001 | 29620000 | 0.04414742 | 0.276984 |
| 5 | 16480001 | 16520000 | 0.056199138 | 0.280428 |
| 5 | 93920001 | 93960000 | 0.00204288 | 0.281923 |
| 5 | 61160001 | 61200000 | 0.91010708 | 0.291588 |
| 5 | 61140001 | 61180000 | 0.468500191 | 0.292133 |
| 5 | 49560001 | 49600000 | 0.454280996 | 0.306444 |
| 5 | 33560001 | 33600000 | 0.38773031 | 0.336093 |
| 5 | 16460001 | 16500000 | 0.234403857 | 0.340221 |
| 5 | 33540001 | 33580000 | 0.533992644 | 0.355336 |
| 5 | 89640001 | 89680000 | 0.660077822 | 0.359827 |
| 5 | 32180001 | 32220000 | 0.02389498 | 0.363383 |
| 5 | 89620001 | 89660000 | 0.139966802 | 0.472696 |
| 6 | 145620001 | 145660000 | 0.714993244 | 0.252785 |
| 6 | 129540001 | 129580000 | 0.827441648 | 0.256609 |
| 6 | 139220001 | 139260000 | 0.274401088 | 0.259962 |
| 6 | 24980001 | 25020000 | 0.207794643 | 0.264602 |
| 6 | 140800001 | 140840000 | 0.212489695 | 0.265303 |
| 6 | 47980001 | 48020000 | 0.751928055 | 0.266499 |
| 6 | 49180001 | 49220000 | 0.79853648 | 0.266657 |
| 6 | 122580001 | 122620000 | 0.009657143 | 0.267269 |
| 6 | 122820001 | 122860000 | 0.141565524 | 0.267492 |
| 6 | 123180001 | 123220000 | 0.02174603 | 0.269234 |
| 6 | 96280001 | 96320000 | 0.347599424 | 0.269287 |
| 6 | 132040001 | 132080000 | 0.28482327 | 0.271009 |
| 6 | 48020001 | 48060000 | 0.229037361 | 0.271565 |
| 6 | 129320001 | 129360000 | 0.496915442 | 0.276823 |
| 6 | 81740001 | 81780000 | 0.019527874 | 0.279758 |
| 6 | 90820001 | 90860000 | 0.125782408 | 0.279807 |
| 6 | 138180001 | 138220000 | 0.469499548 | 0.28125 |
| 6 | 137580001 | 137620000 | 0.106117449 | 0.281927 |
| 6 | 122560001 | 122600000 | 0.117654153 | 0.282584 |
| 6 | 69260001 | 69300000 | 0.712688949 | 0.283114 |
| 6 | 96220001 | 96260000 | 0.38731556 | 0.285692 |
| 6 | 129520001 | 129560000 | 0.848579272 | 0.287794 |
| 6 | 123200001 | 123240000 | 0.34558165 | 0.290066 |
| 6 | 138160001 | 138200000 | 0.374459058 | 0.290109 |
| 6 | 49100001 | 49140000 | 0.371155489 | 0.291264 |
| 6 | 140780001 | 140820000 | 0.266491921 | 0.29179 |
| 6 | 142780001 | 142820000 | 0.414681053 | 0.293584 |
| 6 | 125900001 | 125940000 | 0.330706016 | 0.294737 |
| 6 | 29540001 | 29580000 | 0.098847281 | 0.295761 |
| 6 | 53600001 | 53640000 | 0.383542032 | 0.29693 |
| 6 | 48000001 | 48040000 | 0.863589612 | 0.300445 |
| 6 | 131080001 | 131120000 | 0.34484029 | 0.30423 |
| 6 | 129340001 | 129380000 | 0.202196361 | 0.304866 |
| 6 | 29520001 | 29560000 | 0.129233994 | 0.307208 |
| 6 | 125920001 | 125960000 | 0.339054934 | 0.307465 |
| 6 | 53620001 | 53660000 | 0.075994736 | 0.310091 |
| 6 | 81760001 | 81800000 | 0.057444863 | 0.310923 |
| 6 | 96200001 | 96240000 | 0.221719583 | 0.312482 |
| 6 | 138840001 | 138880000 | 0.298020656 | 0.313675 |
| 6 | 49200001 | 49240000 | 0.812763687 | 0.314794 |
| 6 | 69220001 | 69260000 | 0.643786938 | 0.316433 |
| 6 | 69240001 | 69280000 | 1.022200965 | 0.318947 |
| 6 | 96300001 | 96340000 | 0.40505258 | 0.335128 |
| 6 | 81000001 | 81040000 | 0.503719215 | 0.335128 |
| 6 | 37740001 | 37780000 | 0.099250222 | 0.335939 |
| 6 | 81020001 | 81060000 | 0.298538608 | 0.346578 |
| 6 | 96320001 | 96360000 | 0.514416207 | 0.350014 |
| 6 | 49220001 | 49260000 | 0.511876317 | 0.350462 |
| 6 | 81920001 | 81960000 | 0.087464347 | 0.350961 |
| 6 | 53580001 | 53620000 | 0.213934606 | 0.358207 |
| 6 | 37700001 | 37740000 | 0.149394354 | 0.376452 |
| 6 | 137560001 | 137600000 | 0.341041389 | 0.378415 |
| 6 | 49080001 | 49120000 | 0.285582969 | 0.387798 |
| 6 | 37760001 | 37800000 | 0.242126242 | 0.393801 |
| 6 | 81680001 | 81720000 | 0.070468245 | 0.404262 |
| 6 | 49700001 | 49740000 | 0.060583046 | 0.405388 |
| 6 | 37780001 | 37820000 | 0.146804631 | 0.412504 |
| 6 | 28720001 | 28760000 | 0.164481654 | 0.54463 |
| 6 | 28740001 | 28780000 | 0.218368715 | 0.561053 |
| 7 | 103340001 | 103380000 | 0.268528695 | 0.252785 |
| 7 | 32120001 | 32160000 | 0.179380374 | 0.255474 |
| 7 | 90800001 | 90840000 | 0.436883353 | 0.255524 |
| 7 | 90200001 | 90240000 | 0.19190737 | 0.255753 |
| 7 | 90960001 | 91000000 | 0.427499914 | 0.25583 |
| 7 | 106940001 | 106980000 | 0.149956223 | 0.256835 |
| 7 | 103080001 | 103120000 | 0.034069961 | 0.258491 |
| 7 | 32080001 | 32120000 | 0.015222197 | 0.258693 |
| 7 | 30600001 | 30640000 | 0.498311927 | 0.260808 |
| 7 | 102960001 | 103000000 | 0.271559011 | 0.266679 |
| 7 | 103000001 | 103040000 | 0.282432293 | 0.267469 |
| 7 | 105200001 | 105240000 | 0.059051703 | 0.2687 |
| 7 | 90380001 | 90420000 | 0.123286789 | 0.270584 |
| 7 | 90940001 | 90980000 | 0.526925113 | 0.27183 |
| 7 | 102980001 | 103020000 | 0.252647847 | 0.273744 |
| 7 | 90680001 | 90720000 | 0.01875745 | 0.274017 |
| 7 | 91040001 | 91080000 | 0.523292702 | 0.2746 |
| 7 | 90060001 | 90100000 | 0.101859534 | 0.276357 |
| 7 | 30620001 | 30660000 | 0.56765994 | 0.277145 |
| 7 | 100920001 | 100960000 | 0.234786091 | 0.27993 |
| 7 | 91120001 | 91160000 | 0.507410493 | 0.28087 |
| 7 | 91000001 | 91040000 | 0.686345503 | 0.281701 |
| 7 | 32100001 | 32140000 | 0.15173256 | 0.282059 |
| 7 | 90600001 | 90640000 | 0.189101323 | 0.28556 |
| 7 | 106980001 | 107020000 | 0.06844327 | 0.289751 |
| 7 | 90340001 | 90380000 | 0.312119392 | 0.291552 |
| 7 | 106960001 | 107000000 | 0.00464172 | 0.296735 |
| 7 | 90640001 | 90680000 | 0.043633981 | 0.302729 |
| 7 | 90320001 | 90360000 | 0.299394915 | 0.304079 |
| 7 | 90660001 | 90700000 | 0.013715763 | 0.305812 |
| 7 | 90280001 | 90320000 | 0.342700709 | 0.306168 |
| 7 | 90620001 | 90660000 | 0.109851714 | 0.306384 |
| 7 | 90360001 | 90400000 | 0.398238223 | 0.30742 |
| 7 | 90240001 | 90280000 | 0.131371223 | 0.310439 |
| 7 | 103220001 | 103260000 | 0.519084128 | 0.313593 |
| 7 | 90300001 | 90340000 | 0.464597546 | 0.314505 |
| 7 | 90980001 | 91020000 | 0.885358419 | 0.315039 |
| 7 | 90260001 | 90300000 | 0.344255894 | 0.327098 |
| 7 | 91080001 | 91120000 | 0.731528493 | 0.335604 |
| 7 | 91100001 | 91140000 | 0.713840989 | 0.353824 |
| 7 | 91060001 | 91100000 | 0.931966234 | 0.354319 |
| 7 | 30760001 | 30800000 | 0.438223322 | 0.365189 |
| 7 | 300001 | 340000 | 0.124319231 | 0.374986 |
| 7 | 30780001 | 30820000 | 0.135523422 | 0.377931 |
| 7 | 103440001 | 103480000 | 0.077306757 | 0.400793 |
| 7 | 103320001 | 103360000 | 0.359147899 | 0.421262 |
| 7 | 103240001 | 103280000 | 0.924871985 | 0.428918 |
| 7 | 103260001 | 103300000 | 0.46476544 | 0.457271 |
| 7 | 103280001 | 103320000 | 0.405498845 | 0.483758 |
| 7 | 103300001 | 103340000 | 0.534417005 | 0.487337 |
| 8 | 98360001 | 98400000 | 0.290830085 | 0.253109 |
| 8 | 58460001 | 58500000 | 0.002505129 | 0.253988 |
| 8 | 36600001 | 36640000 | 0.481781279 | 0.254043 |
| 8 | 82520001 | 82560000 | 0.159380034 | 0.254346 |
| 8 | 85220001 | 85260000 | 0.123778867 | 0.254784 |
| 8 | 36940001 | 36980000 | 0.016981244 | 0.25498 |
| 8 | 62360001 | 62400000 | 0.010243251 | 0.255541 |
| 8 | 82800001 | 82840000 | 0.051003765 | 0.255611 |
| 8 | 102480001 | 102520000 | 0.795413244 | 0.256373 |
| 8 | 36620001 | 36660000 | 0.481942478 | 0.256387 |
| 8 | 82900001 | 82940000 | 0.027920094 | 0.256396 |
| 8 | 67300001 | 67340000 | 0.226089231 | 0.256556 |
| 8 | 36720001 | 36760000 | 0.27826116 | 0.256772 |
| 8 | 102540001 | 102580000 | 1.034604048 | 0.256839 |
| 8 | 79460001 | 79500000 | 0.334029028 | 0.256874 |
| 8 | 30960001 | 31000000 | 0.279477307 | 0.256919 |
| 8 | 102900001 | 102940000 | 0.763970651 | 0.256974 |
| 8 | 102500001 | 102540000 | 0.841868418 | 0.257114 |
| 8 | 79580001 | 79620000 | 0.509471825 | 0.25838 |
| 8 | 98420001 | 98460000 | 0.462179115 | 0.258947 |
| 8 | 99640001 | 99680000 | 0.149425767 | 0.260156 |
| 8 | 67540001 | 67580000 | 1.519294108 | 0.260399 |
| 8 | 104720001 | 104760000 | 0.077377731 | 0.260593 |
| 8 | 79600001 | 79640000 | 0.070957333 | 0.261228 |
| 8 | 67280001 | 67320000 | 0.290594421 | 0.261365 |
| 8 | 89640001 | 89680000 | 0.411317817 | 0.263918 |
| 8 | 100760001 | 100800000 | 0.141207886 | 0.264173 |
| 8 | 82540001 | 82580000 | 0.056165926 | 0.264969 |
| 8 | 49040001 | 49080000 | 0.174197566 | 0.266572 |
| 8 | 54720001 | 54760000 | 0.125606884 | 0.267044 |
| 8 | 68100001 | 68140000 | 0.384688665 | 0.267402 |
| 8 | 37860001 | 37900000 | 0.254951067 | 0.267935 |
| 8 | 101960001 | 102000000 | 0.293462113 | 0.267972 |
| 8 | 67340001 | 67380000 | 0.281465266 | 0.268596 |
| 8 | 41760001 | 41800000 | 0.1646059 | 0.269255 |
| 8 | 54740001 | 54780000 | 0.369149607 | 0.269389 |
| 8 | 64220001 | 64260000 | 0.376082167 | 0.269411 |
| 8 | 68060001 | 68100000 | 0.948214013 | 0.269606 |
| 8 | 67260001 | 67300000 | 0.452169506 | 0.269836 |
| 8 | 67000001 | 67040000 | 1.000757013 | 0.26986 |
| 8 | 67980001 | 68020000 | 0.714653237 | 0.269893 |
| 8 | 67320001 | 67360000 | 0.272919869 | 0.269932 |
| 8 | 61720001 | 61760000 | 0.597413757 | 0.270795 |
| 8 | 46860001 | 46900000 | 0.2538522 | 0.270998 |
| 8 | 100560001 | 100600000 | 0.78734756 | 0.270999 |
| 8 | 79040001 | 79080000 | 0.126586415 | 0.271227 |
| 8 | 61740001 | 61780000 | 0.523862686 | 0.271486 |
| 8 | 63360001 | 63400000 | 0.351691654 | 0.271566 |
| 8 | 98380001 | 98420000 | 0.548539778 | 0.272178 |
| 8 | 99220001 | 99260000 | 0.20496848 | 0.2734 |
| 8 | 30980001 | 31020000 | 0.302345808 | 0.27397 |
| 8 | 54900001 | 54940000 | 0.035344774 | 0.273999 |
| 8 | 79920001 | 79960000 | 0.114215313 | 0.274108 |
| 8 | 63340001 | 63380000 | 0.028061498 | 0.27445 |
| 8 | 89760001 | 89800000 | 0.365959084 | 0.274739 |
| 8 | 54940001 | 54980000 | 0.059245444 | 0.276476 |
| 8 | 49000001 | 49040000 | 0.521958983 | 0.276893 |
| 8 | 50500001 | 50540000 | 0.075665337 | 0.277348 |
| 8 | 31000001 | 31040000 | 0.300028368 | 0.277416 |
| 8 | 54920001 | 54960000 | 0.093618643 | 0.277478 |
| 8 | 57460001 | 57500000 | 0.353590107 | 0.278442 |
| 8 | 67140001 | 67180000 | 0.340625568 | 0.281478 |
| 8 | 87860001 | 87900000 | 0.211701986 | 0.281863 |
| 8 | 82280001 | 82320000 | 0.077790926 | 0.282263 |
| 8 | 31020001 | 31060000 | 0.385273141 | 0.282865 |
| 8 | 65340001 | 65380000 | 0.421546468 | 0.283357 |
| 8 | 64200001 | 64240000 | 0.098822106 | 0.2836 |
| 8 | 103740001 | 103780000 | 0.368712643 | 0.283781 |
| 8 | 36580001 | 36620000 | 0.380439637 | 0.283847 |
| 8 | 51080001 | 51120000 | 0.012324411 | 0.283961 |
| 8 | 74300001 | 74340000 | 0.214492028 | 0.284575 |
| 8 | 85200001 | 85240000 | 0.116011748 | 0.284644 |
| 8 | 100660001 | 100700000 | 0.350253668 | 0.284954 |
| 8 | 65280001 | 65320000 | 0.382066908 | 0.285576 |
| 8 | 80460001 | 80500000 | 0.48472371 | 0.285879 |
| 8 | 79400001 | 79440000 | 0.301726911 | 0.285897 |
| 8 | 48900001 | 48940000 | 0.344974199 | 0.286442 |
| 8 | 68040001 | 68080000 | 0.757846619 | 0.28661 |
| 8 | 36560001 | 36600000 | 0.068985856 | 0.286675 |
| 8 | 89700001 | 89740000 | 0.601895734 | 0.287371 |
| 8 | 72920001 | 72960000 | 0.29120358 | 0.289212 |
| 8 | 89680001 | 89720000 | 0.606867471 | 0.289312 |
| 8 | 104740001 | 104780000 | 0.31807816 | 0.289398 |
| 8 | 65320001 | 65360000 | 0.360719396 | 0.289437 |
| 8 | 46760001 | 46800000 | 0.24360875 | 0.290188 |
| 8 | 65240001 | 65280000 | 0.642651113 | 0.290326 |
| 8 | 89660001 | 89700000 | 0.440769106 | 0.290347 |
| 8 | 65260001 | 65300000 | 0.629685825 | 0.290635 |
| 8 | 102880001 | 102920000 | 0.38984089 | 0.291083 |
| 8 | 103640001 | 103680000 | 0.382525015 | 0.291148 |
| 8 | 48960001 | 49000000 | 0.355101688 | 0.291265 |
| 8 | 51600001 | 51640000 | 0.154034879 | 0.292894 |
| 8 | 58500001 | 58540000 | 0.120733896 | 0.293584 |
| 8 | 65300001 | 65340000 | 0.316379028 | 0.294405 |
| 8 | 49380001 | 49420000 | 0.080529578 | 0.295147 |
| 8 | 50540001 | 50580000 | 0.092532906 | 0.295184 |
| 8 | 39060001 | 39100000 | 0.001417282 | 0.295197 |
| 8 | 79440001 | 79480000 | 0.36756701 | 0.295724 |
| 8 | 65220001 | 65260000 | 0.513817139 | 0.295862 |
| 8 | 68080001 | 68120000 | 0.697394843 | 0.296555 |
| 8 | 45140001 | 45180000 | 0.673623274 | 0.296863 |
| 8 | 49360001 | 49400000 | 0.350941297 | 0.297067 |
| 8 | 50520001 | 50560000 | 0.142721724 | 0.297935 |
| 8 | 90320001 | 90360000 | 0.700026295 | 0.297944 |
| 8 | 33120001 | 33160000 | 0.119046616 | 0.298722 |
| 8 | 57440001 | 57480000 | 0.4111732 | 0.299221 |
| 8 | 35920001 | 35960000 | 0.169831701 | 0.299983 |
| 8 | 48940001 | 48980000 | 0.46875769 | 0.301272 |
| 8 | 49340001 | 49380000 | 0.112411722 | 0.302399 |
| 8 | 98400001 | 98440000 | 0.606721691 | 0.302569 |
| 8 | 68000001 | 68040000 | 0.637350317 | 0.303002 |
| 8 | 65460001 | 65500000 | 0.40834563 | 0.303189 |
| 8 | 30520001 | 30560000 | 0.187781938 | 0.303682 |
| 8 | 39080001 | 39120000 | 0.32278638 | 0.304447 |
| 8 | 104300001 | 104340000 | 0.517147868 | 0.305002 |
| 8 | 48980001 | 49020000 | 0.414759628 | 0.306929 |
| 8 | 51640001 | 51680000 | 0.191704195 | 0.307017 |
| 8 | 54880001 | 54920000 | 0.082527378 | 0.308047 |
| 8 | 84440001 | 84480000 | 0.260995803 | 0.309573 |
| 8 | 67080001 | 67120000 | 0.992069811 | 0.309751 |
| 8 | 40020001 | 40060000 | 0.217120685 | 0.309981 |
| 8 | 68020001 | 68060000 | 0.619258071 | 0.31012 |
| 8 | 54800001 | 54840000 | 0.025194152 | 0.310955 |
| 8 | 58480001 | 58520000 | 0.216064569 | 0.311072 |
| 8 | 69580001 | 69620000 | 0.226609038 | 0.311137 |
| 8 | 69600001 | 69640000 | 0.1273854 | 0.312034 |
| 8 | 100740001 | 100780000 | 0.471345508 | 0.312225 |
| 8 | 33100001 | 33140000 | 0.190062 | 0.312882 |
| 8 | 78780001 | 78820000 | 0.044411749 | 0.313688 |
| 8 | 87800001 | 87840000 | 0.080121844 | 0.314465 |
| 8 | 49220001 | 49260000 | 0.133255672 | 0.314517 |
| 8 | 51400001 | 51440000 | 0.175055454 | 0.3151 |
| 8 | 67060001 | 67100000 | 1.039312564 | 0.315155 |
| 8 | 51160001 | 51200000 | 0.420967025 | 0.315586 |
| 8 | 67020001 | 67060000 | 0.942604097 | 0.316197 |
| 8 | 67880001 | 67920000 | 0.944717502 | 0.316856 |
| 8 | 67100001 | 67140000 | 0.772313308 | 0.317771 |
| 8 | 104600001 | 104640000 | 1.104503289 | 0.318149 |
| 8 | 84460001 | 84500000 | 0.57433472 | 0.318825 |
| 8 | 85240001 | 85280000 | 0.188563162 | 0.319211 |
| 8 | 48920001 | 48960000 | 0.454251947 | 0.31995 |
| 8 | 67040001 | 67080000 | 0.974625695 | 0.320447 |
| 8 | 65200001 | 65240000 | 0.556696978 | 0.322778 |
| 8 | 95820001 | 95860000 | 0.473323155 | 0.322905 |
| 8 | 52680001 | 52720000 | 0.307977042 | 0.322955 |
| 8 | 79420001 | 79460000 | 0.221979459 | 0.323034 |
| 8 | 84480001 | 84520000 | 0.317672384 | 0.323602 |
| 8 | 51320001 | 51360000 | 0.309432277 | 0.325241 |
| 8 | 95840001 | 95880000 | 0.223571145 | 0.328245 |
| 8 | 78280001 | 78320000 | 0.113235671 | 0.328719 |
| 8 | 51500001 | 51540000 | 0.072766314 | 0.33012 |
| 8 | 87760001 | 87800000 | 0.423913845 | 0.330611 |
| 8 | 52300001 | 52340000 | 0.050178915 | 0.330901 |
| 8 | 51380001 | 51420000 | 0.094351215 | 0.332559 |
| 8 | 49180001 | 49220000 | 0.578048731 | 0.332894 |
| 8 | 80700001 | 80740000 | 0.101621583 | 0.333134 |
| 8 | 57420001 | 57460000 | 0.591118388 | 0.33389 |
| 8 | 51060001 | 51100000 | 0.107404387 | 0.334122 |
| 8 | 30580001 | 30620000 | 0.061565203 | 0.335151 |
| 8 | 51480001 | 51520000 | 0.088128894 | 0.336049 |
| 8 | 49940001 | 49980000 | 0.306933196 | 0.337048 |
| 8 | 104320001 | 104360000 | 0.358347166 | 0.337118 |
| 8 | 104620001 | 104660000 | 1.343244572 | 0.33893 |
| 8 | 49320001 | 49360000 | 0.244213666 | 0.341317 |
| 8 | 67120001 | 67160000 | 0.834468525 | 0.342084 |
| 8 | 87780001 | 87820000 | 0.407857152 | 0.342814 |
| 8 | 57400001 | 57440000 | 0.676129504 | 0.343227 |
| 8 | 51620001 | 51660000 | 0.126754681 | 0.343862 |
| 8 | 52660001 | 52700000 | 0.541542057 | 0.345469 |
| 8 | 65180001 | 65220000 | 0.388932276 | 0.34569 |
| 8 | 65360001 | 65400000 | 0.405159825 | 0.349196 |
| 8 | 78300001 | 78340000 | 0.125277575 | 0.352529 |
| 8 | 52700001 | 52740000 | 0.33117708 | 0.352747 |
| 8 | 65440001 | 65480000 | 0.401773858 | 0.353877 |
| 8 | 51000001 | 51040000 | 0.428782911 | 0.355808 |
| 8 | 103660001 | 103700000 | 1.040545881 | 0.356357 |
| 8 | 49160001 | 49200000 | 0.779149698 | 0.357477 |
| 8 | 65400001 | 65440000 | 0.232072789 | 0.358897 |
| 8 | 50880001 | 50920000 | 0.099458194 | 0.359626 |
| 8 | 46780001 | 46820000 | 0.524376296 | 0.360161 |
| 8 | 51420001 | 51460000 | 0.29622558 | 0.361507 |
| 8 | 65380001 | 65420000 | 0.230899209 | 0.362754 |
| 8 | 51140001 | 51180000 | 0.379956036 | 0.366045 |
| 8 | 79620001 | 79660000 | 0.207545924 | 0.366405 |
| 8 | 51580001 | 51620000 | 0.333207428 | 0.367278 |
| 8 | 80720001 | 80760000 | 0.141546396 | 0.367629 |
| 8 | 51520001 | 51560000 | 0.066718584 | 0.368188 |
| 8 | 51020001 | 51060000 | 0.406748755 | 0.368566 |
| 8 | 51100001 | 51140000 | 0.371612097 | 0.370539 |
| 8 | 46840001 | 46880000 | 0.516359659 | 0.37171 |
| 8 | 51040001 | 51080000 | 0.296188467 | 0.372003 |
| 8 | 46820001 | 46860000 | 0.528209983 | 0.37322 |
| 8 | 65420001 | 65460000 | 0.470202549 | 0.376266 |
| 8 | 49300001 | 49340000 | 0.553992902 | 0.378463 |
| 8 | 89920001 | 89960000 | 0.188043611 | 0.378848 |
| 8 | 49880001 | 49920000 | 0.217823154 | 0.379464 |
| 8 | 51460001 | 51500000 | 0.236783521 | 0.380052 |
| 8 | 52740001 | 52780000 | 0.40366588 | 0.380913 |
| 8 | 76380001 | 76420000 | 0.101653019 | 0.384198 |
| 8 | 85260001 | 85300000 | 0.07550442 | 0.384863 |
| 8 | 46800001 | 46840000 | 0.58540931 | 0.387107 |
| 8 | 30540001 | 30580000 | 0.454220264 | 0.388068 |
| 8 | 52640001 | 52680000 | 0.496484414 | 0.391217 |
| 8 | 30560001 | 30600000 | 0.429175381 | 0.391508 |
| 8 | 51540001 | 51580000 | 0.146796045 | 0.391584 |
| 8 | 51560001 | 51600000 | 0.307908016 | 0.394376 |
| 8 | 52540001 | 52580000 | 0.503294875 | 0.394929 |
| 8 | 89940001 | 89980000 | 0.374269933 | 0.396466 |
| 8 | 49240001 | 49280000 | 0.577327881 | 0.402521 |
| 8 | 51440001 | 51480000 | 0.435276134 | 0.4051 |
| 8 | 52360001 | 52400000 | 0.12276432 | 0.406071 |
| 8 | 52520001 | 52560000 | 0.41697922 | 0.406272 |
| 8 | 52720001 | 52760000 | 0.533979742 | 0.408889 |
| 8 | 39100001 | 39140000 | 0.199756121 | 0.409602 |
| 8 | 49280001 | 49320000 | 0.691552319 | 0.415237 |
| 8 | 52560001 | 52600000 | 0.628536048 | 0.415977 |
| 8 | 52580001 | 52620000 | 0.643609675 | 0.417919 |
| 8 | 51120001 | 51160000 | 0.467015843 | 0.418708 |
| 8 | 52620001 | 52660000 | 0.61123439 | 0.419583 |
| 8 | 52600001 | 52640000 | 0.655855784 | 0.419605 |
| 8 | 49920001 | 49960000 | 0.396669105 | 0.421374 |
| 8 | 76420001 | 76460000 | 0.599383216 | 0.422175 |
| 8 | 30620001 | 30660000 | 0.347656841 | 0.422218 |
| 8 | 76440001 | 76480000 | 0.370579704 | 0.422783 |
| 8 | 30600001 | 30640000 | 0.352514532 | 0.424368 |
| 8 | 49260001 | 49300000 | 0.667463176 | 0.433023 |
| 8 | 43820001 | 43860000 | 0.002667413 | 0.43351 |
| 8 | 85280001 | 85320000 | 0.085868734 | 0.436911 |
| 8 | 76400001 | 76440000 | 0.164571577 | 0.441778 |
| 8 | 85340001 | 85380000 | 0.067528011 | 0.441925 |
| 8 | 49900001 | 49940000 | 0.400359879 | 0.445222 |
| 8 | 45780001 | 45820000 | 0.149754461 | 0.45001 |
| 8 | 85320001 | 85360000 | 0.452774561 | 0.451772 |
| 8 | 74360001 | 74400000 | 0.474689947 | 0.457844 |
| 8 | 52420001 | 52460000 | 0.355794691 | 0.457995 |
| 8 | 52400001 | 52440000 | 0.131571829 | 0.458624 |
| 8 | 52500001 | 52540000 | 0.567511143 | 0.464284 |
| 8 | 38240001 | 38280000 | 0.132868861 | 0.469726 |
| 8 | 74320001 | 74360000 | 0.8966432 | 0.47049 |
| 8 | 52440001 | 52480000 | 0.522222318 | 0.472895 |
| 8 | 85300001 | 85340000 | 0.396144049 | 0.473524 |
| 8 | 38280001 | 38320000 | 0.250153269 | 0.476015 |
| 8 | 52380001 | 52420000 | 0.291745457 | 0.477822 |
| 8 | 52460001 | 52500000 | 0.471813009 | 0.479762 |
| 8 | 41440001 | 41480000 | 0.082003098 | 0.479818 |
| 8 | 52480001 | 52520000 | 0.653010977 | 0.486804 |
| 8 | 39120001 | 39160000 | 0.057904288 | 0.490436 |
| 8 | 43620001 | 43660000 | 0.190244931 | 0.493041 |
| 8 | 78380001 | 78420000 | 0.105624135 | 0.513622 |
| 8 | 41900001 | 41940000 | 0.085965844 | 0.5189 |
| 8 | 38260001 | 38300000 | 0.425684829 | 0.527558 |
| 8 | 74340001 | 74380000 | 1.159789188 | 0.550123 |
| 8 | 42460001 | 42500000 | 0.115161464 | 0.580052 |
| 8 | 42440001 | 42480000 | 0.142056982 | 0.632079 |
| 9 | 71360001 | 71400000 | 0.001218556 | 0.254565 |
| 9 | 91960001 | 92000000 | 0.114680718 | 0.255273 |
| 9 | 115440001 | 115480000 | 0.212612461 | 0.25599 |
| 9 | 91920001 | 91960000 | 0.105715237 | 0.260697 |
| 9 | 38960001 | 39000000 | 0.282815128 | 0.267936 |
| 9 | 89820001 | 89860000 | 0.061760065 | 0.268423 |
| 9 | 88220001 | 88260000 | 0.162518157 | 0.268843 |
| 9 | 82360001 | 82400000 | 0.042016693 | 0.271311 |
| 9 | 99480001 | 99520000 | 0.024676968 | 0.273763 |
| 9 | 71320001 | 71360000 | 0.016654263 | 0.274038 |
| 9 | 71400001 | 71440000 | 0.005485111 | 0.275823 |
| 9 | 72020001 | 72060000 | 0.220757713 | 0.279251 |
| 9 | 71340001 | 71380000 | 0.00768521 | 0.280165 |
| 9 | 89900001 | 89940000 | 0.117314304 | 0.293865 |
| 9 | 74980001 | 75020000 | 0.308171303 | 0.304815 |
| 9 | 70240001 | 70280000 | 0.12603813 | 0.306394 |
| 9 | 99500001 | 99540000 | 0.072356072 | 0.310156 |
| 9 | 67320001 | 67360000 | 0.020016987 | 0.31926 |
| 9 | 72000001 | 72040000 | 0.373466039 | 0.328796 |
| 9 | 71940001 | 71980000 | 0.252063538 | 0.330025 |
| 9 | 74960001 | 75000000 | 0.422746266 | 0.332755 |
| 9 | 71980001 | 72020000 | 0.331247802 | 0.335306 |
| 9 | 70220001 | 70260000 | 0.151742903 | 0.339909 |
| 9 | 70180001 | 70220000 | 0.133151784 | 0.34007 |
| 9 | 70200001 | 70240000 | 0.160006588 | 0.348445 |
| 9 | 75860001 | 75900000 | 0.081881582 | 0.351462 |
| 9 | 71960001 | 72000000 | 0.322139826 | 0.354095 |
| 9 | 75840001 | 75880000 | 0.226719263 | 0.361991 |
| 9 | 88480001 | 88520000 | 0.362057752 | 0.366057 |
| 9 | 88460001 | 88500000 | 0.246084397 | 0.382073 |
| 9 | 88200001 | 88240000 | 0.255441702 | 0.389826 |
| 9 | 80440001 | 80480000 | 0.11279049 | 0.426405 |
| 10 | 43800001 | 43840000 | 0.520587448 | 0.256689 |
| 10 | 57760001 | 57800000 | 0.020097279 | 0.259719 |
| 10 | 30680001 | 30720000 | 0.109375839 | 0.26491 |
| 10 | 57780001 | 57820000 | 0.0704335 | 0.278878 |
| 10 | 30660001 | 30700000 | 0.27420127 | 0.281843 |
| 10 | 33060001 | 33100000 | 0.136250052 | 0.328451 |
| 10 | 30700001 | 30740000 | 0.019148192 | 0.355208 |
| 10 | 30720001 | 30760000 | 0.011475857 | 0.42167 |
| 11 | 39560001 | 39600000 | 0.174430046 | 0.267213 |
| 11 | 68660001 | 68700000 | 0.247587369 | 0.271037 |
| 11 | 39660001 | 39700000 | 0.328902254 | 0.275755 |
| 11 | 39640001 | 39680000 | 0.098345311 | 0.281413 |
| 11 | 39540001 | 39580000 | 0.49351757 | 0.322797 |
| 12 | 26700001 | 26740000 | 0.325975492 | 0.400181 |
| 13 | 67520001 | 67560000 | 0.411430586 | 0.254262 |
| 13 | 80920001 | 80960000 | 0.018164897 | 0.254387 |
| 13 | 92620001 | 92660000 | 0.254732194 | 0.255686 |
| 13 | 104340001 | 104380000 | 0.21533933 | 0.255692 |
| 13 | 113480001 | 113520000 | 0.799785154 | 0.256098 |
| 13 | 92600001 | 92640000 | 0.251183754 | 0.256789 |
| 13 | 67500001 | 67540000 | 0.315238704 | 0.257335 |
| 13 | 24920001 | 24960000 | 0.032138207 | 0.261301 |
| 13 | 96520001 | 96560000 | 0.277408996 | 0.261704 |
| 13 | 113340001 | 113380000 | 0.006096593 | 0.262895 |
| 13 | 181600001 | 181640000 | 0.340002379 | 0.263306 |
| 13 | 153020001 | 153060000 | 0.051018648 | 0.264066 |
| 13 | 140920001 | 140960000 | 0.281553898 | 0.264432 |
| 13 | 151600001 | 151640000 | 0.003477778 | 0.265392 |
| 13 | 104380001 | 104420000 | 0.172606183 | 0.266531 |
| 13 | 152700001 | 152740000 | 0.142435558 | 0.266998 |
| 13 | 92320001 | 92360000 | 0.73331641 | 0.269831 |
| 13 | 165380001 | 165420000 | 0.65414342 | 0.270321 |
| 13 | 152620001 | 152660000 | 0.01303793 | 0.27036 |
| 13 | 104600001 | 104640000 | 0.057584314 | 0.270369 |
| 13 | 148220001 | 148260000 | 0.041289387 | 0.272856 |
| 13 | 104700001 | 104740000 | 0.0926376 | 0.276167 |
| 13 | 172600001 | 172640000 | 0.413001182 | 0.276273 |
| 13 | 80900001 | 80940000 | 0.130341573 | 0.27775 |
| 13 | 104360001 | 104400000 | 0.168089892 | 0.281705 |
| 13 | 152840001 | 152880000 | 0.138894423 | 0.281896 |
| 13 | 144120001 | 144160000 | 0.110326329 | 0.282069 |
| 13 | 92420001 | 92460000 | 0.246210256 | 0.282283 |
| 13 | 149100001 | 149140000 | 0.045762553 | 0.285074 |
| 13 | 92280001 | 92320000 | 0.507649714 | 0.285803 |
| 13 | 153440001 | 153480000 | 0.003548533 | 0.286468 |
| 13 | 152880001 | 152920000 | 0.00295411 | 0.28905 |
| 13 | 152600001 | 152640000 | 0.2967042 | 0.289082 |
| 13 | 149520001 | 149560000 | 0.022991674 | 0.289473 |
| 13 | 165400001 | 165440000 | 0.115374512 | 0.289488 |
| 13 | 172580001 | 172620000 | 0.47453268 | 0.290316 |
| 13 | 113440001 | 113480000 | 0.012648808 | 0.290401 |
| 13 | 161720001 | 161760000 | 0.171971052 | 0.292094 |
| 13 | 153520001 | 153560000 | 0.203842721 | 0.292207 |
| 13 | 92640001 | 92680000 | 0.28146215 | 0.293106 |
| 13 | 153000001 | 153040000 | 0.223169161 | 0.293647 |
| 13 | 113500001 | 113540000 | 0.457335588 | 0.296612 |
| 13 | 96540001 | 96580000 | 0.219710605 | 0.299752 |
| 13 | 92660001 | 92700000 | 0.145979619 | 0.300127 |
| 13 | 140880001 | 140920000 | 0.520800922 | 0.301461 |
| 13 | 152860001 | 152900000 | 0.186674297 | 0.305292 |
| 13 | 104620001 | 104660000 | 0.084595477 | 0.308111 |
| 13 | 181640001 | 181680000 | 0.657144615 | 0.308409 |
| 13 | 92560001 | 92600000 | 0.888298543 | 0.309434 |
| 13 | 154400001 | 154440000 | 0.211312554 | 0.310719 |
| 13 | 152900001 | 152940000 | 0.006827567 | 0.313727 |
| 13 | 162480001 | 162520000 | 0.140791958 | 0.317253 |
| 13 | 92340001 | 92380000 | 0.143896167 | 0.320724 |
| 13 | 180680001 | 180720000 | 0.405722108 | 0.32129 |
| 13 | 153200001 | 153240000 | 0.009577316 | 0.321551 |
| 13 | 92680001 | 92720000 | 0.031560257 | 0.324055 |
| 13 | 174240001 | 174280000 | 0.063585968 | 0.324826 |
| 13 | 154380001 | 154420000 | 0.147769381 | 0.326192 |
| 13 | 148800001 | 148840000 | 0.442956003 | 0.327368 |
| 13 | 96560001 | 96600000 | 0.14784473 | 0.328042 |
| 13 | 174260001 | 174300000 | 0.137068445 | 0.330068 |
| 13 | 104640001 | 104680000 | 0.006418949 | 0.332358 |
| 13 | 153560001 | 153600000 | 0.316578544 | 0.336988 |
| 13 | 165180001 | 165220000 | 0.011191928 | 0.343781 |
| 13 | 181620001 | 181660000 | 1.118892447 | 0.349723 |
| 13 | 153540001 | 153580000 | 0.473317751 | 0.351207 |
| 13 | 165200001 | 165240000 | 0.115521093 | 0.351573 |
| 13 | 96580001 | 96620000 | 0.358522042 | 0.358841 |
| 13 | 165460001 | 165500000 | 0.129289808 | 0.360415 |
| 13 | 180660001 | 180700000 | 0.354091778 | 0.376992 |
| 13 | 180640001 | 180680000 | 0.181859726 | 0.385124 |
| 13 | 96600001 | 96640000 | 0.617407515 | 0.386974 |
| 13 | 149540001 | 149580000 | 0.006105451 | 0.389838 |
| 13 | 165440001 | 165480000 | 0.143933172 | 0.39863 |
| 13 | 165420001 | 165460000 | 0.057425645 | 0.401422 |
| 13 | 163660001 | 163700000 | 0.053956768 | 0.402556 |
| 13 | 96620001 | 96660000 | 0.502119415 | 0.402835 |
| 14 | 70720001 | 70760000 | 0.275009506 | 0.252862 |
| 14 | 119620001 | 119660000 | 0.066789313 | 0.254179 |
| 14 | 95980001 | 96020000 | 0.208852938 | 0.254779 |
| 14 | 119920001 | 119960000 | 0.348363811 | 0.255231 |
| 14 | 9780001 | 9820000 | 0.132150375 | 0.256322 |
| 14 | 48140001 | 48180000 | 0.670144098 | 0.257871 |
| 14 | 97820001 | 97860000 | 0.254380295 | 0.257927 |
| 14 | 118400001 | 118440000 | 0.121152175 | 0.258433 |
| 14 | 50340001 | 50380000 | 0.365980045 | 0.261347 |
| 14 | 95960001 | 96000000 | 0.459961513 | 0.26135 |
| 14 | 50060001 | 50100000 | 0.403129024 | 0.261564 |
| 14 | 50500001 | 50540000 | 0.783495858 | 0.262136 |
| 14 | 48080001 | 48120000 | 0.187677622 | 0.262211 |
| 14 | 50080001 | 50120000 | 0.736572165 | 0.262703 |
| 14 | 49980001 | 50020000 | 0.147870379 | 0.263064 |
| 14 | 98200001 | 98240000 | 0.886566465 | 0.264048 |
| 14 | 67500001 | 67540000 | 0.107237457 | 0.264266 |
| 14 | 96260001 | 96300000 | 0.436587205 | 0.264367 |
| 14 | 28160001 | 28200000 | 0.151063765 | 0.265155 |
| 14 | 50140001 | 50180000 | 0.893944345 | 0.26617 |
| 14 | 50120001 | 50160000 | 0.466303381 | 0.268007 |
| 14 | 50220001 | 50260000 | 0.873878273 | 0.268516 |
| 14 | 119940001 | 119980000 | 0.292794226 | 0.268567 |
| 14 | 50440001 | 50480000 | 0.70091049 | 0.270978 |
| 14 | 48100001 | 48140000 | 0.062155567 | 0.271312 |
| 14 | 114840001 | 114880000 | 0.002438038 | 0.271574 |
| 14 | 112940001 | 112980000 | 0.08317778 | 0.272935 |
| 14 | 48060001 | 48100000 | 0.148289488 | 0.273201 |
| 14 | 48120001 | 48160000 | 0.384133571 | 0.279325 |
| 14 | 55480001 | 55520000 | 0.010898287 | 0.279357 |
| 14 | 99080001 | 99120000 | 0.273576708 | 0.280633 |
| 14 | 48040001 | 48080000 | 0.144412426 | 0.280669 |
| 14 | 50460001 | 50500000 | 0.685349719 | 0.280834 |
| 14 | 96240001 | 96280000 | 0.414792258 | 0.281044 |
| 14 | 119660001 | 119700000 | 0.137903125 | 0.282573 |
| 14 | 46440001 | 46480000 | 0.003916539 | 0.283765 |
| 14 | 109480001 | 109520000 | 0.230466899 | 0.291145 |
| 14 | 119960001 | 120000000 | 0.352018734 | 0.29389 |
| 14 | 9800001 | 9840000 | 0.385416151 | 0.294083 |
| 14 | 70940001 | 70980000 | 0.822697731 | 0.298315 |
| 14 | 119880001 | 119920000 | 0.020388608 | 0.29907 |
| 14 | 106300001 | 106340000 | 0.076962327 | 0.299072 |
| 14 | 66320001 | 66360000 | 0.156911189 | 0.299285 |
| 14 | 121320001 | 121360000 | 0.045111521 | 0.301706 |
| 14 | 101660001 | 101700000 | 0.857407217 | 0.302233 |
| 14 | 109500001 | 109540000 | 0.357340753 | 0.304607 |
| 14 | 82520001 | 82560000 | 0.188255463 | 0.311669 |
| 14 | 55500001 | 55540000 | 0.143143222 | 0.311842 |
| 14 | 106260001 | 106300000 | 0.352647311 | 0.317827 |
| 14 | 70680001 | 70720000 | 0.419854231 | 0.317904 |
| 14 | 119980001 | 120020000 | 0.344887166 | 0.319998 |
| 14 | 106340001 | 106380000 | 0.380825441 | 0.322255 |
| 14 | 57520001 | 57560000 | 0.181118736 | 0.323286 |
| 14 | 70660001 | 70700000 | 0.276647882 | 0.323863 |
| 14 | 121420001 | 121460000 | 0.058548782 | 0.324231 |
| 14 | 119640001 | 119680000 | 0.597956177 | 0.325834 |
| 14 | 106280001 | 106320000 | 0.420522712 | 0.329293 |
| 14 | 58200001 | 58240000 | 0.165448191 | 0.331581 |
| 14 | 121600001 | 121640000 | 0.072199836 | 0.338728 |
| 14 | 57540001 | 57580000 | 0.183816737 | 0.341499 |
| 14 | 99800001 | 99840000 | 0.878904493 | 0.343259 |
| 14 | 70780001 | 70820000 | 0.264784779 | 0.34573 |
| 14 | 101680001 | 101720000 | 0.836686494 | 0.347684 |
| 14 | 106420001 | 106460000 | 0.210519512 | 0.349535 |
| 14 | 118380001 | 118420000 | 0.206731612 | 0.350496 |
| 14 | 106360001 | 106400000 | 0.478349884 | 0.354 |
| 14 | 82500001 | 82540000 | 0.204668951 | 0.366374 |
| 14 | 106400001 | 106440000 | 0.35983272 | 0.37078 |
| 14 | 58220001 | 58260000 | 0.28007463 | 0.372861 |
| 14 | 99780001 | 99820000 | 1.133387623 | 0.376569 |
| 14 | 67520001 | 67560000 | 0.297877329 | 0.380509 |
| 14 | 121340001 | 121380000 | 0.348812282 | 0.381409 |
| 14 | 121380001 | 121420000 | 0.367883691 | 0.393776 |
| 14 | 121400001 | 121440000 | 0.402085645 | 0.395225 |
| 14 | 120100001 | 120140000 | 0.350665445 | 0.399158 |
| 14 | 121360001 | 121400000 | 0.436207414 | 0.401031 |
| 14 | 120060001 | 120100000 | 0.53315324 | 0.430183 |
| 14 | 120080001 | 120120000 | 0.866233928 | 0.4427 |
| 14 | 70840001 | 70880000 | 0.393527037 | 0.462105 |
| 14 | 120040001 | 120080000 | 0.317789034 | 0.490005 |
| 14 | 70800001 | 70840000 | 0.418197804 | 0.515374 |
| 14 | 70820001 | 70860000 | 0.22425356 | 0.567848 |
| 15 | 97800001 | 97840000 | 0.379650844 | 0.270093 |
| 15 | 94720001 | 94760000 | 0.039008082 | 0.270239 |
| 15 | 94560001 | 94600000 | 0.537748925 | 0.271555 |
| 15 | 85840001 | 85880000 | 0.422967799 | 0.275717 |
| 15 | 85860001 | 85900000 | 0.351959111 | 0.279939 |
| 15 | 5320001 | 5360000 | 0.242661109 | 0.282894 |
| 15 | 5300001 | 5340000 | 0.459721584 | 0.284693 |
| 15 | 91340001 | 91380000 | 0.038793947 | 0.298642 |
| 15 | 94700001 | 94740000 | 0.550260502 | 0.308724 |
| 15 | 94580001 | 94620000 | 0.867069848 | 0.315244 |
| 15 | 94740001 | 94780000 | 0.210138373 | 0.315951 |
| 15 | 94340001 | 94380000 | 0.366856943 | 0.317662 |
| 15 | 90720001 | 90760000 | 0.114281506 | 0.326281 |
| 15 | 90820001 | 90860000 | 0.178123458 | 0.328595 |
| 15 | 90740001 | 90780000 | 0.107118314 | 0.331473 |
| 15 | 94320001 | 94360000 | 0.176596843 | 0.332887 |
| 15 | 90700001 | 90740000 | 0.166963953 | 0.337455 |
| 15 | 90760001 | 90800000 | 0.210612082 | 0.338695 |
| 15 | 90660001 | 90700000 | 0.190203548 | 0.339347 |
| 15 | 90640001 | 90680000 | 0.182483046 | 0.342714 |
| 15 | 90620001 | 90660000 | 0.170755453 | 0.342968 |
| 15 | 90780001 | 90820000 | 0.305830412 | 0.34385 |
| 15 | 90680001 | 90720000 | 0.219223767 | 0.344389 |
| 15 | 90800001 | 90840000 | 0.342964792 | 0.348627 |
| 15 | 94360001 | 94400000 | 0.008602779 | 0.365752 |
| 15 | 94660001 | 94700000 | 0.510607394 | 0.379364 |
| 15 | 94680001 | 94720000 | 1.456430205 | 0.41098 |
| 16 | 49400001 | 49440000 | 0.118221431 | 0.256504 |
| 16 | 19220001 | 19260000 | 1.735450143 | 0.260256 |
| 16 | 17220001 | 17260000 | 0.578906669 | 0.265476 |
| 16 | 38960001 | 39000000 | 0.029834955 | 0.267958 |
| 16 | 43000001 | 43040000 | 0.580985031 | 0.271364 |
| 16 | 25620001 | 25660000 | 0.016208089 | 0.275025 |
| 16 | 39080001 | 39120000 | 0.269582192 | 0.280431 |
| 16 | 39040001 | 39080000 | 0.192745688 | 0.289121 |
| 16 | 43020001 | 43060000 | 0.811558548 | 0.291993 |
| 16 | 53960001 | 54000000 | 0.162491412 | 0.300359 |
| 16 | 44740001 | 44780000 | 0.086214209 | 0.300812 |
| 16 | 57040001 | 57080000 | 0.037767952 | 0.303348 |
| 16 | 53940001 | 53980000 | 0.063350548 | 0.307484 |
| 16 | 17200001 | 17240000 | 0.602809444 | 0.308205 |
| 16 | 28580001 | 28620000 | 0.00674584 | 0.310622 |
| 16 | 39060001 | 39100000 | 0.472159306 | 0.317269 |
| 16 | 47780001 | 47820000 | 0.06837484 | 0.446803 |
| 17 | 24940001 | 24980000 | 0.317639506 | 0.25712 |
| 17 | 36360001 | 36400000 | 0.030934842 | 0.273731 |
| 17 | 35440001 | 35480000 | 0.098374742 | 0.276991 |
| 17 | 35420001 | 35460000 | 0.116268797 | 0.30299 |
| 17 | 56300001 | 56340000 | 0.435444492 | 0.303076 |
| 17 | 56340001 | 56380000 | 0.142845232 | 0.303972 |
| 17 | 56320001 | 56360000 | 0.393761744 | 0.310636 |
| 17 | 50720001 | 50760000 | 0.257474779 | 0.339958 |
| 17 | 50700001 | 50740000 | 0.096993436 | 0.419041 |

**Table S10.** The selected genes harbored in selection regions of Anhui pig population versus commercial pig population

| Gene stable ID | Gene name | Gene description |
| --- | --- | --- |
| ENSSSCG00000009755 | AACS | acetoacetyl-CoA synthetase [Source:VGNC Symbol;Acc:VGNC:84937] |
| ENSSSCG00000003776 | ACADM | acyl-CoA dehydrogenase medium chain [Source:VGNC Symbol;Acc:VGNC:85000] |
| ENSSSCG00000017566 | ACSF2 | acyl-CoA synthetase family member 2 [Source:VGNC Symbol;Acc:VGNC:85031] |
| ENSSSCG00000038452 | ADAMTS17 | ADAM metallopeptidase with thrombospondin type 1 motif 17 [Source:VGNC Symbol;Acc:VGNC:85079] |
| ENSSSCG00000007950 | ADCY9 | adenylate cyclase 9 [Source:VGNC Symbol;Acc:VGNC:85113] |
| ENSSSCG00000003761 | ADGRL2 | adhesion G protein-coupled receptor L2 [Source:VGNC Symbol;Acc:VGNC:85136] |
| ENSSSCG00000040199 | AFG1L | AFG1 like ATPase [Source:VGNC Symbol;Acc:VGNC:85170] |
| ENSSSCG00000006872 | AGL | amylo-alpha-1, 6-glucosidase, 4-alpha-glucanotransferase [Source:HGNC Symbol;Acc:HGNC:321] |
| ENSSSCG00000015310 | AKAP9 | A-kinase anchoring protein 9 [Source:VGNC Symbol;Acc:VGNC:85225] |
| ENSSSCG00000008948 | ALB | albumin [Source:VGNC Symbol;Acc:VGNC:98911] |
| ENSSSCG00000008936 | AMTN | amelotin [Source:VGNC Symbol;Acc:VGNC:85290] |
| ENSSSCG00000016042 | ANKAR | ankyrin and armadillo repeat containing [Source:VGNC Symbol;Acc:VGNC:96126] |
| ENSSSCG00000015314 | ANKIB1 | ankyrin repeat and IBR domain containing 1 [Source:VGNC Symbol;Acc:VGNC:85316] |
| ENSSSCG00000001534 | ANKS1A | ankyrin repeat and sterile alpha motif domain containing 1A [Source:VGNC Symbol;Acc:VGNC:85348] |
| ENSSSCG00000009978 | AP1B1 | adaptor related protein complex 1 subunit beta 1 [Source:VGNC Symbol;Acc:VGNC:85377] |
| ENSSSCG00000007460 | ARFGEF2 | ADP ribosylation factor guanine nucleotide exchange factor 2 [Source:VGNC Symbol;Acc:VGNC:95672] |
| ENSSSCG00000009029 | ARHGAP10 | Rho GTPase activating protein 10 [Source:VGNC Symbol;Acc:VGNC:85458] |
| ENSSSCG00000026224 | ARHGAP31 | Rho GTPase activating protein 31 [Source:VGNC Symbol;Acc:VGNC:85470] |
| ENSSSCG00000027278 | ARL6 | ADP ribosylation factor like GTPase 6 [Source:VGNC Symbol;Acc:VGNC:85524] |
| ENSSSCG00000010571 | ARMH3 | armadillo like helical domain containing 3 [Source:VGNC Symbol;Acc:VGNC:85534] |
| ENSSSCG00000015333 | ASB4 | ankyrin repeat and SOCS box containing 4 [Source:VGNC Symbol;Acc:VGNC:97891] |
| ENSSSCG00000004821 | ASB7 | ankyrin repeat and SOCS box containing 7 [Source:VGNC Symbol;Acc:VGNC:85566] |
| ENSSSCG00000008072 | ASPN | asporin [Source:VGNC Symbol;Acc:VGNC:85586] |
| ENSSSCG00000013065 | ASRGL1 | asparaginase and isoaspartyl peptidase 1 [Source:VGNC Symbol;Acc:VGNC:85589] |
| ENSSSCG00000005502 | ASTN2 | astrotactin 2 [Source:VGNC Symbol;Acc:VGNC:85594] |
| ENSSSCG00000010438 | ATAD1 | ATPase family AAA domain containing 1 [Source:VGNC Symbol;Acc:VGNC:85598] |
| ENSSSCG00000006346 | ATF6 | activating transcription factor 6 [Source:VGNC Symbol;Acc:VGNC:85609] |
| ENSSSCG00000011575 | ATG7 | autophagy related 7 [Source:VGNC Symbol;Acc:VGNC:85625] |
| ENSSSCG00000033278 | ATP6V1D | ATPase H+ transporting V1 subunit D [Source:VGNC Symbol;Acc:VGNC:85674] |
| ENSSSCG00000038806 | AXL | AXL receptor tyrosine kinase [Source:VGNC Symbol;Acc:VGNC:85709] |
| ENSSSCG00000007465 | B4GALT5 | beta-1,4-galactosyltransferase 5 [Source:VGNC Symbol;Acc:VGNC:96499] |
| ENSSSCG00000016968 | BDP1 | B double prime 1, subunit of RNA polymerase III transcription initiation factor IIIB [Source:VGNC Symbol;Acc:VGNC:85796] |
| ENSSSCG00000008802 | BEND4 | BEN domain containing 4 [Source:VGNC Symbol;Acc:VGNC:85801] |
| ENSSSCG00000006640 | BNIPL | BCL2 interacting protein like [Source:VGNC Symbol;Acc:VGNC:85858] |
| ENSSSCG00000032831 | BRI3BP | BRI3 binding protein [Source:VGNC Symbol;Acc:VGNC:85879] |
| ENSSSCG00000022333 | C16orf87 | chromosome 6 C16orf87 homolog [Source:VGNC Symbol;Acc:VGNC:96927] |
| ENSSSCG00000004874 | C18orf63 | chromosome 1 C18orf63 homolog [Source:VGNC Symbol;Acc:VGNC:85966] |
| ENSSSCG00000006639 | C1orf56 | chromosome 4 C1orf56 homolog [Source:VGNC Symbol;Acc:VGNC:86032] |
| ENSSSCG00000033941 | C2orf88 | chromosome 15 C2orf88 homolog [Source:VGNC Symbol;Acc:VGNC:96170] |
| ENSSSCG00000009069 | C4orf33 | chromosome 8 C4orf33 homolog [Source:VGNC Symbol;Acc:VGNC:97907] |
| ENSSSCG00000042317 | C4orf51 | chromosome 4 open reading frame 51 [Source:HGNC Symbol;Acc:HGNC:37264] |
| ENSSSCG00000004301 | C6orf163 | chromosome 1 C6orf163 homolog [Source:VGNC Symbol;Acc:VGNC:85968] |
| ENSSSCG00000031105 | C9orf85 | chromosome 1 C9orf85 homolog [Source:VGNC Symbol;Acc:VGNC:85976] |
| ENSSSCG00000003126 | CABP5 | calcium binding protein 5 [Source:VGNC Symbol;Acc:VGNC:86112] |
| ENSSSCG00000039238 | CABS1 | calcium binding protein, spermatid associated 1 [Source:VGNC Symbol;Acc:VGNC:86113] |
| ENSSSCG00000022486 | CBLB | Cbl proto-onco B [Source:VGNC Symbol;Acc:VGNC:86223] |
| ENSSSCG00000002284 | CCDC196 | coiled-coil domain containing 196 [Source:HGNC Symbol;Acc:HGNC:20100] |
| ENSSSCG00000003016 | CCDC97 | coiled-coil domain containing 97 [Source:VGNC Symbol;Acc:VGNC:86331] |
| ENSSSCG00000031881 | CDC42SE1 | CDC42 small effector 1 [Source:VGNC Symbol;Acc:VGNC:86460] |
| ENSSSCG00000026900 | CDIN1 | CDAN1 interacting nuclease 1 [Source:VGNC Symbol;Acc:VGNC:85960] |
| ENSSSCG00000008921 | CENPC | centromere protein C [Source:VGNC Symbol;Acc:VGNC:86546] |
| ENSSSCG00000008070 | CENPP | centromere protein P [Source:VGNC Symbol;Acc:VGNC:98865] |
| ENSSSCG00000002410 | CEP128 | centrosomal protein 128 [Source:VGNC Symbol;Acc:VGNC:86560] |
| ENSSSCG00000008909 | CLOCK | clock circadian regulator [Source:VGNC Symbol;Acc:VGNC:86774] |
| ENSSSCG00000011666 | CLSTN2 | calsyntenin 2 [Source:VGNC Symbol;Acc:VGNC:86785] |
| ENSSSCG00000009519 | CLYBL | citramalyl-CoA lyase [Source:VGNC Symbol;Acc:VGNC:86796] |
| ENSSSCG00000038160 | CNGA1 | cyclic nucleotide gated channel subunit alpha 1 [Source:VGNC Symbol;Acc:VGNC:86815] |
| ENSSSCG00000027849 | CNR2 | cannabinoid receptor 2 [Source:VGNC Symbol;Acc:VGNC:86844] |
| ENSSSCG00000044472 | COL19A1 | collagen type XIX alpha 1 chain [Source:HGNC Symbol;Acc:HGNC:2196] |
| ENSSSCG00000006386 | COPA | COPI coat complex subunit alpha [Source:VGNC Symbol;Acc:VGNC:86895] |
| ENSSSCG00000008854 | CPE | carboxypeptidase E [Source:VGNC Symbol;Acc:VGNC:86936] |
| ENSSSCG00000038031 | CPSF6 | cleavage and polyadenylation specific factor 6 [Source:VGNC Symbol;Acc:VGNC:86962] |
| ENSSSCG00000007951 | CREBBP | CREB binding protein [Source:VGNC Symbol;Acc:VGNC:86985] |
| ENSSSCG00000010095 | CRKL | CRK like proto-onco, adaptor protein [Source:VGNC Symbol;Acc:VGNC:86997] |
| ENSSSCG00000025534 | CSE1L | chromosome segregation 1 like [Source:VGNC Symbol;Acc:VGNC:96039] |
| ENSSSCG00000009267 | CSN3 | casein kappa [Source:VGNC Symbol;Acc:VGNC:87041] |
| ENSSSCG00000036480 | CTNNA3 | catenin alpha 3 [Source:VGNC Symbol;Acc:VGNC:87064] |
| ENSSSCG00000011104 | CUL2 | cullin 2 [Source:VGNC Symbol;Acc:VGNC:96090] |
| ENSSSCG00000004875 | CYB5A | cytochrome b5 type A [Source:VGNC Symbol;Acc:VGNC:96727] |
| ENSSSCG00000003006 | CYP2B22 | cytochrome P450 2B22 [Source:NCBI gene (formerly Entrezgene);Acc:403104] |
| ENSSSCG00000035347 | CYP2C42 | cytochrome P450 C42 [Source:NCBI gene (formerly Entrezgene);Acc:403111] |
| ENSSSCG00000032803 | CYP2S1 | cytochrome P450 family 2 subfamily S member 1 [Source:VGNC Symbol;Acc:VGNC:103351] |
| ENSSSCG00000015311 | CYP51A1 | cytochrome P450 family 51 subfamily A member 1 [Source:VGNC Symbol;Acc:VGNC:103377] |
| ENSSSCG00000009023 | DCLK2 | doublecortin like kinase 2 [Source:VGNC Symbol;Acc:VGNC:98918] |
| ENSSSCG00000021427 | DCTN4 | dynactin subunit 4 [Source:VGNC Symbol;Acc:VGNC:99635] |
| ENSSSCG00000007854 | DCUN1D3 | defective in cullin neddylation 1 domain containing 3 [Source:VGNC Symbol;Acc:VGNC:87196] |
| ENSSSCG00000008831 | DCUN1D4 | defective in cullin neddylation 1 domain containing 4 [Source:VGNC Symbol;Acc:VGNC:87197] |
| ENSSSCG00000006365 | DEDD | death effector domain containing [Source:VGNC Symbol;Acc:VGNC:98777] |
| ENSSSCG00000030182 | DEDD2 | death effector domain containing 2 [Source:VGNC Symbol;Acc:VGNC:87237] |
| ENSSSCG00000040638 | DIO2 | iodothyronine deiodinase 2 [Source:NCBI gene (formerly Entrezgene);Acc:414379] |
| ENSSSCG00000029196 | DIP2B | disco interacting B [Source:VGNC Symbol;Acc:VGNC:87306] |
| ENSSSCG00000036527 | DNASE1 | deoxyribonuclease 1 [Source:VGNC Symbol;Acc:VGNC:102564] |
| ENSSSCG00000026532 | DNASE2B | deoxyribonuclease 2 beta [Source:VGNC Symbol;Acc:VGNC:96980] |
| ENSSSCG00000007252 | DNMT3B | DNA methyltransferase 3 beta [Source:VGNC Symbol;Acc:VGNC:96228] |
| ENSSSCG00000017008 | DOCK2 | dedicator of cytokinesis 2 [Source:VGNC Symbol;Acc:VGNC:87392] |
| ENSSSCG00000005240 | DOCK8 | dedicator of cytokinesis 8 [Source:VGNC Symbol;Acc:VGNC:87398] |
| ENSSSCG00000035254 | DPH6 | diphthamine biosynthesis 6 [Source:VGNC Symbol;Acc:VGNC:87417] |
| ENSSSCG00000010029 | DRG1 | developmentally regulated GTP binding protein 1 [Source:VGNC Symbol;Acc:VGNC:87448] |
| ENSSSCG00000010797 | EARS2 | glutamyl-tRNA synthetase 2, mitochondrial [Source:VGNC Symbol;Acc:VGNC:87524] |
| ENSSSCG00000009655 | EBF2 | EBF transcription factor 2 [Source:VGNC Symbol;Acc:VGNC:87526] |
| ENSSSCG00000028549 | ECM2 | extracellular matrix protein 2 [Source:VGNC Symbol;Acc:VGNC:87534] |
| ENSSSCG00000033295 | EIF2S1 | eukaryotic translation initiation factor 2 subunit alpha [Source:VGNC Symbol;Acc:VGNC:87614] |
| ENSSSCG00000010030 | EIF4ENIF1 | eukaryotic translation initiation factor 4E nuclear import factor 1 [Source:VGNC Symbol;Acc:VGNC:87630] |
| ENSSSCG00000029920 | ELF2 | E74 like ETS transcription factor 2 [Source:VGNC Symbol;Acc:VGNC:87642] |
| ENSSSCG00000003125 | ELSPBP1 | epididymal sperm binding protein 1 [Source:VGNC Symbol;Acc:VGNC:97049] |
| ENSSSCG00000008919 | EPHA5 | EPH receptor A5 [Source:VGNC Symbol;Acc:VGNC:87733] |
| ENSSSCG00000001561 | ETV7 | ETS variant transcription factor 7 [Source:VGNC Symbol;Acc:VGNC:87816] |
| ENSSSCG00000006901 | EVI5 | ecotropic viral integration site 5 [Source:VGNC Symbol;Acc:VGNC:98793] |
| ENSSSCG00000009974 | EWSR1 | EWS RNA binding protein 1 [Source:VGNC Symbol;Acc:VGNC:87822] |
| ENSSSCG00000000985 | EXOC2 | exocyst complex component 2 [Source:VGNC Symbol;Acc:VGNC:87825] |
| ENSSSCG00000014057 | FAF2 | Fas associated factor family member 2 [Source:VGNC Symbol;Acc:VGNC:87880] |
| ENSSSCG00000002286 | FAM71D | family with sequence similarity 71 member D [Source:VGNC Symbol;Acc:VGNC:87978] |
| ENSSSCG00000020879 | FBXW7 | F-box and WD repeat domain containing 7 [Source:VGNC Symbol;Acc:VGNC:98925] |
| ENSSSCG00000047156 | FDCSP | follicular dendritic cell secreted protein [Source:HGNC Symbol;Acc:HGNC:19215] |
| ENSSSCG00000022419 | FEM1B | fem-1 homolog B [Source:VGNC Symbol;Acc:VGNC:88083] |
| ENSSSCG00000035244 | FOXB1 | forkhead box B1 [Source:VGNC Symbol;Acc:VGNC:88195] |
| ENSSSCG00000027183 | FSTL5 | follistatin like 5 [Source:VGNC Symbol;Acc:VGNC:88257] |
| ENSSSCG00000027659 | FUCA1 | alpha-L-fucosidase 1 [Source:VGNC Symbol;Acc:VGNC:88262] |
| ENSSSCG00000036436 | FZD1 | frizzled class receptor 1 [Source:VGNC Symbol;Acc:VGNC:88278] |
| ENSSSCG00000009048 | GAB1 | GRB2 associated binding protein 1 [Source:VGNC Symbol;Acc:VGNC:88294] |
| ENSSSCG00000006636 | GABPB2 | GA binding protein transcription factor subunit beta 2 [Source:VGNC Symbol;Acc:VGNC:98796] |
| ENSSSCG00000030515 | GABRA4 | gamma-aminobutyric acid type A receptor subunit alpha4 [Source:VGNC Symbol;Acc:VGNC:88303] |
| ENSSSCG00000035621 | GABRB1 | gamma-aminobutyric acid type A receptor subunit beta1 [Source:VGNC Symbol;Acc:VGNC:88306] |
| ENSSSCG00000025564 | GAS2L1 | growth arrest specific 2 like 1 [Source:VGNC Symbol;Acc:VGNC:88355] |
| ENSSSCG00000004584 | GCNT3 | glucosaminyl (N-acetyl) transferase 3, mucin type [Source:VGNC Symbol;Acc:VGNC:103097] |
| ENSSSCG00000010798 | GGA2 | golgi associated, gamma adaptin ear containing, ARF binding protein 2 [Source:VGNC Symbol;Acc:VGNC:88432] |
| ENSSSCG00000033562 | GJB7 | gap junction protein beta 7 [Source:VGNC Symbol;Acc:VGNC:88467] |
| ENSSSCG00000040981 | GMFG | glia maturation factor gamma [Source:VGNC Symbol;Acc:VGNC:88510] |
| ENSSSCG00000034192 | GNAO1 | G protein subunit alpha o1 [Source:VGNC Symbol;Acc:VGNC:88525] |
| ENSSSCG00000033763 | GNAQ | G protein subunit alpha q [Source:VGNC Symbol;Acc:VGNC:103100] |
| ENSSSCG00000002285 | GPHN | gephyrin [Source:VGNC Symbol;Acc:VGNC:88587] |
| ENSSSCG00000004144 | HECA | hdc homolog, cell cycle regulator [Source:VGNC Symbol;Acc:VGNC:88831] |
| ENSSSCG00000004561 | HERC1 | HECT and RLD domain containing E3 ubiquitin protein ligase family member 1 [Source:VGNC Symbol;Acc:VGNC:88849] |
| ENSSSCG00000036472 | HIC2 | HIC ZBTB transcriptional repressor 2 [Source:HGNC Symbol;Acc:HGNC:18595] |
| ENSSSCG00000030049 | HNRNPUL1 | heteroous nuclear ribonucleoprotein U like 1 [Source:VGNC Symbol;Acc:VGNC:88926] |
| ENSSSCG00000035251 | HTR1E | 5-hydroxytryptamine receptor 1E [Source:VGNC Symbol;Acc:VGNC:103108] |
| ENSSSCG00000008068 | IARS1 | isoleucyl-tRNA synthetase 1 [Source:HGNC Symbol;Acc:HGNC:5330] |
| ENSSSCG00000030560 | IGF1R | insulin like growth factor 1 receptor [Source:NCBI gene (formerly Entrezgene);Acc:397350] |
| ENSSSCG00000008162 | IL1R1 | interleukin 1 receptor type 1 [Source:VGNC Symbol;Acc:VGNC:89092] |
| ENSSSCG00000032795 | IL1RL1 | interleukin 1 receptor like 1 [Source:HGNC Symbol;Acc:HGNC:5998] |
| ENSSSCG00000001527 | ILRUN | inflammation and lipid regulator with UBA-like and NBR1-like domains [Source:VGNC Symbol;Acc:VGNC:89122] |
| ENSSSCG00000009050 | INPP4B | inositol polyphosphate-4-phosphatase type II B [Source:VGNC Symbol;Acc:VGNC:89141] |
| ENSSSCG00000013566 | INSR | insulin receptor [Source:VGNC Symbol;Acc:VGNC:89153] |
| ENSSSCG00000017010 | INSYN2B | inhibitory synaptic factor family member 2B [Source:VGNC Symbol;Acc:VGNC:89157] |
| ENSSSCG00000005388 | INVS | inversin [Source:VGNC Symbol;Acc:VGNC:89171] |
| ENSSSCG00000035623 | IQCM | IQ motif containing M [Source:HGNC Symbol;Acc:HGNC:53443] |
| ENSSSCG00000004961 | ITGA11 | integrin subunit alpha 11 [Source:VGNC Symbol;Acc:VGNC:89233] |
| ENSSSCG00000005235 | KANK1 | KN motif and ankyrin repeat domains 1 [Source:VGNC Symbol;Acc:VGNC:103112] |
| ENSSSCG00000001562 | KCTD20 | potassium channel tetramerization domain containing 20 [Source:VGNC Symbol;Acc:VGNC:89399] |
| ENSSSCG00000007080 | KIF16B | kinesin family member 16B [Source:HGNC Symbol;Acc:HGNC:15869] |
| ENSSSCG00000008842 | KIT | KIT proto-onco, receptor tyrosine kinase [Source:VGNC Symbol;Acc:VGNC:98060] |
| ENSSSCG00000026297 | KLB | klotho beta [Source:VGNC Symbol;Acc:VGNC:89485] |
| ENSSSCG00000026006 | KLF13 | Kruppel like factor 13 [Source:VGNC Symbol;Acc:VGNC:89493] |
| ENSSSCG00000038597 | KLHDC9 | kelch domain containing 9 [Source:VGNC Symbol;Acc:VGNC:89510] |
| ENSSSCG00000008858 | KLHL2 | kelch like family member 2 [Source:VGNC Symbol;Acc:VGNC:89518] |
| ENSSSCG00000027002 | KLHL32 | kelch like family member 32 [Source:VGNC Symbol;Acc:VGNC:89527] |
| ENSSSCG00000038469 | KLHL7 | kelch like family member 7 [Source:HGNC Symbol;Acc:HGNC:15646] |
| ENSSSCG00000004469 | LCA5 | lebercilin LCA5 [Source:VGNC Symbol;Acc:VGNC:89654] |
| ENSSSCG00000026579 | LEKR1 | leucine, glutamate and lysine rich 1 [Source:HGNC Symbol;Acc:HGNC:33765] |
| ENSSSCG00000000469 | LEMD3 | LEM domain containing 3 [Source:VGNC Symbol;Acc:VGNC:89679] |
| ENSSSCG00000003128 | LIG1 | DNA ligase 1 [Source:VGNC Symbol;Acc:VGNC:89720] |
| ENSSSCG00000010025 | LIMK2 | LIM domain kinase 2 [Source:VGNC Symbol;Acc:VGNC:89728] |
| ENSSSCG00000004820 | LINS1 | lines homolog 1 [Source:VGNC Symbol;Acc:VGNC:89736] |
| ENSSSCG00000009021 | LRBA | LPS responsive beige-like anchor protein [Source:VGNC Symbol;Acc:VGNC:98074] |
| ENSSSCG00000034308 | LRMDA | leucine rich melanocyte differentiation associated [Source:HGNC Symbol;Acc:HGNC:23405] |
| ENSSSCG00000008832 | LRRC66 | leucine rich repeat containing 66 [Source:HGNC Symbol;Acc:HGNC:34299] |
| ENSSSCG00000003793 | LRRC7 | leucine rich repeat containing 7 [Source:HGNC Symbol;Acc:HGNC:18531] |
| ENSSSCG00000027941 | LSAMP | limbic system associated membrane protein [Source:VGNC Symbol;Acc:VGNC:89868] |
| ENSSSCG00000036360 | LURAP1L | leucine rich adaptor protein 1 like [Source:VGNC Symbol;Acc:VGNC:89896] |
| ENSSSCG00000003532 | LUZP1 | leucine zipper protein 1 [Source:VGNC Symbol;Acc:VGNC:89897] |
| ENSSSCG00000023191 | LYPLA2 | lysophospholipase 2 [Source:VGNC Symbol;Acc:VGNC:89914] |
| ENSSSCG00000010151 | LYST | lysosomal trafficking regulator [Source:VGNC Symbol;Acc:VGNC:89921] |
| ENSSSCG00000000492 | LYZ | lysozyme [Source:HGNC Symbol;Acc:HGNC:6740] |
| ENSSSCG00000009022 | MAB21L2 | mab-21 like 2 [Source:VGNC Symbol;Acc:VGNC:89933] |
| ENSSSCG00000030655 | MAMDC2 | MAM domain containing 2 [Source:VGNC Symbol;Acc:VGNC:89962] |
| ENSSSCG00000035648 | MANEA | mannosidase endo-alpha [Source:VGNC Symbol;Acc:VGNC:89971] |
| ENSSSCG00000010081 | MAPK1 | mitogen-activated protein kinase 1 [Source:VGNC Symbol;Acc:VGNC:89996] |
| ENSSSCG00000015650 | MAPKAPK2 | MAPK activated protein kinase 2 [Source:VGNC Symbol;Acc:VGNC:90011] |
| ENSSSCG00000007253 | MAPRE1 | microtubule associated protein RP/EB family member 1 [Source:VGNC Symbol;Acc:VGNC:96410] |
| ENSSSCG00000039175 | MARCHF1 | membrane associated ring-CH-type finger 1 [Source:VGNC Symbol;Acc:VGNC:98939] |
| ENSSSCG00000011720 | MBNL1 | muscleblind like splicing regulator 1 [Source:VGNC Symbol;Acc:VGNC:90054] |
| ENSSSCG00000028411 | MC5R | melanocortin 5 receptor [Source:HGNC Symbol;Acc:HGNC:6933] |
| ENSSSCG00000006281 | METTL11B | methyltransferase like 11B [Source:VGNC Symbol;Acc:VGNC:90155] |
| ENSSSCG00000041409 | MFSD4B | major facilitator superfamily domain containing 4B [Source:HGNC Symbol;Acc:HGNC:21053] |
| ENSSSCG00000030423 | MIER1 | MIER1 transcriptional regulator [Source:VGNC Symbol;Acc:VGNC:90219] |
| ENSSSCG00000030278 | MLLT11 | MLLT11 transcription factor 7 cofactor [Source:VGNC Symbol;Acc:VGNC:96591] |
| ENSSSCG00000009039 | MMAA | metabolism of cobalamin associated A [Source:HGNC Symbol;Acc:HGNC:18871] |
| ENSSSCG00000037532 | MNDA | myeloid cell nuclear differentiation antigen [Source:HGNC Symbol;Acc:HGNC:7183] |
| ENSSSCG00000002287 | MPP5 | membrane palmitoylated protein 5 [Source:VGNC Symbol;Acc:VGNC:98136] |
| ENSSSCG00000026004 | MSRB3 | methionine sulfoxide reductase B3 [Source:VGNC Symbol;Acc:VGNC:90427] |
| ENSSSCG00000016047 | MSTN | myostatin [Source:VGNC Symbol;Acc:VGNC:96320] |
| ENSSSCG00000004543 | MTFMT | mitochondrial methionyl-tRNA formyltransferase [Source:VGNC Symbol;Acc:VGNC:90445] |
| ENSSSCG00000036534 | MYOM3 | myomesin 3 [Source:VGNC Symbol;Acc:VGNC:90541] |
| ENSSSCG00000032838 | MYOZ2 | myozenin 2 [Source:VGNC Symbol;Acc:VGNC:90543] |
| ENSSSCG00000023871 | NAALADL2 | N-acetylated alpha-linked acidic dipeptidase like 2 [Source:VGNC Symbol;Acc:VGNC:90559] |
| ENSSSCG00000004542 | NARS1 | asparaginyl-tRNA synthetase 1 [Source:VGNC Symbol;Acc:VGNC:90583] |
| ENSSSCG00000006385 | NCSTN | nicastrin [Source:VGNC Symbol;Acc:VGNC:90621] |
| ENSSSCG00000026861 | NECTIN4 | nectin cell adhesion molecule 4 [Source:VGNC Symbol;Acc:VGNC:90664] |
| ENSSSCG00000025085 | NEGR1 | neuronal growth regulator 1 [Source:VGNC Symbol;Acc:VGNC:90671] |
| ENSSSCG00000029578 | NFATC3 | nuclear factor of activated T cells 3 [Source:VGNC Symbol;Acc:VGNC:90710] |
| ENSSSCG00000010994 | NFX1 | nuclear transcription factor, X-box binding 1 [Source:VGNC Symbol;Acc:VGNC:96442] |
| ENSSSCG00000008816 | NFXL1 | nuclear transcription factor, X-box binding like 1 [Source:VGNC Symbol;Acc:VGNC:90728] |
| ENSSSCG00000037793 | NIT1 | nitrilase 1 [Source:VGNC Symbol;Acc:VGNC:90753] |
| ENSSSCG00000036607 | NKAIN2 | sodium/potassium transporting ATPase interacting 2 [Source:VGNC Symbol;Acc:VGNC:103141] |
| ENSSSCG00000027954 | NMU | neuromedin U [Source:VGNC Symbol;Acc:VGNC:90803] |
| ENSSSCG00000024596 | NOCT | nocturnin [Source:VGNC Symbol;Acc:VGNC:90810] |
| ENSSSCG00000008069 | NOL8 | nucleolar protein 8 [Source:VGNC Symbol;Acc:VGNC:90816] |
| ENSSSCG00000008111 | NPHP1 | nephrocystin 1 [Source:VGNC Symbol;Acc:VGNC:90848] |
| ENSSSCG00000037766 | NR3C2 | nuclear receptor subfamily 3 group C member 2 [Source:VGNC Symbol;Acc:VGNC:90884] |
| ENSSSCG00000023793 | NUP42 | nucleoporin 42 [Source:VGNC Symbol;Acc:VGNC:90981] |
| ENSSSCG00000009266 | ODAM | odontogenic, ameloblast associated [Source:VGNC Symbol;Acc:VGNC:91015] |
| ENSSSCG00000034607 | OGN | osteoglycin [Source:VGNC Symbol;Acc:VGNC:91028] |
| ENSSSCG00000008073 | OMD | osteomodulin [Source:VGNC Symbol;Acc:VGNC:91040] |
| ENSSSCG00000004540 | ONECUT2 | one cut homeobox 2 [Source:VGNC Symbol;Acc:VGNC:91043] |
| ENSSSCG00000006423 | OR6N2 | olfactory receptor family 6 subfamily N member 2 [Source:HGNC Symbol;Acc:HGNC:15035] |
| ENSSSCG00000004306 | ORC3 | origin recognition complex subunit 3 [Source:VGNC Symbol;Acc:VGNC:91061] |
| ENSSSCG00000016044 | ORMDL1 | ORMDL sphingolipid biosynthesis regulator 1 [Source:HGNC Symbol;Acc:HGNC:16036] |
| ENSSSCG00000016043 | OSGEPL1 | O-sialoglycoprotein endopeptidase like 1 [Source:VGNC Symbol;Acc:VGNC:95988] |
| ENSSSCG00000022442 | PAF1 | PAF1 homolog, Paf1/RNA polymerase II complex component [Source:VGNC Symbol;Acc:VGNC:91150] |
| ENSSSCG00000010437 | PAPSS2 | 3'-phosphoadenosine 5'-phosphosulfate synthase 2 [Source:VGNC Symbol;Acc:VGNC:91173] |
| ENSSSCG00000010027 | PATZ1 | POZ/BTB and AT hook containing zinc finger 1 [Source:VGNC Symbol;Acc:VGNC:91190] |
| ENSSSCG00000036086 | PAX5 | paired box 5 [Source:VGNC Symbol;Acc:VGNC:91194] |
| ENSSSCG00000010627 | PDCD4 | programmed cell death 4 [Source:VGNC Symbol;Acc:VGNC:91244] |
| ENSSSCG00000008908 | PDCL2 | phosducin like 2 [Source:VGNC Symbol;Acc:VGNC:91246] |
| ENSSSCG00000016929 | PDE4D | phosphodiesterase 4D [Source:VGNC Symbol;Acc:VGNC:91256] |
| ENSSSCG00000009106 | PDE5A | phosphodiesterase 5A [Source:VGNC Symbol;Acc:VGNC:91257] |
| ENSSSCG00000024960 | PDGFC | platelet derived growth factor C [Source:VGNC Symbol;Acc:VGNC:91266] |
| ENSSSCG00000023112 | PDS5A | PDS5 cohesin associated factor A [Source:HGNC Symbol;Acc:HGNC:29088] |
| ENSSSCG00000006366 | PFDN2 | prefoldin subunit 2 [Source:VGNC Symbol;Acc:VGNC:91333] |
| ENSSSCG00000005243 | PGM5 | phosphoglucomutase 5 [Source:VGNC Symbol;Acc:VGNC:91358] |
| ENSSSCG00000013067 | PHEROC | pheromaxein C subunit [Source:NCBI gene (formerly Entrezgene);Acc:100144470] |
| ENSSSCG00000035259 | PHF3 | PHD finger protein 3 [Source:VGNC Symbol;Acc:VGNC:91387] |
| ENSSSCG00000010092 | PI4KA | phosphatidylinositol 4-kinase alpha [Source:VGNC Symbol;Acc:VGNC:98191] |
| ENSSSCG00000004958 | PIAS1 | protein inhibitor of activated STAT 1 [Source:VGNC Symbol;Acc:VGNC:91410] |
| ENSSSCG00000003749 | PIK3C3 | phosphatidylinositol 3-kinase catalytic subunit type 3 [Source:VGNC Symbol;Acc:VGNC:91439] |
| ENSSSCG00000010026 | PIK3IP1 | phosphoinositide-3-kinase interacting protein 1 [Source:VGNC Symbol;Acc:VGNC:91444] |
| ENSSSCG00000030547 | PIP5K1A | phosphatidylinositol-4-phosphate 5-kinase type 1 alpha [Source:VGNC Symbol;Acc:VGNC:98834] |
| ENSSSCG00000002290 | PLEK2 | pleckstrin 2 [Source:VGNC Symbol;Acc:VGNC:91532] |
| ENSSSCG00000016045 | PMS1 | PMS1 homolog 1, mismatch repair system component [Source:VGNC Symbol;Acc:VGNC:98205] |
| ENSSSCG00000015332 | PON1 | paraoxonase 1 [Source:VGNC Symbol;Acc:VGNC:91661] |
| ENSSSCG00000029515 | PON3 | paraoxonase 3 [Source:NCBI gene (formerly Entrezgene);Acc:733674] |
| ENSSSCG00000037132 | POU2F2 | POU class 2 homeobox 2 [Source:VGNC Symbol;Acc:VGNC:91673] |
| ENSSSCG00000010083 | PPIL2 | peptidylprolyl isomerase like 2 [Source:VGNC Symbol;Acc:VGNC:91698] |
| ENSSSCG00000010079 | PPM1F | protein phosphatase, Mg2+/Mn2+ dependent 1F [Source:VGNC Symbol;Acc:VGNC:91706] |
| ENSSSCG00000040920 | PRKACB | protein kinase cAMP-activated catalytic subunit beta [Source:VGNC Symbol;Acc:VGNC:91800] |
| ENSSSCG00000010429 | PRKG1 | protein kinase cGMP-dependent 1 [Source:VGNC Symbol;Acc:VGNC:91816] |
| ENSSSCG00000006641 | PRUNE1 | prune exopolyphosphatase 1 [Source:VGNC Symbol;Acc:VGNC:91890] |
| ENSSSCG00000006628 | PSMD4 | proteasome 26S subunit, non-ATPase 4 [Source:VGNC Symbol;Acc:VGNC:98839] |
| ENSSSCG00000005227 | PUM3 | pumilio RNA binding family member 3 [Source:VGNC Symbol;Acc:VGNC:92003] |
| ENSSSCG00000036420 | PXT1 | peroxisomal testis enriched protein 1 [Source:VGNC Symbol;Acc:VGNC:92016] |
| ENSSSCG00000006149 | RALYL | RALY RNA binding protein like [Source:HGNC Symbol;Acc:HGNC:27036] |
| ENSSSCG00000004303 | RARS2 | arginyl-tRNA synthetase 2, mitochondrial [Source:VGNC Symbol;Acc:VGNC:92100] |
| ENSSSCG00000009977 | RASL10A | RAS like family 10 member A [Source:VGNC Symbol;Acc:VGNC:92117] |
| ENSSSCG00000007917 | RBFOX1 | RNA binding fox-1 homolog 1 [Source:VGNC Symbol;Acc:VGNC:92139] |
| ENSSSCG00000008994 | RBM46 | RNA binding motif protein 46 [Source:VGNC Symbol;Acc:VGNC:92157] |
| ENSSSCG00000025703 | REV3L | REV3 like, DNA directed polymerase zeta catalytic subunit [Source:VGNC Symbol;Acc:VGNC:92226] |
| ENSSSCG00000007855 | REXO5 | RNA exonuclease 5 [Source:VGNC Symbol;Acc:VGNC:92230] |
| ENSSSCG00000008788 | RFC1 | replication factor C subunit 1 [Source:VGNC Symbol;Acc:VGNC:92231] |
| ENSSSCG00000004589 | RNF111 | ring finger protein 111 [Source:VGNC Symbol;Acc:VGNC:92346] |
| ENSSSCG00000025855 | RNMT | RNA guanine-7 methyltransferase [Source:VGNC Symbol;Acc:VGNC:92402] |
| ENSSSCG00000026978 | ROS1 | ROS proto-onco 1, receptor tyrosine kinase [Source:VGNC Symbol;Acc:VGNC:92411] |
| ENSSSCG00000025514 | RUFY3 | RUN and FYVE domain containing 3 [Source:VGNC Symbol;Acc:VGNC:98959] |
| ENSSSCG00000010706 | SAMD4B | sterile alpha motif domain containing 4B [Source:VGNC Symbol;Acc:VGNC:98610] |
| ENSSSCG00000008836 | SCFD2 | sec1 family domain containing 2 [Source:VGNC Symbol;Acc:VGNC:98301] |
| ENSSSCG00000013068 | SCGB1D1 | secretoglobin family 1D member 1 [Source:NCBI gene (formerly Entrezgene);Acc:449006] |
| ENSSSCG00000015353 | SCIN | scinderin [Source:NCBI gene (formerly Entrezgene);Acc:100512981] |
| ENSSSCG00000033392 | SCML4 | Scm polycomb group protein like 4 [Source:VGNC Symbol;Acc:VGNC:92629] |
| ENSSSCG00000004382 | SEC63 | SEC63 homolog, protein translocation regulator [Source:VGNC Symbol;Acc:VGNC:103172] |
| ENSSSCG00000020702 | SENP6 | SUMO specific peptidase 6 [Source:VGNC Symbol;Acc:VGNC:92715] |
| ENSSSCG00000016969 | SERF1A | small EDRK-rich factor 1A [Source:HGNC Symbol;Acc:HGNC:10755] |
| ENSSSCG00000004490 | SETBP1 | SET binding protein 1 [Source:VGNC Symbol;Acc:VGNC:92756] |
| ENSSSCG00000010628 | SHOC2 | SHOC2 leucine rich repeat scaffold protein [Source:VGNC Symbol;Acc:VGNC:98318] |
| ENSSSCG00000011735 | SI | sucrase-isomaltase [Source:VGNC Symbol;Acc:VGNC:92863] |
| ENSSSCG00000004493 | SIGLEC15 | sialic acid binding Ig like lectin 15 [Source:VGNC Symbol;Acc:VGNC:92871] |
| ENSSSCG00000010169 | SIPA1L2 | signal induced proliferation associated 1 like 2 [Source:VGNC Symbol;Acc:VGNC:92883] |
| ENSSSCG00000008821 | SLAIN2 | SLAIN motif family member 2 [Source:VGNC Symbol;Acc:VGNC:92910] |
| ENSSSCG00000009035 | SLC10A7 | solute carrier family 10 member 7 [Source:VGNC Symbol;Acc:VGNC:92919] |
| ENSSSCG00000005222 | SLC1A1 | solute carrier family 1 member 1 [Source:VGNC Symbol;Acc:VGNC:92963] |
| ENSSSCG00000015336 | SLC25A13 | solute carrier family 25 member 13 [Source:VGNC Symbol;Acc:VGNC:92992] |
| ENSSSCG00000010960 | SLC28A3 | solute carrier family 28 member 3 [Source:VGNC Symbol;Acc:VGNC:95810] |
| ENSSSCG00000008801 | SLC30A9 | solute carrier family 30 member 9 [Source:VGNC Symbol;Acc:VGNC:98964] |
| ENSSSCG00000004304 | SLC35A1 | solute carrier family 35 member A1 [Source:VGNC Symbol;Acc:VGNC:93067] |
| ENSSSCG00000006871 | SLC35A3 | solute carrier family 35 member A3 [Source:VGNC Symbol;Acc:VGNC:98860] |
| ENSSSCG00000004250 | SLC35F1 | solute carrier family 35 member F1 [Source:VGNC Symbol;Acc:VGNC:93081] |
| ENSSSCG00000003777 | SLC44A5 | solute carrier family 44 member 5 [Source:VGNC Symbol;Acc:VGNC:93121] |
| ENSSSCG00000008943 | SLC4A4 | solute carrier family 4 member 4 [Source:VGNC Symbol;Acc:VGNC:93133] |
| ENSSSCG00000007954 | SLX4 | SLX4 structure-specific endonuclease subunit [Source:VGNC Symbol;Acc:VGNC:93215] |
| ENSSSCG00000009040 | SMAD1 | SMAD family member 1 [Source:VGNC Symbol;Acc:VGNC:93216] |
| ENSSSCG00000005232 | SMARCA2 | SWI/SNF related, matrix associated, actin dependent regulator of chromatin, subfamily a, member 2 [Source:VGNC Symbol;Acc:VGNC:93226] |
| ENSSSCG00000004300 | SMIM8 | small integral membrane protein 8 [Source:VGNC Symbol;Acc:VGNC:93256] |
| ENSSSCG00000010094 | SNAP29 | synaptosome associated protein 29 [Source:VGNC Symbol;Acc:VGNC:93278] |
| ENSSSCG00000018233 | SNORA62 | Small nucleolar RNA SNORA62/SNORA6 family [Source:RFAM;Acc:RF00091] |
| ENSSSCG00000019921 | SNORD22 | Small nucleolar RNA SNORD22 [Source:RFAM;Acc:RF00099] |
| ENSSSCG00000000567 | SOX5 | SRY-box transcription factor 5 [Source:VGNC Symbol;Acc:VGNC:93357] |
| ENSSSCG00000008834 | SPATA18 | spermatosis associated 18 [Source:VGNC Symbol;Acc:VGNC:98966] |
| ENSSSCG00000004544 | SPG21 | SPG21 abhydrolase domain containing, maspardin [Source:VGNC Symbol;Acc:VGNC:93403] |
| ENSSSCG00000006277 | SPIDR | scaffold protein involved in DNA repair [Source:VGNC Symbol;Acc:VGNC:93406] |
| ENSSSCG00000009083 | SPRY1 | sprouty RTK signaling antagonist 1 [Source:VGNC Symbol;Acc:VGNC:93424] |
| ENSSSCG00000036293 | SRSF10 | serine and arginine rich splicing factor 10 [Source:VGNC Symbol;Acc:VGNC:93472] |
| ENSSSCG00000038971 | ST8SIA3 | ST8 alpha-N-acetyl-neuraminide alpha-2,8-sialyltransferase 3 [Source:VGNC Symbol;Acc:VGNC:93518] |
| ENSSSCG00000008923 | STAP1 | signal transducing adaptor family member 1 [Source:VGNC Symbol;Acc:VGNC:93528] |
| ENSSSCG00000010605 | STN1 | STN1 subunit of CST complex [Source:VGNC Symbol;Acc:VGNC:93564] |
| ENSSSCG00000009111 | SYNPO2 | synaptopodin 2 [Source:VGNC Symbol;Acc:VGNC:93672] |
| ENSSSCG00000001533 | TAF11 | TATA-box binding protein associated factor 11 [Source:NCBI gene (formerly Entrezgene);Acc:100151814] |
| ENSSSCG00000004290 | TBX18 | T-box transcription factor 18 [Source:VGNC Symbol;Acc:VGNC:103187] |
| ENSSSCG00000004535 | TCF4 | transcription factor 4 [Source:VGNC Symbol;Acc:VGNC:93823] |
| ENSSSCG00000005389 | TEX10 | testis expressed 10 [Source:VGNC Symbol;Acc:VGNC:98364] |
| ENSSSCG00000027646 | TIPARP | TCDD inducible poly(ADP-ribose) polymerase [Source:HGNC Symbol;Acc:HGNC:23696] |
| ENSSSCG00000026079 | TKTL2 | transketolase like 2 [Source:VGNC Symbol;Acc:VGNC:98968] |
| ENSSSCG00000008890 | TMA16 | translation machinery associated 16 homolog [Source:VGNC Symbol;Acc:VGNC:94038] |
| ENSSSCG00000008192 | TMEM131 | transmembrane protein 131 [Source:VGNC Symbol;Acc:VGNC:94078] |
| ENSSSCG00000009012 | TMEM154 | transmembrane protein 154 [Source:VGNC Symbol;Acc:VGNC:94098] |
| ENSSSCG00000032234 | TMEM165 | transmembrane protein 165 [Source:VGNC Symbol;Acc:VGNC:94104] |
| ENSSSCG00000015487 | TNFSF18 | TNF superfamily member 18 [Source:VGNC Symbol;Acc:VGNC:94272] |
| ENSSSCG00000009100 | TNIP3 | TNFAIP3 interacting protein 3 [Source:VGNC Symbol;Acc:VGNC:94277] |
| ENSSSCG00000007235 | TPX2 | TPX2 microtubule nucleation factor [Source:VGNC Symbol;Acc:VGNC:95559] |
| ENSSSCG00000007952 | TRAP1 | TNF receptor associated protein 1 [Source:VGNC Symbol;Acc:VGNC:94371] |
| ENSSSCG00000004143 | TXLNB | taxilin beta [Source:VGNC Symbol;Acc:VGNC:94601] |
| ENSSSCG00000019589 | U1 | U1 spliceosomal RNA [Source:RFAM;Acc:RF00003] |
| ENSSSCG00000027893 | U6 | U6 spliceosomal RNA [Source:RFAM;Acc:RF00026] |
| ENSSSCG00000008924 | UBA6 | ubiquitin like modifier activating enzyme 6 [Source:VGNC Symbol;Acc:VGNC:94630] |
| ENSSSCG00000033602 | UBE2K | ubiquitin conjugating enzyme E2 K [Source:VGNC Symbol;Acc:VGNC:94646] |
| ENSSSCG00000000908 | UBE2NL | ubiquitin conjugating enzyme E2 N like (gene/pseudogene) [Source:HGNC Symbol;Acc:HGNC:31710] |
| ENSSSCG00000040265 | UBFD1 | ubiquitin family domain containing 1 [Source:VGNC Symbol;Acc:VGNC:94658] |
| ENSSSCG00000001532 | UHRF1BP1 | UHRF1 binding protein 1 [Source:VGNC Symbol;Acc:VGNC:94690] |
| ENSSSCG00000004614 | UNC13C | unc-13 homolog C [Source:VGNC Symbol;Acc:VGNC:94702] |
| ENSSSCG00000022206 | UOX | urate oxidase [Source:NCBI gene (formerly Entrezgene);Acc:397510] |
| ENSSSCG00000024623 | USP25 | ubiquitin specific peptidase 25 [Source:VGNC Symbol;Acc:VGNC:94755] |
| ENSSSCG00000030424 | USP31 | ubiquitin specific peptidase 31 [Source:HGNC Symbol;Acc:HGNC:20060] |
| ENSSSCG00000005300 | VCP | valosin containing protein [Source:NCBI gene (formerly Entrezgene);Acc:397524] |
| ENSSSCG00000004255 | VGLL2 | vestigial like family member 2 [Source:VGNC Symbol;Acc:VGNC:94822] |
| ENSSSCG00000036431 | VPS13C | vacuolar protein sorting 13 homolog C [Source:VGNC Symbol;Acc:VGNC:94840] |
| ENSSSCG00000011013 | WAC | WW domain containing adaptor with coiled-coil [Source:VGNC Symbol;Acc:VGNC:95845] |
| ENSSSCG00000008789 | WDR19 | WD repeat domain 19 [Source:VGNC Symbol;Acc:VGNC:94909] |
| ENSSSCG00000004615 | WDR72 | WD repeat domain 72 [Source:VGNC Symbol;Acc:VGNC:94938] |
| ENSSSCG00000028056 | ZFP36 | ZFP36 ring finger protein [Source:VGNC Symbol;Acc:VGNC:95145] |
| ENSSSCG00000035281 | ZNF292 | zinc finger protein 292 [Source:VGNC Symbol;Acc:VGNC:95218] |
| ENSSSCG00000021371 | ZNF300 | zinc finger protein 300 [Source:VGNC Symbol;Acc:VGNC:98424] |
| ENSSSCG00000033971 | ZNF407 | zinc finger protein 407 [Source:VGNC Symbol;Acc:VGNC:103207] |
| ENSSSCG00000008066 | ZNF484 | zinc finger protein 484 [Source:VGNC Symbol;Acc:VGNC:95249] |
| ENSSSCG00000028054 | ZNF526 | zinc finger protein 526 [Source:VGNC Symbol;Acc:VGNC:95260] |

**Table S11.** KEGG analysis of the selected genes of Anhui pig population versus commercial pig population

| ID | #Term | P-Value | Input |
| --- | --- | --- | --- |
| ssc04916 | Melanogenesis | 2.74521E-05 | FZD1|GNAQ|GNAO1|ADCY9|KIT|MAPK1|PRKACB|CREBBP |
| ssc04520 | Adherens junction | 0.000193894 | IGF1R|INSR|MAPK1|NECTIN4|CREBBP|CTNNA3 |
| ssc04120 | Ubiquitin mediated proteolysis | 0.00020195 | CBLB|UBE2K|UBA6|HERC1|PPIL2|PIAS1|CUL2|FBXW7 |
| ssc04015 | Rap1 signaling pathway | 0.000203188 | IGF1R|PDGFC|GNAQ|CRKL|GNAO1|ADCY9|KIT|INSR|MAPK1|SIPA1L2 |
| ssc04726 | Serotonergic synapse | 0.000368513 | HTR1E|GNAQ|GNAO1|MAPK1|CYP2C42|PRKACB|GABRB1 |
| ssc04540 | Gap junction | 0.00055319 | PDGFC|GNAQ|ADCY9|MAPK1|PRKG1|PRKACB |
| ssc04727 | GABAergic synapse | 0.000620973 | GNAO1|ADCY9|PRKACB|GABRA4|GABRB1|GPHN |
| ssc04730 | Long-term depression | 0.000693131 | GNAO1|IGF1R|MAPK1|PRKG1|GNAQ |
| ssc04666 | Fc gamma R-mediated phagocytosis | 0.000695023 | CRKL|LIMK2|PIP5K1A|DOCK2|MAPK1|SCIN |
| ssc05032 | Morphine addiction | 0.000734519 | GNAO1|ADCY9|PRKACB|GABRA4|PDE4D|GABRB1 |
| ssc04022 | cGMP-PKG signaling pathway | 0.000814781 | NFATC3|GNAQ|ADCY9|CNGA1|INSR|MAPK1|PRKG1|PDE5A |
| ssc04140 | Autophagy - animal | 0.000824308 | IGF1R|SNAP29|MAPK1|ATG7|PRKACB|PIK3C3|EIF2S1 |
| ssc04713 | Circadian entrainment | 0.0009587 | GNAQ|GNAO1|ADCY9|MAPK1|PRKG1|PRKACB |
| ssc05211 | Renal cell carcinoma | 0.001353095 | CRKL|MAPK1|CUL2|CREBBP|GAB1 |
| ssc04014 | Ras signaling pathway | 0.001494665 | EXOC2|IGF1R|PDGFC|INSR|GAB1|SHOC2|MAPK1|PRKACB|KIT |
| ssc04723 | Retrograde endocannabinoid signaling | 0.00164073 | GNAQ|GNAO1|ADCY9|MAPK1|PRKACB|GABRA4|GABRB1 |
| ssc04072 | Phospholipase D signaling pathway | 0.00170333 | PDGFC|PIP5K1A|ADCY9|GAB1|INSR|MAPK1|KIT |
| ssc04010 | MAPK signaling pathway | 0.001923402 | IGF1R|PDGFC|CRKL|KIT|INSR|NFATC3|PRKACB|MAPK1|MAPKAPK2|IL1R1 |
| ssc04724 | Glutamatergic synapse | 0.001954025 | GNAQ|GNAO1|ADCY9|MAPK1|PRKACB|SLC1A1 |
| ssc04970 | Salivary secretion | 0.002033057 | PRKACB|PRKG1|GNAQ|LYZ|ADCY9 |
| ssc01521 | EGFR tyrosine kinase inhibitor resistance | 0.002389939 | MAPK1|IGF1R|PDGFC|AXL|GAB1 |
| ssc04913 | Ovarian steroidogenesis | 0.002514778 | IGF1R|ADCY9|INSR|PRKACB |
| ssc05163 | Human cytomegalovirus infection | 0.003719617 | NFATC3|GNAQ|CRKL|GNAO1|ADCY9|MAPK1|PRKACB|IL1R1 |
| ssc00230 | Purine metabolism | 0.004152851 | PAPSS2|UOX|ADCY9|PRUNE1|PDE4D|PDE5A |
| ssc04923 | Regulation of lipolysis in adipocytes | 0.004272477 | INSR|PRKG1|ADCY9|PRKACB |
| ssc04213 | Longevity regulating pathway - multiple species | 0.006033722 | IGF1R|ADCY9|INSR|PRKACB |
| ssc04720 | Long-term potentiation | 0.007071838 | MAPK1|GNAQ|CREBBP|PRKACB |
| ssc04928 | Parathyroid hormone synthesis, secretion and action | 0.007615363 | MAPK1|PRKACB|PDE4D|GNAQ|ADCY9 |
| ssc04921 | Oxytocin signaling pathway | 0.007796867 | NFATC3|GNAQ|GNAO1|ADCY9|MAPK1|PRKACB |
| ssc04066 | HIF-1 signaling pathway | 0.00882835 | IGF1R|MAPK1|INSR|CUL2|CREBBP |
| ssc05170 | Human immunodeficiency virus 1 infection | 0.009772479 | NFATC3|GNAQ|CRKL|LIMK2|GNAO1|MAPK1|AP1B1 |
| ssc04918 | Thyroid hormone synthesis | 0.009935719 | PRKACB|ALB|GNAQ|ADCY9 |
| ssc04960 | Aldosterone-regulated sodium reabsorption | 0.010021096 | NR3C2|INSR|MAPK1 |
| ssc04725 | Cholinergic synapse | 0.010168672 | GNAO1|MAPK1|PRKACB|GNAQ|ADCY9 |
| ssc00562 | Inositol phosphate metabolism | 0.010397714 | PIP5K1A|PIK3C3|INPP4B|PI4KA |
| ssc04810 | Regulation of actin cytoskeleton | 0.010506179 | PDGFC|ITGA11|CRKL|LIMK2|PIP5K1A|MAPK1|SCIN |
| ssc05100 | Bacterial invasion of epithelial cells | 0.010873383 | GAB1|ARHGAP10|CRKL|CTNNA3 |
| ssc04024 | cAMP signaling pathway | 0.011816884 | ADCY9|CNGA1|MAPK1|PRKACB|HTR1E|PDE4D|CREBBP |
| ssc04611 | Platelet activation | 0.014114956 | MAPK1|PRKACB|PRKG1|GNAQ|ADCY9 |
| ssc05166 | Human T-cell leukemia virus 1 infection | 0.014153967 | NFATC3|ADCY9|ZFP36|MAPK1|CREBBP|PRKACB|IL1R1 |
| ssc04962 | Vasopressin-regulated water reabsorption | 0.014662861 | ADCY9|DCTN4|PRKACB |
| ssc04012 | ErbB signaling pathway | 0.017046581 | CBLB|MAPK1|CRKL|GAB1 |
| ssc05167 | Kaposi sarcoma-associated herpesvirus infection | 0.017121269 | NFATC3|ZFP36|MAPK1|PIK3C3|MAPKAPK2|CREBBP |
| ssc04961 | Endocrine and other factor-regulated calcium reabsorption | 0.017368139 | PRKACB|GNAQ|ADCY9 |
| ssc04270 | Vascular smooth muscle contraction | 0.01792893 | MAPK1|PRKACB|PRKG1|GNAQ|ADCY9 |
| ssc04915 | Estrogen signaling pathway | 0.018448936 | GNAO1|MAPK1|PRKACB|GNAQ|ADCY9 |
| ssc04914 | Progesterone-mediated oocyte maturation | 0.01973783 | IGF1R|MAPK1|ADCY9|PRKACB |
| ssc04371 | Apelin signaling pathway | 0.02006831 | PRKACB|PIK3C3|GNAQ|MAPK1|ADCY9 |
| ssc04211 | Longevity regulating pathway | 0.020449194 | IGF1R|ADCY9|INSR|PRKACB |
| ssc04912 | GnRH signaling pathway | 0.020449194 | MAPK1|PRKACB|GNAQ|ADCY9 |
| ssc04910 | Insulin signaling pathway | 0.020628105 | CBLB|INSR|MAPK1|CRKL|PRKACB |
| ssc01522 | Endocrine resistance | 0.021918773 | IGF1R|MAPK1|ADCY9|PRKACB |
| ssc05215 | Prostate cancer | 0.024241376 | MAPK1|IGF1R|PDGFC|CREBBP |
| ssc05231 | Choline metabolism in cancer | 0.025869381 | MAPK1|PIP5K1A|PDGFC|SLC44A5 |
| ssc04070 | Phosphatidylinositol signaling system | 0.026707441 | PIP5K1A|PIK3C3|INPP4B|PI4KA |
| ssc03430 | Mismatch repair | 0.026908726 | RFC1|LIG1 |
| ssc04750 | Inflammatory mediator regulation of TRP channels | 0.02756161 | PRKACB|GNAQ|IL1R1|ADCY9 |
| ssc05205 | Proteoglycans in cancer | 0.028550416 | FZD1|IGF1R|GAB1|MAPK1|PRKACB|PDCD4 |
| ssc04934 | Cushing syndrome | 0.030269643 | FZD1|PRKACB|GNAQ|MAPK1|ADCY9 |
| ssc04150 | mTOR signaling pathway | 0.030269643 | ATP6V1D|IGF1R|MAPK1|INSR|FZD1 |
| ssc05165 | Human papillomavirus infection | 0.030714609 | ATP6V1D|NFX1|MPP5|ITGA11|PRKACB|MAPK1|FZD1|CREBBP |
| ssc05200 | Pathways in cancer | 0.031568195 | FZD1|IGF1R|GNAQ|CRKL|ADCY9|KIT|MAPK1|CREBBP|PRKACB|CUL2|CTNNA3 |
| ssc04625 | C-type lectin receptor signaling pathway | 0.032075646 | CBLB|NFATC3|MAPKAPK2|MAPK1 |
| ssc04310 | Wnt signaling pathway | 0.034019408 | FZD1|NFATC3|INVS|CREBBP|PRKACB |
| ssc04927 | Cortisol synthesis and secretion | 0.03618758 | PRKACB|GNAQ|ADCY9 |
| ssc05225 | Hepatocellular carcinoma | 0.03804542 | FZD1|IGF1R|MAPK1|SMARCA2|GAB1 |
| ssc00970 | Aminoacyl-tRNA biosynthesis | 0.042035976 | MTFMT|RARS2|EARS2 |
| ssc04742 | Taste transduction | 0.043567784 | GABRA4|PRKACB|HTR1E |
| ssc04114 | Oocyte meiosis | 0.044579929 | IGF1R|MAPK1|ADCY9|PRKACB |
| ssc01100 | Metabolic pathways | 0.045149377 | ATP6V1D|EARS2|UOX|AACS|ACADM|PAPSS2|PI4KA|GCNT3|TKTL2|  INPP4B|CYP2C42|ASRGL1|B4GALT5|PDE5A|CYP2B22|DNMT3B|PIK3C3|  GPHN|PIP5K1A|ADCY9|SI|PRUNE1|PDE4D |
| ssc05218 | Melanoma | 0.046714 | MAPK1|IGF1R|PDGFC |
| ssc04976 | Bile secretion | 0.046714 | SLC4A4|ADCY9|PRKACB |
| ssc04919 | Thyroid hormone signaling pathway | 0.046894208 | MAPK1|DIO2|CREBBP|PRKACB |
| ssc04722 | Neurotrophin signaling pathway | 0.046894208 | MAPK1|MAPKAPK2|CRKL|GAB1 |
| ssc05202 | Transcriptional misregulation in cancer | 0.046939467 | PAX5|IGF1R|EWSR1|ETV7|SMAD1 |
| ssc00512 | Mucin type O-glycan biosynthesis | 0.049423134 | B4GALT5|GCNT3 |
| ssc04971 | Gastric acid secretion | 0.051637304 | PRKACB|GNAQ|ADCY9 |
| ssc04062 | Chemokine signaling pathway | 0.053840473 | DOCK2|MAPK1|ADCY9|CRKL|PRKACB |
| ssc04136 | Autophagy - other | 0.055099889 | PIK3C3|ATG7 |
| ssc05135 | Yersinia infection | 0.056805842 | MAPK1|PIP5K1A|NFATC3|CRKL |
| ssc05020 | Prion diseases | 0.058020178 | MAPK1|PRKACB |
| ssc04728 | Dopaminergic synapse | 0.058117957 | GNAO1|PRKACB|GNAQ|CLOCK |
| ssc04721 | Synaptic vesicle cycle | 0.058574738 | ATP6V1D|UNC13C|SLC1A1 |
| ssc05203 | Viral carcinogenesis | 0.060200005 | MAPK1|SCIN|MAPKAPK2|CREBBP|PRKACB |
| ssc04926 | Relaxin signaling pathway | 0.062150931 | GNAO1|MAPK1|ADCY9|PRKACB |
| ssc04068 | FoxO signaling pathway | 0.063527307 | IGF1R|MAPK1|INSR|CREBBP |
| ssc03030 | DNA replication | 0.064016801 | RFC1|LIG1 |
| ssc04550 | Signaling pathways regulating pluripotency of stem cells | 0.070647165 | FZD1|IGF1R|MAPK1|SMAD1 |
| ssc04510 | Focal adhesion | 0.072920081 | MAPK1|IGF1R|ITGA11|CRKL|PDGFC |
| ssc05033 | Nicotine addiction | 0.073379356 | GABRA4|GABRB1 |
| ssc04911 | Insulin secretion | 0.07367865 | PRKACB|GNAQ|ADCY9 |
| ssc04261 | Adrenergic signaling in cardiomyocytes | 0.082848449 | MAPK1|PRKACB|GNAQ|ADCY9 |
| ssc05224 | Breast cancer | 0.086050808 | FZD1|IGF1R|MAPK1|KIT |
| ssc04972 | Pancreatic secretion | 0.088161269 | SLC4A4|GNAQ|ADCY9 |
| ssc05146 | Amoebiasis | 0.088161269 | GNAQ|IL1R1|PRKACB |
| ssc05226 | Gastric cancer | 0.089312677 | FZD1|GAB1|MAPK1|CTNNA3 |
| ssc04350 | TGF-beta signaling pathway | 0.09032135 | MAPK1|SMAD1|CREBBP |
| ssc05414 | Dilated cardiomyopathy (DCM) | 0.092503422 | ITGA11|ADCY9|PRKACB |
| ssc04925 | Aldosterone synthesis and secretion | 0.092503422 | PRKACB|GNAQ|ADCY9 |
| ssc03420 | Nucleotide excision repair | 0.093287248 | RFC1|LIG1 |
| ssc04390 | Hippo signaling pathway | 0.094315505 | FZD1|SMAD1|MPP5|CTNNA3 |
| ssc05164 | Influenza A | 0.094315505 | MAPK1|EIF2S1|HNRNPUL1|CREBBP |
| ssc04930 | Type II diabetes mellitus | 0.10376062 | INSR|MAPK1 |
| ssc04330 | Notch signaling pathway | 0.10376062 | CREBBP|NCSTN |
| ssc04922 | Glucagon signaling pathway | 0.10837065 | GNAQ|CREBBP|PRKACB |
| ssc03460 | Fanconi anemia pathway | 0.110912354 | REV3L|SLX4 |
| ssc04141 | Protein processing in endoplasmic reticulum | 0.113749105 | VCP|ATF6|EIF2S1|SEC63 |
| ssc00280 | Valine, leucine and isoleucine degradation | 0.114535525 | AACS|ACADM |
| ssc00920 | Sulfur metabolism | 0.116142126 | PAPSS2 |
| ssc04714 | Thermogenesis | 0.122179425 | SMARCA2|PRKG1|ADCY9|KLB|PRKACB |
| ssc04660 | T cell receptor signaling pathway | 0.122745778 | CBLB|NFATC3|MAPK1 |
| ssc05142 | Chagas disease (American trypanosomiasis) | 0.125206503 | GNAO1|MAPK1|GNAQ |
| ssc04659 | Th17 cell differentiation | 0.125206503 | MAPK1|NFATC3|IL1R1 |
| ssc05010 | Alzheimer disease | 0.12696119 | MAPK1|ATF6|GNAQ|NCSTN |
| ssc04370 | VEGF signaling pathway | 0.133079602 | MAPK1|MAPKAPK2 |
| ssc04144 | Endocytosis | 0.136854359 | CBLB|IGF1R|SPG21|PIP5K1A|ARFGEF2 |
| ssc00830 | Retinol metabolism | 0.140677121 | CYP2C42|CYP2B22 |
| ssc05213 | Endometrial cancer | 0.144510335 | MAPK1|CTNNA3 |
| ssc04142 | Lysosome | 0.15338337 | DNASE2B|AP1B1|GGA2 |
| ssc04360 | Axon guidance | 0.155175161 | LIMK2|MAPK1|EPHA5|NFATC3 |
| ssc05204 | Chemical carcinogenesis | 0.156136147 | CYP2C42|CYP2B22 |
| ssc05230 | Central carbon metabolism in cancer | 0.163982501 | MAPK1|KIT |
| ssc05221 | Acute myeloid leukemia | 0.167931717 | MAPK1|KIT |
| ssc00450 | Selenocompound metabolism | 0.182946019 | PAPSS2 |
| ssc04151 | PI3K-Akt signaling pathway | 0.183067658 | IGF1R|PDGFC|ITGA11|KIT|INSR|MAPK1 |
| ssc04080 | Neuroactive ligand-receptor interaction | 0.184730444 | NMU|CNR2|HTR1E|GABRA4|MC5R|GABRB1 |
| ssc04924 | Renin secretion | 0.187904043 | GNAQ|PRKACB |
| ssc01524 | Platinum drug resistance | 0.187904043 | MAPK1|REV3L |
| ssc00511 | Other glycan degradation | 0.192069375 | FUCA1 |
| ssc00670 | One carbon pool by folate | 0.192069375 | MTFMT |
| ssc05214 | Glioma | 0.195983396 | IGF1R|MAPK1 |
| ssc05220 | Chronic myeloid leukemia | 0.208180102 | MAPK1|CRKL |
| ssc05412 | Arrhythmogenic right ventricular cardiomyopathy (ARVC) | 0.212263481 | ITGA11|CTNNA3 |
| ssc04662 | B cell receptor signaling pathway | 0.224557351 | MAPK1|NFATC3 |
| ssc04964 | Proximal tubule bicarbonate reclamation | 0.22755861 | SLC4A4 |
| ssc05160 | Hepatitis C | 0.232196493 | MAPK1|PIAS1|EIF2S1 |
| ssc03060 | Protein export | 0.23618541 | SEC63 |
| ssc00650 | Butanoate metabolism | 0.24471618 | AACS |
| ssc00790 | Folate biosynthesis | 0.24471618 | GPHN |
| ssc04218 | Cellular senescence | 0.255964135 | MAPK1|NFATC3|MAPKAPK2 |
| ssc03015 | mRNA surveillance pathway | 0.257548956 | CPSF6|RNMT |
| ssc04966 | Collecting duct acid secretion | 0.261493877 | ATP6V1D |
| ssc05161 | Hepatitis B | 0.261960183 | MAPK1|NFATC3|CREBBP |
| ssc04657 | IL-17 signaling pathway | 0.265818351 | USP25|MAPK1 |
| ssc04640 | Hematopoietic cell lineage | 0.265818351 | IL1R1|KIT |
| ssc04744 | Phototransduction | 0.2697429 | CNGA1 |
| ssc00030 | Pentose phosphate pathway | 0.277900085 | TKTL2 |
| ssc04658 | Th1 and Th2 cell differentiation | 0.278220857 | MAPK1|NFATC3 |
| ssc05152 | Tuberculosis | 0.283074464 | MAPK1|PIK3C3|CREBBP |
| ssc05235 | PD-L1 expression and PD-1 checkpoint pathway in cancer | 0.286482291 | MAPK1|NFATC3 |
| ssc04710 | Circadian rhythm | 0.293943003 | CLOCK |
| ssc05206 | MicroRNAs in cancer | 0.300265504 | DNMT3B|PDCD4|CRKL|CREBBP |
| ssc00052 | Galactose metabolism | 0.301830741 | SI |
| ssc00860 | Porphyrin and chlorophyll metabolism | 0.309630649 | EARS2 |
| ssc00500 | Starch and sucrose metabolism | 0.317343702 | SI |
| ssc03410 | Base excision repair | 0.317343702 | LIG1 |
| ssc04130 | SNARE interactions in vesicular transport | 0.317343702 | SNAP29 |
| ssc05145 | Toxoplasmosis | 0.327570082 | GNAO1|MAPK1 |
| ssc05143 | African trypanosomiasis | 0.33251309 | GNAQ |
| ssc04060 | Cytokine-cytokine receptor interaction | 0.337981688 | MSTN|TNFSF18|IL1RL1|IL1R1 |
| ssc05216 | Thyroid cancer | 0.33997132 | MAPK1 |
| ssc00250 | Alanine, aspartate and glutamate metabolism | 0.347346488 | ASRGL1 |
| ssc05219 | Bladder cancer | 0.354639515 | MAPK1 |
| ssc05016 | Huntington disease | 0.365196874 | DCTN4|GNAQ|CREBBP |
| ssc00071 | Fatty acid degradation | 0.376034823 | ACADM |
| ssc04216 | Ferroptosis | 0.383008307 | ATG7 |
| ssc04973 | Carbohydrate digestion and absorption | 0.383008307 | SI |
| ssc04020 | Calcium signaling pathway | 0.383338461 | PRKACB|GNAQ|ADCY9 |
| ssc04071 | Sphingolipid signaling pathway | 0.383918618 | MAPK1|GNAQ |
| ssc03022 | Basal transcription factors | 0.38990411 | TAF11 |
| ssc04340 | Hedgehog signaling pathway | 0.396723095 | PRKACB |
| ssc03050 | Proteasome | 0.396723095 | PSMD4 |
| ssc04110 | Cell cycle | 0.399659185 | ORC3|CREBBP |
| ssc04152 | AMPK signaling pathway | 0.399659185 | IGF1R|INSR |
| ssc04380 | Osteoclast differentiation | 0.411340823 | MAPK1|IL1R1 |
| ssc05030 | Cocaine addiction | 0.416727619 | PRKACB |
| ssc04940 | Type I diabetes mellitus | 0.416727619 | CPE |
| ssc00270 | Cysteine and methionine metabolism | 0.429695263 | DNMT3B |
| ssc00140 | Steroid hormone biosynthesis | 0.436070922 | CYP2B22 |
| ssc04210 | Apoptosis | 0.449436829 | MAPK1|EIF2S1 |
| ssc05162 | Measles | 0.456889672 | CBLB|EIF2S1 |
| ssc05134 | Legionellosis | 0.460870975 | VCP |
| ssc01212 | Fatty acid metabolism | 0.478754915 | ACADM |
| ssc04145 | Phagosome | 0.482508045 | ATP6V1D|PIK3C3 |
| ssc05034 | Alcoholism | 0.496809659 | GNAO1|MAPK1 |
| ssc00590 | Arachidonic acid metabolism | 0.507257034 | CYP2B22 |
| ssc04932 | Non-alcoholic fatty liver disease (NAFLD) | 0.507369721 | INSR|EIF2S1 |
| ssc04630 | Jak-STAT signaling pathway | 0.510857623 | PIAS1|CREBBP |
| ssc05217 | Basal cell carcinoma | 0.512768251 | FZD1 |
| ssc04664 | Fc epsilon RI signaling pathway | 0.518218028 | MAPK1 |
| ssc05223 | Non-small cell lung cancer | 0.518218028 | MAPK1 |
| ssc05031 | Amphetamine addiction | 0.518218028 | PRKACB |
| ssc05140 | Leishmaniasis | 0.528935986 | MAPK1 |
| ssc05168 | Herpes simplex virus 1 infection | 0.53182596 | POU2F2|ZNF484|EIF2S1|ZNF300 |
| ssc01230 | Biosynthesis of amino acids | 0.549664161 | TKTL2 |
| ssc04917 | Prolactin signaling pathway | 0.549664161 | MAPK1 |
| ssc05212 | Pancreatic cancer | 0.559684768 | MAPK1 |
| ssc04530 | Tight junction | 0.561198101 | MPP5|PRKACB |
| ssc05133 | Pertussis | 0.564611417 | MAPK1 |
| ssc03320 | PPAR signaling pathway | 0.574300497 | ACADM |
| ssc03008 | Ribosome biogenesis in eukaryotes | 0.588432628 | REXO5 |
| ssc05132 | Salmonella infection | 0.597593282 | MAPK1 |
| ssc04512 | ECM-receptor interaction | 0.615309371 | ITGA11 |
| ssc04974 | Protein digestion and absorption | 0.623873696 | SLC1A1 |
| ssc05210 | Colorectal cancer | 0.628084325 | MAPK1 |
| ssc05410 | Hypertrophic cardiomyopathy (HCM) | 0.636365159 | ITGA11 |
| ssc05323 | Rheumatoid arthritis | 0.636365159 | ATP6V1D |
| ssc04620 | Toll-like receptor signaling pathway | 0.660121159 | MAPK1 |
| ssc00564 | Glycerophospholipid metabolism | 0.660121159 | LYPLA2 |
| ssc04064 | NF-kappa B signaling pathway | 0.67509262 | IL1R1 |
| ssc04933 | AGE-RAGE signaling pathway in diabetic complications | 0.678731621 | MAPK1 |
| ssc04650 | Natural killer cell mediated cytotoxicity | 0.678731621 | MAPK1 |
| ssc04668 | TNF signaling pathway | 0.703092128 | MAPK1 |
| ssc04931 | Insulin resistance | 0.709707764 | INSR |
| ssc04670 | Leukocyte transendothelial migration | 0.712960278 | CTNNA3 |
| ssc01200 | Carbon metabolism | 0.719356741 | TKTL2 |
| ssc03040 | Spliceosome | 0.752080362 | SRSF10 |
| ssc00190 | Oxidative phosphorylation | 0.776003849 | ATP6V1D |
| ssc05418 | Fluid shear stress and atherosclerosis | 0.780999489 | IL1R1 |
| ssc05012 | Parkinson disease | 0.799895847 | PRKACB |
| ssc04514 | Cell adhesion molecules (CAMs) | 0.802140617 | NEGR1 |
| ssc04621 | NOD-like receptor signaling pathway | 0.812993524 | MAPK1 |
| ssc03013 | RNA transport | 0.815091832 | EIF2S1 |
| ssc05169 | Epstein-Barr virus infection | 0.890015213 | PSMD4 |
| ssc04740 | Olfactory transduction | 0.999923784 | PRKG1|PRKACB |

**Table S12.** GO analysis of the selected genes of Anhui pig population versus commercial pig population

| GO_accession | Description | P-Value | Input |
| --- | --- | --- | --- |
| GO:0005634 | nucleus | 8.54E-10 | AGL|RBFOX1|CLOCK|LIMK2|PSMD4|SYNPO2|RFC1|PPIL2|NFATC3|NOCT|SRSF10|USP31|HECA|USP25|RALYL|EIF2S1|MAPKAPK2|SOX5|KLF13|PPM1F|ELF2|MAB21L2|PXT1|DNASE1|PRKACB|LIG1|RNF111|TBX18|MBNL1|PDCD4|FEM1B|EIF4ENIF1|SLC30A9|RNMT|GABPB2|TCF4|PFDN2|NR3C2|MIER1|ZNF407|SMARCA2|VCP|VGLL2|REV3L|TIPARP|EBF2|FOXB1|PMS1|SCML4|UBA6|UBE2K|ZNF526|ETV7|ODAM|PDE4D|NFXL1|RBM46|BNIPL|MAPK1|PDS5A|PIAS1|STN1|REXO5|ATF6|POU2F2|EWSR1|ZNF292 |
| GO:0005654 | nucleoplasm | 2.55E-08 | NFATC3|CRKL|PSMD4|RFC1|PUM3|SPRY1|NAALADL2|SPIDR|TPX2|SLX4|HIC2|TMA16|STN1|MAPKAPK2|STAP1|RNF111|MBNL1|FEM1B|DNMT3B|NR3C2|NFX1|MIER1|BDP1|WAC|MLLT11|VCP|KLHL7|PATZ1|SENP6|ANKS1A|HNRNPUL1|CENPP|PAX5|DPH6|ETV7|ODAM|PDS5A|ONECUT2|SNAP29|SHOC2|SYNPO2|CSE1L|DEDD2|EWSR1 |
| GO:0005829 | cytosol | 7.33E-07 | NFATC3|IL1RL1|CRKL|PSMD4|ARL6|SPRY1|PFDN2|SRSF10|AACS|USP25|ELF2|EIF2S1|BNIPL|NFX1|DRG1|ANKS1A|SLX4|SPG21|PRKACB|RNF111|EIF4ENIF1|MBNL1|AP1B1|FEM1B|PDGFC|ARHGAP10|PPM1F|SAMD4B|PGM5|RUFY3|EVI5|MLLT11|VCP|KLHL7|SENP6|PIK3C3|PDCD4|GPHN|PAX5|LRBA|ODAM|LYST|CABP5|SNAP29|SHOC2|LSAMP|SYNPO2|PDE4D|SETBP1 |
| GO:0005737 | cytoplasm | 4.86E-06 | DOCK8|AGL|PI4KA|CLOCK|LIMK2|LIG1|LYPLA2|SMAD1|MSRB3|PRUNE1|MAB21L2|ANKIB1|CPSF6|MAPKAPK2|BNIPL|DRG1|CNR2|GAS2L1|KANK1|TKTL2|INPP4B|MPP5|AFG1L|MAPK1|CYP2C42|ATG7|RNF111|VGLL2|MBNL1|KCTD20|CYP2B22|PFDN2|RUFY3|SPATA18|TXLNB|VCP|EIF4ENIF1|KLHL2|HECA|PIK3C3|MC5R|GPHN|UBA6|UBE2K|RBFOX1|ODAM|ALB|METTL11B|PRKACB|SCIN|CREBBP|SRSF10 |
| GO:0004714 | transmembrane receptor protein tyrosine kinase activity | 1.93E-04 | EPHA5|IGF1R|ROS1|AXL|KIT |
| GO:0005794 | Golgi apparatus | 0.00023526 | CPE|GNAQ|ST8SIA3|B4GALT5|SLC35A3|ALB|TAF11|AKAP9|NCSTN|SPRY1|TMEM165|SLC35A1|MANEA|PRKG1|AP1B1|MAPRE1|FBXW7 |
| GO:0031625 | ubiquitin protein ligase binding | 0.000246682 | UBE2K|ASB4|FAF2|VCP|DIO2|PRKACB|USP25|CUL2|FBXW7 |
| GO:0030553 | cGMP binding | 0.000280888 | CNGA1|PRKG1|PDE5A |
| GO:0003677 | DNA binding | 0.000281859 | SETBP1|NR3C2|CLOCK|ALB|ORC3|RFC1|DNASE1|AKAP9|TAF11|REV3L|LIG1|SMARCA2|POU2F2|DEDD2|ZNF292 |
| GO:0005524 | ATP binding | 0.000308315 | EARS2|LIMK2|EPHA5|RFC1|DCLK2|INSR|RARS2|AACS|SMARCA2|ROS1|AXL|PAPSS2|TRAP1|MAPKAPK2|AFG1L|MAPK1|PRKG1|LIG1|KIF16B|IGF1R|VCP|PMS1|PIK3C3|UBE2K|PIP5K1A|ATAD1|KIT|PRKACB |
| GO:0016567 | protein ubiquitination | 0.000437488 | CBLB|ASB7|UBA6|HERC1|VCP|KLHL7|PPIL2|KLHL2|FEM1B|CUL2|FBXW7 |
| GO:0031116 | positive regulation of microtubule polymerization | 0.000566681 | DRG1|MAPRE1|AKAP9 |
| GO:0001228 | DNA-binding transcription activator activity, RNA polymerase II-specific | 0.000752695 | KLF13|TCF4|NFATC3|ONECUT2|SMAD1|ZNF300|PAX5|EBF2|TBX18|PATZ1|POU2F2|ZNF292 |
| GO:0046872 | metal ion binding | 0.000906982 | LIMK2|GNAO1|MSRB3|ANKIB1|OSGEPL1|PPM1F|CLYBL|MBNL1|EWSR1|B4GALT5|PDE5A|RUFY3|ZNF300|TIPARP|EBF2|GNAQ|ZNF484|CYB5A|KIT|ZFP36|PDE4D|PHF3 |
| GO:0021766 | hippocampus development | 0.000919022 | UBA6|EPHA5|CRKL|DCLK2 |
| GO:0006260 | DNA replication | 0.001218286 | LIG1|RFC1|SLX4|ORC3 |
| GO:0016020 | membrane | 0.00125003 | RASL10A|TMEM131|ARL6|GNAQ|GCNT3|SLC44A5|MAMDC2|SPRY1|CYB5A|SI|PI4KA|PIK3C3|LYST|LRBA |
| GO:0042802 | identical protein binding | 0.001577811 | BNIPL|IGF1R|CRKL|ALB|TNFSF18|CENPC|SMAD1|LYZ|SLC1A1|VCP|MSTN|KLHL2|ST8SIA3|DRG1|MAPRE1|EWSR1|FBXW7 |
| GO:0007283 | spermatogenesis | 0.001834088 | CABS1|CRKL|LIMK2|KIT|PATZ1|ROS1|AXL |
| GO:0016236 | macroautophagy | 0.002340413 | VCP|PIK3C3|ATG7 |
| GO:0005730 | nucleolus | 0.002351855 | NFX1|TMA16|DPH6|PUM3|ZNF300|KLHL7|REV3L|REXO5|NOL8|CENPP|DEDD2|EWSR1|FBXW7 |
| GO:0015183 | L-aspartate transmembrane transporter activity | 0.002511576 | SLC25A13|SLC1A1 |
| GO:1900028 | negative regulation of ruffle assembly | 0.002511576 | STAP1|KANK1 |
| GO:0034098 | VCP-NPL4-UFD1 AAA ATPase complex | 0.002511576 | VCP|FAF2 |
| GO:0008152 | metabolic process | 0.002511576 | SI|LYZ |
| GO:0003729 | mRNA binding | 0.002884188 | RBFOX1|RBM46|PUM3|NOCT|EIF4ENIF1|CPSF6|SAMD4B |
| GO:0007169 | transmembrane receptor protein tyrosine kinase signaling pathway | 0.002933916 | EPHA5|STAP1|ROS1|AXL|KIT |
| GO:0031593 | polyubiquitin modification-dependent protein binding | 0.003299858 | VCP|PSMD4|AGL |
| GO:0004064 | arylesterase activity | 0.003324131 | PON1|PON3 |
| GO:0006807 | nitrogen compound metabolic process | 0.003324131 | NIT1|SEC63 |
| GO:0005912 | adherens junction | 0.003396792 | PGM5|EPHA5|NECTIN4|MPP5|CTNNA3 |
| GO:0030335 | positive regulation of cell migration | 0.003451909 | IGF1R|PDGFC|ONECUT2|RUFY3|KIT|INSR |
| GO:0003723 | RNA binding | 0.003595239 | RBFOX1|RBM46|NOL8|SEC63|REXO5|SRSF10|MBNL1|RALYL|EIF2S1|EWSR1|HNRNPUL1|RNMT |
| GO:0060348 | bone development | 0.003665611 | PAPSS2|SMAD1|PDGFC |
| GO:0010629 | negative regulation of gene expression | 0.004092709 | GAS2L1|TIPARP|ROS1|CRKL|FBXW7 |
| GO:0045647 | negative regulation of erythrocyte differentiation | 0.004242469 | KLF13|GAS2L1 |
| GO:0019373 | epoxygenase P450 pathway | 0.004242469 | CYP2C42|CYP2B22 |
| GO:0008392 | arachidonic acid epoxygenase activity | 0.004242469 | CYP2C42|CYP2B22 |
| GO:0045893 | positive regulation of transcription, DNA-templated | 0.004248263 | FZD1|WAC|MSTN|MLLT11|INSR|RNF111|ELF2|CREBBP |
| GO:0001784 | phosphotyrosine residue binding | 0.004468902 | CBLB|STAP1|CRKL |
| GO:0052742 | phosphatidylinositol kinase activity | 0.005264143 | PIK3C3|PI4KA |
| GO:0038083 | peptidyl-tyrosine autophosphorylation | 0.005370774 | IGF1R|ROS1|INSR |
| GO:0043406 | positive regulation of MAP kinase activity | 0.005370774 | PDGFC|PDE5A|KIT |
| GO:0016604 | nuclear body | 0.005800499 | DRG1|CENPC|ORC3|ZNF300|SETBP1|ELF2|CREBBP |
| GO:0015629 | actin cytoskeleton | 0.005862845 | ONECUT2|KLHL2|SYNPO2|MYOZ2|AXL|SCIN |
| GO:0001655 | urogenital system development | 0.006386749 | FOXB1|CRKL |
| GO:0007568 | aging | 0.006386749 | PAX5|PDE4D |
| GO:0022010 | central nervous system myelination | 0.006386749 | B4GALT5|NCSTN |
| GO:0031994 | insulin-like growth factor I binding | 0.006386749 | IGF1R|INSR |
| GO:0007020 | microtubule nucleation | 0.006386749 | TPX2|AKAP9 |
| GO:0048015 | phosphatidylinositol-mediated signaling | 0.00748236 | IGF1R|PIK3C3|PI4KA |
| GO:0031462 | Cul2-RING ubiquitin ligase complex | 0.007607921 | ASB4|CUL2 |
| GO:0034450 | ubiquitin-ubiquitin ligase activity | 0.007607921 | UBE2K|PPIL2 |
| GO:0036092 | phosphatidylinositol-3-phosphate biosynthetic process | 0.007607921 | PIK3C3|INPP4B |
| GO:0051443 | positive regulation of ubiquitin-protein transferase activity | 0.007607921 | DCUN1D3|FBXW7 |
| GO:0007212 | dopamine receptor signaling pathway | 0.008925335 | GNAO1|NCSTN |
| GO:0022851 | GABA-gated chloride ion channel activity | 0.008925335 | GABRA4|GABRB1 |
| GO:0006497 | protein lipidation | 0.008925335 | PIK3C3|ATG7 |
| GO:0006082 | organic acid metabolic process | 0.008925335 | CYP2C42|CYP2B22 |
| GO:0031532 | actin cytoskeleton reorganization | 0.010021096 | KIT|PLEK2|GAB1 |
| GO:0010494 | cytoplasmic stress granule | 0.010021096 | VCP|MBNL1|EIF2S1 |
| GO:0005925 | focal adhesion | 0.010168672 | PGM5|ITGA11|MAPRE1|IL1RL1|SYNPO2 |
| GO:0032446 | protein modification by small protein conjugation | 0.010336706 | UBA6|ATG7 |
| GO:0004553 | hydrolase activity, hydrolyzing O-glycosyl compounds | 0.010336706 | SI|KLB |
| GO:0042738 | exogenous drug catabolic process | 0.010336706 | CYP2C42|CYP2B22 |
| GO:0043548 | phosphatidylinositol 3-kinase binding | 0.010336706 | IGF1R|INSR |
| GO:0071222 | cellular response to lipopolysaccharide | 0.010397714 | TNIP3|STAP1|PDE4D|AXL |
| GO:0016887 | ATPase activity | 0.010442743 | KIF16B|ATAD1|VCP|AFG1L|PMS1|SMARCA2 |
| GO:0048565 | digestive tract development | 0.011839786 | PDGFC|KIT |
| GO:0097193 | intrinsic apoptotic signaling pathway | 0.011839786 | PPM1F|MLLT11 |
| GO:0030970 | retrograde protein transport, ER to cytosol | 0.011839786 | VCP|FAF2 |
| GO:0032689 | negative regulation of interferon-gamma production | 0.011839786 | IL1RL1|AXL |
| GO:0050877 | nervous system process | 0.012215005 | GABRA4|WDR19|GABRB1 |
| GO:0018108 | peptidyl-tyrosine phosphorylation | 0.012915624 | IGF1R|EPHA5|AXL|KIT |
| GO:0008104 | protein localization | 0.013002542 | CEP128|LYST|LRBA |
| GO:0045944 | positive regulation of transcription by RNA polymerase II | 0.013136451 | KLF13|TCF4|NFATC3|GABPB2|ATF6|PAX5|TBX18|SLC30A9|EBF2|ZNF300|VGLL2|PATZ1|SMARCA2|ZNF292|POU2F2|CREBBP|SOX5 |
| GO:0030318 | melanocyte differentiation | 0.013432369 | KIT|LRMDA |
| GO:0046966 | thyroid hormone receptor binding | 0.013432369 | TAF11|GAS2L1 |
| GO:0002092 | positive regulation of receptor internalization | 0.013432369 | INSR|ATAD1 |
| GO:0030512 | negative regulation of transforming growth factor beta receptor signaling pathway | 0.013818451 | ONECUT2|ASPN|LEMD3 |
| GO:0001725 | stress fiber | 0.013818451 | PGM5|GAS2L1|SYNPO2 |
| GO:0019901 | protein kinase binding | 0.014053854 | EXOC2|TPX2|LRBA|TRAP1|LYST|SMAD1|AP1B1|MAPRE1 |
| GO:0005874 | microtubule | 0.014558745 | GAS2L1|KIF16B|TPX2|MAPRE1|DCLK2 |
| GO:0006413 | translational initiation | 0.014662861 | MTFMT|TAF11|EIF2S1 |
| GO:0046854 | phosphatidylinositol phosphorylation | 0.014662861 | PIP5K1A|PIK3C3|PI4KA |
| GO:1902711 | GABA-A receptor complex | 0.015112281 | GABRA4|GABRB1 |
| GO:0008395 | steroid hydroxylase activity | 0.015112281 | CYP2C42|CYP2B22 |
| GO:0045995 | regulation of embryonic development | 0.015112281 | INSR|NOCT |
| GO:0016712 | oxidoreductase activity, acting on paired donors, with incorporation or reduction of molecular oxygen, reduced flavin or flavoprotein as one donor, and incorporation of one atom of oxygen | 0.015112281 | CYP2C42|CYP2B22 |
| GO:0004890 | GABA-A receptor activity | 0.015112281 | GABRA4|GABRB1 |
| GO:0009267 | cellular response to starvation | 0.016437615 | GAS2L1|ALB|ATG7 |
| GO:0000407 | phagophore assembly site | 0.016877391 | PIK3C3|ATG7 |
| GO:0033137 | negative regulation of peptidyl-serine phosphorylation | 0.016877391 | PPM1F|PDE4D |
| GO:0005739 | mitochondrion | 0.017175696 | MTFMT|NIT1|PFDN2|BRI3BP|EARS2|TRAP1|LIG1|OSGEPL1|AFG1L|STAP1|NOCT|RARS2|ACADM|FBXW7 |
| GO:0046627 | negative regulation of insulin receptor signaling pathway | 0.0187256 | MSTN|KANK1 |
| GO:0043473 | pigmentation | 0.0187256 | LYST|KIT |
| GO:0045202 | synapse | 0.019009953 | GNAQ|VCP|UNC13C|HTR1E|GABRA4|EIF2S1|GABRB1 |
| GO:0033674 | positive regulation of kinase activity | 0.020333069 | EPHA5|AXL|KIT |
| GO:0000932 | P-body | 0.020333069 | EIF4ENIF1|SAMD4B|NOCT |
| GO:0030511 | positive regulation of transforming growth factor beta receptor signaling pathway | 0.020654847 | CREBBP|RNF111 |
| GO:0007214 | gamma-aminobutyric acid signaling pathway | 0.020654847 | GABRA4|GABRB1 |
| GO:0048008 | platelet-derived growth factor receptor signaling pathway | 0.020654847 | PDGFC|TIPARP |
| GO:0043065 | positive regulation of apoptotic process | 0.021198009 | PXT1|DCUN1D3|ATG7|MLLT11|SCIN |
| GO:0030534 | adult behavior | 0.022663105 | PAX5|NCSTN |
| GO:0031624 | ubiquitin conjugating enzyme binding | 0.022663105 | ANKIB1|DCUN1D3 |
| GO:0005795 | Golgi stack | 0.022663105 | LYPLA2|AKAP9 |
| GO:0043235 | receptor complex | 0.024201238 | NR3C2|IGF1R|EPHA5|AXL|KIT |
| GO:0019222 | regulation of metabolic process | 0.024748384 | CNR2|MC5R |
| GO:0031683 | G-protein beta/gamma-subunit complex binding | 0.024748384 | GNAO1|GNAQ |
| GO:0046718 | viral entry into host cell | 0.024748384 | NECTIN4|AXL |
| GO:0007026 | negative regulation of microtubule depolymerization | 0.024748384 | TPX2|GAS2L1 |
| GO:0048701 | embryonic cranial skeleton morphogenesis | 0.024748384 | PAX5|WDR19 |
| GO:0006814 | sodium ion transport | 0.024748384 | SLC4A4|SLC10A7 |
| GO:0120162 | positive regulation of cold-induced thermogenesis | 0.025854095 | PDGFC|EBF2|DIO2 |
| GO:0072686 | mitotic spindle | 0.025854095 | LIMK2|TPX2|ODAM |
| GO:0003682 | chromatin binding | 0.026203306 | CREBBP|TCF4|NFATC3|WAC|SLC30A9|EBF2|PATZ1|SCML4 |
| GO:0007189 | adenylate cyclase-activating G protein-coupled receptor signaling pathway | 0.026707441 | CNR2|GNAQ|MC5R|ADCY9 |
| GO:0043231 | intracellular membrane-bounded organelle | 0.026828945 | IGF1R|SPG21|SPATA18|CYB5A|CYP2C42|AXL|CYP2B22 |
| GO:0034237 | protein kinase A regulatory subunit binding | 0.026908726 | AKAP9|PRKACB |
| GO:0007595 | lactation | 0.026908726 | FOXB1|CSN3 |
| GO:0008584 | male gonad development | 0.027045181 | PATZ1|INSR|CRKL |
| GO:0043547 | positive regulation of GTPase activity | 0.02739807 | DOCK8|GNAQ|ODAM|ARHGAP31|ARHGAP10|SIPA1L2 |
| GO:0006974 | cellular response to DNA damage stimulus | 0.02756161 | VCP|UBA6|SPATA18|MAPKAPK2 |
| GO:0031514 | motile cilium | 0.028265147 | WDR19|CABS1|NPHP1 |
| GO:0007626 | locomotory behavior | 0.028265147 | NEGR1|GNAO1|UBA6 |
| GO:0000724 | double-strand break repair via homologous recombination | 0.028265147 | SPIDR|REV3L|SLX4 |
| GO:0000187 | activation of MAPK activity | 0.028265147 | INSR|CRKL|KIT |
| GO:0007165 | signal transduction | 0.02865057 | CBLB|NMU|RASL10A|AKAP9|SHOC2|ARHGAP10|GABRB1|GABRA4|PDE4D|PDE5A |
| GO:0001657 | ureteric bud development | 0.029142209 | SPRY1|SMAD1 |
| GO:0006805 | xenobiotic metabolic process | 0.029142209 | CYP2C42|CYP2B22 |
| GO:0010811 | positive regulation of cell-substrate adhesion | 0.029142209 | PPM1F|ECM2 |
| GO:0009636 | response to toxic substance | 0.029142209 | PON1|PON3 |
| GO:0003779 | actin binding | 0.030269643 | MYOZ2|GMFG|DNASE1|KLHL2|SYNPO2 |
| GO:0006954 | inflammatory response | 0.030269643 | KIT|ODAM|MAPKAPK2|AXL|SMAD1 |
| GO:0047485 | protein N-terminus binding | 0.030791486 | EXOC2|TAF11|DCTN4 |
| GO:0006511 | ubiquitin-dependent protein catabolic process | 0.030949918 | UBA6|RNF111|ANKIB1|USP31|USP25|CUL2 |
| GO:0001558 | regulation of cell growth | 0.031446943 | ROS1|CRKL |
| GO:0031334 | positive regulation of protein-containing complex assembly | 0.031446943 | VCP|SPIDR |
| GO:0001578 | microtubule bundle formation | 0.031446943 | GAS2L1|MAPRE1 |
| GO:0030217 | T cell differentiation | 0.031446943 | PATZ1|KIT |
| GO:0007612 | learning | 0.031446943 | UBA6|ATAD1 |
| GO:0005777 | peroxisome | 0.032097716 | PXT1|PIK3C3|UOX |
| GO:0004842 | ubiquitin-protein transferase activity | 0.033027352 | HERC1|NFX1|PPIL2|CUL2 |
| GO:0030018 | Z disc | 0.03343254 | MYOZ2|PGM5|SYNPO2 |
| GO:0071407 | cellular response to organic cyclic compound | 0.033821071 | TIPARP|SMAD1 |
| GO:0050873 | brown fat cell differentiation | 0.033821071 | EBF2|DIO2 |
| GO:0015293 | symporter activity | 0.033821071 | SLC28A3|SLC10A7 |
| GO:0006417 | regulation of translation | 0.033821071 | PUM3|EIF2S1 |
| GO:0000209 | protein polyubiquitination | 0.035980778 | ANKIB1|UBE2K|PPIL2|RNF111 |
| GO:0000381 | regulation of alternative mRNA splicing, via spliceosome | 0.03618758 | MBNL1|SRSF10|RBFOX1 |
| GO:0030332 | cyclin binding | 0.036262768 | FBXW7|KLHDC9 |
| GO:0032391 | photoreceptor connecting cilium | 0.036262768 | WDR19|LCA5 |
| GO:0000976 | transcription regulatory region sequence-specific DNA binding | 0.03803184 | KLF13|GABPB2|POU2F2|SOX5 |
| GO:0005667 | transcription regulator complex | 0.03804542 | TCF4|NFATC3|SMAD1|CREBBP|CLOCK |
| GO:0071560 | cellular response to transforming growth factor beta stimulus | 0.038770242 | CRKL|SOX5 |
| GO:0031463 | Cul3-RING ubiquitin ligase complex | 0.038770242 | KLHL7|KLHL2 |
| GO:0005801 | cis-Golgi network | 0.038770242 | LIMK2|AKAP9 |
| GO:0045211 | postsynaptic membrane | 0.039055729 | ATAD1|CLSTN2|GPHN |
| GO:0035556 | intracellular signal transduction | 0.039676747 | ASB7|ASB4|DCLK2|MAPK1|UNC13C|MAPKAPK2|PLEK2 |
| GO:0043161 | proteasome-mediated ubiquitin-dependent protein catabolic process | 0.041231825 | VCP|PSMD4|UBE2K|CUL2 |
| GO:0086091 | regulation of heart rate by cardiac conduction | 0.04134173 | AKAP9|CTNNA3 |
| GO:0048471 | perinuclear region of cytoplasm | 0.042259694 | VCP|KLHL7|DCUN1D3|NOCT|PDE4D|ROS1|FBXW7 |
| GO:0000978 | RNA polymerase II cis-regulatory region sequence-specific DNA binding | 0.042479029 | KLF13|TCF4|NFATC3|ZNF526|HIC2|CLOCK|ONECUT2|SMAD1|PAX5|EBF2|PATZ1 |
| GO:0016358 | dendrite development | 0.043975504 | PRKG1|CRKL |
| GO:0008144 | drug binding | 0.043975504 | PDE4D|ALB |
| GO:0042752 | regulation of circadian rhythm | 0.043975504 | NOCT|FBXW7 |
| GO:0034644 | cellular response to UV | 0.046669862 | EIF2S1|CREBBP |
| GO:0030594 | neurotransmitter receptor activity | 0.046714 | GABRA4|HTR1E|GABRB1 |
| GO:0005509 | calcium ion binding | 0.047295814 | CBLB|CABS1|CABP5|FSTL5|PON1|ASTN2|UNC13C|SLC25A13|ASPN|CLSTN2|SCIN |
| GO:0008017 | microtubule binding | 0.047891167 | KIF16B|TPX2|MAPRE1|DRG1|GAS2L1 |
| GO:0008284 | positive regulation of cell population proliferation | 0.048101775 | IGF1R|PDGFC|CRKL|KLB|MAB21L2|INSR |
| GO:0001764 | neuron migration | 0.048328079 | PRKG1|CRKL|AXL |
| GO:0032991 | protein-containing complex | 0.048921792 | PPM1F|CRKL|ALB|MPP5|STAP1|RNF111 |
| GO:0001889 | liver development | 0.049423134 | ONECUT2|SEC63 |
| GO:0007519 | skeletal muscle tissue development | 0.049423134 | VGLL2|MYOZ2 |

**Table S13.** The information of two variants located on CABS1

| #chr | start | end | ref | alt | structure_type | function_type | function_gene | Symbol |
| --- | --- | --- | --- | --- | --- | --- | --- | --- |
| 8 | 67162901 | 67162901 | G | T | exonic | synonymous SNV | ncbi_110262120:XM_021101416.1:exon1:c.G1047T:p.S349S, | CABS1 |
| 8 | 67162923 | 67162923 | C | G | exonic | nonsynonymous SNV | ncbi_110262120:XM_021101416.1:exon1:c.C1069G:p.L357V, | CABS1 |

Table S14. The information of variants located on INSL6

| #chr | start | end | ref | alt | structure_type | function_type | function_gene |
| --- | --- | --- | --- | --- | --- | --- | --- |
| 1 | 216793185 | 216793185 | C | T | intronic | - | - |
| 1 | 216793283 | 216793283 | A | G | intronic | - | - |
| 1 | 216793287 | 216793287 | A | G | intronic | - | - |
| 1 | 216793421 | 216793421 | A | C | intronic | - | - |
| 1 | 216793603 | 216793603 | A | C | intronic | - | - |
| 1 | 216793719 | 216793719 | C | T | intronic | - | - |
| 1 | 216793819 | 216793819 | T | C | intronic | - | - |
| 1 | 216793865 | 216793865 | G | A | intronic | - | - |
| 1 | 216793917 | 216793917 | T | A | intronic | - | - |
| 1 | 216794108 | 216794108 | A | G | intronic | - | - |
| 1 | 216794134 | 216794134 | G | A | intronic | - | - |
| 1 | 216794285 | 216794285 | A | T | intronic | - | - |
| 1 | 216794309 | 216794309 | G | T | intronic | - | - |
| 1 | 216794329 | 216794329 | T | A | intronic | - | - |
| 1 | 216794377 | 216794377 | G | A | intronic | - | - |
| 1 | 216794394 | 216794394 | C | T | intronic | - | - |
| 1 | 216794430 | 216794430 | T | A | intronic | - | - |
| 1 | 216794496 | 216794496 | C | T | intronic | - | - |
| 1 | 216794534 | 216794534 | T | C | intronic | - | - |
| 1 | 216794603 | 216794603 | C | T | intronic | - | - |
| 1 | 216794674 | 216794674 | A | C | intronic | - | - |
| 1 | 216794834 | 216794834 | G | A | intronic | - | - |
| 1 | 216794906 | 216794906 | T | A | intronic | - | - |
| 1 | 216794916 | 216794916 | A | G | intronic | - | - |
| 1 | 216794959 | 216794959 | T | G | intronic | - | - |
| 1 | 216794999 | 216794999 | A | G | intronic | - | - |
| 1 | 216795089 | 216795089 | G | C | intronic | - | - |
| 1 | 216795124 | 216795124 | G | A | intronic | - | - |
| 1 | 216795220 | 216795220 | C | T | intronic | - | - |
| 1 | 216795240 | 216795240 | C | T | intronic | - | - |
| 1 | 216795441 | 216795441 | C | T | intronic | - | - |
| 1 | 216795574 | 216795574 | G | A | intronic | - | - |
| 1 | 216795616 | 216795616 | G | T | intronic | - | - |
| 1 | 216795752 | 216795752 | G | A | intronic | - | - |
| 1 | 216795770 | 216795770 | G | T | intronic | - | - |
| 1 | 216795845 | 216795845 | T | C | intronic | - | - |
| 1 | 216795852 | 216795852 | A | G | intronic | - | - |
| 1 | 216795968 | 216795968 | C | G | intronic | - | - |
| 1 | 216795977 | 216795977 | T | C | intronic | - | - |
| 1 | 216796057 | 216796057 | C | T | intronic | - | - |
| 1 | 216796066 | 216796066 | A | G | intronic | - | - |
| 1 | 216796112 | 216796112 | C | T | intronic | - | - |
| 1 | 216796136 | 216796136 | T | A | intronic | - | - |
| 1 | 216796388 | 216796388 | T | C | intronic | - | - |
| 1 | 216796653 | 216796653 | C | G | intronic | - | - |
| 1 | 216796804 | 216796804 | G | A | intronic | - | - |
| 1 | 216796905 | 216796905 | C | G | intronic | - | - |
| 1 | 216796913 | 216796913 | A | G | intronic | - | - |
| 1 | 216797050 | 216797050 | T | G | intronic | - | - |
| 1 | 216797073 | 216797073 | G | T | intronic | - | - |
| 1 | 216797096 | 216797096 | A | G | intronic | - | - |
| 1 | 216797356 | 216797356 | C | A | intronic | - | - |
| 1 | 216797561 | 216797561 | A | G | intronic | - | - |
| 1 | 216797591 | 216797591 | G | T | intronic | - | - |
| 1 | 216797600 | 216797600 | T | G | intronic | - | - |
| 1 | 216797723 | 216797723 | A | G | intronic | - | - |
| 1 | 216797890 | 216797890 | A | G | intronic | - | - |
| 1 | 216797895 | 216797895 | C | A | intronic | - | - |
| 1 | 216798104 | 216798104 | T | C | intronic | - | - |
| 1 | 216798112 | 216798112 | C | G | intronic | - | - |
| 1 | 216798187 | 216798187 | G | A | intronic | - | - |
| 1 | 216798256 | 216798256 | C | T | intronic | - | - |
| 1 | 216798577 | 216798577 | C | T | intronic | - | - |
| 1 | 216798590 | 216798590 | T | A | intronic | - | - |
| 1 | 216798622 | 216798622 | T | C | intronic | - | - |
| 1 | 216798789 | 216798789 | A | T | intronic | - | - |
| 1 | 216798791 | 216798791 | T | C | intronic | - | - |
| 1 | 216798796 | 216798796 | C | T | intronic | - | - |
| 1 | 216798803 | 216798803 | C | G | intronic | - | - |
| 1 | 216798820 | 216798820 | T | C | intronic | - | - |
| 1 | 216798821 | 216798821 | G | A | intronic | - | - |
| 1 | 216798838 | 216798838 | G | A | intronic | - | - |
| 1 | 216798846 | 216798846 | A | G | intronic | - | - |
| 1 | 216798895 | 216798895 | C | T | intronic | - | - |
| 1 | 216798911 | 216798911 | C | T | intronic | - | - |
| 1 | 216798915 | 216798915 | G | C | intronic | - | - |
| 1 | 216798950 | 216798950 | G | A | intronic | - | - |
| 1 | 216799001 | 216799001 | G | A | intronic | - | - |
| 1 | 216799037 | 216799037 | A | G | intronic | - | - |
| 1 | 216799053 | 216799053 | A | G | intronic | - | - |
| 1 | 216799063 | 216799063 | C | T | intronic | - | - |
| 1 | 216799076 | 216799076 | T | C | intronic | - | - |
| 1 | 216799168 | 216799168 | C | A | intronic | - | - |
| 1 | 216799195 | 216799195 | T | C | intronic | - | - |
| 1 | 216799337 | 216799337 | A | G | intronic | - | - |
| 1 | 216799562 | 216799562 | G | T | intronic | - | - |
| 1 | 216799618 | 216799618 | A | G | intronic | - | - |
| 1 | 216799666 | 216799666 | G | T | intronic | - | - |
| 1 | 216799763 | 216799763 | T | C | intronic | - | - |
| 1 | 216799842 | 216799842 | A | T | intronic | - | - |
| 1 | 216799899 | 216799899 | A | G | intronic | - | - |
| 1 | 216799922 | 216799922 | T | C | intronic | - | - |
| 1 | 216800133 | 216800133 | A | G | intronic | - | - |
| 1 | 216800142 | 216800142 | A | G | intronic | - | - |
| 1 | 216800234 | 216800234 | C | T | intronic | - | - |
| 1 | 216800373 | 216800373 | C | T | intronic | - | - |
| 1 | 216800497 | 216800497 | T | A | intronic | - | - |
| 1 | 216800542 | 216800542 | G | T | intronic | - | - |
| 1 | 216800546 | 216800546 | C | G | intronic | - | - |
| 1 | 216800673 | 216800673 | A | T | intronic | - | - |
| 1 | 216800682 | 216800682 | C | T | intronic | - | - |
| 1 | 216800707 | 216800707 | T | A | intronic | - | - |
| 1 | 216800858 | 216800858 | T | C | intronic | - | - |
| 1 | 216800961 | 216800961 | A | G | intronic | - | - |
| 1 | 216801013 | 216801013 | T | A | intronic | - | - |
| 1 | 216801127 | 216801127 | C | T | intronic | - | - |
| 1 | 216801139 | 216801139 | A | C | intronic | - | - |
| 1 | 216801171 | 216801171 | G | T | intronic | - | - |
| 1 | 216801185 | 216801185 | C | A | intronic | - | - |
| 1 | 216801213 | 216801213 | T | C | intronic | - | - |
| 1 | 216801244 | 216801244 | C | T | intronic | - | - |
| 1 | 216801261 | 216801261 | A | T | intronic | - | - |
| 1 | 216801398 | 216801398 | T | A | intronic | - | - |
| 1 | 216801443 | 216801443 | T | A | intronic | - | - |
| 1 | 216801491 | 216801491 | A | G | intronic | - | - |
| 1 | 216801558 | 216801558 | A | G | intronic | - | - |
| 1 | 216801563 | 216801563 | A | G | intronic | - | - |
| 1 | 216801600 | 216801600 | T | A | intronic | - | - |
| 1 | 216801604 | 216801604 | A | T | intronic | - | - |
| 1 | 216801611 | 216801611 | A | T | intronic | - | - |
| 1 | 216801672 | 216801672 | T | C | intronic | - | - |
| 1 | 216801735 | 216801735 | A | C | intronic | - | - |
| 1 | 216801776 | 216801776 | T | C | intronic | - | - |
| 1 | 216801777 | 216801777 | T | C | intronic | - | - |
| 1 | 216801796 | 216801796 | C | G | intronic | - | - |
| 1 | 216801902 | 216801902 | A | G | intronic | - | - |
| 1 | 216801923 | 216801923 | C | T | intronic | - | - |
| 1 | 216802029 | 216802029 | A | C | intronic | - | - |
| 1 | 216802032 | 216802032 | T | G | intronic | - | - |
| 1 | 216802036 | 216802036 | A | G | intronic | - | - |
| 1 | 216802046 | 216802046 | A | C | intronic | - | - |
| 1 | 216802052 | 216802052 | T | C | intronic | - | - |
| 1 | 216802053 | 216802053 | G | A | intronic | - | - |
| 1 | 216802112 | 216802112 | G | C | intronic | - | - |
| 1 | 216802149 | 216802149 | A | G | intronic | - | - |
| 1 | 216802204 | 216802204 | T | C | intronic | - | - |
| 1 | 216802227 | 216802227 | C | T | intronic | - | - |
| 1 | 216802268 | 216802268 | A | C | intronic | - | - |
| 1 | 216802302 | 216802302 | C | A | intronic | - | - |
| 1 | 216802353 | 216802353 | A | G | intronic | - | - |
| 1 | 216802354 | 216802354 | T | C | intronic | - | - |
| 1 | 216802497 | 216802497 | T | C | intronic | - | - |
| 1 | 216802498 | 216802498 | G | A | intronic | - | - |
| 1 | 216802516 | 216802516 | C | T | intronic | - | - |
| 1 | 216802556 | 216802556 | A | G | intronic | - | - |
| 1 | 216802580 | 216802580 | G | A | intronic | - | - |
| 1 | 216802656 | 216802656 | C | A | intronic | - | - |
| 1 | 216802658 | 216802658 | G | C | intronic | - | - |
| 1 | 216802659 | 216802659 | G | A | intronic | - | - |
| 1 | 216802752 | 216802752 | G | T | intronic | - | - |
| 1 | 216802790 | 216802790 | C | A | intronic | - | - |
| 1 | 216802800 | 216802800 | T | C | intronic | - | - |
| 1 | 216802896 | 216802896 | C | A | intronic | - | - |
| 1 | 216802937 | 216802937 | T | C | intronic | - | - |
| 1 | 216802947 | 216802947 | C | T | intronic | - | - |
| 1 | 216802950 | 216802950 | G | T | intronic | - | - |
| 1 | 216802991 | 216802991 | G | C | intronic | - | - |
| 1 | 216803044 | 216803044 | C | T | intronic | - | - |
| 1 | 216803105 | 216803105 | C | A | intronic | - | - |
| 1 | 216803110 | 216803110 | T | C | intronic | - | - |
| 1 | 216803148 | 216803148 | A | C | intronic | - | - |
| 1 | 216803181 | 216803181 | G | T | intronic | - | - |
| 1 | 216803227 | 216803227 | C | G | intronic | - | - |
| 1 | 216803232 | 216803232 | T | A | intronic | - | - |
| 1 | 216803443 | 216803443 | A | C | intronic | - | - |
| 1 | 216803543 | 216803543 | A | G | intronic | - | - |
| 1 | 216803556 | 216803556 | A | G | intronic | - | - |
| 1 | 216803572 | 216803572 | C | T | intronic | - | - |
| 1 | 216804220 | 216804220 | T | C | intronic | - | - |
| 1 | 216804248 | 216804248 | A | G | intronic | - | - |
| 1 | 216804374 | 216804374 | C | T | intronic | - | - |
| 1 | 216804385 | 216804385 | T | C | intronic | - | - |
| 1 | 216804402 | 216804402 | T | G | intronic | - | - |
| 1 | 216804406 | 216804406 | T | G | intronic | - | - |
| 1 | 216804499 | 216804499 | T | A | intronic | - | - |
| 1 | 216804534 | 216804534 | T | C | intronic | - | - |
| 1 | 216804541 | 216804541 | C | G | intronic | - | - |
| 1 | 216804568 | 216804568 | A | T | intronic | - | - |
| 1 | 216804605 | 216804605 | C | T | intronic | - | - |
| 1 | 216804616 | 216804616 | G | A | intronic | - | - |
| 1 | 216804718 | 216804718 | C | T | intronic | - | - |
| 1 | 216804726 | 216804726 | G | T | intronic | - | - |
| 1 | 216804877 | 216804877 | A | G | intronic | - | - |
| 1 | 216804881 | 216804881 | G | C | intronic | - | - |
| 1 | 216804889 | 216804889 | A | T | intronic | - | - |
| 1 | 216805052 | 216805052 | C | T | intronic | - | - |
| 1 | 216805141 | 216805141 | T | A | intronic | - | - |
| 1 | 216805176 | 216805176 | G | T | intronic | - | - |
| 1 | 216805254 | 216805254 | C | G | intronic | - | - |
| 1 | 216805258 | 216805258 | A | C | intronic | - | - |
| 1 | 216805320 | 216805320 | A | G | intronic | - | - |
| 1 | 216805333 | 216805333 | A | C | intronic | - | - |
| 1 | 216805379 | 216805379 | G | A | intronic | - | - |
| 1 | 216805446 | 216805446 | T | G | intronic | - | - |
| 1 | 216805581 | 216805581 | T | C | intronic | - | - |
| 1 | 216805602 | 216805602 | G | T | intronic | - | - |
| 1 | 216805641 | 216805641 | G | C | intronic | - | - |
| 1 | 216805745 | 216805745 | T | C | intronic | - | - |
| 1 | 216805760 | 216805760 | A | G | intronic | - | - |
| 1 | 216805764 | 216805764 | G | T | intronic | - | - |
| 1 | 216805798 | 216805798 | G | A | intronic | - | - |
| 1 | 216805809 | 216805809 | A | G | intronic | - | - |
| 1 | 216805955 | 216805955 | C | T | intronic | - | - |
| 1 | 216805960 | 216805960 | G | C | intronic | - | - |
| 1 | 216805982 | 216805982 | T | C | intronic | - | - |
| 1 | 216806051 | 216806051 | G | A | intronic | - | - |
| 1 | 216806081 | 216806081 | T | G | intronic | - | - |
| 1 | 216806108 | 216806108 | T | A | intronic | - | - |
| 1 | 216806134 | 216806134 | T | C | intronic | - | - |
| 1 | 216806351 | 216806351 | A | T | intronic | - | - |
| 1 | 216806407 | 216806407 | T | C | intronic | - | - |
| 1 | 216806417 | 216806417 | A | C | intronic | - | - |
| 1 | 216806458 | 216806458 | T | C | intronic | - | - |
| 1 | 216806463 | 216806463 | T | G | intronic | - | - |
| 1 | 216806495 | 216806495 | A | T | intronic | - | - |
| 1 | 216806523 | 216806523 | C | T | intronic | - | - |
| 1 | 216806636 | 216806636 | A | T | intronic | - | - |
| 1 | 216806637 | 216806637 | C | T | intronic | - | - |
| 1 | 216806652 | 216806652 | A | C | intronic | - | - |
| 1 | 216806752 | 216806752 | G | A | intronic | - | - |
| 1 | 216806776 | 216806776 | C | T | intronic | - | - |
| 1 | 216806805 | 216806805 | T | C | intronic | - | - |
| 1 | 216806831 | 216806831 | T | G | intronic | - | - |
| 1 | 216806847 | 216806847 | C | A | intronic | - | - |
| 1 | 216806861 | 216806861 | A | C | intronic | - | - |
| 1 | 216806913 | 216806913 | G | A | intronic | - | - |
| 1 | 216806961 | 216806961 | T | C | intronic | - | - |
| 1 | 216807023 | 216807023 | T | A | intronic | - | - |
| 1 | 216807113 | 216807113 | T | C | intronic | - | - |
| 1 | 216807169 | 216807169 | C | T | intronic | - | - |
| 1 | 216807264 | 216807264 | T | C | intronic | - | - |
| 1 | 216807318 | 216807318 | A | T | intronic | - | - |
| 1 | 216807326 | 216807326 | T | C | intronic | - | - |
| 1 | 216807352 | 216807352 | A | G | intronic | - | - |
| 1 | 216807439 | 216807439 | T | C | intronic | - | - |
| 1 | 216807444 | 216807444 | A | G | intronic | - | - |
| 1 | 216807455 | 216807455 | C | T | intronic | - | - |
| 1 | 216807457 | 216807457 | A | G | intronic | - | - |
| 1 | 216807469 | 216807469 | T | C | intronic | - | - |
| 1 | 216807485 | 216807485 | C | T | intronic | - | - |
| 1 | 216807599 | 216807599 | T | C | intronic | - | - |
| 1 | 216807677 | 216807677 | G | C | intronic | - | - |
| 1 | 216807700 | 216807700 | A | G | intronic | - | - |
| 1 | 216807724 | 216807724 | G | A | intronic | - | - |
| 1 | 216807833 | 216807833 | C | T | intronic | - | - |
| 1 | 216807869 | 216807869 | A | C | intronic | - | - |
| 1 | 216807870 | 216807870 | G | A | intronic | - | - |
| 1 | 216807906 | 216807906 | G | A | intronic | - | - |
| 1 | 216807929 | 216807929 | A | G | intronic | - | - |
| 1 | 216808014 | 216808014 | A | G | intronic | - | - |
| 1 | 216808033 | 216808033 | C | T | intronic | - | - |
| 1 | 216808074 | 216808074 | G | A | intronic | - | - |
| 1 | 216808104 | 216808104 | A | C | intronic | - | - |
| 1 | 216808128 | 216808128 | C | A | intronic | - | - |
| 1 | 216808145 | 216808145 | A | G | intronic | - | - |
| 1 | 216808174 | 216808174 | A | T | intronic | - | - |
| 1 | 216808233 | 216808233 | G | A | intronic | - | - |
| 1 | 216808325 | 216808325 | C | A | intronic | - | - |
| 1 | 216808500 | 216808500 | C | A | intronic | - | - |
| 1 | 216808538 | 216808538 | G | A | intronic | - | - |
| 1 | 216808559 | 216808559 | C | G | intronic | - | - |
| 1 | 216808597 | 216808597 | A | C | intronic | - | - |
| 1 | 216808625 | 216808625 | C | T | intronic | - | - |
| 1 | 216808672 | 216808672 | G | T | intronic | - | - |
| 1 | 216808719 | 216808719 | C | T | intronic | - | - |
| 1 | 216808768 | 216808768 | C | T | intronic | - | - |
| 1 | 216809076 | 216809076 | G | A | intronic | - | - |
| 1 | 216809099 | 216809099 | A | T | intronic | - | - |
| 1 | 216809179 | 216809179 | T | C | intronic | - | - |
| 1 | 216809275 | 216809275 | G | C | intronic | - | - |
| 1 | 216809317 | 216809317 | A | G | intronic | - | - |
| 1 | 216809409 | 216809409 | C | A | intronic | - | - |
| 1 | 216809456 | 216809456 | C | A | intronic | - | - |
| 1 | 216809485 | 216809485 | A | G | intronic | - | - |
| 1 | 216809663 | 216809663 | T | G | intronic | - | - |
| 1 | 216809730 | 216809730 | G | T | intronic | - | - |
| 1 | 216809731 | 216809731 | G | A | intronic | - | - |
| 1 | 216809741 | 216809741 | A | G | intronic | - | - |
| 1 | 216809807 | 216809807 | A | G | intronic | - | - |
| 1 | 216809808 | 216809808 | G | A | intronic | - | - |
| 1 | 216809906 | 216809906 | G | A | intronic | - | - |
| 1 | 216810421 | 216810421 | G | T | intronic | - | - |
| 1 | 216810475 | 216810475 | A | G | intronic | - | - |
| 1 | 216810503 | 216810503 | C | T | intronic | - | - |
| 1 | 216810701 | 216810701 | C | A | intronic | - | - |
| 1 | 216810784 | 216810784 | G | A | intronic | - | - |
| 1 | 216810921 | 216810921 | T | C | intronic | - | - |
| 1 | 216810971 | 216810971 | A | G | intronic | - | - |
| 1 | 216811129 | 216811129 | G | A | intronic | - | - |
| 1 | 216811288 | 216811288 | G | A | intronic | - | - |
| 1 | 216811300 | 216811300 | A | T | intronic | - | - |
| 1 | 216811314 | 216811314 | T | C | intronic | - | - |
| 1 | 216811394 | 216811394 | C | T | intronic | - | - |
| 1 | 216811403 | 216811403 | G | A | intronic | - | - |
| 1 | 216811513 | 216811513 | A | T | intronic | - | - |
| 1 | 216811546 | 216811546 | G | A | intronic | - | - |
| 1 | 216811595 | 216811595 | T | A | intronic | - | - |
| 1 | 216811603 | 216811603 | G | A | intronic | - | - |
| 1 | 216811680 | 216811680 | C | A | intronic | - | - |
| 1 | 216811691 | 216811691 | T | C | intronic | - | - |
| 1 | 216811768 | 216811768 | T | G | intronic | - | - |
| 1 | 216811780 | 216811780 | T | C | intronic | - | - |
| 1 | 216811854 | 216811854 | G | A | intronic | - | - |
| 1 | 216811898 | 216811898 | T | C | intronic | - | - |
| 1 | 216811954 | 216811954 | C | T | intronic | - | - |
| 1 | 216811989 | 216811989 | G | T | intronic | - | - |
| 1 | 216812056 | 216812056 | A | G | intronic | - | - |
| 1 | 216812097 | 216812097 | T | C | intronic | - | - |
| 1 | 216812125 | 216812125 | G | A | intronic | - | - |
| 1 | 216812130 | 216812130 | T | C | intronic | - | - |
| 1 | 216812167 | 216812167 | A | G | intronic | - | - |
| 1 | 216812193 | 216812193 | T | C | intronic | - | - |
| 1 | 216812216 | 216812216 | C | T | intronic | - | - |
| 1 | 216812235 | 216812235 | C | T | intronic | - | - |
| 1 | 216812459 | 216812459 | T | G | intronic | - | - |
| 1 | 216812477 | 216812477 | C | T | intronic | - | - |
| 1 | 216812515 | 216812515 | T | A | intronic | - | - |
| 1 | 216812527 | 216812527 | A | G | intronic | - | - |
| 1 | 216812540 | 216812540 | A | G | intronic | - | - |
| 1 | 216812615 | 216812615 | G | A | intronic | - | - |
| 1 | 216812626 | 216812626 | T | C | intronic | - | - |
| 1 | 216812875 | 216812875 | A | G | intronic | - | - |
| 1 | 216812947 | 216812947 | A | G | intronic | - | - |
| 1 | 216812963 | 216812963 | C | T | intronic | - | - |
| 1 | 216813001 | 216813001 | C | A | intronic | - | - |
| 1 | 216813022 | 216813022 | G | A | intronic | - | - |
| 1 | 216813152 | 216813152 | C | T | intronic | - | - |
| 1 | 216813328 | 216813328 | G | A | intronic | - | - |
| 1 | 216813362 | 216813362 | T | C | intronic | - | - |
| 1 | 216813473 | 216813473 | T | C | intronic | - | - |
| 1 | 216813518 | 216813518 | C | A | intronic | - | - |
| 1 | 216813521 | 216813521 | G | A | intronic | - | - |
| 1 | 216813861 | 216813861 | T | C | intronic | - | - |
| 1 | 216813947 | 216813947 | A | G | intronic | - | - |
| 1 | 216814046 | 216814046 | A | G | intronic | - | - |
| 1 | 216814206 | 216814206 | G | A | intronic | - | - |
| 1 | 216814336 | 216814336 | G | A | intronic | - | - |
| 1 | 216814347 | 216814347 | A | G | intronic | - | - |
| 1 | 216814348 | 216814348 | C | T | intronic | - | - |
| 1 | 216814374 | 216814374 | A | G | intronic | - | - |
| 1 | 216814557 | 216814557 | T | C | intronic | - | - |
| 1 | 216814583 | 216814583 | C | T | intronic | - | - |
| 1 | 216814627 | 216814627 | T | A | intronic | - | - |
| 1 | 216814764 | 216814764 | C | A | intronic | - | - |
| 1 | 216814802 | 216814802 | A | G | intronic | - | - |
| 1 | 216814999 | 216814999 | T | C | intronic | - | - |
| 1 | 216815007 | 216815007 | G | A | intronic | - | - |
| 1 | 216815099 | 216815099 | G | A | intronic | - | - |
| 1 | 216815100 | 216815100 | T | A | intronic | - | - |
| 1 | 216815133 | 216815133 | T | C | intronic | - | - |
| 1 | 216815166 | 216815166 | G | A | intronic | - | - |
| 1 | 216815210 | 216815210 | A | T | intronic | - | - |
| 1 | 216815250 | 216815250 | C | T | intronic | - | - |
| 1 | 216815264 | 216815264 | A | C | intronic | - | - |
| 1 | 216815304 | 216815304 | T | G | intronic | - | - |
| 1 | 216815333 | 216815333 | A | C | intronic | - | - |
| 1 | 216815428 | 216815428 | T | G | intronic | - | - |
| 1 | 216815470 | 216815470 | C | T | intronic | - | - |
| 1 | 216815494 | 216815494 | T | C | intronic | - | - |
| 1 | 216815521 | 216815521 | C | A | intronic | - | - |
| 1 | 216815525 | 216815525 | T | G | intronic | - | - |
| 1 | 216815860 | 216815860 | T | A | intronic | - | - |
| 1 | 216815876 | 216815876 | A | G | intronic | - | - |
| 1 | 216815877 | 216815877 | T | C | intronic | - | - |
| 1 | 216815904 | 216815904 | A | G | intronic | - | - |
| 1 | 216815932 | 216815932 | G | A | intronic | - | - |
| 1 | 216816001 | 216816001 | A | T | intronic | - | - |
| 1 | 216816115 | 216816115 | A | T | intronic | - | - |
| 1 | 216816156 | 216816156 | A | G | intronic | - | - |
| 1 | 216816172 | 216816172 | A | G | intronic | - | - |
| 1 | 216816185 | 216816185 | T | C | intronic | - | - |
| 1 | 216816251 | 216816251 | T | C | intronic | - | - |
| 1 | 216816422 | 216816422 | T | C | intronic | - | - |
| 1 | 216816491 | 216816491 | G | T | intronic | - | - |
| 1 | 216816506 | 216816506 | C | T | intronic | - | - |
| 1 | 216816657 | 216816657 | A | G | intronic | - | - |
| 1 | 216816700 | 216816700 | G | A | intronic | - | - |
| 1 | 216816828 | 216816828 | G | A | intronic | - | - |
| 1 | 216816924 | 216816924 | A | G | intronic | - | - |
| 1 | 216816955 | 216816955 | C | T | intronic | - | - |
| 1 | 216817003 | 216817003 | G | A | intronic | - | - |
| 1 | 216817014 | 216817014 | G | T | intronic | - | - |
| 1 | 216817206 | 216817206 | C | T | intronic | - | - |
| 1 | 216817222 | 216817222 | C | T | intronic | - | - |
| 1 | 216817277 | 216817277 | C | T | intronic | - | - |
| 1 | 216817391 | 216817391 | C | T | intronic | - | - |
| 1 | 216817393 | 216817393 | T | A | intronic | - | - |
| 1 | 216817435 | 216817435 | C | T | intronic | - | - |
| 1 | 216817461 | 216817461 | A | G | intronic | - | - |
| 1 | 216817513 | 216817513 | A | G | intronic | - | - |
| 1 | 216817608 | 216817608 | A | C | intronic | - | - |
| 1 | 216817666 | 216817666 | C | T | intronic | - | - |
| 1 | 216817824 | 216817824 | G | A | intronic | - | - |
| 1 | 216817836 | 216817836 | A | G | intronic | - | - |
| 1 | 216817846 | 216817846 | A | G | intronic | - | - |
| 1 | 216817946 | 216817946 | C | T | intronic | - | - |
| 1 | 216817992 | 216817992 | G | A | intronic | - | - |
| 1 | 216818042 | 216818042 | T | C | intronic | - | - |
| 1 | 216818314 | 216818314 | C | T | intronic | - | - |
| 1 | 216818344 | 216818344 | G | A | intronic | - | - |
| 1 | 216818355 | 216818355 | G | C | intronic | - | - |
| 1 | 216818479 | 216818479 | C | T | intronic | - | - |
| 1 | 216818517 | 216818517 | T | C | intronic | - | - |
| 1 | 216818561 | 216818561 | G | A | intronic | - | - |
| 1 | 216818636 | 216818636 | A | G | intronic | - | - |
| 1 | 216818701 | 216818701 | G | A | intronic | - | - |
| 1 | 216818746 | 216818746 | C | T | intronic | - | - |
| 1 | 216818837 | 216818837 | A | C | intronic | - | - |
| 1 | 216818857 | 216818857 | T | C | intronic | - | - |
| 1 | 216818881 | 216818881 | A | C | intronic | - | - |
| 1 | 216818988 | 216818988 | C | G | intronic | - | - |
| 1 | 216819033 | 216819033 | A | G | intronic | - | - |
| 1 | 216819276 | 216819276 | G | A | intronic | - | - |
| 1 | 216819308 | 216819308 | A | G | intronic | - | - |
| 1 | 216819404 | 216819404 | A | T | intronic | - | - |
| 1 | 216819430 | 216819430 | A | G | intronic | - | - |
| 1 | 216819599 | 216819599 | C | G | exonic | nonsynonymous SNV | ncbi_100158105:XM_001926013.5:exon2:c.C403G:p.Q135E, |

**Table S15.** The information of variants located on MAPK1

| #chr | start | end | ref | alt | structure_type | function_type | function_gene |
| --- | --- | --- | --- | --- | --- | --- | --- |
| 14 | 50083246 | 50083246 | C | T | exonic | synonymous SNV | ncbi_100153927:XM_021071922.1:exon1:c.C144T:p.L48L, |
| 14 | 50084000 | 50084000 | C | G | intronic | - | - |
| 14 | 50086634 | 50086634 | G | A | intronic | - | - |
| 14 | 50087022 | 50087022 | C | T | intronic | - | - |
| 14 | 50088109 | 50088109 | C | T | intronic | - | - |
| 14 | 50088265 | 50088265 | A | G | intronic | - | - |
| 14 | 50088866 | 50088866 | G | A | intronic | - | - |
| 14 | 50089759 | 50089759 | G | A | intronic | - | - |
| 14 | 50092061 | 50092061 | A | T | intronic | - | - |
| 14 | 50096603 | 50096603 | C | T | intronic | - | - |
| 14 | 50104171 | 50104171 | A | G | intronic | - | - |
| 14 | 50105458 | 50105458 | G | A | intronic | - | - |
| 14 | 50107259 | 50107259 | C | T | intronic | - | - |
| 14 | 50107266 | 50107266 | C | T | intronic | - | - |
| 14 | 50110462 | 50110462 | G | A | intronic | - | - |
| 14 | 50111672 | 50111672 | A | G | intronic | - | - |
| 14 | 50112647 | 50112647 | G | A | intronic | - | - |
| 14 | 50113218 | 50113218 | G | A | intronic | - | - |
| 14 | 50113277 | 50113277 | A | T | intronic | - | - |
| 14 | 50115931 | 50115931 | C | T | intronic | - | - |
| 14 | 50118463 | 50118463 | G | A | intronic | - | - |
| 14 | 50119775 | 50119775 | G | A | intronic | - | - |
| 14 | 50121558 | 50121558 | A | G | intronic | - | - |
| 14 | 50122454 | 50122454 | G | A | intronic | - | - |
| 14 | 50122902 | 50122902 | C | T | intronic | - | - |
| 14 | 50122937 | 50122937 | G | A | intronic | - | - |
| 14 | 50123784 | 50123784 | G | T | intronic | - | - |
| 14 | 50124563 | 50124563 | G | A | intronic | - | - |
| 14 | 50125640 | 50125640 | G | A | intronic | - | - |
| 14 | 50127750 | 50127750 | G | A | intronic | - | - |
| 14 | 50128327 | 50128327 | C | G | intronic | - | - |
| 14 | 50130809 | 50130809 | G | A | intronic | - | - |
| 14 | 50132649 | 50132649 | C | A | intronic | - | - |
| 14 | 50132667 | 50132667 | A | G | intronic | - | - |
| 14 | 50134551 | 50134551 | G | A | intronic | - | - |
| 14 | 50134941 | 50134941 | T | C | intronic | - | - |
| 14 | 50137193 | 50137193 | G | T | intronic | - | - |
| 14 | 50137253 | 50137253 | T | A | intronic | - | - |
| 14 | 50142415 | 50142415 | G | A | intronic | - | - |
| 14 | 50144406 | 50144406 | G | A | intronic | - | - |
| 14 | 50144480 | 50144480 | G | A | intronic | - | - |
| 14 | 50144637 | 50144637 | G | A | intronic | - | - |
| 14 | 50147924 | 50147924 | A | G | intronic | - | - |
| 14 | 50148260 | 50148260 | A | G | intronic | - | - |
| 14 | 50151967 | 50151967 | A | G | intronic | - | - |
| 14 | 50154976 | 50154976 | T | C | intronic | - | - |
| 14 | 50157003 | 50157003 | C | T | intronic | - | - |
| 14 | 50158521 | 50158521 | G | A | intronic | - | - |
| 14 | 50161327 | 50161327 | C | T | intronic | - | - |
| 14 | 50162902 | 50162902 | G | A | intronic | - | - |
| 14 | 50164317 | 50164317 | A | G | UTR3 | - | - |
| 14 | 50165102 | 50165102 | C | T | UTR3 | - | - |

**Table S16.** The information of variants located on SNX19

| #chr | start | end | ref | alt | structure_type | function_type | function_gene |
| --- | --- | --- | --- | --- | --- | --- | --- |
| 9 | 57519020 | 57519020 | T | C | exonic | nonsynonymous SNV | ncbi_100519207:XM_013979586.2:exon11:c.A1037G:p.N346S |
| 9 | 57547921 | 57547921 | G | A | exonic | nonsynonymous SNV | ncbi_100519207:XM_013979586.2:exon7:c.C487T:p.P163S |
| 9 | 57552682 | 57552682 | C | A | exonic | nonsynonymous SNV | ncbi_100519207:XM_013979586.2:exon4:c.G94T:p.V32L |
| 9 | 57554131 | 57554131 | G | A | exonic | nonsynonymous SNV | ncbi_100519207:XM_021063163.1:exon2:c.C1834T:p.L612F |
| 9 | 57557825 | 57557825 | C | G | exonic | nonsynonymous SNV | ncbi_100519207:XM_021063163.1:exon1:c.G37C:p.A13P |
| 9 | 57519193 | 57519193 | G | A | intronic | - | - |
| 9 | 57519639 | 57519639 | A | G | intronic | - | - |
| 9 | 57523319 | 57523319 | T | G | intronic | - | - |
| 9 | 57523922 | 57523922 | A | T | intronic | - | - |
| 9 | 57523923 | 57523923 | C | A | intronic | - | - |
| 9 | 57527440 | 57527440 | G | A | intronic | - | - |
| 9 | 57528917 | 57528917 | A | G | intronic | - | - |
| 9 | 57529916 | 57529916 | C | T | intronic | - | - |
| 9 | 57530638 | 57530638 | G | A | intronic | - | - |
| 9 | 57533329 | 57533329 | A | C | intronic | - | - |
| 9 | 57535226 | 57535226 | C | T | intronic | - | - |
| 9 | 57536143 | 57536143 | G | A | intronic | - | - |
| 9 | 57536414 | 57536414 | T | C | intronic | - | - |
| 9 | 57536534 | 57536534 | G | A | intronic | - | - |
| 9 | 57536825 | 57536825 | G | A | intronic | - | - |
| 9 | 57536903 | 57536903 | A | G | intronic | - | - |
| 9 | 57537004 | 57537004 | T | C | intronic | - | - |
| 9 | 57537017 | 57537017 | T | A | intronic | - | - |
| 9 | 57537302 | 57537302 | T | C | intronic | - | - |
| 9 | 57537336 | 57537336 | A | C | intronic | - | - |
| 9 | 57537472 | 57537472 | T | C | intronic | - | - |
| 9 | 57537518 | 57537518 | T | C | intronic | - | - |
| 9 | 57537932 | 57537932 | C | T | intronic | - | - |
| 9 | 57538029 | 57538029 | C | T | intronic | - | - |
| 9 | 57538035 | 57538035 | T | C | intronic | - | - |
| 9 | 57538061 | 57538061 | G | C | intronic | - | - |
| 9 | 57539218 | 57539218 | G | A | intronic | - | - |
| 9 | 57539240 | 57539240 | T | A | intronic | - | - |
| 9 | 57539460 | 57539460 | A | G | intronic | - | - |
| 9 | 57539494 | 57539494 | T | C | intronic | - | - |
| 9 | 57539789 | 57539789 | C | T | intronic | - | - |
| 9 | 57539982 | 57539982 | G | A | intronic | - | - |
| 9 | 57540454 | 57540454 | G | T | intronic | - | - |
| 9 | 57540726 | 57540726 | T | C | intronic | - | - |
| 9 | 57540940 | 57540940 | A | C | intronic | - | - |
| 9 | 57541125 | 57541125 | T | A | intronic | - | - |
| 9 | 57541761 | 57541761 | G | A | intronic | - | - |
| 9 | 57541811 | 57541811 | C | T | intronic | - | - |
| 9 | 57541995 | 57541995 | C | A | intronic | - | - |
| 9 | 57543676 | 57543676 | G | A | intronic | - | - |
| 9 | 57543973 | 57543973 | A | T | intronic | - | - |
| 9 | 57546815 | 57546815 | G | A | intronic | - | - |
| 9 | 57547412 | 57547412 | A | G | intronic | - | - |
| 9 | 57547594 | 57547594 | G | A | intronic | - | - |
| 9 | 57548093 | 57548093 | T | C | intronic | - | - |
| 9 | 57548433 | 57548433 | A | G | intronic | - | - |
| 9 | 57548877 | 57548877 | C | A | intronic | - | - |
| 9 | 57548960 | 57548960 | C | A | intronic | - | - |
| 9 | 57549476 | 57549476 | C | T | intronic | - | - |
| 9 | 57550060 | 57550060 | G | C | intronic | - | - |
| 9 | 57550418 | 57550418 | G | A | intronic | - | - |
| 9 | 57550882 | 57550882 | G | A | intronic | - | - |
| 9 | 57551286 | 57551286 | T | C | intronic | - | - |
| 9 | 57551359 | 57551359 | T | C | intronic | - | - |
| 9 | 57552453 | 57552453 | A | G | intronic | - | - |
| 9 | 57553115 | 57553115 | C | T | intronic | - | - |
| 9 | 57555493 | 57555493 | G | A | intronic | - | - |

**Table S17.** The information of variants located on MSRB3

| #chr | start | end | ref | alt | structure_type |
| --- | --- | --- | --- | --- | --- |
| 5 | 29698376 | 29698376 | G | A | intronic |
| 5 | 29699195 | 29699195 | A | G | intronic |
| 5 | 29699844 | 29699844 | A | C | intronic |
| 5 | 29701063 | 29701063 | G | A | intronic |
| 5 | 29701533 | 29701533 | A | G | intronic |
| 5 | 29701774 | 29701774 | A | G | intronic |
| 5 | 29703044 | 29703044 | A | G | intronic |
| 5 | 29703415 | 29703415 | A | G | intronic |
| 5 | 29703865 | 29703865 | G | A | intronic |
| 5 | 29704448 | 29704448 | C | T | intronic |
| 5 | 29704583 | 29704583 | G | C | intronic |
| 5 | 29705105 | 29705105 | T | C | intronic |
| 5 | 29705855 | 29705855 | T | A | intronic |
| 5 | 29706567 | 29706567 | C | T | intronic |
| 5 | 29706885 | 29706885 | G | T | intronic |
| 5 | 29706912 | 29706912 | A | T | intronic |
| 5 | 29707444 | 29707444 | A | T | intronic |
| 5 | 29707878 | 29707878 | A | G | intronic |
| 5 | 29708247 | 29708247 | T | C | intronic |
| 5 | 29708530 | 29708530 | A | G | intronic |
| 5 | 29708542 | 29708542 | C | T | intronic |
| 5 | 29708903 | 29708903 | A | G | intronic |
| 5 | 29709126 | 29709126 | A | C | intronic |
| 5 | 29709384 | 29709384 | G | C | intronic |
| 5 | 29709934 | 29709934 | A | G | intronic |
| 5 | 29710043 | 29710043 | C | A | intronic |
| 5 | 29710328 | 29710328 | A | G | intronic |
| 5 | 29710672 | 29710672 | G | A | intronic |
| 5 | 29710674 | 29710674 | A | T | intronic |
| 5 | 29710950 | 29710950 | A | G | intronic |
| 5 | 29711180 | 29711180 | T | G | intronic |
| 5 | 29711185 | 29711185 | A | G | intronic |
| 5 | 29711243 | 29711243 | A | C | intronic |
| 5 | 29711848 | 29711848 | G | A | intronic |
| 5 | 29712977 | 29712977 | T | A | intronic |
| 5 | 29714283 | 29714283 | G | A | intronic |
| 5 | 29714334 | 29714334 | C | T | intronic |
| 5 | 29715248 | 29715248 | A | G | intronic |
| 5 | 29715294 | 29715294 | T | G | intronic |
| 5 | 29715330 | 29715330 | T | C | intronic |
| 5 | 29715452 | 29715452 | T | C | intronic |
| 5 | 29715557 | 29715557 | A | G | intronic |
| 5 | 29715925 | 29715925 | C | T | intronic |
| 5 | 29716135 | 29716135 | T | C | intronic |
| 5 | 29716157 | 29716157 | A | G | intronic |
| 5 | 29717310 | 29717310 | A | T | intronic |
| 5 | 29717311 | 29717311 | G | A | intronic |
| 5 | 29717606 | 29717606 | G | A | intronic |
| 5 | 29718230 | 29718230 | C | A | intronic |
| 5 | 29718394 | 29718394 | T | C | intronic |
| 5 | 29718419 | 29718419 | G | T | intronic |
| 5 | 29718530 | 29718530 | G | T | intronic |
| 5 | 29718959 | 29718959 | T | C | intronic |
| 5 | 29718972 | 29718972 | A | G | intronic |
| 5 | 29719019 | 29719019 | C | T | intronic |
| 5 | 29719716 | 29719716 | G | T | intronic |
| 5 | 29720945 | 29720945 | A | G | intronic |
| 5 | 29721586 | 29721586 | A | G | intronic |
| 5 | 29721889 | 29721889 | A | G | intronic |
| 5 | 29721972 | 29721972 | T | C | intronic |
| 5 | 29722011 | 29722011 | T | C | intronic |
| 5 | 29722884 | 29722884 | T | C | intronic |
| 5 | 29723111 | 29723111 | T | C | intronic |
| 5 | 29723113 | 29723113 | G | A | intronic |
| 5 | 29724992 | 29724992 | T | C | intronic |
| 5 | 29725552 | 29725552 | A | C | intronic |
| 5 | 29725618 | 29725618 | G | A | intronic |
| 5 | 29726023 | 29726023 | A | G | intronic |
| 5 | 29726076 | 29726076 | A | G | intronic |
| 5 | 29727586 | 29727586 | T | C | intronic |
| 5 | 29727698 | 29727698 | G | C | intronic |
| 5 | 29728063 | 29728063 | C | T | intronic |
| 5 | 29728526 | 29728526 | G | A | intronic |
| 5 | 29729140 | 29729140 | G | A | intronic |
| 5 | 29731363 | 29731363 | C | T | intronic |
| 5 | 29732332 | 29732332 | G | A | intronic |
| 5 | 29733088 | 29733088 | G | A | intronic |
| 5 | 29733658 | 29733658 | G | C | intronic |
| 5 | 29734019 | 29734019 | A | C | intronic |
| 5 | 29736115 | 29736115 | T | C | intronic |
| 5 | 29736683 | 29736683 | T | G | intronic |
| 5 | 29738321 | 29738321 | A | G | intronic |
| 5 | 29738849 | 29738849 | C | A | intronic |
| 5 | 29739273 | 29739273 | T | G | intronic |
| 5 | 29739801 | 29739801 | A | G | intronic |
| 5 | 29740916 | 29740916 | G | A | intronic |
| 5 | 29741061 | 29741061 | T | A | intronic |
| 5 | 29741063 | 29741063 | G | T | intronic |
| 5 | 29741065 | 29741065 | G | C | intronic |
| 5 | 29744090 | 29744090 | A | G | intronic |
| 5 | 29744143 | 29744143 | A | G | intronic |
| 5 | 29744740 | 29744740 | A | G | intronic |
| 5 | 29744761 | 29744761 | T | C | intronic |
| 5 | 29745362 | 29745362 | C | T | intronic |
| 5 | 29745783 | 29745783 | C | A | intronic |
| 5 | 29747008 | 29747008 | C | A | intronic |
| 5 | 29747012 | 29747012 | C | T | intronic |
| 5 | 29747425 | 29747425 | A | T | intronic |
| 5 | 29748320 | 29748320 | A | G | intronic |
| 5 | 29748646 | 29748646 | T | C | intronic |
| 5 | 29749130 | 29749130 | T | C | intronic |
| 5 | 29749301 | 29749301 | C | T | intronic |
| 5 | 29749352 | 29749352 | T | C | intronic |
| 5 | 29750365 | 29750365 | C | T | intronic |
| 5 | 29750441 | 29750441 | A | G | intronic |
| 5 | 29750638 | 29750638 | A | C | intronic |
| 5 | 29750681 | 29750681 | T | A | intronic |
| 5 | 29751013 | 29751013 | C | T | intronic |
| 5 | 29752112 | 29752112 | G | T | intronic |
| 5 | 29752627 | 29752627 | T | C | intronic |
| 5 | 29753139 | 29753139 | C | T | intronic |
| 5 | 29753195 | 29753195 | C | T | intronic |
| 5 | 29753703 | 29753703 | C | T | intronic |
| 5 | 29754258 | 29754258 | G | T | intronic |
| 5 | 29754809 | 29754809 | A | G | intronic |
| 5 | 29754851 | 29754851 | T | C | intronic |
| 5 | 29755223 | 29755223 | T | G | intronic |
| 5 | 29755384 | 29755384 | G | T | intronic |
| 5 | 29755680 | 29755680 | A | T | intronic |
| 5 | 29756036 | 29756036 | T | C | intronic |
| 5 | 29756129 | 29756129 | G | A | intronic |
| 5 | 29756157 | 29756157 | A | G | intronic |
| 5 | 29756167 | 29756167 | G | A | intronic |
| 5 | 29756176 | 29756176 | G | C | intronic |
| 5 | 29756202 | 29756202 | G | A | intronic |
| 5 | 29756270 | 29756270 | A | C | intronic |
| 5 | 29756292 | 29756292 | C | T | intronic |
| 5 | 29756591 | 29756591 | G | A | intronic |
| 5 | 29756614 | 29756614 | G | A | intronic |
| 5 | 29756619 | 29756619 | T | C | intronic |
| 5 | 29756676 | 29756676 | C | G | intronic |
| 5 | 29756789 | 29756789 | A | G | intronic |
| 5 | 29756892 | 29756892 | T | A | intronic |
| 5 | 29756973 | 29756973 | A | G | intronic |
| 5 | 29757028 | 29757028 | C | A | intronic |
| 5 | 29757118 | 29757118 | G | A | intronic |
| 5 | 29757338 | 29757338 | G | A | intronic |
| 5 | 29758830 | 29758830 | A | G | intronic |
| 5 | 29758921 | 29758921 | G | T | intronic |
| 5 | 29759043 | 29759043 | T | C | intronic |
| 5 | 29759044 | 29759044 | G | A | intronic |
| 5 | 29759144 | 29759144 | C | A | intronic |
| 5 | 29759278 | 29759278 | G | C | intronic |
| 5 | 29759710 | 29759710 | T | A | intronic |
| 5 | 29760047 | 29760047 | G | T | intronic |
| 5 | 29760104 | 29760104 | A | G | intronic |
| 5 | 29760148 | 29760148 | G | A | intronic |
| 5 | 29760582 | 29760582 | G | A | intronic |
| 5 | 29760906 | 29760906 | G | A | intronic |
| 5 | 29761160 | 29761160 | G | A | intronic |
| 5 | 29761339 | 29761339 | G | A | intronic |
| 5 | 29762141 | 29762141 | A | G | intronic |
| 5 | 29762810 | 29762810 | G | A | intronic |
| 5 | 29762936 | 29762936 | C | T | intronic |
| 5 | 29762948 | 29762948 | C | G | intronic |
| 5 | 29763181 | 29763181 | G | A | intronic |
| 5 | 29763400 | 29763400 | G | C | intronic |
| 5 | 29763407 | 29763407 | A | G | intronic |
| 5 | 29763419 | 29763419 | C | A | intronic |
| 5 | 29763432 | 29763432 | G | A | intronic |
| 5 | 29763458 | 29763458 | T | C | intronic |
| 5 | 29763960 | 29763960 | C | T | intronic |
| 5 | 29764037 | 29764037 | C | A | intronic |
| 5 | 29764477 | 29764477 | G | T | intronic |
| 5 | 29764807 | 29764807 | T | C | intronic |
| 5 | 29765628 | 29765628 | A | T | intronic |
| 5 | 29766825 | 29766825 | A | C | intronic |
| 5 | 29766949 | 29766949 | G | A | intronic |
| 5 | 29767189 | 29767189 | A | T | intronic |
| 5 | 29767355 | 29767355 | C | G | intronic |
| 5 | 29767559 | 29767559 | T | A | intronic |
| 5 | 29768150 | 29768150 | G | A | intronic |
| 5 | 29768755 | 29768755 | T | A | intronic |
| 5 | 29768974 | 29768974 | A | T | intronic |
| 5 | 29769303 | 29769303 | G | A | intronic |
| 5 | 29769356 | 29769356 | T | C | intronic |
| 5 | 29769481 | 29769481 | G | A | intronic |
| 5 | 29769524 | 29769524 | T | C | intronic |
| 5 | 29769598 | 29769598 | T | C | intronic |
| 5 | 29769681 | 29769681 | G | A | intronic |
| 5 | 29770148 | 29770148 | T | C | intronic |
| 5 | 29770194 | 29770194 | A | C | intronic |
| 5 | 29770226 | 29770226 | G | A | intronic |
| 5 | 29770253 | 29770253 | T | C | intronic |
| 5 | 29770544 | 29770544 | G | T | intronic |
| 5 | 29770734 | 29770734 | A | G | intronic |
| 5 | 29770810 | 29770810 | C | T | intronic |
| 5 | 29770835 | 29770835 | T | C | intronic |
| 5 | 29779522 | 29779522 | C | T | intronic |
| 5 | 29779548 | 29779548 | G | A | intronic |
| 5 | 29783468 | 29783468 | C | T | intronic |
| 5 | 29787777 | 29787777 | A | G | intronic |
| 5 | 29797334 | 29797334 | T | C | intronic |
| 5 | 29801471 | 29801471 | A | T | intronic |
| 5 | 29803911 | 29803911 | T | G | intronic |
| 5 | 29805120 | 29805120 | T | C | intronic |
| 5 | 29805860 | 29805860 | T | C | intronic |
| 5 | 29806651 | 29806651 | G | A | intronic |
| 5 | 29807477 | 29807477 | T | A | intronic |
| 5 | 29808405 | 29808405 | G | A | intronic |
| 5 | 29809064 | 29809064 | A | C | intronic |
| 5 | 29810621 | 29810621 | G | A | intronic |
| 5 | 29812180 | 29812180 | A | G | intronic |
| 5 | 29814883 | 29814883 | C | T | intronic |
| 5 | 29816100 | 29816100 | T | C | intronic |
| 5 | 29816752 | 29816752 | T | C | intronic |
| 5 | 29817446 | 29817446 | C | T | intronic |
| 5 | 29818367 | 29818367 | G | A | intronic |
| 5 | 29820880 | 29820880 | C | T | intronic |
| 5 | 29823196 | 29823196 | T | C | intronic |
| 5 | 29826656 | 29826656 | T | G | intronic |
| 5 | 29828444 | 29828444 | T | C | intronic |
| 5 | 29831326 | 29831326 | A | C | intronic |
| 5 | 29832963 | 29832963 | T | G | intronic |
| 5 | 29832972 | 29832972 | G | A | intronic |
| 5 | 29840579 | 29840579 | G | A | intronic |
| 5 | 29841538 | 29841538 | C | T | intronic |
| 5 | 29843618 | 29843618 | T | C | intronic |
| 5 | 29844591 | 29844591 | A | G | intronic |
| 5 | 29848739 | 29848739 | A | C | intronic |
| 5 | 29857505 | 29857505 | G | A | intronic |
| 5 | 29858427 | 29858427 | G | A | intronic |
| 5 | 29858812 | 29858812 | C | T | intronic |
| 5 | 29862412 | 29862412 | T | C | UTR3 |

**Table S18.** The information of variants located on IGF1R

| Chr | start | end | ref | alt | structure_type | function_type | function_gene |
| --- | --- | --- | --- | --- | --- | --- | --- |
| 1 | 137383664 | 137383664 | A | T | UTR3 | - | - |
| 1 | 137383922 | 137383922 | A | G | UTR3 | - | - |
| 1 | 137385558 | 137385558 | C | T | UTR3 | - | - |
| 1 | 137386825 | 137386825 | G | A | UTR3 | - | - |
| 1 | 137386990 | 137386990 | C | T | UTR3 | - | - |
| 1 | 137387397 | 137387397 | A | C | UTR3 | - | - |
| 1 | 137387847 | 137387847 | T | G | UTR3 | - | - |
| 1 | 137387962 | 137387962 | T | C | UTR3 | - | - |
| 1 | 137388124 | 137388124 | T | C | UTR3 | - | - |
| 1 | 137388283 | 137388283 | C | T | UTR3 | - | - |
| 1 | 137389483 | 137389483 | C | A | UTR3 | - | - |
| 1 | 137389539 | 137389539 | C | T | UTR3 | - | - |
| 1 | 137389595 | 137389595 | C | T | UTR3 | - | - |
| 1 | 137390982 | 137390982 | C | T | intronic | - | - |
| 1 | 137391542 | 137391542 | T | C | intronic | - | - |
| 1 | 137391556 | 137391556 | C | G | intronic | - | - |
| 1 | 137392058 | 137392058 | G | A | intronic | - | - |
| 1 | 137392063 | 137392063 | T | C | intronic | - | - |
| 1 | 137392097 | 137392097 | A | G | intronic | - | - |
| 1 | 137392235 | 137392235 | A | T | intronic | - | - |
| 1 | 137392302 | 137392302 | A | C | intronic | - | - |
| 1 | 137392445 | 137392445 | A | T | intronic | - | - |
| 1 | 137392457 | 137392457 | T | C | intronic | - | - |
| 1 | 137392466 | 137392466 | C | T | intronic | - | - |
| 1 | 137392507 | 137392507 | A | G | intronic | - | - |
| 1 | 137392695 | 137392695 | G | T | intronic | - | - |
| 1 | 137392716 | 137392716 | G | A | intronic | - | - |
| 1 | 137392722 | 137392722 | C | T | intronic | - | - |
| 1 | 137392888 | 137392888 | C | T | intronic | - | - |
| 1 | 137392953 | 137392953 | A | T | intronic | - | - |
| 1 | 137393215 | 137393215 | C | T | intronic | - | - |
| 1 | 137393334 | 137393334 | C | A | intronic | - | - |
| 1 | 137394491 | 137394491 | G | C | intronic | - | - |
| 1 | 137394693 | 137394693 | G | A | intronic | - | - |
| 1 | 137394793 | 137394793 | C | T | intronic | - | - |
| 1 | 137394858 | 137394858 | C | T | intronic | - | - |
| 1 | 137395103 | 137395103 | A | G | intronic | - | - |
| 1 | 137395131 | 137395131 | G | T | intronic | - | - |
| 1 | 137396084 | 137396084 | A | C | intronic | - | - |
| 1 | 137396172 | 137396172 | A | G | intronic | - | - |
| 1 | 137396250 | 137396250 | G | A | intronic | - | - |
| 1 | 137396756 | 137396756 | C | T | intronic | - | - |
| 1 | 137396812 | 137396812 | G | A | intronic | - | - |
| 1 | 137396916 | 137396916 | T | G | intronic | - | - |
| 1 | 137397027 | 137397027 | G | A | intronic | - | - |
| 1 | 137397135 | 137397135 | C | T | intronic | - | - |
| 1 | 137397252 | 137397252 | A | C | intronic | - | - |
| 1 | 137397289 | 137397289 | C | T | intronic | - | - |
| 1 | 137398055 | 137398055 | C | T | intronic | - | - |
| 1 | 137398497 | 137398497 | G | A | intronic | - | - |
| 1 | 137398557 | 137398557 | G | T | intronic | - | - |
| 1 | 137398749 | 137398749 | C | G | intronic | - | - |
| 1 | 137399108 | 137399108 | A | G | intronic | - | - |
| 1 | 137399225 | 137399225 | C | T | intronic | - | - |
| 1 | 137399287 | 137399287 | T | C | intronic | - | - |
| 1 | 137400372 | 137400372 | T | C | intronic | - | - |
| 1 | 137400586 | 137400586 | C | T | intronic | - | - |
| 1 | 137401031 | 137401031 | A | C | intronic | - | - |
| 1 | 137401808 | 137401808 | A | G | intronic | - | - |
| 1 | 137401956 | 137401956 | C | A | intronic | - | - |
| 1 | 137402015 | 137402015 | A | G | intronic | - | - |
| 1 | 137402244 | 137402244 | C | T | intronic | - | - |
| 1 | 137402285 | 137402285 | T | A | intronic | - | - |
| 1 | 137402525 | 137402525 | T | A | intronic | - | - |
| 1 | 137402534 | 137402534 | T | A | intronic | - | - |
| 1 | 137402574 | 137402574 | G | A | intronic | - | - |
| 1 | 137402786 | 137402786 | A | G | intronic | - | - |
| 1 | 137403419 | 137403419 | C | T | intronic | - | - |
| 1 | 137404499 | 137404499 | C | A | intronic | - | - |
| 1 | 137405078 | 137405078 | G | T | intronic | - | - |
| 1 | 137405925 | 137405925 | T | A | intronic | - | - |
| 1 | 137405926 | 137405926 | C | T | intronic | - | - |
| 1 | 137405928 | 137405928 | T | G | intronic | - | - |
| 1 | 137406550 | 137406550 | T | C | intronic | - | - |
| 1 | 137406698 | 137406698 | T | C | intronic | - | - |
| 1 | 137407385 | 137407385 | G | A | intronic | - | - |
| 1 | 137407483 | 137407483 | A | T | intronic | - | - |
| 1 | 137408201 | 137408201 | C | T | intronic | - | - |
| 1 | 137408699 | 137408699 | C | T | intronic | - | - |
| 1 | 137408906 | 137408906 | C | T | intronic | - | - |
| 1 | 137408949 | 137408949 | A | G | intronic | - | - |
| 1 | 137409406 | 137409406 | G | A | intronic | - | - |
| 1 | 137409509 | 137409509 | C | T | intronic | - | - |
| 1 | 137409574 | 137409574 | C | T | intronic | - | - |
| 1 | 137409661 | 137409661 | A | G | exonic | synonymous SNV | ncbi_397350:XM_021082918.1:exon16:c.T3078C:p.R1026R |
| 1 | 137409736 | 137409736 | G | A | exonic | synonymous SNV | ncbi_397350:XM_021082918.1:exon16:c.C3003T:p.V1001V |
| 1 | 137409985 | 137409985 | T | C | intronic | - | - |
| 1 | 137410105 | 137410105 | C | T | intronic | - | - |
| 1 | 137410261 | 137410261 | G | A | intronic | - | - |
| 1 | 137411657 | 137411657 | C | T | intronic | - | - |
| 1 | 137412114 | 137412114 | A | G | intronic | - | - |
| 1 | 137412406 | 137412406 | C | T | intronic | - | - |
| 1 | 137412470 | 137412470 | G | C | intronic | - | - |
| 1 | 137412856 | 137412856 | C | T | intronic | - | - |
| 1 | 137413149 | 137413149 | A | T | intronic | - | - |
| 1 | 137413212 | 137413212 | G | T | intronic | - | - |
| 1 | 137413658 | 137413658 | T | C | intronic | - | - |
| 1 | 137413799 | 137413799 | C | T | intronic | - | - |
| 1 | 137413811 | 137413811 | C | T | intronic | - | - |
| 1 | 137414135 | 137414135 | T | C | intronic | - | - |
| 1 | 137414609 | 137414609 | T | G | intronic | - | - |
| 1 | 137414939 | 137414939 | C | T | intronic | - | - |
| 1 | 137415519 | 137415519 | T | C | intronic | - | - |
| 1 | 137415935 | 137415935 | C | A | intronic | - | - |
| 1 | 137416015 | 137416015 | G | A | intronic | - | - |
| 1 | 137416070 | 137416070 | G | C | intronic | - | - |
| 1 | 137416097 | 137416097 | C | T | intronic | - | - |
| 1 | 137416314 | 137416314 | A | G | intronic | - | - |
| 1 | 137416355 | 137416355 | G | T | intronic | - | - |
| 1 | 137416748 | 137416748 | C | T | intronic | - | - |
| 1 | 137417116 | 137417116 | A | T | intronic | - | - |
| 1 | 137417157 | 137417157 | T | C | intronic | - | - |
| 1 | 137418944 | 137418944 | T | C | intronic | - | - |
| 1 | 137419108 | 137419108 | C | T | intronic | - | - |
| 1 | 137419398 | 137419398 | C | T | intronic | - | - |
| 1 | 137419440 | 137419440 | C | T | intronic | - | - |
| 1 | 137419448 | 137419448 | C | T | intronic | - | - |
| 1 | 137419532 | 137419532 | C | T | intronic | - | - |
| 1 | 137419826 | 137419826 | T | C | intronic | - | - |
| 1 | 137420022 | 137420022 | G | A | intronic | - | - |
| 1 | 137420902 | 137420902 | A | G | intronic | - | - |
| 1 | 137422068 | 137422068 | A | G | intronic | - | - |
| 1 | 137422650 | 137422650 | G | A | intronic | - | - |
| 1 | 137423137 | 137423137 | G | A | exonic | synonymous SNV | ncbi_397350:XM_021082918.1:exon11:c.C2193T:p.N731N |
| 1 | 137423412 | 137423412 | C | T | intronic | - | - |
| 1 | 137424049 | 137424049 | C | T | intronic | - | - |
| 1 | 137424200 | 137424200 | C | G | intronic | - | - |
| 1 | 137424452 | 137424452 | C | T | intronic | - | - |
| 1 | 137424581 | 137424581 | G | A | intronic | - | - |
| 1 | 137424599 | 137424599 | T | C | intronic | - | - |
| 1 | 137425934 | 137425934 | G | A | intronic | - | - |
| 1 | 137426313 | 137426313 | C | T | intronic | - | - |
| 1 | 137426365 | 137426365 | G | A | intronic | - | - |
| 1 | 137426395 | 137426395 | C | T | intronic | - | - |
| 1 | 137427265 | 137427265 | C | G | intronic | - | - |
| 1 | 137427474 | 137427474 | C | A | intronic | - | - |
| 1 | 137429025 | 137429025 | C | T | intronic | - | - |
| 1 | 137429669 | 137429669 | C | T | intronic | - | - |
| 1 | 137430806 | 137430806 | G | A | intronic | - | - |
| 1 | 137431025 | 137431025 | A | T | intronic | - | - |
| 1 | 137432536 | 137432536 | C | T | intronic | - | - |
| 1 | 137432646 | 137432646 | C | T | intronic | - | - |
| 1 | 137432891 | 137432891 | T | C | intronic | - | - |
| 1 | 137433005 | 137433005 | G | A | intronic | - | - |
| 1 | 137433137 | 137433137 | C | A | exonic | synonymous SNV | ncbi_397350:XM_021082918.1:exon7:c.G1428T:p.L476L |
| 1 | 137433198 | 137433198 | T | C | intronic | - | - |
| 1 | 137433311 | 137433311 | T | C | intronic | - | - |
| 1 | 137433813 | 137433813 | G | A | intronic | - | - |
| 1 | 137434209 | 137434209 | T | C | intronic | - | - |
| 1 | 137434275 | 137434275 | G | A | intronic | - | - |
| 1 | 137434285 | 137434285 | T | C | intronic | - | - |
| 1 | 137435255 | 137435255 | T | C | intronic | - | - |
| 1 | 137435320 | 137435320 | G | T | intronic | - | - |
| 1 | 137436137 | 137436137 | T | C | intronic | - | - |
| 1 | 137436307 | 137436307 | G | A | intronic | - | - |
| 1 | 137436522 | 137436522 | A | G | intronic | - | - |
| 1 | 137437208 | 137437208 | G | A | intronic | - | - |
| 1 | 137437213 | 137437213 | C | T | intronic | - | - |
| 1 | 137437258 | 137437258 | C | A | intronic | - | - |
| 1 | 137437479 | 137437479 | C | T | intronic | - | - |
| 1 | 137437623 | 137437623 | T | A | intronic | - | - |
| 1 | 137437862 | 137437862 | C | A | intronic | - | - |
| 1 | 137438751 | 137438751 | T | C | intronic | - | - |
| 1 | 137439684 | 137439684 | C | T | intronic | - | - |
| 1 | 137440243 | 137440243 | T | G | intronic | - | - |
| 1 | 137440460 | 137440460 | C | T | intronic | - | - |
| 1 | 137440753 | 137440753 | G | A | intronic | - | - |
| 1 | 137441478 | 137441478 | A | G | intronic | - | - |
| 1 | 137441815 | 137441815 | C | A | intronic | - | - |
| 1 | 137442395 | 137442395 | A | G | intronic | - | - |
| 1 | 137442669 | 137442669 | C | T | intronic | - | - |
| 1 | 137443737 | 137443737 | G | A | intronic | - | - |
| 1 | 137443842 | 137443842 | G | A | intronic | - | - |
| 1 | 137445348 | 137445348 | C | T | intronic | - | - |
| 1 | 137445417 | 137445417 | T | C | intronic | - | - |
| 1 | 137446262 | 137446262 | C | G | intronic | - | - |
| 1 | 137447213 | 137447213 | A | C | intronic | - | - |
| 1 | 137447281 | 137447281 | G | A | intronic | - | - |
| 1 | 137447358 | 137447358 | T | G | intronic | - | - |
| 1 | 137447414 | 137447414 | T | C | intronic | - | - |
| 1 | 137447710 | 137447710 | T | C | intronic | - | - |
| 1 | 137447716 | 137447716 | G | A | intronic | - | - |
| 1 | 137447728 | 137447728 | C | A | intronic | - | - |
| 1 | 137447740 | 137447740 | T | C | intronic | - | - |
| 1 | 137447936 | 137447936 | T | C | intronic | - | - |
| 1 | 137447961 | 137447961 | A | G | intronic | - | - |
| 1 | 137448331 | 137448331 | T | C | intronic | - | - |
| 1 | 137449000 | 137449000 | G | A | intronic | - | - |
| 1 | 137449285 | 137449285 | A | T | intronic | - | - |
| 1 | 137449395 | 137449395 | A | G | intronic | - | - |
| 1 | 137449484 | 137449484 | T | C | intronic | - | - |
| 1 | 137449485 | 137449485 | T | C | intronic | - | - |
| 1 | 137449538 | 137449538 | C | T | intronic | - | - |
| 1 | 137449748 | 137449748 | T | C | intronic | - | - |
| 1 | 137449749 | 137449749 | G | A | intronic | - | - |
| 1 | 137449993 | 137449993 | T | C | intronic | - | - |
| 1 | 137450194 | 137450194 | T | A | intronic | - | - |
| 1 | 137451333 | 137451333 | A | G | intronic | - | - |
| 1 | 137452278 | 137452278 | T | C | intronic | - | - |
| 1 | 137452401 | 137452401 | G | A | intronic | - | - |
| 1 | 137452480 | 137452480 | G | C | exonic | synonymous SNV | ncbi_397350:XM_021082918.1:exon3:c.C852G:p.G284G |
| 1 | 137452645 | 137452645 | T | C | exonic | synonymous SNV | ncbi_397350:XM_021082918.1:exon3:c.A687G:p.T229T |
| 1 | 137453221 | 137453221 | A | G | intronic | - | - |
| 1 | 137453908 | 137453908 | T | C | intronic | - | - |
| 1 | 137454021 | 137454021 | A | G | intronic | - | - |
| 1 | 137454023 | 137454023 | A | T | intronic | - | - |
| 1 | 137454430 | 137454430 | A | T | intronic | - | - |
| 1 | 137454491 | 137454491 | A | G | intronic | - | - |
| 1 | 137454552 | 137454552 | A | G | intronic | - | - |
| 1 | 137454599 | 137454599 | G | A | intronic | - | - |
| 1 | 137454615 | 137454615 | C | T | intronic | - | - |
[truncated: 42,676 more chars]
